# Establishment of a comprehensive set of fact sheets for cancer predisposition genes for medical oncologists practicing cancer genome profiling

**DOI:** 10.1007/s10147-025-02746-w

**Published:** 2025-04-04

**Authors:** Manami Matsukawa, Chikako Tomozawa, Yoshiaki Nakamura, Takao Fujisawa, Kaori Kimura, Yumie Hiraoka, Riu Yamashita, Shinji Kosugi, Akihiro Sakurai, Issei Imoto, Masakazu Nishigaki, Makoto Hirata, Takeshi Kuwata, Takayuki Yoshino

**Affiliations:** 1 Department of Genetic Medicine and Services, National Cancer Center Hospital, Tokyo, Japan; 2 Department of Genetic Medicine and Services, National Cancer Center Hospital East, Kashiwa, Japan; 3 Department of Gastroenterology and Gastrointestinal Oncology, National Cancer Center Hospital East, Kashiwa, Japan; 4 Translational Research Support Office, National Cancer Center Hospital East, Kashiwa, Japan; 5 Department of Head and Neck Medical Oncology, National Cancer Center Hospital East, Kashiwa, Japan; 6 Division of Translational Informatics, Exploratory Oncology Research and Clinical Trial Center, National Cancer Center, Kashiwa, Japan; 7 Department of Genomic Medicine, Kyoto University School of Public Health, Kyoto, Japan; 8 Department of Medical Genetics and Genomics, Sapporo Medical University School of Medicine, Sapporo, Japan; 9 Aichi Cancer Center Research Institute, Nagoya, Japan; 10 Department of Genetic Counseling, International University of Health and Welfare, Tokyo, Japan; 11 Division for the Promotion of Drug and Diagnostic Development, National Cancer Center Hospital East, Kashiwa, Japan

**Keywords:** Comprehensive genomic profiling, Cancer predisposition gene, Fact sheet, Genetic counseling

## Abstract

**Background:**

Comprehensive genomic profiling (CGP) is widely performed worldwide, increasing opportunities for medical oncologists to explain cancer predisposition at the time of informed consent and return of results. How medical oncologists communicate about (suspected) cancer predisposition genes is a key factor in referring patients for consultation with genetic services. In this study, we developed a set of fact sheets on cancer predisposition genes to support medical oncologists in their practice under the nationwide cancer genome screening project MONSTAR-SCREEN-2 study in Japan.

**Methods:**

The Genetic Specialist Committee, comprising clinical geneticists, genetic counselors, bioinformaticians, and medical oncologists, drafted the fact sheet and external Genetic Experts reviewed its elements and contents. A fact sheet evaluation survey was conducted one year after the fact sheet was completed and distributed to medical oncologists at the National Cancer Center Hospital East.

**Results:**

The content of the fact sheet included an overview of diseases, inheritance, family impact, lifetime risk, and surveillance. In the evaluation survey, 83.3% of respondents rated it as “useful.” Notably, the sections “What is genetic counseling” (100%) and “Lifetime risk” (94.4%) received high ratings.

**Conclusion:**

Our study suggests that a fact sheet developed by the Genetic Specialist Committee may help medical oncologists explain CGP results and connect patients to genetic services. It also functions as an educational resource that requires periodic updates and is in line with revisions to the guidelines.

**Supplementary Information:**

The online version contains supplementary material available at 10.1007/s10147-025-02746-w.

## Introduction

Comprehensive genomic profiling (CGP) is a standard method for cancer treatment worldwide [1]. Germline pathogenic variants (GPV) or presumed germline pathogenic variants (PGPV) can be detected in 4.1% – 17.5% of patients [2–9]. Patients with GPV/PGPV are referred to genetic services for genetic counseling, and confirmatory testing and/or cascade testing is provided [1, 10–13]. Several guidelines were reported for GPV/PGPV screening through CGP [12–17]. Koster et al. validated an effective workflow for genetic counseling referrals and concluded that using a Dutch gene list maximized the GPV yield and minimized unnecessary referrals [13]. The Japanese GPV/PGPV screening guidelines integrate germline conversion rates, clinical features, and a literature-based list of 55 genes [15, 16].

The CGP unexpectedly acts as a family health screening tool for some patients. Even in the absence of a suspected family history, the CGP can be used to identify carriers of low-penetrance genes [17–20]. For example, *BRCA1/2* often exhibits GPVs even in off-tumor cancer types [2, 6, 8, 9, 21]. Connecting to genetic services for confirmatory and cascade testing can support health management of individuals and their families. However, patients detected with GPV/PGPV are not always referred to genetic services due to poor conditions or decline [7, 8, 22, 23]. If the patient (proband) is not referred for genetic counseling, cascade testing cannot be performed and relatives may lose the opportunity to undergo genetic testing. Hence, medical oncologists play a key role in addressing the risk of cancer predisposition in the patients' families. Some medical oncologists consider educating patients about GPV/PGPV, providing detailed information on cancer predisposition genes, and explaining the implications of GPV/PGPV to patients’ family members challenging [24]. Concise and straightforward explanations of CGP test results and tools are needed to effectively address GPV/PGPV [24, 25].

Cancer predisposition gene-related information is available online. GeneReviews and ClinGen Summary Reports [26, 27] include detailed management information. Some facilities publish fact sheets translated into several languages [28, 29]. GeneReviews Japan [30] is a well-known resource translated from GeneReviews by volunteers. However, comprehensive resources for referencing genes that can be detected using the CGP are unavailable.

The MONSTAR-SCREEN-2 study conducted under the nationwide molecular profiling project SCRUM-Japan MONSTAR-SCREEN, designed to reveal GPV/PGPV through CARIS MI Profile testing, followed by single-site analysis (and multi-gene panel testing) to confirm GPV/PGPV [31–33]. Medical oncologists involved in this study would receive a report on pathogenic variants of cancer predisposition genes. This study aimed to create fact sheets on cancer predisposition genes to help non-genetic specialists explain GPV/PGPV and refer patients for genetic counseling.

## Materials and methods

### Workflow of the fact sheet development

Under MONSTAR-SCREEN-2, blood samples and tumor tissues were submitted, and medical oncologists returned a molecular profiling report of germline and somatic mutations to the patients. If GPV/PGPV was detected, it was explained to the patient by a medical oncologist, who was then referred for genetic services. To manage the GPVs/PGPVs detected in this study, a Genetic Specialist Committee was established. The Genetic Specialist Committee is composed of external evaluators and members of the MONSTAR-SCREEN-2 study. In the creation of the MONSTAR-SCREEN-2 Fact Sheet (hereafter, the “Fact Sheet”), four experienced clinical geneticists and one genetic counselor (hereafter, the “Genetic Experts”) from the research group for the research project on ethical, legal, and social issues supported by the Health, Labor, and Welfare Sciences Research Grant “Extraction of Ethical and Social Issues and Improvement of Social Environment toward the Realization of a Society Where People Can Benefit from Genome Medicine Without Anxiety” and the Actionability Working Group-Japan, who were involved in developing the Japanese GPV/PGPV screening guidelines and are not involved in the sampling or analysis of the MONSTAR-SCREEN-2 research, participated as third-party evaluators. Additionally, the MONSTAR-SCREEN-2 study team includes an experienced clinical geneticist, three leading medical oncologists, and four genetic counselors who participated in the committee. The oncologists on the committee are highly experienced and involved in educational programs. The Fact Sheet was developed from April 2021 to February 2022 and distributed to participating institutions in May 2022.

**Fig. 1:**
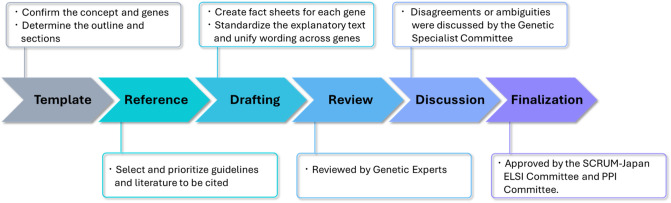
Developmental Process of the Fact Sheet. ELSI Ethical, legal, and social implications; *PPI* Patient and public involvement

The Fact Sheet was developed using the following five steps (Figure 1): (1) items included in the Fact Sheet template were determined based on existing fact sheets and medical guides [28, 29], (2) guidelines and literature were referenced for each gene (Table 1, Supplementary files 1 and 2), and a draft was created; (3) the drafts were reviewed by Genetic Experts; (4) the Genetic Specialist Committee met to discuss points of disagreement or unclear notation; and (5) the draft was finalized after review by the SCRUM-Japan, Ethical, Legal, and Social Implications Committee and Patient and Public Involvement Committee.

**Table 1:** Agreement of genetic experts for the fact sheet contents

Topic	Decision	Reason
Purpose	Handouts for medical oncologists who need to explain the PGPV variants before referring the patient to the genetic department	・ Medical oncologists are not always familiar with hereditary cancers.
Gene selection	*APC, ATM, BAP1, BARD1, BMPR1A, BRCA1, BRCA2, BRIP1, CDH1, CDK4, CDKN2A, CHEK2, EPCAM, FH, FLCN, HNF1A, MAX, MEN1, MET, MLH1, MSH2, MSH6, MUTYH, NBN, NF1, NF2, PALB2, PMS2, POLD1. POLE, POT1, PTEN, RAD51C, RAD51D, RB1, RET, SDHA, SDHAF2, SDHB, SDHC, SDHD, SMAD3, SMAD4, SMARCB1, STK11, TERF2IP, TERT, TGFBR1, TGFBR2, TMEM127, TP53, TSC1, TSC2, VHL, WT1*	・The gene list from The Japanese PGV/PGPV screening guideline: Guidelines for the Communication Process in Genomic Medicine.
References	Prioritize as follows: Japanese guideline, NCCN guideline, GeneReviews Japan or ClinGen Summary report, and GeneReviews	・Medical oncologists can access detailed, reliable information. ・ Japanese guidelines are published based on the Japanese healthcare system. ・ Not all moderate- and law-risk genes are covered in the Japanese guidelines.
Phenotype description	Describe a medically actionable cancer or tumor phenotype and adult onset or high penetrance for non-cancer phenotypes	・ This fact sheet is intended for use by medical oncologists who treat adult cancer patients
AR Phenotype description	・Explain the AR phenotype: *MUTYH* ・Not explain the AR phenotype: *ATM, BRCA2, BRIP1, EPCAM, FH, MLH1, MSH2, MSH6, NBN, PALB2, PMS2, POLD1, POLE, RAD51C, SDHA, SDHB, SDHD, TERT, VHL*	・ This fact sheet is intended for use by medical oncologists who treat adult cancer patients ・The occurrence of mutations in both alleles is very rare.
*De novo* frequency	・ Describe the *de novo* rate if there is more than one reliable report. ・GENE-specific *de novo* frequency data available: *BRCA1, BRCA2* ・ Only *de novo* DISEASE frequency data are available: *APC, BMPR1A, MEN1, NF1, NF2, SMAD3, SMAD4, RB1, RET, TGFBR1, TGFBR2, TP53, TSC1, TSC2, VHL*	・ The *de novo* data could be useful for cancer patients who have no family history but are suspected of hereditary cancer based on the CGP result.

*PGPV* presumed germline pathogenic variant, *AR* autosomal recessive, *CGP* comprehensive genomic profiling

### Fact Sheet evaluation survey

The survey questionnaire was developed based on literatures on the development of medical educational tools [34–36]. The questionnaire consisted of six sections (24 questions): (1) awareness and frequency of use, (2) overall evaluation, (3) layout and source evaluation, (4) content evaluation, (5) free comments, and (6) demographics. The web survey URL and Fact Sheet were distributed via email to medical oncologists who registered patients for the MONSTAR-SCREEN2 study at the National Cancer Center Hospital East, Kashiwa, Japan.

The survey was distributed approximately one year after the MONSTAR SCREEN-2 study started, from 27^th^ November to 5^th^ December 2023. A reminder was sent one week after the first e-mail recruitment.

Descriptive statistical analysis was conducted using Microsoft Excel, utilizing built-in functions such as frequencies, averages, and ranges, to summarize the data.

### Ethical approval

The MONSTAR-SCREEN-2 was approved by the Institutional Review Board of the National Cancer Center Hospital East (2020-496).

## Results

### Gene selection and contents of the fact sheet

The 55 genes listed in the Guidelines for the Communication Process in Genomic Medicine [15, 16] were selected for the Fact Sheet (Table 1), all of which were cancer predisposition genes except for four genes: *HNF1A*, *SMAD3*, *TGFBR1*, and *TGFBR2*. All members of the Genetic Specialist Committee agreed on the selected genes. The guidelines were intended for use in cases where CGP results indicated a PGPV of germline origin, and the genes listed in the guidelines may be medically actionable. Committee members reviewed the criteria for gene selection, determined whether the cancer predisposition genes included in the molecular profiling provided by MONSTAR-SCREEN-2 were sufficient, and agreed on the selected genes.

The contents of the Fact Sheet were discussed from the perspective of what should be explained to medical oncologists treating adult patients with suspected GPV in cancer predisposition genes. The Fact Sheet was created using genes because the CGP reports were based on gene names (Table 1). The contents of the Fact Sheet were finalized with four sections, as described in Table 2. In the first section, we have described the correlation between GPV/PGPV of cancer predisposition gene identified through CGP and corresponding cancer predisposition syndromes. In the second section, we have described what this means to patients confirmed with germline pathogenic variants, the risk of cancer/symptoms in their families, and genetic counseling. In the third section, we have stated the lifetime risk for GPV carriers and compare it with the data for non-carriers. Recommended surveillance has also been described. References are provided in the fourth section (Table 2 and Supplementary Files 1 and 2).

**Table 2:** The outline of the Fact Sheet

Section		Summary	Formats	
1	Genetic change and hereditary cancer syndrome	If you have genetic changes that are responsible for hereditary cancer syndrome, you may be at higher risk for certain types of cancer. Genetic testing from a blood sample can help determine if you have an increased risk of developing cancer.	Text	
	What is this GENE related to?	Genetic change in the GENE gene associated with X disease.	Text	
2	The benefits of confirmatory testing	Understanding your cancer risk(s) and receiving clinical follow-up care will lead you to the early detection of cancer.	Text	
	Risk of family members	Each of your children, siblings, and parents has a 50% chance of inheriting the genetic change in the GENE gene. Sharing your genetic information with your relatives may change their medical management.	Text	+ Figure
	What is Genetic Counseling?	Genetic counseling provides you with information about how genetic conditions may affect you and your family.	Text	
3	Lifetime risk and recommended management	Shows Japanese general lifetime risk, career lifetime risk, and recommended management for the cancer type.	Text	+ Table
4	Reference	Site reliable data from Japanese guidelines, NCCN guidelines, GeneReviews Japan, ClinGen Summary Report, and GeneReviews.	Text	

*NCCN* National comprehensive cancer network

### Discussion and agreement on the fact sheet descriptions

We did not include disease frequency in the Fact Sheet. In addition to the fact that germline conversion rates [12] were not available at that time, we also considered that in patients with PGPV, germline probability may outweigh disease frequency. We also intended to use plain language; for instance, we did not use the term “secondary findings” but used “PGPV.”

The references were prioritized in the following order: (1) Japanese guidelines; (2) National Comprehensive Cancer Network (NCCN) guidelines, GeneReviews Japan, or the ClinGen Summary Report; and (3) GeneReviews. When the Fact Sheet was created, 32 of the 55 genes met the Japanese guidelines. There were 3 genes that could only be referred to individually in the ClinGen Summary Report or GeneReviews. It is important to note that, overall, the lower the penetrance of cancer is, the lesser the availability of statistical data and evidence for surveillance is.

Only the actionable phenotypes are listed in the recommended surveillance table. Symptoms in adults that are neither cancerous nor actionable have been described briefly. For example, there is no medical screening recommendation for *NF1* carrier neurofibromas, so we added the following note: “In addition to the symptoms listed in Table 1, other skin, nerve, or eye-related characteristics may be detected” (Supplementary files 1 and 2). Contrastingly, actionable non-cancer symptoms such as primary hyperparathyroidism for *MEN1* were included in the surveillance table (Supplementary files 1 and 2). However, symptoms of *PTEN*, including macrocephaly and mental retardation *PTEN,* are not listed in the table because they are not medically actionable.

Since the detection rate of biallelic pathogenic variants is extremely low, including ataxia telangiectasia for *ATM* and Fanconi anemia for *BRCA1* and *BRCA2*, we did not describe the autosomal recessive mode of inheritance in the Fact Sheet, except for *MUTYH*.

We also discuss *de novo* mutation rates. Although *de novo* cases are rare, we conclude that *de novo* mutation rates provide useful information for patients who incidentally have genetic diseases, such as no family history and/or off-tumor cancer. Since there are few reliable gene-specific *de novo* data available that calculate rates based on the general population regardless of phenotype, apart from *BRCA1* and *BRCA2*, we provided the *de novo* rates for 15 genes (Table 1) with the note, “There is an approximately Y% probability that individuals diagnosed with Z syndrome did not inherit it.”

When citing data from other countries, it is important to consider differences in disease frequency between ethnic groups. For example, the incidence of malignant melanoma differs between Japanese and Caucasian populations; melanoma is a rare cancer in the Japanese population. To make it more understandable, we noted in the melanoma-related genes Fact Sheet, “The annual prevalence of malignant melanoma is reported to be 24.3 in 100,000 people for Caucasians and 1.7 in 100,000 people for Asians, and the risk is considered to vary according to race, region, and other genetic factors.”

### Fact sheet evaluation survey

Eighteen out of the 40 medical oncologists responded to the survey (45.0%), with no bias in terms of medical department and years of experience (Table 3). Six medical oncologists read the Fact Sheet before the survey, and one medical oncologist had used it in a clinic.

**Table 3:** Characteristics of the fact sheet evaluation survey respondents

Variable	n / mean	( % / range )
Department		
Gastrointestinal oncology	5	( 27.8 )
Hepatobiliary and pancreatic oncology	4	( 22.2 )
Medical oncology	4	( 22.2 )
Head and neck medical oncology	2	( 11.1 )
Experimental therapeutics	1	( 5.6 )
N/A	2	( 11.1 )
The Japanese society of clinical oncology board-certified		
Yes	7	( 38.9 )
No	10	( 55.6 )
N/A	1	( 5.6 )
The Japanese board of cancer therapy-certified		
Yes	2	( 11.1 )
No	15	( 83.3 )
N/A	1	( 5.6 )
Years of clinical experience		
Years	15.5	( 7 - 25 )

*N/A* no answer

**Fig. 2:**
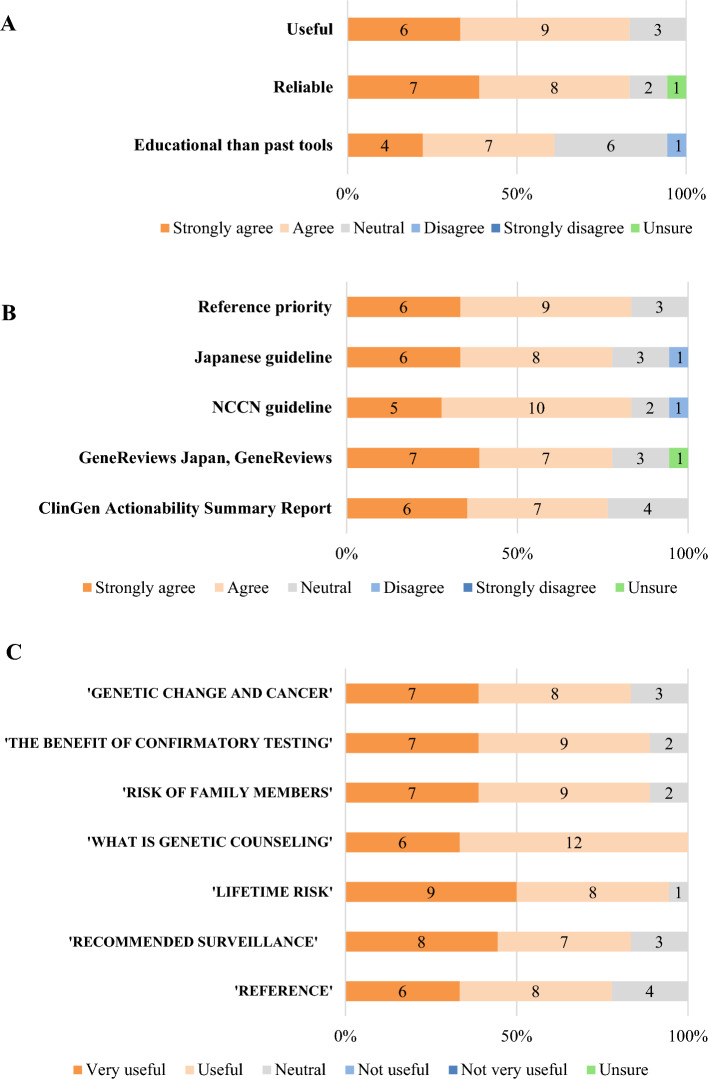
Evaluation of the Fact Sheet by oncologists (*n* =18). **A.** Overall evaluation of the Fact Sheet. **B.** Evaluation of each content. **C.** Evaluation of reference selection. *NCCN* National comprehensive cancer network

Most medical oncologists reviewed the Fact Sheet, and 15 medical oncologists (83.3%) found the gene-organized Fact Sheet “useful” with no “not useful” responses. The layouts were rated as very good (*n *= 8; 44.4%), good (*n *= 6; 33.3%), or neutral (*n *= 3; 16.7%). Reliability and usefulness were validated by 15 (83.3%) medical oncologists, and 11 (61.1%) felt that the Fact Sheet was more educational than previous tools for gathering information (Figure 2A).

All the content was rated as “very useful” or “useful” by most of the medical oncologists, with “What is genetic counseling” (*n *= 18, 100%) and “Lifetime risk” (*n *= 17, 94.4%) highly rated (Figure 2B). Fifteen medical oncologists (83.3%) rated the priority order of the references, and more than 14 medical oncologists (77.8%) agreed with each reference (Figure 2C). One of the medical oncologists commented, “*A list of all the genes or a table of contents if it is to be a booklet would be helpful*” for future Fact Sheet revisions.

## Discussion

This study created a fact sheet of genes associated with cancer predisposition and/or useful for medical oncologists in explaining the CGP results and gathering feedback. Notably, the Fact Sheet was specifically designed for each gene and tailored to adult patients with cancer. High-risk gene groups such as *BRCA1/2* have been previously documented, but resources for middle- and low-risk gene groups, such as *BAP1*, *POT1*, and *SDHx*, which can be detected through CGP, remain limited. Furthermore, this study is the first to develop a Japanese-language fact sheet of genes associated with cancer predisposition.

The Fact Sheet was created for 55 cancer predisposition and/or actionable genes by the Genetic Specialist Committee. In fact, 88.9% of the medical oncologists considered the Fact Sheet by gene “useful.” Traditionally, hereditary diseases, including cancer predisposition syndrome, are suspected based on medical symptoms, and genetic testing is conducted after explaining the disease at the first genetic counseling session, testing at the second session, and returning the results at the third visit (a three-visit model) [37]. Genetic testing of cancer predisposition genes is now moving toward a comprehensive analysis. Genetic testing is performed globally during the initial session, with the results and gene disease information explained in a two-visit model of post-test genetic counseling [13, 37]. An interview study with 24 American cancer genetic counselors found that a brief pretest education followed by post-test counseling was efficient [38]. Koster et al. showed that the rates of GPVs were high in *BRCA1, BRCA2, PALB2, MLH1, MSH2*, and *MSH6* but varied by gene. These results suggest that gene-based educational tools are crucial in the genetics-first era, highlighting the versatility of the Fact Sheet for germline testing.

More than 90% of medical oncologists found “genetic counseling” and “lifetime cancer risk” information useful. Therefore, these contents are important for explaining the implications of GPV/PGPV to patients and their relatives for connecting them to a genetic service. Consistently, a previous report showed that some medical oncologists have difficulty explaining the details of GPV/PGPV and its impact on relatives, suggesting that this gene-based Fact Sheet could be valuable in this field [24]. In the MONSTAR-SCREEN-2 study, however, the majority of GPVs were *BRCA1/2*, which oncologists are already familiar with, and Fact Sheets are not frequently referred. On the other hand, when the GPVs were identified in the gene with which oncologists are not familiar, including moderate- or low-risk genes, the oncologists would not explain the details of the variants but simply refer the patients to the genetic counseling section. In fact, not as expected, Fact Sheet became more frequently referred by genetic specialists rather than oncologists. At the National Cancer Center Hospital and East Hospital, printed and digital materials support both oncologists and genetic services, especially for explaining secondary findings and CGP-detected variants. Therefore, this Fact Sheet may have a potential to be used in genetic services as well.

Although evidence of associated cancers, frequencies, and surveillance for each gene is accumulating, middle- and low-risk genes lack data, making it difficult to create fact sheets with evidence equivalent to that of high-risk genes [39–42]. This may be because middle- and low-risk genes were tested only with comprehensive genetic testing, and the number of carriers affected by cancer was small owing to low penetrance [43–46].

As research progresses, data have been accumulated and it has become possible to provide information based on population distributions [47–49] and founder mutations [50, 51]. However, it is also important to consider differences in the types of cancers based on their frequencies in specific populations, such as the Japanese population exhibiting low frequency of malignant melanoma. Therefore, we have described the limitations of the data. Further research can determine whether the data are highly individualized or generalized.

This Fact Sheet was intended as an explanatory document for non-genetic healthcare providers who refer patients with GPV/PGPV to genetic services. Therefore, emphasis is placed on describing the core information from the clinical guidelines. Possibly due to this, only 61.1% of medical oncologists rated it as “educational” than previous tools. Chatbot AI has become a tool that helps people understand complex information easily. Moreover, ChatGPT has been shown to provide accurate information on general topics related to genetics and genetic counseling but provides nuanced and inaccurate information on the correct cancer risk and/or surveillance for hereditary diseases[52–54]. This suggests that there are limitations to obtaining information from AI-based tools and that tools including the present fact sheets will be important for determining whether the level of evidence is accurate.

Recently, active germline variant detection in CGP has been emphasized in drug selection [55, 56]. However, Kage et al. revealed that some patients did not wish to disclose germline findings because they might not have been linked to the treatment [57]. Patients and medical oncologists believe that knowing “heritability” may influence patients, posing difficulty in disclosing and explaining the result [24]. Molecularly targeted drugs including PARP inhibitors and immune checkpoint inhibitors may be linked to germline variants that are unknown to patients. Based on current evidence, medical oncologists must ensure germline carrier status determination.

Overall, the purpose of the Fact Sheet and its concepts were acceptable to both medical oncologists and clinical geneticists. The cheaper the CGP, the more likely it will be the first choice of cancer gene testing for cancer patients. Healthcare professionals unfamiliar with cancer predisposition genes may encounter GPV carriers at a certain frequency. In the evaluation survey, medical oncologists rated the NCCN guidelines higher than other resources with which they may not be familiar, such as GeneReviews and the ClinGen Actionability Summary Report. This indicates that genetic specialists, including genetic counselors, nurses, and medical geneticists play an important role in distributing the latest detailed information.

Some GPVs were missed by the CGP, suggesting that CGP is not an ideal genetic test for exploring GPV. Therefore, selecting genetic testing based on the patient's personal and family histories is crucial [58]. On the other hands, germline pathogenic variants are detected in patients who did not meet the screening or the criteria for a cancer predisposition gene [2, 59, 60]. Without a family or personal history related to the GPV/PGPV results, some patients may be unable to properly internalize their cancer risks. Therefore, introducing patients to genetic counseling is important before further processes. Genetic specialists provide psychosocial follow-up, including guidance on how to inform relatives about the CGP results.

## Conclusion

As CGP has become essential in cancer treatment, the detection of GPV/PGPV cases has increased, rendering “cancer predisposition gene explanations” routine for medical oncologists. The Fact Sheet developed in this study, under the supervision of Genetic Experts, provides key information on each gene, including associated cancers, surveillance information, and genetic counseling guidance. The evaluation survey targeting oncologists also suggests that the Fact Sheet may be helping them. It also serves as an educational tool that requires regular updates and aligns with the guideline revisions.

## Supplementary Information

Below is the link to the electronic supplementary material.

Supplementary file1 (DOCX 670 KB)

Supplementary file2 (DOCX 569 KB)

Supplementary file3 (DOCX 26 KB)

## Data Availability

The datasets analyzed in the current study are available from the corresponding author upon request.
