# Supplementary material for: Establishment of a comprehensive set of fact sheets for cancer predisposition genes for medical oncologists practicing cancer genome profiling
Source: Int J Clin Oncol. 2025 Apr 4;30(5):827–36. doi: 10.1007/s10147-025-02746-w (PMC12014809; doi:10.1007/s10147-025-02746-w)
Supplement: Supplementary file 1 — Supplementary file1 (DOCX 670 KB) [file 10147_2025_2746_MOESM1_ESM.docx]

***APC*遺伝子について**

*がんの発症と関連する遺伝子の変化について*

- - 一般的にがんの5~10%は「生まれつきの遺伝子の変化」が原因といわれています。
  - 「生まれつきの遺伝子の変化」をもっている場合、がんになりやすい体質をもっていると考えられます。
  - がんになりやすい体質は親、子、兄弟、姉妹 などの血のつながった家族と共有している可能性があります。
  - 体質を知ることで、その情報をご自身やご家族の健康管理に役立てることができます。
  - 今回の遺伝子検査でみつかった変化が「生まれつきの遺伝子の変化」かどうかは、血液検査により確認できます。

*APC*遺伝子とは？

- *APC遺伝子*の生まれつきの変化は***家族性大腸腺腫症***の原因となることが知られています。裏面の資料 (表) をご参照ください。

「生まれつきの遺伝子の変化」であるかどうかを知ることはどんなことに役立ちますか？

- 発症リスクが高いがんを知り、それに対する検診を行うことで、早期発見につながる可能性があります。


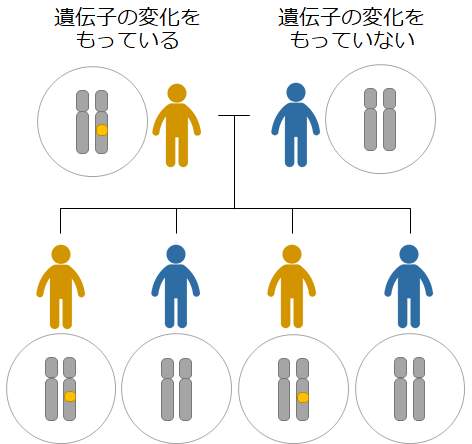
家族にどんな影響がありますか？

- 人は２つの*APC*遺伝子をそれぞれ両親から1つずつ受け継いでいます。
- *APC*遺伝子の変化を生まれつきもっていた場合、両親、兄弟、姉妹、子どもが同じ変化をもっている確率は50%です。同じ変化をもっているかは、遺伝子検査で確認することができます。
- おば、おじ、いとこなども一定の確率で*同じ遺伝子の変化をもっている*可能性があります。
- 遺伝子の変化についてご家族の方と情報共有することは、ご家族の方の健康管理に役立つ可能性があります。

※生まれつきの*APC*遺伝子の変化は、両親いずれかから受け継いだものではなく、新規に起こった変化である可能性が20~25%^1)^ (*注意：家族性大腸腺腫症として*) といわれています。この場合のご家族への影響については、遺伝専門外来におたずねください。

遺伝カウンセリングについて

遺伝カウンセリングでは、生まれつきの遺伝子の変化と体質との関連、ご家族への影響などについてより詳しくご説明します。お気軽にご相談ください。　注意：遺伝学的検査の保険適用には条件があり、関連する症状を発症していないご家族の遺伝子検査や検診は自費となります。詳しくは担当者におたずね下さい。

＜参考資料表＞

|  | 一般的な日本人 | *APC*遺伝子に病気の原因となる変化を生まれつきもっている | | | |
| --- | --- | --- | --- | --- | --- |
|  | 生涯を通して  罹患する確率^2,3)^ | 罹患する確率 | | 推奨される対応 | |
|  |  | 女性^1,4,5,6)^ | 男性^1,4,5,6)^ | 女性^1,4)^ | 男性^1,4)^ |
| 大腸ポリポーシス  (がん化するリスクあり) | (大腸がん)  10.2 ％ (男性)  8.0 ％ (女性) | （大腸ポリープ）  35歳までに95%  （大腸がん）  40~55歳までに50 % | | 予防的大腸切除術：  一般に20歳代だが、症状や社会的背景によって総合的に判断  大腸内視鏡検査：  1~3年ごとにポリープの状態に応じて | |
| 胃底腺ポリポーシス  ・胃腺腫  (がん化するリスクあり) | (胃がん)  10.3 ％ (男性)  4.7 ％ (女性) | 50歳までに  （胃腺腫）  21.8 %  （胃がん）  3.8％ | | 上部消化管内視鏡検査：  20~25歳以降、年１回 | |
| 十二指腸腺腫  (がん化するリスクあり) | (小腸がん)  0.2% (男性)  0.1% (女性) | (十二指腸がん)  4~12 % | |  |  |
| デスモイド腫瘍 | データなし | 10~15 % | | 年１回の腹部触診、CTあるいはMRI | |
| 甲状腺がん | 0.6 ％ (男性)  1.7 ％ (女性) | (甲状腺乳頭がん、主に女性)  1~12% | | 年１回の甲状腺の触診と  超音波検査  (主に女性が対象) | |
| 脳腫瘍 | 0.3 ％ (男性)  0.2 ％ (女性) | リスクが高まる可能性 | | 年１回の診察 | |

- 上表以外にも、肝芽腫、副腎腫瘍などがみられることがあります。詳細は専門診療科あるいは遺伝専門外来におたずねください。

※*APC遺伝子*の変化が生まれつきの変化であっても、必ずがんを発症するというわけではありません。

※遺伝子の変化をもっていた場合の罹患する確率、推奨される対応の記載は日本の診療ガイドライン^1)^および米国のガイドライン (NCCNガイドライン)^4)^を参照しています。推奨事項については日本で必ずしも実施されているとは限りませんので、詳細は各施設の専門診療科あるいは遺伝専門外来におたずねください。

※表は2021年時点の記載です。今後、研究が進み推奨内容が変わる可能性があります。

【参考文献】

- 大腸癌研究会. 遺伝性大腸癌診療ガイドライン 2020年版. (2020年4月) [ref. 1]
- 国立がん研究センターがん情報サービス「累積がん罹患リスク(2018年データ)」<https://ganjoho.jp/reg_stat/statistics/stat/summary.html> [ref. 2]
- 厚生労働省健康局がん・疾病対策課. 平成30年全国がん登録 罹患数・率報告 2018 [ref. 3]
- NCCN Guidelines® Genetic/Familial High-Risk Assessment: Colorectal. ver.1.2021 (2021.5.11) [ref. 4]
- GeneReviews Japan: *APC関連ポリポーシス*. ver.2018.5.6 [ref. 5]
- Upper gastrointestinal tumours in Japanese familial adenomatous polyposis patients. Jpn J Clin Oncol, 2016; 46(4): 310. [ref. 6]
- ClinGen Actionability Reports: APC Adult. ver.3.0.4 (2021.5.6)

監修：厚労科研小杉班・Actionability Working Group-Japan

編集： MONSTAR-SCREEN-2遺伝事務局

***ATM*遺伝子について**

*がんの発症と関連する遺伝子の変化について*

- - 一般的にがんの5~10%は「生まれつきの遺伝子の変化」が原因といわれています。
  - 「生まれつきの遺伝子の変化」をもっている場合、がんになりやすい体質をもっていると考えられます。
  - がんになりやすい体質は親、子、兄弟、姉妹 などの血のつながった家族と共有している可能性があります。
  - 体質を知ることで、その情報をご自身やご家族の健康管理に役立てることができます。
  - 今回の遺伝子検査でみつかった変化が「生まれつきの遺伝子の変化」かどうかは、血液検査により確認できます。

*ATM*遺伝子とは？

- *がんを発症するリスクと関連することが示されている遺伝子です。裏面の資料* (*表*) *をご参照ください*。

「生まれつきの遺伝子の変化」であるかどうかを知ることはどんなことに役立ちますか？

- 発症リスクが高いがんを知り、それに対する検診を行うことで、早期発見につながる可能性があります。


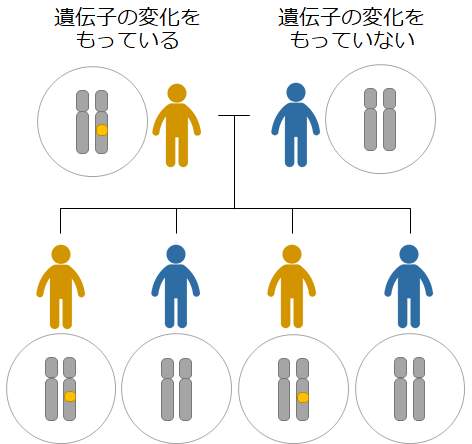
家族にどんな影響がありますか？

- 人は２つの*ATM*遺伝子をそれぞれ両親から1つずつ受け継いでいます。
- *ATM*遺伝子の変化を生まれつきもっていた場合、両親、兄弟、姉妹、子どもが同じ変化をもっている確率は50%です。同じ変化をもっているかは、遺伝子検査で確認することができます。
- おば、おじ、いとこなども一定の確率で*同じ遺伝子の変化をもっている*可能性があります。
- 遺伝子の変化についてご家族の方と情報共有することは、ご家族の方の健康管理に役立つ可能性があります。

※生まれつきの*ATM*遺伝子の変化は、両親いずれかから受け継いだものではなく、新規に起こった変化である可能性もあります。この場合のご家族への影響については、遺伝専門外来におたずねください。

遺伝カウンセリングについて

遺伝カウンセリングでは、生まれつきの遺伝子の変化と体質との関連、ご家族への影響などについてより詳しくご説明します。お気軽にご相談ください。 注意：遺伝学的検査の保険適用には条件があり、関連する症状を発症していないご家族の遺伝子検査や検診は自費となります。詳しくは担当者におたずね下さい。

＜参考資料表＞

|  | 一般的な日本人 | *ATM遺伝子*に病気の原因となる変化を生まれつきもっている | | | |
| --- | --- | --- | --- | --- | --- |
|  | 生涯を通して  罹患する確率^1)^ | 生涯を通して  罹患する確率 | | 推奨される対応 | |
|  |  | 女性^2)^ | 男性^2)^ | 女性^2)^ | 男性^2)^ |
| 乳がん | 10.9 ％ (女性) | 15~40 ％ | ― | 40歳以降:年1回のマンモグラフィと乳房造影MRI検査。 (注) | ― |
| 膵がん | 2.6 % (女性)  2.6 % (男性) | ≦10 % | | ※現時点では検診方法は確立されていません。 | |
| 卵巣がん | 1.6 % (女性) | < 3 % | ― | ※現時点では検診方法は確立されていません。家族歴や既往歴によってリスクは異なると考えられています。 | ― |
| 前立腺がん | 10.8 ％ (男性) | ― | データ  不十分 | ― | ― |

(注) 記載の推奨事項については、本邦での具体的な検診方法は示されていません。詳細は各施設の遺伝専門外来におたずねください。

※*ATM遺伝子*の変化が生まれつきの変化であっても、必ずがんを発症するというわけではありません。

※遺伝子の変化をもっていた場合の罹患する確率、推奨される対応の記載は米国のガイドライン (NCCNガイドライン)^2)^ を参照しています。

※表は2021年時点の記載です。今後、研究が進み推奨内容が変わる可能性があります。

【参考文献】

- 国立がん研究センターがん情報サービス「累積がん罹患リスク (2018年データ)」https://ganjoho.jp/reg_stat/statistics/stat/summary.html [ref. 1]
- NCCN Guidelines® Genetic/Familial High-Risk Assessment: Breast, Ovarian, and Pancreatic. ver.1.2022 (2021.8.11) [ref. 2]
- ClinGen Actionability Reports: ATM, CHEK2 Adult. ver.1.1.1 (2020.4.16)

監修：厚労科研小杉班・Actionability Working Group-Japan

編集： MONSTAR-SCREEN-2遺伝事務局

***ATM*遺伝子について**

*がんの発症と関連する遺伝子の変化について*

- - 一般的にがんの5~10%は「生まれつきの遺伝子の変化」が原因といわれています。
  - 「生まれつきの遺伝子の変化」をもっている場合、がんになりやすい体質をもっていると考えられます。
  - がんになりやすい体質は親、子、兄弟、姉妹 などの血のつながった家族と共有している可能性があります。
  - 体質を知ることで、その情報をご自身やご家族の健康管理に役立てることができます。
  - 今回の遺伝子検査でみつかった変化が「生まれつきの遺伝子の変化」かどうかは、血液検査により確認できます。

*ATM*遺伝子とは？

- *がんを発症するリスクと関連することが示されている遺伝子です。裏面の資料* (*表*) *をご参照ください*。

「生まれつきの遺伝子の変化」であるかどうかを知ることはどんなことに役立ちますか？

- 発症リスクが高いがんを知り、それに対する検診を行うことで、早期発見につながる可能性があります。


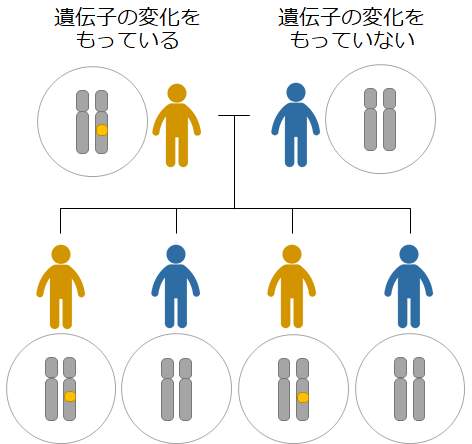
家族にどんな影響がありますか？

- 人は２つの*ATM*遺伝子をそれぞれ両親から1つずつ受け継いでいます。
- *ATM*遺伝子の変化を生まれつきもっていた場合、両親、兄弟、姉妹、子どもが同じ変化をもっている確率は50%です。同じ変化をもっているかは、遺伝子検査で確認することができます。
- おば、おじ、いとこなども一定の確率で*同じ遺伝子の変化をもっている*可能性があります。
- 遺伝子の変化についてご家族の方と情報共有することは、ご家族の方の健康管理に役立つ可能性があります。

※生まれつきの*ATM*遺伝子の変化は、両親いずれかから受け継いだものではなく、新規に起こった変化である可能性もあります。この場合のご家族への影響については、遺伝専門外来におたずねください。

遺伝カウンセリングについて

遺伝カウンセリングでは、生まれつきの遺伝子の変化と体質との関連、ご家族への影響などについてより詳しくご説明します。お気軽にご相談ください。 注意：遺伝学的検査の保険適用には条件があり、関連する症状を発症していないご家族の遺伝子検査や検診は自費となります。詳しくは担当者におたずね下さい。

＜参考資料表＞

|  | 一般的な日本人 | *ATM遺伝子*に病気の原因となる変化を生まれつきもっている | | | |
| --- | --- | --- | --- | --- | --- |
|  | 生涯を通して  罹患する確率^1)^ | 生涯を通して  罹患する確率 | | 推奨される対応 | |
|  |  | 女性^2)^ | 男性^2)^ | 女性^2)^ | 男性^2)^ |
| 乳がん | 10.9 ％ (女性) | 15~40 ％ | ― | 40歳以降:年1回のマンモグラフィと乳房造影MRI検査。 (注) | ― |
| 膵がん | 2.6 % (女性)  2.6 % (男性) | ≦10 % | | ※現時点では検診方法は確立されていません。 | |
| 卵巣がん | 1.6 % (女性) | < 3 % | ― | ※現時点では検診方法は確立されていません。家族歴や既往歴によってリスクは異なると考えられています。 | ― |
| 前立腺がん | 10.8 ％ (男性) | ― | データ  不十分 | ― | ― |

(注) 記載の推奨事項については、本邦での具体的な検診方法は示されていません。詳細は各施設の遺伝専門外来におたずねください。

※*ATM遺伝子*の変化が生まれつきの変化であっても、必ずがんを発症するというわけではありません。

※遺伝子の変化をもっていた場合の罹患する確率、推奨される対応の記載は米国のガイドライン (NCCNガイドライン)^2)^ を参照しています。

※表は2021年時点の記載です。今後、研究が進み推奨内容が変わる可能性があります。

【参考文献】

- 国立がん研究センターがん情報サービス「累積がん罹患リスク (2018年データ)」https://ganjoho.jp/reg_stat/statistics/stat/summary.html [ref. 1]
- NCCN Guidelines® Genetic/Familial High-Risk Assessment: Breast, Ovarian, and Pancreatic. ver.1.2022 (2021.8.11) [ref. 2]
- ClinGen Actionability Reports: ATM, CHEK2 Adult. ver.1.1.1 (2020.4.16)

監修：厚労科研小杉班・Actionability Working Group-Japan

編集： MONSTAR-SCREEN-2遺伝事務局

***BAP1*遺伝子について**

*がんの発症と関連する遺伝子の変化について*

- - 一般的にがんの5~10%は「生まれつきの遺伝子の変化」が原因といわれています。
  - 「生まれつきの遺伝子の変化」をもっている場合、がんになりやすい体質をもっていると考えられます。
  - がんになりやすい体質は親、子、兄弟、姉妹 などの血のつながった家族と共有している可能性があります。
  - 体質を知ることで、その情報をご自身やご家族の健康管理に役立てることができます。
  - 今回の遺伝子検査でみつかった変化が「生まれつきの遺伝子の変化」かどうかは、血液検査により確認できます。

*BAP1*遺伝子とは？

- *BAP1遺伝子*の生まれつきの変化は***BAP1*腫瘍素因症候群**の原因となることが知られています。裏面の資料 (表) をご参照ください。

「生まれつきの遺伝子の変化」であるかどうかを知ることはどんなことに役立ちますか？

- 発症リスクが高いがんを知り、それに対する検診を行うことで、早期発見につながる可能性があります。

家族にどんな影響がありますか？
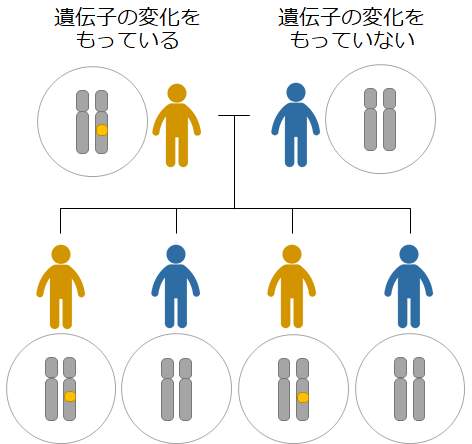


- 人は２つの*BAP1*遺伝子をそれぞれ両親から1つずつ受け継いでいます。
- *BAP1*遺伝子の変化を生まれつきもっていた場合、両親、兄弟、姉妹、子どもが同じ変化をもっている確率は50%です。同じ変化をもっているかは、遺伝子検査で確認することができます。
- おば、おじ、いとこなども一定の確率で*同じ遺伝子の変化をもっている*可能性があります。
- 遺伝子の変化についてご家族の方と情報共有することは、ご家族の方の健康管理に役立つ可能性があります。

※生まれつきの*BAP1*遺伝子の変化は、両親いずれかから受け継いだものではなく、新規に起こった変化である可能性もあります。この場合のご家族への影響については、遺伝専門外来におたずねください。

遺伝カウンセリングについて

遺伝カウンセリングでは、生まれつきの遺伝子の変化と体質との関連、ご家族への影響などについてより詳しくご説明します。お気軽にご相談ください。　注意：遺伝学的検査の保険適用には条件があり、関連する症状を発症していないご家族の遺伝子検査や検診は自費となります。詳しくは担当者におたずね下さい。

＜参考資料表＞

|  | 一般的な日本人 | | *BAP1*遺伝子に病気の原因となる変化を生まれつきもっている | |
| --- | --- | --- | --- | --- |
|  | 生涯を通して  罹患する確率^1,2)^ | | 生涯を通して  罹患する確率^3)^ | 推奨される対応^3,4,5)^ |
|  | 女性 | 男性 |  |  |
| 非典型Spitz腫瘍 | 不明 | | 76 % | 皮膚科の精密検査 (年1回)：20歳以降 (注2) |
| 皮膚悪性黒色腫 (注1) | 0.1 % | 0.1 % | 13 % |  |
| 基底細胞がん | (皮膚がん) | | 6.3 ％ |  |
|  | 0.5 % | 0.6 % |  |  |
| ぶどう膜悪性黒色腫 | ※発生頻度：  年間50例程度 | | 31 % | 眼科の精密検査 (年1回)：11歳以降  (注2) |
| 悪性中皮腫 | (中皮腫) | | 39 % | 現時点では推奨される検診方法は確立されていません。 |
|  | 0.0 % | 0.1 % |  |  |
| 腎細胞がん | (腎 (腎盂を除く)) | | 10 % | 腹部MRI (推奨) またはCT (2年に1回)：30歳以降 (注2) |
|  | 0.4 % | 1.2 % |  |  |

(注1) 悪性黒色腫の罹病率 (年) は、白人が 24.3 人/10 万人、アジア人が 1.7 人/10 万人との報告があり^6)^、人種、地域、その他の遺伝的要因によってリスクは異なると考えられています。

(注2) 記載の推奨事項については、本邦での具体的な検診方法は示されていません。詳細は各施設の遺伝専門外来におたずねください。

※*BAP1遺伝子*の変化が生まれつきの変化であっても、必ずがんを発症するというわけではありません。

※遺伝子の変化をもっていた場合の罹患する確率、推奨される対応は海外の資料^3,4,5)^ を参照しています。

※表は2021年時点の記載です。今後、研究が進み推奨内容が変わる可能性があります。

【参考文献】

- 厚生労働健康局がん・疾病対策課. 平成30年全国がん登録 罹患数・率報告 2018 [ref. 1]
- 国立がん研究センター希少がんセンター<https://www.ncc.go.jp/jp/rcc/index.html> [ref. 2]
- ClinGen Actionability Reports: BAP1 Adult. ver.1.2.1 (2020.4.22) [ref. 3]
- NCCN Guidelines®: Kidney Cancer. ver.4.2022 (2021.12.21) [ref. 4]
- GeneReviews Japan: *BAP1*腫瘍素因症候群. ver.2019.3.22 [ref. 5]

- 日本皮膚科学会. 皮膚悪性腫瘍ガイドライン第 3 版 メラノーマ診療ガイドライン 2019. 日皮会誌, 2019; 129(9): 1759. [ref. 6]

監修：厚労科研小杉班・Actionability Working Group-Japan

編集： MONSTAR-SCREEN-2遺伝事務局

***BARD1*遺伝子について**

*がんの発症と関連する遺伝子の変化について*

- - 一般的にがんの5~10%は「生まれつきの遺伝子の変化」が原因といわれています。
  - 「生まれつきの遺伝子の変化」をもっている場合、がんになりやすい体質をもっていると考えられます。
  - がんになりやすい体質は親、子、兄弟、姉妹 などの血のつながった家族と共有している可能性があります。
  - 体質を知ることで、その情報をご自身やご家族の健康管理に役立てることができます。
  - 今回の遺伝子検査でみつかった変化が「生まれつきの遺伝子の変化」かどうかは、血液検査により確認できます。

*BARD1*遺伝子とは？

- がんを発症するリスクと関連があることが示されている遺伝子です。裏面の資料 (表) をご参照ください。

「生まれつきの遺伝子変化」であるかどうかを知ることはどんなことに役立ちますか？

- 発症リスクが高いがんを知り、それに対する検診を行うことで、早期発見につながる可能性があります。

家族にどんな影響がありますか？
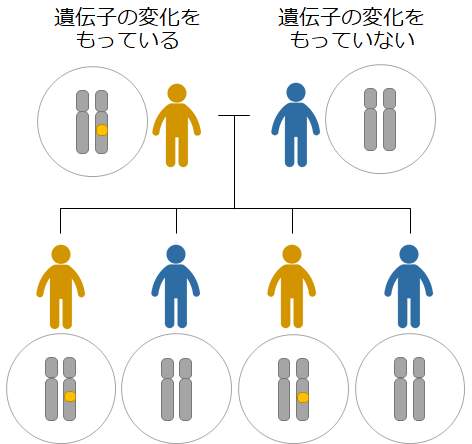


- 人は２つの*BARD1*遺伝子をそれぞれ両親から1つずつ受け継いでいます。
- *BARD1*遺伝子の変化を生まれつきもっていた場合、両親、兄弟、姉妹、子どもが同じ変化をもっている確率は50%です。同じ変化をもっているかは、遺伝子検査で確認することができます。
- おば、おじ、いとこなども一定の確率で*同じ遺伝子の変化をもっている*可能性があります。
- 遺伝子の変化についてご家族の方と情報共有することは、ご家族の方の健康管理に役立つ可能性があります。

※生まれつきの*BARD1*遺伝子の変化は、両親いずれかから受け継いだものではなく、新規に起こった変化である可能性もあります。この場合のご家族への影響については、遺伝専門外来におたずねください。

遺伝カウンセリングについて

遺伝カウンセリングでは、生まれつきの遺伝子の変化と体質との関連、ご家族への影響などについてより詳しくご説明します。お気軽にご相談ください。 注意：遺伝学的検査の保険適用には条件があり、関連する症状を発症していないご家族の遺伝子検査や検診は自費となります。詳しくは担当者におたずね下さい。

＜参考資料表＞

|  | 一般的な日本人 | *BARD1遺伝子*に病気の原因となる変化を生まれつきもっている | | | | |
| --- | --- | --- | --- | --- | --- | --- |
|  | 生涯を通して  罹患する確率^1)^ | | 生涯を通して  罹患する確率 | | 推奨される対応 | |
|  |  |  | 女性^2)^ | 男性 | 女性^2)^ | 男性 |
| 乳がん | 10.9 ％（女性） | | 15~40 ％ | ― | 40歳以降:年1回のマンモグラフィと乳房造影MRI検査（注） | ― |

(注) 記載の推奨事項については、本邦での具体的な検診方法は示されていません。詳細は各施設の遺伝専門外来におたずねください。

※*BARD1遺伝子*の変化が生まれつきの変化であっても、必ずがんを発症するというわけではありません。

※遺伝子の変化をもっていた場合の罹患する確率、推奨される対応の記載は米国のガイドライン (NCCNガイドライン)^2)^ を参照しています。

※表は2021年時点の記載です。今後、研究が進み推奨内容が変わる可能性があります。

【参考文献】

- 国立がん研究センターがん情報サービス「累積がん罹患リスク (2018年データ)」 https://ganjoho.jp/reg_stat/statistics/stat/summary.html [ref. 1]
- NCCN Guidelines® Genetic/Familial High-Risk Assessment: Breast, Ovarian, and Pancreatic. ver.1.2022 (2021.8.11) [ref. 2]

監修：厚労科研小杉班・Actionability Working Group-Japan

編集： MONSTAR-SCREEN-2遺伝事務局

***BMPR1A*遺伝子について**

*がんの発症と関連する遺伝子の変化について*

- - 一般的にがんの5~10%は「生まれつきの遺伝子の変化」が原因といわれています。
  - 「生まれつきの遺伝子の変化」をもっている場合、がんになりやすい体質をもっていると考えられます。
  - がんになりやすい体質は親、子、兄弟、姉妹 などの血のつながった家族と共有している可能性があります。
  - 体質を知ることで、その情報をご自身やご家族の健康管理に役立てることができます。
  - 今回の遺伝子検査でみつかった変化が「生まれつきの遺伝子の変化」かどうかは、血液検査により確認できます。

*BMPR1A*遺伝子とは？

- *BMPR1A遺伝子*の生まれつきの変化は**若年性ポリポーシス症候群**の原因となることが知られています。裏面の資料 (表) をご参照ください。

「生まれつきの遺伝子の変化」であるかどうかを知ることはどんなことに役立ちますか？

- 発症リスクが高いがんを知り、それに対する検診を行うことで、早期発見につながる可能性があります。

家族にどんな影響がありますか？
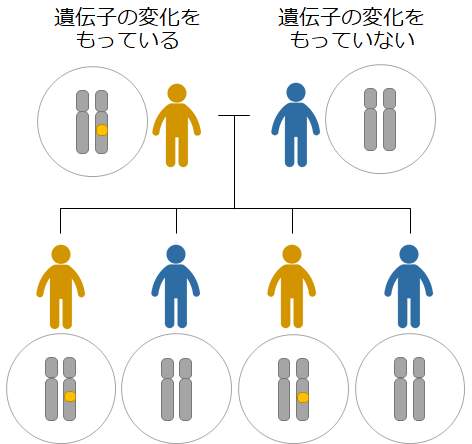


- 人は２つの*BMPR1A*遺伝子をそれぞれ両親から1つずつ受け継いでいます。
- *BMPR1A*遺伝子の変化を生まれつきもっていた場合、両親、兄弟、姉妹、子どもが同じ変化をもっている確率は50%です。同じ変化をもっているかは、遺伝子検査で確認することができます。
- おば、おじ、いとこなども一定の確率で*同じ遺伝子の変化をもっている*可能性があります。
- 遺伝子の変化についてご家族の方と情報共有することは、ご家族の方の健康管理に役立つ可能性があります。

※生まれつきの*BMPR1A*遺伝子の変化は、両親いずれかから受け継いだものではなく、新規に起こった変化である可能性もあります。この場合のご家族への影響については、遺伝専門外来におたずねください。

遺伝カウンセリングについて

遺伝カウンセリングでは、生まれつきの遺伝子の変化と体質との関連、ご家族への影響などについてより詳しくご説明します。お気軽にご相談ください。　注意：遺伝学的検査の保険適用には条件があり、関連する症状を発症していないご家族の遺伝子検査や検診は自費となります。詳しくは担当者におたずね下さい。

＜参考資料表＞

|  | 一般的な日本人 | *BMPR1A*遺伝子に病気の原因となる変化を生まれつきもっている | | | |
| --- | --- | --- | --- | --- | --- |
|  | 生涯を通して  罹患する確率^1,2)^ | 生涯を通して  罹患する確率 | | 推奨される対応 | |
|  |  | 女性^3,4)^ | 男性^3,4)^ | 女性^3,5)^ | 男性^3,5)^ |
| 大腸ポリープ(がん化するリスクあり) | (大腸がん)  10.2 ％ (男性)  8.0 ％ (女性) | （なんらかのポリープ）  ≦90 %  (大腸がん)  ≦68 %  (胃・小腸がん)  ≦21 %  （注） | | 上部・下部消化管内視鏡検査：  1~3 年おきに検査を行う | |
| 胃ポリープ  (がん化するリスクあり) | (胃がん)  10.3 ％ (男性)  4.7 ％ (女性) |  |  |  |  |
| 小腸ポリープ | (小腸がん)  0.2 % (男性)  0.1 % (女性) |  |  |  |  |

（注）若年性ポリポーシスとしての値となります。

※*BMPR1A遺伝子*の変化が生まれつきの変化であっても、必ずがんを発症するというわけではありません。ほとんどの若年性ポリポーシス症候群の患者さんは20歳までに何個かのポリープを認めることがわかっています。詳細は専門診療科あるいは遺伝専門外来にておたずねください。

※遺伝子の変化をもっていた場合の罹患する確率、推奨される対応の記載は日本の診療ガイドライン^3)^ および米国のガイドライン (NCCNガイドライン)^5)^を参照しています。推奨事項については、各医療機関において必ずしも実施されているとは限りませんので、詳細は各施設の専門診療科あるいは遺伝専門外来におたずねください。

※表は2021年時点の記載です。今後、研究が進み推奨内容が変わる可能性があります。

【参考文献】

- 国立がん研究センターがん情報サービス「累積がん罹患リスク (2018年データ)」<https://ganjoho.jp/reg_stat/statistics/stat/summary.html> [ref. 1]
- 厚生労働省健康局がん・疾病対策課 平成30年全国がん登録 罹患数・率報告 2018 [ref. 2]
- 小児・成人のための若年性ポリポーシス 症候群診療ガイドライン 2020年版. 遺伝性腫瘍, 2020; 20(2): 79. [ref. 3]
- ClinGen Actionability Reports: SMAD4, BMPR1A Adult. ver.1.1.1 (2021.1.19) [ref. 4]
- NCCN Guidelines® Genetic/Familial High-Risk Assessment: Colorectal. ver.1.2021 (2021.5.11) [ref. 5]
- GeneReviews Japan: 若年性ポリポーシス症候群. ver.2014.3.3.

監修：厚労科研小杉班・Actionability Working Group-Japan

編集： MONSTAR-SCREEN-2遺伝事務局

***BRCA1*遺伝子について**

*がんの発症と関連する遺伝子の変化について*

- 一般的にがんの5~10%は「生まれつきの遺伝子の変化」が原因といわれています。
  - 「生まれつきの遺伝子の変化」をもっている場合、がんになりやすい体質をもっていると考えられます。
  - がんになりやすい体質は親、子、兄弟、姉妹などの血のつながった家族と共有している可能性があります。
  - 体質を知ることで、その情報をご自身やご家族の健康管理に役立てることができます。
  - 今回の遺伝子検査でみつかった変化が「生まれつきの遺伝子の変化」かどうかは、血液検査により確認できます。

*BRCA1*遺伝子とは？

- *BRCA1遺伝子*の生まれつきの変化は**遺伝性乳癌卵巣癌**の原因となることが知られています。*裏面の資料* (*表*) をご参照ください。

「生まれつきの遺伝子変化」であるかどうかを知ることはどんなことに役立ちますか？

- 発症リスクが高いがんを知り、それに対する検診を行うことで、早期発見につながる可能性があります。

家族にどんな影響がありますか？
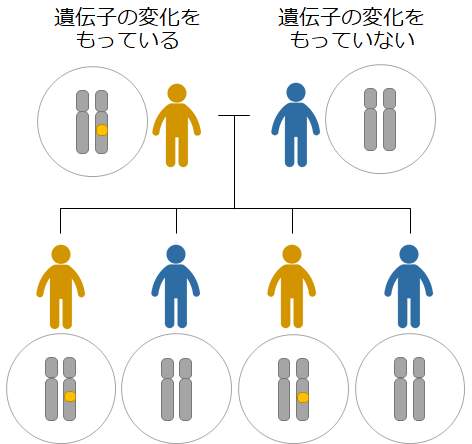


- 人は２つの*BRCA1*遺伝子をそれぞれ両親から1つずつ受け継いでいます。
- *BRCA1*遺伝子の変化を生まれつきもっていた場合、両親、兄弟、姉妹、子どもが同じ変化をもっている確率は50%です。同じ変化をもっているかは、遺伝子検査で確認することができます。
- おば、おじ、いとこなども一定の確率で*同じ遺伝子の変化をもっている*可能性があります。
- 遺伝子の変化についてご家族の方と情報共有することは、ご家族の方の健康管理に役立つ可能性があります。

※生まれつきの*BRCA1*遺伝子の変化は、両親いずれかから受け継いだものではなく、新規に起こった変化である可能性が0.1%^1)^といわれています。この場合のご家族への影響については、遺伝専門外来におたずねください。

遺伝カウンセリングについて

遺伝カウンセリングでは、生まれつきの遺伝子の変化と体質との関連、ご家族への影響などについてより詳しくご説明します。お気軽にご相談ください。 注意：遺伝学的検査の保険適用には条件があり、関連する症状を発症していないご家族の遺伝子検査や検診は自費となります。詳しくは担当者におたずね下さい。

＜参考資料表＞

|  | 一般的な日本人 | *BRCA1遺伝子*に病気の原因となる変化を生まれつきもっている | | | | |
| --- | --- | --- | --- | --- | --- | --- |
|  | 生涯で  罹患する  確率^2,3)^ | 80歳までに  罹患する確率 | | | 推奨される対応 | |
|  |  | 女性^3,4,5)^ | 男性^3,6)^ | 女性^3,4,5,8)^ | | 男性^3,6,8)^ |
| 乳がん | 10.9 ％  (女性)  0.1 ％  (男性) | 57~72 ％ | 1.2 ％ | 18歳以降:自己乳房検診  25歳以降:半年～１年ごとに医師による視触診  25~29歳:年1回の乳房造影MRI検査  30~75歳:年1回のマンモグラフィーと乳房MRI検査  リスク低減乳房切除術 (RRM) を検討 | | 35歳以降:  自己乳房検診、１年ごとに医師による診察  (女性化乳房が認められる男性) 50歳以降:  年1回のマンモグラフィー |
| 卵巣がん | 1.6 %  (女性) | 40~44 % | ― | 35~40歳:リスク低減卵管卵巣摘出術(RRSO) を推奨  30~35歳以降:経腟超音波検査、CA125検査を考慮 | | ― |
| 前立腺  がん | 10.8 ％  (男性) | ― | 65歳までに　8.6 ％(注) | ― | | 40歳以降:前立腺がん検診 (PSA) を考慮 |
| 膵がん | 2.6 %  (男女) | ≦5% | | MRIまたは超音波内視鏡を用いたスクリーニングを考慮。 | | |

(注) 一般に比べ、罹患リスクが1.8～3.8倍上昇するとされています (65歳未満)^6,7)^。

※*BRCA1遺伝子*の変化が生まれつきの変化であっても、必ずがんを発症するというわけではありません。

※遺伝子の変化をもっていた場合の罹患する率、推奨される対応の記載は日本の診療ガイドライン^3)^および米国のガイドライン (NCCNガイドライン)^8)^ を参照しています。推奨事項については、日本で必ずしも実施されているとは限りませんので、詳細は各施設の遺伝専門外来におたずねください。

※表は2021年時点の記載です。今後、研究が進み推奨内容が変わる可能性があります。

【参考文献】

- Breast and ovarian cancer predisposition due to de novo BRCA1 and BRCA2 mutations. Oncogene, 2016; 35(10): 1324. [ref. 1]
- 国立がん研究センターがん情報サービス「累積がん罹患リスク (2018年データ)」 <https://ganjoho.jp/reg_stat/statistics/stat/summary.html> [ref. 2]
- 日本遺伝性乳癌卵巣癌総合診療精度機構. 遺伝性乳癌卵巣癌 (HBOC) 診療ガイドライン2021年版. (2021年7月) [ref. 3]
- Meta-analysis of BRCA2 and BRCA2 penetrance. J Clin Oncol, 2007; 25(11): 1329. [ref. 4]
- Risks of Breast, Ovarian, and Contralateral Breast Cancer for BRCA2 and BRCA2 Mutation Carriers. JAMA, 2017; 317(23): 2402. [ref. 5]
- Germline BRCA1 mutations increase prostate cancer risk. Br J Cancer, 2012; 106(10): 1697. [ref. 6]
- Cancer incidence in BRCA1 mutation carriers. J Natl Cancer Inst, 2002; 94(18): 1358. [ref. 7]
- NCCN Guidelines® Genetic/Familial High-Risk Assessment: Breast, Ovarian, and Pancreatic. ver.1.2022 (2021.8.11) [ref. 8]
- GeneReviews Japan: *BRCA1*および*BRCA2*関連遺伝性乳癌卵巣癌. ver.2016.12.25 (minor revision; 2017.2.20)
- ClinGen Actionability Reports: BRCA1, BRCA2 Adult. ver.1.1.3 (2021.9.15)

監修：厚労科研小杉班・Actionability Working Group-Japan

編集： MONSTAR-SCREEN-2遺伝事務局

***BRCA2*遺伝子について**

*がんの発症と関連する遺伝子の変化について*

- 一般的にがんの5~10%は「生まれつきの遺伝子の変化」が原因といわれています。
  - 「生まれつきの遺伝子の変化」をもっている場合、がんになりやすい体質をもっていると考えられます。
  - がんになりやすい体質は親、子、兄弟、姉妹などの血のつながった家族と共有している可能性があります。
  - 体質を知ることで、その情報をご自身やご家族の健康管理に役立てることができます。
  - 今回の遺伝子検査でみつかった変化が「生まれつきの遺伝子の変化」かどうかは、血液検査により確認できます。

*BRCA2*遺伝子とは？

- *BRCA2遺伝子*の生まれつきの変化は**遺伝性乳癌卵巣癌**の原因となることが知られています。裏面の資料 (表) をご参照ください。

「生まれつきの遺伝子変化」であるかどうかを知ることはどんなことに役立ちますか？

- 発症リスクが高いがんを知り、それに対する検診を行うことで、早期発見につながる可能性があります。


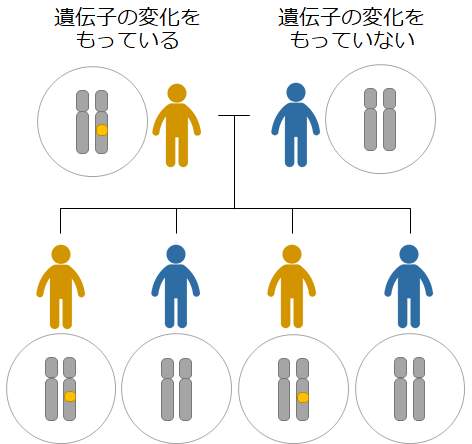
家族にどんな影響がありますか？

- 人は２つの*BRCA2*遺伝子をそれぞれ両親から1つずつ受け継いでいます。
- *BRCA2*遺伝子の変化を生まれつきもっていた場合、両親、兄弟、姉妹、子どもが同じ変化をもっている確率は50%です。同じ変化をもっているかは、遺伝子検査で確認することができます。
- おば、おじ、いとこなども一定の確率で*同じ遺伝子の変化をもっている*可能性があります。
- 遺伝子の変化についてご家族の方と情報共有することは、ご家族の方の健康管理に役立つ可能性があります。

※生まれつきの*BRCA2*遺伝子の変化は、両親いずれかから受け継いだものではなく、新規に起こった変化である可能性が0.7 %^1)^といわれています。この場合のご家族への影響については、遺伝専門外来におたずねください。

遺伝カウンセリングについて

遺伝カウンセリングでは、生まれつきの遺伝子の変化と体質との関連、ご家族への影響などについてより詳しくご説明します。お気軽にご相談ください。 注意：遺伝学的検査の保険適用には条件があり、関連する症状を発症していないご家族の遺伝子検査や検診は自費となります。詳しくは担当者におたずね下さい。

＜参考資料表＞

|  | 一般的な日本人 | *BRCA2遺伝子*に病気の原因となる変化を生まれつきもっている | | | |
| --- | --- | --- | --- | --- | --- |
|  | 生涯で  罹患する確率^2,3)^ | 80歳までに  罹患する確率 | | 推奨される対応 | |
|  |  | 女性^3,4,5)^ | 男性^3,6,7,8,9)^ | 女性^3,10)^ | 男性^3,10)^ |
| 乳がん | 10.9 ％  (女性)  0.1 ％  (男性) | 49~69 ％ | 7~8 ％ | 18歳以降:自己乳房検診  25歳以降:半年～１年ごとに医師による視触診  25~29歳:年1回の乳房造影MRI検査  30~75歳:年1回のマンモグラフィーと乳房MRI検査  リスク低減乳房切除術 (RRM) を検討 | 35歳以降:自己乳房検診、１年ごとに医師による診察  (女性化乳房が認められる男性) 50歳以降:年1回のマンモグラフィー |
| 卵巣  がん | 1.6%  (女性) | 17~18 % | ― | 35~40歳:リスク低減卵管卵巣摘出術 (RRSO) を検討  30~35歳以降:経腟超音波検査、CA125検査を考慮 | ― |
| 前立腺がん | 10.8 ％  (男性) | ― | 7.5~15 ％  (注) | ― | 40歳以降:前立腺がん検診 (PSA) を推奨 |
| 膵がん | 2.6 %  (男女) | 5~10 % | | MRIまたは超音波内視鏡を用いたスクリーニングを考慮。 | |

(注) 一般に比べ、罹患リスクが7.3～8.6倍上昇するとされています (65~70歳未満)^8,9)^。

※*BRCA2遺伝子*の変化が生まれつきの変化であっても、必ずがんを発症するというわけではありません。

※遺伝子の変化をもっていた場合の罹患する確率、推奨される対応の記載は日本の診療ガイドライン^3)^および米国のガイドライン (NCCNガイドライン)^9)^ を参照しています。推奨事項については、日本で必ずしも実施されているとは限りませんので、詳細は各施設の遺伝専門外来におたずねください。

※表は2021年時点の記載です。今後、研究が進み推奨内容が変わる可能性があります。

【参考文献】

- Breast and ovarian cancer predisposition due to de novo BRCA1 and BRCA2 mutations. Oncogene, 2016; 35(10): 1324. [ref. 1]
- 国立がん研究センターがん情報サービス「累積がん罹患リスク (2018年データ)」https://ganjoho.jp/reg_stat/statistics/stat/summary.html [ref. 2]
- 日本遺伝性乳癌卵巣癌総合診療精度機構. 遺伝性乳癌卵巣癌 (HBOC) 診療ガイドライン2021年版. (2021年7月) [ref. 3]
- Meta-analysis of BRCA2 and BRCA2 penetrance. J Clin Oncol, 2007; 25(11): 1329. [ref. 4]
- Risks of Breast, Ovarian, and Contralateral Breast Cancer for BRCA2 and BRCA2 Mutation Carriers. JAMA, 2017; 317(23): 2402. [ref. 5]
- Breast cancer risk among male BRCA1 and BRCA2 mutation carriers. J Natl Cancer Ins, 2007; 99(23): 1811. [ref. 6]
- Risk of breast cancer in male BRCA2 carriers. J Med Genet, 2010; 47(10): 710. [ref. 7]
- Germline BRCA mutations are associated with higher risk of nodal 11 involvement, distant metastasis, and poor survival outcomes in prostate cancer. J Clin Oncol, 2013; 31(14): 1748. [ref. 8]
- Breast Cancer Linkage Consortium Cancer risks in BRCA2 mutation carriers. J Natl Cancer Inst, 1999; 91(15): 1310. [ref. 9]
- NCCN Guidelines® Genetic/Familial High-Risk Assessment: Breast, Ovarian, and Pancreatic. ver.1.2022 (2021.8.11) [ref. 10]
- GeneReviews Japan: *BRCA1*および*BRCA2*関連遺伝性乳癌卵巣癌. ver.2016.12.25 (minor revision; 2017.2.20)
- ClinGen Actionability Reports: BRCA1, BRCA2 Adult. ver.1.1.3 (2020.9.15)

監修：厚労科研小杉班・Actionability Working Group-Japan

編集： MONSTAR-SCREEN-2遺伝事務局

***BRIP1*遺伝子について**

*がんの発症と関連する遺伝子の変化について*

- - 一般的にがんの5~10%は「生まれつきの遺伝子の変化」が原因といわれています。
  - 「生まれつきの遺伝子の変化」をもっている場合、がんになりやすい体質をもっていると考えられます。
  - がんになりやすい体質は親、子、兄弟、姉妹 などの血のつながった家族と共有している可能性があります。
  - 体質を知ることで、その情報をご自身やご家族の健康管理に役立てることができます。
  - 今回の遺伝子検査でみつかった変化が「生まれつきの遺伝子の変化」かどうかは、血液検査により確認できます。

*BRIP1*遺伝子とは？

- *がんを発症するリスクと関連することが示されている遺伝子です。裏面の資料* (*表*) *をご参照ください*。

「生まれつきの遺伝子変化」であるかどうかを知ることはどんなことに役立ちますか？

- 発症リスクが高いがんを知り、それに対する検診を行うことで、早期発見につながる可能性があります。

家族にどんな影響がありますか？
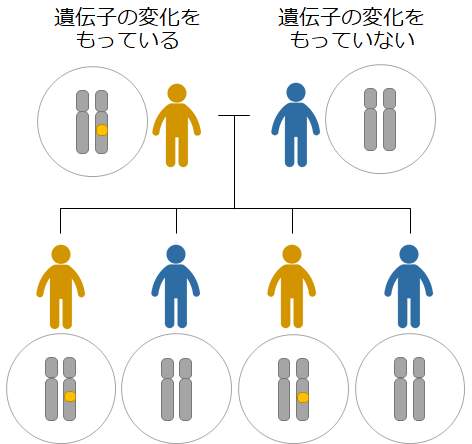


- 人は２つの*BRIP1*遺伝子をそれぞれ両親から1つずつ受け継いでいます。
- *BRIP1*遺伝子の変化を生まれつきもっていた場合、両親、兄弟、姉妹、子どもが同じ変化をもっている確率は50%です。同じ変化をもっているかは、遺伝子検査で確認することができます。
- おば、おじ、いとこなども一定の確率で*同じ遺伝子の変化をもっている*可能性があります。
- 遺伝子の変化についてご家族の方と情報共有することは、ご家族の方の健康管理に役立つ可能性があります。

※生まれつきの*BRIP1*遺伝子の変化は、両親いずれかから受け継いだものではなく、新規に起こった変化である可能性もあります。この場合のご家族への影響については、遺伝専門外来におたずねください。

遺伝カウンセリングについて

遺伝カウンセリングでは、生まれつきの遺伝子の変化と体質との関連、ご家族への影響などについてより詳しくご説明します。お気軽にご相談ください。 注意：遺伝学的検査の保険適用には条件があり、関連する症状を発症していないご家族の遺伝子検査や検診は自費となります。詳しくは担当者におたずね下さい。

＜参考資料表＞

|  | 一般的な日本人 | *BRIP1遺伝子*に病気の原因となる変化を生まれつきもっている | | | |
| --- | --- | --- | --- | --- | --- |
|  | 生涯を通して  罹患する確率^1)^ | 生涯を通して  罹患する確率 | | 推奨される対応 | |
|  |  | 女性^2)^ | 男性 | 女性^2)^ | 男性 |
| 卵巣がん | 1.6% (女性) | >10 % | ― | 45~50歳以降:リスク低減卵管卵巣摘出術 (RRSO) を考慮（注）  ※家族歴や既往歴によってリスクは異なると考えられています。 | ― |
| 乳がん | 10.9 ％ (女性) | リスクが上がる  可能性 | ― | ※家族歴や既往歴によってリスクは異なると考えられています。 | ― |

(注) 記載の推奨事項については、本邦での具体的な検診方法は示されていません。詳細は各施設の遺伝専門外来におたずねください。

※*BRIP1遺伝子*の変化が生まれつきの変化であっても、必ずがんを発症するというわけではありません。

※遺伝子の変化をもっていた場合の罹患する確率、推奨される対応の記載は米国のガイドライン (NCCNガイドライン)^2)^ を参照しています。

※表は2021年時点の記載です。今後、研究が進み推奨内容が変わる可能性があります。

【参考文献】

- 国立がん研究センターがん情報サービス「累積がん罹患リスク (2018年データ)」https://ganjoho.jp/reg_stat/statistics/stat/summary.html [ref. 1]
- NCCN Guidelines® Genetic/Familial High-Risk Assessment: Breast, Ovarian, and Pancreatic. ver.1.2022 (2021.8.11) [ref. 2]
- ClinGen Actionability Reports: BRIP1, RAD51C, RAD51D Adult. ver.1.1.1 (2020.7.13)

監修：厚労科研小杉班・Actionability Working Group-Japan

編集： MONSTAR-SCREEN-2遺伝事務局

***CDH1*遺伝子について**

*がんの発症と関連する遺伝子の変化について*

- - 一般的にがんの5~10%は「生まれつきの遺伝子の変化」が原因といわれています。
  - 「生まれつきの遺伝子の変化」をもっている場合、がんになりやすい体質をもっていると考えられます。
  - がんになりやすい体質は親、子、兄弟、姉妹 などの血のつながった家族と共有している可能性があります。
  - 体質を知ることで、その情報をご自身やご家族の健康管理に役立てることができます。
  - 今回の遺伝子検査でみつかった変化が「生まれつきの遺伝子の変化」かどうかは、血液検査により確認できます。

*CDH1*遺伝子とは？

- *CDH1遺伝子*の生まれつきの変化は**遺伝性びまん性胃がん**の原因となることが知られています。裏面の資料 (表) をご参照ください。

「生まれつきの遺伝子の変化」であるかどうかを知ることはどんなことに役立ちますか？

- 発症リスクが高いがんを知り、それに対する検診を行うことで、早期発見につながる可能性があります。

家族にどんな影響がありますか？
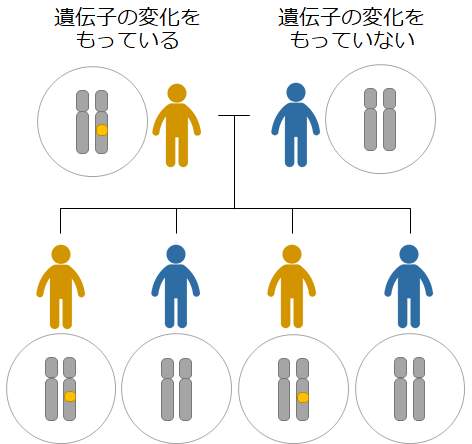


- 人は２つの*CDH1*遺伝子をそれぞれ両親から1つずつ受け継いでいます。
- *CDH1*遺伝子の変化を生まれつきもっていた場合、あなたの両親、兄弟、姉妹、子どもが同じ変化をもっている確率は50%です。同じ変化をもっているかは、遺伝子検査で確認することができます。
- おば、おじ、いとこなども一定の確率で*同じ遺伝子の変化をもっている*可能性があります。
- 遺伝子の変化についてご家族の方と情報共有することは、ご家族の方の健康管理に役立つ可能性があります。

※生まれつきの*CDH1*遺伝子の変化は、両親から受け継いだものではなく、新規に起こった変化である可能性もあります。この場合のご家族への影響については、遺伝専門外来におたずねください。

遺伝カウンセリングについて

遺伝カウンセリングでは、生まれつきの遺伝子の変化と体質との関連、ご家族への影響などについてより詳しくご説明します。お気軽にご相談ください。　注意：遺伝学的検査の保険適用には条件があり、関連する症状を発症していないご家族の遺伝子検査や検診は自費となります。詳しくは担当者におたずね下さい。

＜参考資料表＞

|  | 一般的な日本人 | *CDH1*遺伝子に病気の原因となる変化を生まれつきもっている | | | |
| --- | --- | --- | --- | --- | --- |
|  | 生涯を通して  罹患する確率^1)^ | 生涯を通して  罹患する確率 | | 推奨される対応 | |
|  |  | 女性^2,3)^ | 男性^2,3)^ | 女性^2,3)^ | 男性^2,3)^ |
| 乳がん | 10.9 ％ (女性) | 39~52 % | ― | 30歳以降：マンモグラフィー/乳房MRI (年１回) (注) | ― |
| 胃がん | 10.3 ％ (男性)  4.7 ％ (女性) | （80歳までに）83 % | （80歳までに）67 % | 上部内視鏡検査と無作為生検によるスクリーニング  (半年～１年ごと) (注)  18歳～40歳までの間に：予防的胃全摘術を推奨。  (※家族の発症年齢等に応じて) (注)  家族歴や既往歴によってリスクは異なると考えられています。 | |

(注) 記載の推奨事項については、本邦での具体的な検診方法は示されていません。詳細は各施設の遺伝専門外来におたずねください。

※*CDH1遺伝子*の変化が生まれつきの変化であっても、必ずがんを発症するというわけではありません。

※遺伝子の変化をもっていた場合の罹患する確率、推奨される対応の記載は米国のガイドライン (NCCNガイドライン)^2,3)^ を参照しています。

※表は2021年時点の記載です。今後、研究が進み推奨内容が変わる可能性があります。

【参考文献】

- 国立がん研究センターがん情報サービス「累積がん罹患リスク (2018年データ)」<https://ganjoho.jp/reg_stat/statistics/stat/summary.html> [ref. 1]
- NCCN Guidelines® Genetic/Familial High-Risk Assessment: Breast, Ovarian, and Pancreatic. ver.1.2022 (2021.8.11) [ref. 2]
- NCCN Guidelines® Gastric cancer. ver.2.2022 (2022.1.11) [ref. 3]
- GeneReviews®: Hereditary Diffuse Gastric Cancer. ver.2018.3.22.
- ClinGen Actionability Reports: CDH1 Adult. ver.1.1.3 (2021.9.3)

監修：厚労科研小杉班・Actionability Working Group-Japan

編集： MONSTAR-SCREEN-2遺伝事務局

***CDK4*遺伝子について**

*がんの発症と関連する遺伝子の変化について*

- - 一般的にがんの5~10%は「生まれつきの遺伝子の変化」が原因といわれています。
  - 「生まれつきの遺伝子の変化」をもっている場合、がんになりやすい体質をもっていると考えられます。
  - がんになりやすい体質は親、子、兄弟、姉妹 などの血のつながった家族と共有している可能性があります。
  - 体質を知ることで、その情報をご自身やご家族の健康管理に役立てることができます。
  - 今回の遺伝子検査でみつかった変化が「生まれつきの遺伝子の変化」かどうかは、血液検査により確認できます。

*CDK4*遺伝子とは？

- *がんを発症するリスクと関連することが示されている遺伝子です*^1,2)^*。*裏面の資料 (表) をご参照ください。

「生まれつきの遺伝子の変化」であるかどうかを知ることはどんなことに役立ちますか？

- 発症リスクが高いがんを知り、それに対する検診を行うことで、早期発見につながる可能性があります。

家族にどんな影響がありますか？
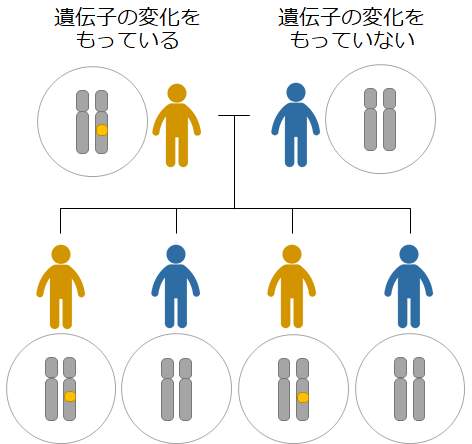


- 人は２つの*CDK4*遺伝子をそれぞれ両親から1つずつ受け継いでいます。
- 遺伝子の変化を生まれつきもっていた場合、両親、兄弟、姉妹、子どもが同じ変化をもっている確率は50%です。同じ変化をもっているかは、遺伝子検査で確認することができます。
- おば、おじ、いとこなども一定の確率で*同じ遺伝子の変化をもっている*可能性があります。
- 遺伝子の変化についてご家族の方と情報共有することは、ご家族の方の健康管理に役立つ可能性があります。

※生まれつきの*CDK4*遺伝子の変化は、両親いずれかから受け継いだものではなく、新規に起こった変化である可能性もあります。この場合のご家族への影響については、遺伝専門外来におたずねください。

遺伝カウンセリングについて

遺伝カウンセリングでは、生まれつきの遺伝子の変化と体質との関連、ご家族への影響などについてより詳しくご説明します。お気軽にご相談ください。　注意：遺伝学的検査の保険適用には条件があり、関連する症状を発症していないご家族の遺伝子検査や検診は自費となります。詳しくは担当者におたずね下さい。

＜参考資料表＞

|  | 一般的な日本人 | *CDK4*遺伝子に病気の原因となる変化を生まれつきもっている | | | |
| --- | --- | --- | --- | --- | --- |
|  | 生涯を通して  罹患する確率^3,4)^ | 生涯を通して  罹患する確率 | | 推奨される対応 | |
|  |  | 女性^1,2)^ | 男性^1,2)^ | 女性^5,6)^ | 男性^5,6)^ |
| 悪性黒色腫 (注1) | 0.1 % (男性)  0.1 % (女性) | リスクが高まる可能性 | | 皮膚、頭皮、口腔粘膜、生殖器の皮膚科での精密検査 (半年に1回、母斑が安定している場合は年１回) (注2)  月１回の母斑自己検診（注2） | |
| 膵がん | 2.6 % (男女) | リスクが高まる可能性 | | 膵臓の超音波内視鏡/MRI検査：40歳あるいは家族で最も若い膵がん診断年齢より10歳若い年齢以降 (注2) | |

(注1) 悪性黒色腫の罹病率 (年) は、白人が 24.3 人/10 万人、アジア人が 1.7 人/10 万人との報告があり^7)^、人種、地域、その他の遺伝的要因によってリスクは異なると考えられています。

(注2) 記載の推奨事項については、本邦での具体的な検診方法は示されていません。詳細は各施設の遺伝専門外来におたずねください。

※*CDK4遺伝子*の変化が生まれつきの変化であっても、必ずがんを発症するというわけではありません。

※遺伝子の変化をもっていた場合の罹患する確率、推奨される対応は海外の資料^1,2,5,6)^を参照しています。

※表は2021年時点の記載です。今後、研究が進み推奨内容が変わる可能性があります。

【参考文献】

- Hereditary melanoma: update on syndrome and management -Genetics of familial atypical multiple mole melanoma syndrome. J Am Acad Dernatol, 2016; 74(3): 395. [ref. 1]
- Genotype-phenotype relationships in U.S. melanoma-prone families with *CDKN2A* and *CDK4* mutations. J Natl Cancer Inst, 2000; 92(12): 1006. [ref. 2]
- 厚生労働省健康局がん・疾病対策課. 平成30年全国がん登録 罹患数・率報告 2018 [ref. 3]
- 国立がん研究センターがん情報サービス「累積がん罹患リスク (2018年データ)」 <https://ganjoho.jp/reg_stat/statistics/stat/summary.html> [ref. 4]
- ClinGen Actionability Reports: CDKN2A Adult. ver.1.2.1 (2020.8.19) [ref. 5]
- NCCN Guidelines® Genetic/Familial High-Risk Assessment: Breast, Ovarian, and Pancreatic. ver.1.2022 (2021.8.11) [ref. 6]
- 日本皮膚科学会. 皮膚悪性腫瘍ガイドライン第 3 版 メラノーマ診療ガイドライン 2019. 日皮会誌, 2019; 129(9): 1759. [ref. 7]

監修：厚労科研小杉班・Actionability Working Group-Japan

編集： MONSTAR-SCREEN-2遺伝事務局

***CDKN2A*遺伝子について**

*がんの発症と関連する遺伝子の変化について*

- - 一般的にがんの5~10%は「生まれつきの遺伝子の変化」が原因といわれています。
  - 「生まれつきの遺伝子の変化」をもっている場合、がんになりやすい体質をもっていると考えられます。
  - がんになりやすい体質は親、子、兄弟、姉妹 などの血のつながった家族と共有している可能性があります。
  - 体質を知ることで、その情報をご自身やご家族の健康管理に役立てることができます。
  - 今回の遺伝子検査でみつかった変化が「生まれつきの遺伝子の変化」かどうかは、血液検査により確認できます。

*CDKN2A*遺伝子とは？

- *CDKN2A遺伝子*の生まれつきの変化は**膵臓がん・悪性黒色腫*症候群***の原因となることが知られています。裏面の資料 (表) をご参照ください。

「生まれつきの遺伝子の変化」であるかどうかを知ることはどんなことに役立ちますか？

- 発症リスクが高いがんを知り、それに対する検診を行うことで、早期発見につながる可能性があります。

家族にどんな影響がありますか？
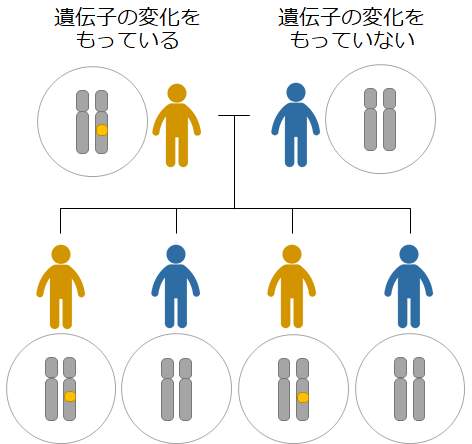


- 人は２つの*CDKN2A*遺伝子をそれぞれ両親から1つずつ受け継いでいます。
- *CDKN2A*遺伝子の変化を生まれつきもっていた場合、両親、兄弟、姉妹、子どもが同じ変化をもっている確率は50%です。同じ変化をもっているかは、遺伝子検査で確認することができます。
- おば、おじ、いとこなども一定の確率で*同じ遺伝子の変化をもっている*可能性があります。
- 遺伝子の変化についてご家族の方と情報共有することは、ご家族の方の健康管理に役立つ可能性があります。

※生まれつきの*CDKN2A*遺伝子の変化は、両親いずれかから受け継いだものではなく、新規に起こった変化である可能性もあります。この場合のご家族への影響については、遺伝専門外来におたずねください。

遺伝カウンセリングについて

遺伝カウンセリングでは、生まれつきの遺伝子の変化と体質との関連、ご家族への影響などについてより詳しくご説明します。お気軽にご相談ください。　注意：遺伝学的検査の保険適用には条件があり、関連する症状を発症していないご家族の遺伝子検査や検診は自費となります。詳しくは担当者におたずね下さい。

＜参考資料表＞

|  | 一般的な日本人 | *CDKN2A*遺伝子に病気の原因となる変化を生まれつきもっている | | | |
| --- | --- | --- | --- | --- | --- |
|  | 生涯を通して  罹患する確率^1,2)^ | 生涯を通して  罹患する確率 | | 推奨される対応 | |
|  |  | 女性^3,4)^ | 男性^3,4)^ | 女性^3,4,5)^ | 男性^3,4,5)^ |
| 膵がん | 2.6 %  （男女） | >15 %  （一般の20~47倍） | | 膵臓の超音波内視鏡/MRI検査：40歳あるいは家族で最も若い膵がん診断年齢より10歳若い年齢以降(注2) | |
| 悪性黒色腫  (注1) | 0.1 % (男性)  0.1 % (女性) | 28～76％ | | 皮膚、頭皮、口腔粘膜、生殖器の皮膚科での精密検査（半年に1回、母斑が安定している場合は年１回) (注2)  月１回の母斑自己検診 (注2) | |

(注1) 悪性黒色腫の罹病率 (年) は、白人が 24.3 人/10 万人、アジア人が 1.7 人/10 万人との報告があり^6)^、人種、地域、その他の遺伝的要因によってリスクは異なると考えられています。

(注2) 記載の推奨事項については、本邦での具体的な検診方法は示されていません。詳細は各施設の遺伝専門外来におたずねください。

※*CDKN2A遺伝子*の変化が生まれつきの変化であっても、必ずがんを発症するというわけではありません。

※遺伝子の変化をもっていた場合の罹患する確率、推奨される対応の記載は海外の資料^3,4,5)^ を参照しています。

※表は2021年時点の記載です。今後、研究が進み推奨内容が変わる可能性があります。

【参考文献】

- 国立がん研究センターがん情報サービス「累積がん罹患リスク (2018年データ)」 <https://ganjoho.jp/reg_stat/statistics/stat/summary.html> [ref. 1]
- 厚生労働省健康局がん・疾病対策課 平成30年全国がん登録 罹患数・率報告 2018 [ref. 2]
- NCCN Guidelines® Genetic/Familial High-Risk Assessment: Breast, Ovarian, and Pancreatic. ver.1.2022 (2021.8.11) [ref. 3]
- NCCN Guidelines®: Pancreatic Adenocarcinoma. ver.2.2021 (2021.2.25) [ref. 4]
- ClinGen Actionability Reports: CDKN2A Adult. ver.1.2.1 (2020.8.19) [ref. 5]
- 日本皮膚科学会. 皮膚悪性腫瘍ガイドライン第 3 版 メラノーマ診療ガイドライン 2019. 日皮会誌, 2019; 129(9):1759. [ref. 6]

監修：厚労科研小杉班・Actionability Working Group-Japan

編集： MONSTAR-SCREEN-2遺伝事務局

***CHEK2*遺伝子について**

*がんの発症と関連する遺伝子の変化について*

- - 一般的にがんの5~10%は「生まれつきの遺伝子の変化」が原因といわれています。
  - 「生まれつきの遺伝子の変化」をもっている場合、がんになりやすい体質をもっていると考えられます。
  - がんになりやすい体質は親、子、兄弟、姉妹 などの血のつながった家族と共有している可能性があります。
  - 体質を知ることで、その情報をご自身やご家族の健康管理に役立てることができます。
  - 今回の遺伝子検査でみつかった変化が「生まれつきの遺伝子の変化」かどうかは、血液検査により確認できます。

*CHEK2*遺伝子とは？

- *がんを発症するリスクと関連することが示されている遺伝子です。裏面の資料* (*表*) *をご参照ください。*

「生まれつきの遺伝子変化」であるかどうかを知ることはどんなことに役立ちますか？

- 発症リスクが高いがんを知り、それに対する検診を行うことで、早期発見につながる可能性があります。

家族にどんな影響がありますか？
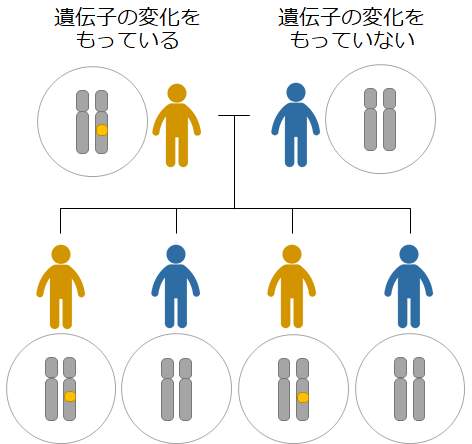


- 人は２つの*CHEK2*遺伝子をそれぞれ両親から1つずつ受け継いでいます。
- *CHEK2*遺伝子の変化を生まれつきもっていた場合、両親、兄弟、姉妹、子どもが同じ変化をもっている確率は50%です。同じ変化をもっているかは、遺伝子検査で確認することができます。
- おば、おじ、いとこなども一定の確率で*同じ遺伝子の変化をもっている*可能性があります。
- 遺伝子の変化についてご家族の方と情報共有することは、ご家族の方の健康管理に役立つ可能性があります。

※生まれつきの*CHEK2*遺伝子の変化は、両親いずれかから受け継いだものではなく、新規に起こった変化である可能性もあります。この場合のご家族への影響については、遺伝専門外来におたずねください。

遺伝カウンセリングについて

遺伝カウンセリングでは、生まれつきの遺伝子の変化と体質との関連、ご家族への影響などについてより詳しくご説明します。お気軽にご相談ください。 注意：遺伝学的検査の保険適用には条件があり、関連する症状を発症していないご家族の遺伝子検査や検診は自費となります。詳しくは担当者におたずね下さい。

＜参考資料表＞

|  | 一般的な日本人 | *CHEK2遺伝子*に病気の原因となる変化を生まれつきもっている | | | | |
| --- | --- | --- | --- | --- | --- | --- |
|  | 生涯を通して  罹患する確率^1)^ | | 生涯を通して  罹患する確率 | | 推奨される対応 | |
|  |  |  | 女性^2,3)^ | 男性^2,3)^ | 女性^2,3)^ | 男性^2,3)^ |
| 乳がん | 10.9 ％ (女性) | | 15~40 ％ | ― | 40歳以降:年1回のマンモグラフィと乳房造影MRI検査 (注) | ― |
| 大腸がん | 8.0 % (女性)　10.2 % (男性) | | リスクが上がる可能性 | | 40歳 (または第一度近親者の大腸がん診断時年齢より10歳若い年齢) 以降:5年ごとの大腸内視鏡検査 (注) | |

(注) 記載の推奨事項については、本邦での具体的な検診方法は示されていません。詳細は各施設の遺伝専門外来におたずねください。

※*CHEK2遺伝子*の変化が生まれつきの変化であっても、必ずがんを発症するというわけではありません。

※上表は*CHEK2*遺伝子の一部の種類の変化におけるデータです。遺伝子の変化の種類に応じて検診の方法や頻度を検討することが推奨されています。

※遺伝子の変化をもっていた場合の罹患する確率、推奨される対応の記載は米国のガイドライン (NCCNガイドライン) ^2,3)^ を参照しています。

※表は2021年時点の記載です。今後、研究が進み推奨内容が変わる可能性があります。

【参考文献】

- 国立がん研究センターがん情報サービス「累積がん罹患リスク (2018年データ)」https://ganjoho.jp/reg_stat/statistics/stat/summary.html [ref. 1]
- NCCN Guidelines® Genetic/Familial High-Risk Assessment: Breast, Ovarian, and Pancreatic. ver.1.2022 (2021.8.11) [ref. 2]
- NCCN Guidelines® Genetic/Familial High-Risk Assessment: Colorectal. ver.1.2021 (2021.5.11) [ref. 3]
- ClinGen Actionability Reports: ATM, CHEK2 Adult. ver.1.1.1 (2020.4.16)

監修：厚労科研小杉班・Actionability Working Group-Japan

編集： MONSTAR-SCREEN-2遺伝事務局

***EPCAM*遺伝子について**

*がんの発症と関連する遺伝子の変化について*

- - 一般的にがんの5~10%は「生まれつきの遺伝子の変化」が原因といわれています。
  - 「生まれつきの遺伝子の変化」をもっている場合、がんになりやすい体質をもっていると考えられます。
  - がんになりやすい体質は親、子、兄弟、姉妹 などの血のつながった家族と共有している可能性があります。
  - 体質を知ることで、その情報をご自身やご家族の健康管理に役立てることができます。
  - 今回の遺伝子検査でみつかった変化が「生まれつきの遺伝子の変化」かどうかは、血液検査により確認できます。

*EPCAM*遺伝子とは？

- *EPCAM遺伝子*の生まれつきの変化は**リンチ症候群**の原因となることが知られています。裏面の資料 (表) をご参照ください。

「生まれつきの遺伝子の変化」であるかどうかを知ることはどんなことに役立ちますか？

- 発症リスクが高いがんを知り、それに対する検診を行うことで、早期発見につながる可能性があります。

家族にどんな影響がありますか？
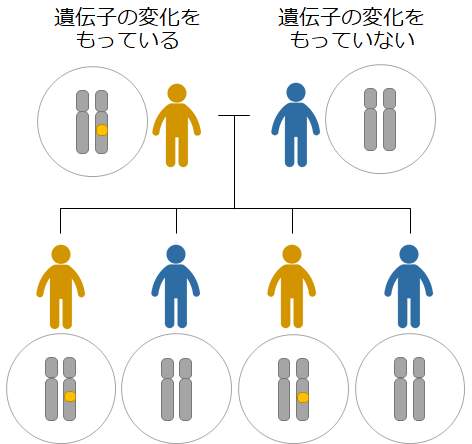


- 人は２つの*EPCAM*遺伝子をそれぞれ両親から1つずつ受け継いでいます。
- *EPCAM*遺伝子の変化を生まれつきもっていた場合、両親、兄弟、姉妹、子どもが同じ変化をもっている確率は50%です。同じ変化をもっているかは、遺伝子検査で確認することができます。
- おば、おじ、いとこなども一定の確率で*同じ遺伝子の変化をもっている*可能性があります。
- 遺伝子の変化についてご家族の方と情報共有することは、ご家族の方の健康管理に役立つ可能性があります。

※生まれつきの*EPCAM*遺伝子の変化は、両親いずれかから受け継いだものではなく、新規に起こった変化である可能性もあります。この場合のご家族への影響については、遺伝専門外来におたずねください。

遺伝カウンセリングについて

遺伝カウンセリングでは、生まれつきの遺伝子の変化と体質との関連、ご家族への影響などについてより詳しくご説明します。お気軽にご相談ください。　注意：遺伝学的検査の保険適用には条件があり、関連する症状を発症していないご家族の遺伝子検査や検診は自費となります。詳しくは担当者におたずね下さい。

＜参考資料表＞

|  | 一般的な日本人 | *EPCAM*遺伝子に病気の原因となる変化を生まれつきもっている | | | |
| --- | --- | --- | --- | --- | --- |
|  | 生涯を通して  罹患する確率^1)^ | 80歳までに  罹患する確率 | | 推奨される対応 | |
|  |  | 女性^2)^ | 男性^2)^ | 女性^3)^ | 男性^3)^ |
| 大腸がん | 10.2 ％（男性）  8.0 ％（女性） | 33~52 % | | 20~25歳以降：  1~2年ごとに大腸内視鏡検査 | |
| 子宮内膜がん | 2.0%（女性） | 21~57 % | ― | 30~35歳以降：1年ごとに経腟超音波検査、子宮内膜組織診 (または細胞診)、(血清マーカー検査) |  |
| 卵巣がん | 1.6% (女性) | 8~38 % | ― |  |  |
| 胃がん | 10.3 ％ (男性)  4.7 ％ (女性) | 0.2~9 % (注) | | (胃・十二指腸がんの家族歴がある場合に考慮)  30~35歳以降：  ヘリコバクター・ピロリ菌除菌  1~3年ごとに上部消化管内視鏡検査 | |
| 腎盂・尿管がん | 2.2 % (男性)  1.1 % (女性) | 2.2~28 % | | (尿路上皮がんの家族歴がある場合に考慮)  30~35歳以降：  1年ごとに検尿 (または尿細胞診) | |
| 膀胱がん | 2.1 % (男性)  0.7 % (女性) | 4.4~12.8 % | |  |  |

(注) 記載した確率は、東アジア人では欧米諸国に比べ高いことが知られています^4)^。リンチ症候群と診断された日本人において60~70歳までに胃がんに罹患する確率は14.5~24%との報告^5,6)^があります。

※*EPCAM遺伝子*の変化が生まれつきの変化であっても、必ずがんを発症するというわけではありません。

※遺伝子の変化をもっていた場合の罹患する確率、推奨される対応の記載は日本の診療ガイドライン^3)^および海外のガイドライン (米国)^2)^ を参照しています。推奨事項については、各医療機関において必ずしも実施されているとは限りませんので、詳細については各施設の専門診療科あるいは遺伝専門外来におたずねください。

※表は2021年時点の記載です。今後、研究が進み推奨内容が変わる可能性があります。

【参考文献】

- 国立がん研究センターがん情報サービス「累積がん罹患リスク (2018年データ)」 <https://ganjoho.jp/reg_stat/statistics/stat/summary.html> [ref. 1]
- NCCN Guidelines® Genetic/Familial High-Risk Assessment: Colorectal. ver.1.2021 (2021.5.11) [ref. 2]
- 大腸癌研究会編. 遺伝性大腸癌診療ガイドライン 2020年版. (2020年4月) [ref. 3]
- Managing gastric cancer risk in lynch syndrome: controversies and recommendations. Fam Cancer, 2021 (online ahead of print) [ref. 4]
- Tumor development in Japanese patients with Lynch syndrome. PLoS One, 2018; 13(4): e0195572 [ref. 5]
- Comparison of clinical features between suspected familial colorectal cancer type X and Lynch syndrome in Japanese patients with colorectal cancer: a cross-sectional study conducted by the Japanese Society for Cancer of the Colon and Rectum. Jpn J Clin Oncol, 2015; 45(2): 153. [ref. 6]
- GeneReviews Japan: リンチ症候群. ver.2021.4.21
- ClinGen Actionability Reports: MLH1, MSH2, MSH6, PMS2, EPCAM Adult. ver.1.1.2 (2021.9.15)

監修：厚労科研小杉班・Actionability Working Group-Japan

編集： MONSTAR-SCREEN-2遺伝事務局

***FH*遺伝子について**

*がんの発症と関連する遺伝子の変化について*

- - 一般的にがんの5~10%は「生まれつきの遺伝子の変化」が原因といわれています。
  - 「生まれつきの遺伝子の変化」をもっている場合、がんになりやすい体質をもっていると考えられます。
  - がんになりやすい体質は親、子、兄弟、姉妹 などの血のつながった家族と共有している可能性があります。
  - 体質を知ることで、その情報をご自身やご家族の健康管理に役立てることができます。
  - 今回の遺伝子検査でみつかった変化が「生まれつきの遺伝子の変化」かどうかは、血液検査により確認できます。

*FH*遺伝子とは？

- *FH遺伝子*の生まれつきの変化は**遺伝性平滑筋腫症・腎細胞がん症候群**の原因となることが知られています。裏面の資料 (表) をご参照ください。

「生まれつきの遺伝子の変化」であるかどうかを知ることはどんなことに役立ちますか？

- 発症リスクが高い腫瘍を知り、それに対する検診を行うことで、早期発見につながる可能性があります。

家族にどんな影響がありますか？
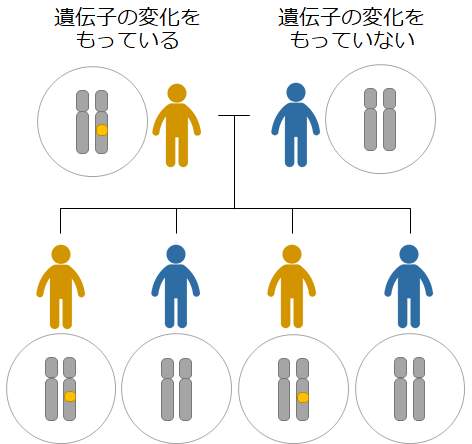


- 人は２つの*FH*遺伝子をそれぞれ両親から1つずつ受け継いでいます。
- *FH*遺伝子の変化を生まれつきもっていた場合、あなたの両親、兄弟、姉妹、子どもが同じ変化をもっている確率は50%です。同じ変化をもっているかは、遺伝子検査で確認することができます。
- おば、おじ、いとこなども一定の確率で*同じ遺伝子の変化をもっている*可能性があります。
- 遺伝子の変化についてご家族の方と情報共有することは、ご家族の方の健康管理に役立つ可能性があります。

※生まれつきの*FH*遺伝子の変化は、両親いずれかから受け継いだものではなく、新規に起こった変化である可能性もあります。この場合のご家族への影響については、遺伝専門外来におたずねください。

遺伝カウンセリングについて

遺伝カウンセリングでは、生まれつきの遺伝子の変化と体質との関連、ご家族への影響などについてより詳しくご説明します。お気軽にご相談ください。　注意：遺伝学的検査の保険適用には条件があり、関連する症状を発症していないご家族の遺伝子検査や検診は自費となります。詳しくは担当者におたずね下さい。

＜参考資料表＞

|  | 一般的な日本人 | *FH*遺伝子に病気の原因となる変化を生まれつきもっている | | | | |
| --- | --- | --- | --- | --- | --- | --- |
|  | 生涯を通して罹患する確率^1,2,3,4)^ | 生涯を通して  罹患する確率 | | | 推奨される対応 | |
|  |  | 女性^5,6)^ | 男性^5,6)^ | | 女性^5,7,8,9)^ | 男性^5,7,8,9)^ |
| 皮膚平滑筋腫  皮膚平滑筋肉腫 | ※発生頻度：悪性軟部腫瘍は100万人あたり年間40例 | 76 %  （皮膚平滑筋腫） | | | 皮膚科の精密検査 (年に1～2回) (注) | |
| 子宮平滑筋腫  子宮平滑筋肉腫 | (子宮体がん)  ２％ | 77 %  （子宮平滑筋腫） | | ― | 婦人科診察 (年1回)：20歳以降 (または症状がある場合は20歳以前に) (注) | ― |
| 腎細胞がん | (腎 (腎盂を除く))  1.2% (男性)  0.4% (女性) | 10~16 %  ※1.7~5.8%との推定値もあり | | | 腎腫瘍の評価 (腎超音波検査および MRI、年1回)  ：8～10歳以降 (注) | |
| 褐色細胞腫  /パラガングリオーマ | ※患者数全国で  約1500人/年 | リスクが上がる可能性 | | | 既往・家族歴のある人は、ベースラインに全身MRIと血中遊離メタネフリン測定を考慮 (注) | |

(注) 記載の推奨事項については、本邦での具体的な検診方法は示されていません。詳細は各施設の遺伝専門外来におたずねください。

※*FH遺伝子*の変化が生まれつきの変化であっても、必ず症状がみられるというわけではありません。

※遺伝子の変化をもっていた場合の罹患する確率、推奨される対応の記載は海外の資料^5,7,8)^ を参照しています。

※表は2021年時点の記載です。今後、研究が進み推奨内容が変わる可能性があります。

【参考文献】.

- 日本整形外科学会. 軟部腫瘍診療ガイドライン2020 改訂第3版. (2020年7月) [ref. 1]
- 国立がん研究センターがん情報サービス「累積がん罹患リスク (2018年データ)」 <https://ganjoho.jp/reg_stat/statistics/stat/summary.html> [ref. 2]
- 厚生労働省健康局がん・疾病対策課. 平成30年全国がん登録 罹患数・率報告 2018. [ref. 3]
- 国立がん研究センター希少がんセンター: パラガングリオーマ <https://www.ncc.go.jp/jp/rcc/about/paraganguriouma/index.html> [ref. 4]
- ClinGen Actionability Reports: FH Adult. ver.1.2.1 (2020.4.27) [ref. 5]
- Estimation of the carrier frequency of fumarase hydratase alterations and implications for kidney cancer risk in hereditary leiomyomatosis and renal cancer. Cancer, 2020; 126(16): 3657. [ref. 6]
- NCCN Guidelines®: Kidney Cancer. ver.4.2022 (2021.12.21) [ref. 7]
- GeneReviews Japan: *FH*腫瘍易罹患性症候群. ver.2021.5.1 [ref. 8]
- 日本内分泌学会. 褐色細胞腫・パラガングリオーマ診療ガイドライン2018. (2018年7月) [ref. 9]

監修：厚労科研小杉班・Actionability Working Group-Japan

編集： MONSTAR-SCREEN-2遺伝事務局

***FLCN*遺伝子について**

*腫瘍と関連する遺伝子の変化について*

- - 一般的に腫瘍の一部は「生まれつきの遺伝子の変化」が原因といわれています。
  - 「生まれつきの遺伝子の変化」をもっている場合、腫瘍ができやすい体質をもっていると考えられます。
  - 腫瘍ができやすい体質は親、子、兄弟、姉妹 などの血のつながった家族と共有している可能性があります。
  - 体質を知ることで、その情報をご自身やご家族の健康管理に役立てることができます。
  - 今回の遺伝子検査でみつかった変化が「生まれつきの遺伝子の変化」かどうかは、血液検査により確認できます。

*FLCN*遺伝子とは？

- *FLCN遺伝子*の生まれつきの変化は**Birt-Hogg-Dubé (バート・ホッグ・デュベ) 症候群**の原因となることが知られています。裏面の資料 (表) をご参照ください。

「生まれつきの遺伝子の変化」であるかどうかを知ることはどんなことに役立ちますか？

- 発症リスクが高い症状を知り、それに対する検診を行うことで、早期発見につながる可能性があります。

家族にどんな影響がありますか？
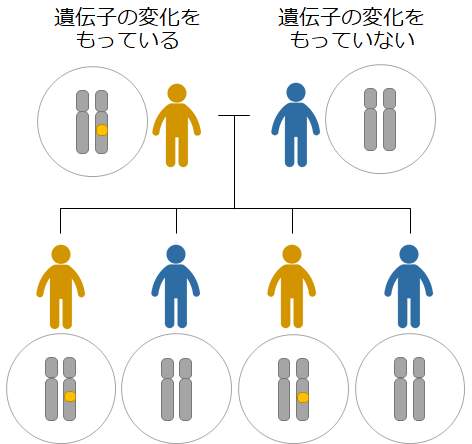


- 人は２つの*FLCN*遺伝子をそれぞれ両親から1つずつ受け継いでいます。
- *FLCN*遺伝子の変化を生まれつきもっていた場合、両親、兄弟、姉妹、子どもが同じ変化をもっている確率は50%です。同じ変化をもっているかは、遺伝子検査で確認することができます。
- おば、おじ、いとこなども一定の確率で*同じ遺伝子の変化をもっている*可能性があります。
- 遺伝子の変化についてご家族の方と情報共有することは、ご家族の方の健康管理に役立つ可能性があります。

※生まれつきの*FLCN*遺伝子の変化は、両親いずれかから受け継いだものではなく、新規に起こった変化である可能性もあります。この場合のご家族への影響については、遺伝専門外来におたずねください。

遺伝カウンセリングについて

遺伝カウンセリングでは、生まれつきの遺伝子の変化と体質との関連、ご家族への影響などについてより詳しくご説明します。お気軽にご相談ください。　注意：遺伝学的検査の保険適用には条件があり、関連する症状を発症していないご家族の遺伝子検査や検診は自費となります。詳しくは担当者におたずね下さい。

＜参考資料表＞

|  | 一般的な日本人 | *FLCN*遺伝子に病気の原因となる変化を生まれつきもっている | | | |
| --- | --- | --- | --- | --- | --- |
|  | 生涯を通して罹患する確率^1)^ | 生涯を通して  罹患する確率 | | 推奨される対応 | |
|  |  | 女性^2)^ | 男性^2)^ | 女性^3,4)^ | 男性^3,4)^ |
| 肺嚢胞 | 不明 | 89 % | | 高分解能CT (HRCT) または胸部CT検査  ：気胸の兆候がある場合、適切なフォローアップを行う。(注) | |
| 腎腫瘍 | (腎 (腎盂を除く))  1.2% (男性)  0.4% (女性) | 6.5~34 % | | 腹部MRI (推奨) またはCT (３年に1回)  ：20歳以降 (注) | |
| 線維毛包腫 | 不明 | 73~85 % | | 皮膚科の定期診察 (注) | |

(注) 記載の推奨事項については、本邦での具体的な検診方法は示されていません。詳細は各施設の遺伝専門外来におたずねください。

※*FLCN遺伝子*の変化が生まれつきの変化であっても、必ず症状がみられるというわけではありません。

※遺伝子の変化をもっていた場合の罹患する確率、推奨される対応の記載は海外の資料^1,3)^ を参照しています。

※表は2021年時点の記載です。今後、研究が進み推奨内容が変わる可能性があります。

【参考文献】

- 厚生労働省健康局がん・疾病対策課. 平成30年全国がん登録 罹患数・率報告 2018 [ref. 1]
- ClinGen Actionability Reports: FLCN Adult. ver.1.2.1 (2020.5.2) [ref. 2]
- GeneReviews Japan: Birt-Hogg-Dubé症候群. ver.2018.8.22 [ref. 3]
- NCCN Guidelines®: Kidney Cancer. ver.4.2022 (2021.12.21) [ref. 4]

監修：厚労科研小杉班・Actionability Working Group-Japan

編集： MONSTAR-SCREEN-2遺伝事務局

***HNF1A*遺伝子について**

*HNF1A*遺伝子とは？

- 内分泌疾患のリスクと関連があることが示されている遺伝子です。
- *HNF1A遺伝子*の生まれつきの変化は**若年発症成人型糖尿病3型 (MODY3)** の原因となることが知られています。裏面の資料 (表) をご参照ください。
- 今回の遺伝子検査でみつかった変化が「生まれつきの遺伝子の変化」かどうかは、血液検査により確認できます。

「生まれつきの遺伝子の変化」であるかどうかを知ることはどんなことに役立ちますか？

- 発症リスクが高い症状を知り、それに対する検診を行うことで、早期発見につながる可能性があります。

家族にどんな影響がありますか？
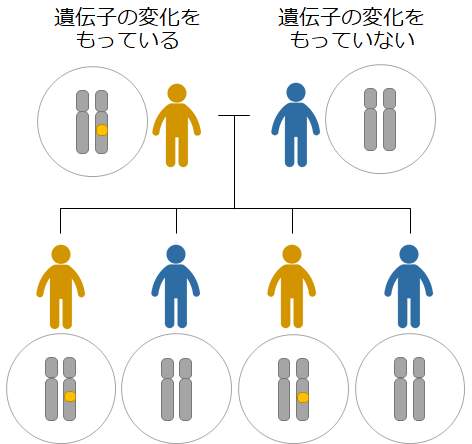


- 人は２つの*HNF1A*遺伝子をそれぞれ両親から1つずつ受け継いでいます。
- *HNF1A*遺伝子の変化を生まれつきもっていた場合、両親、兄弟、姉妹、子供が同じ変化をもっている確率は50%です。同じ変化をもっているかは、遺伝子検査で確認することができます。
- おば、おじ、いとこなども一定の確率で*同じ遺伝子の変化をもっている*可能性があります。
- 認められた遺伝子の変化についてご家族の方と情報共有することは、ご家族の方の健康管理に役立つ可能性があります。

※生まれつきの*HNF1A*遺伝子の変化は、両親いずれかから受け継いだものではなく、新規に起こった変化である可能性もあります。この場合のご家族への影響については、遺伝専門外来におたずねください。

遺伝カウンセリングについて

遺伝カウンセリングでは、生まれつきの遺伝子の変化と体質との関連、ご家族への影響などについてより詳しくご説明します。お気軽にご相談ください。　注意：遺伝学的検査の保険適用には条件があり、関連する症状を発症していないご家族の遺伝子検査や検診は自費となります。詳しくは担当者におたずね下さい。

＜参考資料表＞

|  | 一般的な日本人 | *HNF1A*遺伝子に病気の原因となる変化を生まれつきもっている | | | |
| --- | --- | --- | --- | --- | --- |
|  | 糖尿病が強く疑われる人の割合^1)^ | 発症する確率^2)^ | | 推奨される対応^3)^ | |
|  |  | 女性 | 男性 | 女性 | 男性 |
| インスリン分泌  不全型糖尿病 | 19.7 % (男性)  10.8 % (女性)  （注） | 25歳までに63%  55歳までに96% | | 一般の糖尿病と同様の治療 (食事、運動、薬物療法) と合併症の管理 | |

（注）インスリン分泌不全型以外の糖尿病も含みます。

※*HNF1A遺伝子*の変化が生まれつきの変化であっても、必ず症状がみられるというわけではありません。

※詳細については各施設の専門診療科あるいは遺伝専門外来におたずねください。

【参考文献】

- 厚生労働省. 令和元 年国民健康・栄養調査結果の概要. [ref. 1]
- GeneReviews®: Maturity-Onset Diabetes of the Young Overview. ver.2018.5.24 [ref. 2]
- Maturity-onset diabetes of the young as a model for elucidating the multifactorial origin of type 2 diabetes mellitus. J Diabetes Investig, 2018; 9(4): 704. [ref. 3]
- ClinGen Actionability Reports: HNF1A Adult. ver.1.2.1 (2020.4.27)

監修：厚労科研小杉班・Actionability Working Group-Japan

編集： MONSTAR-SCREEN-2遺伝事務局

***MAX*遺伝子について**

腫瘍*の発症と関連する遺伝子の変化について*

- 一般的に腫瘍の一部は「生まれつきの遺伝子の変化」が原因といわれています。
  - 「生まれつきの遺伝子の変化」をもっている場合、腫瘍ができやすい体質をもっていると考えられます。
  - 腫瘍ができやすい体質は親、子、兄弟、姉妹などの血のつながった家族と共有している可能性があります。
  - 体質を知ることで、その情報をご自身やご家族の健康管理に役立てることができます。
  - 今回の遺伝子検査でみつかった変化が「生まれつきの遺伝子の変化」かどうかは、血液検査により確認できます。

*MAX*遺伝子とは？

- 腫瘍を発症するリスクと関連があることが示されている遺伝子です。裏面の資料 (表) をご参照ください。

「生まれつきの遺伝子変化」であるかどうかを知ることはどんなことに役立ちますか？

- 発症リスクが高い腫瘍を知り、それに対する検診を行うことで、早期発見につながる可能性があります。

家族にどんな影響がありますか？
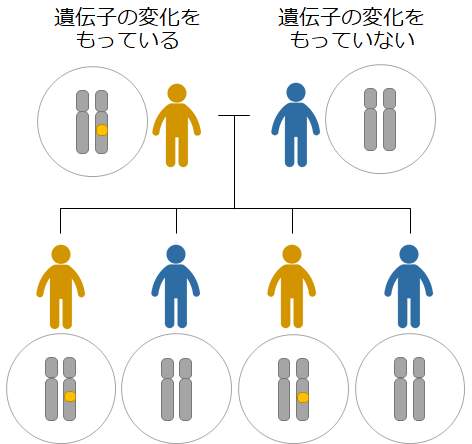


- 人は２つの*MAX*遺伝子をそれぞれ両親から1つずつ受け継いでいます。
- *MAX*遺伝子の変化を生まれつきもっていた場合、両親、兄弟、姉妹、子どもが同じ変化をもっている確率は50%です。同じ変化をもっているかは、遺伝子検査で確認することができます。
- おば、おじ、いとこなども一定の確率で*同じ遺伝子の変化をもっている*可能性があります。
- 遺伝子の変化についてご家族の方と情報共有することは、ご家族の方の健康管理に役立つ可能性があります。

※生まれつきの*MAX*遺伝子の変化は、両親いずれかから受け継いだものではなく、新規に起こった変化である可能性もあります。この場合のご家族への影響については、遺伝専門外来におたずねください。

遺伝カウンセリングについて

遺伝カウンセリングでは、生まれつきの遺伝子の変化と体質との関連、ご家族への影響などについてより詳しくご説明します。お気軽にご相談ください。 注意：遺伝学的検査の保険適用には条件があり、関連する症状を発症していないご家族の遺伝子検査や検診は自費となります。詳しくは担当者におたずね下さい。

＜参考資料表＞

|  | 一般的な日本人 | *MAX*遺伝子に病気の原因となる変化を生まれつきもっている | | | |
| --- | --- | --- | --- | --- | --- |
|  | 生涯を通して  罹患する確率^1,2,3)^ | 生涯を通して  罹患する確率 | | 推奨される対応 | |
|  |  | 女性^3)^ | 男性^3)^ | 女性^3)^ | 男性^3)^ |
| パラガングリ  オーマ | 患者数：全国で  約1500人/年  ※米国データ：100万人に2人 | リスクが上がる  可能性 | | 1年ごとの血液検査 (血中遊離メタネフリン)  2年ごとのCTまたはMRI検査  3年ごとのI-MIBGシンチグラフィ (画像検査) | |
| 副腎  褐色細胞腫 | 0.01~0.02 ％ | リスクが上がる  可能性 | |  |  |

※今回見つかった*MAX遺伝子*の変化が生まれつきの変化であっても、必ず症状がみられるというわけではありません。

※上記に示す罹患する確率は、良性・境界性の腫瘍を含めた確率であり、悪性腫瘍はその一部です。

※記載は日本の診療ガイドライン^3)^を参照しています。詳細は各施設の遺伝専門外来におたずねください。

※表は2021年時点の記載です。今後、研究が進み推奨内容が変わる可能性があります。

【参考文献】

- 国立がん研究センター希少がんセンター: パラガングリオーマ <https://www.ncc.go.jp/jp/rcc/about/paraganguriouma/index.html> [ref. 1]
- National Cancer Institute Center for Cancer Research <https://www.cancer.gov/pediatric-adult-rare-tumor/rare-tumors/rare-endocrine-tumor/paraganglioma> [ref. 2]
- 日本内分泌学会. 褐色細胞腫・パラガングリオーマ診療ガイドライン2018. (2018年7月) [ref. 3]
- GeneReviews Japan: 遺伝性パラガングリオーマ・褐色細胞腫症候群. ver.2020.7.15
- ClinGen Actionability Reports: MAX, SDHA, SDHAF2, SDHB, SDHC, SDHD, TMEM127 Adult. ver.1.1.3 (2022.1.3)

監修：厚労科研小杉班・Actionability Working Group-Japan

編集： MONSTAR-SCREEN-2遺伝事務局

***MEN1*遺伝子について**

*腫瘍と関連する遺伝子の変化について*

- 一般的に腫瘍の一部は「生まれつきの遺伝子の変化」が原因といわれています。
  - 「生まれつきの遺伝子の変化」をもっている場合、腫瘍ができやすい体質をもっていると考えられます。
  - 腫瘍ができやすい体質は親、子、兄弟、姉妹などの血のつながった家族と共有している可能性があります。
  - 体質を知ることで、その情報をご自身やご家族の健康管理に役立てることができます。
  - 今回の遺伝子検査でみつかった変化が「生まれつきの遺伝子の変化」かどうかは、血液検査により確認できます。

*MEN1*遺伝子とは？

- *MEN1遺伝子*の生まれつきの変化は**多発性内分泌腫瘍症1型**の原因となることが知られています。裏面の資料 (表) をご参照ください。

「生まれつきの遺伝子の変化」であるかどうかを知ることはどんなことに役立ちますか？

- 発症リスクが高い症状を知り、それに対する検診を行うことで、早期発見につながる可能性があります。

家族にどんな影響がありますか？
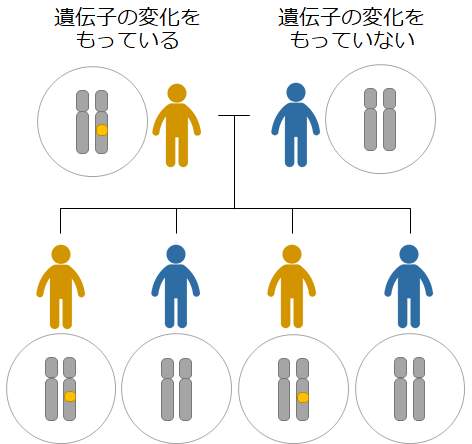


- 人は２つの*MEN1*遺伝子をそれぞれ両親から1つずつ受け継いでいます。
- *MEN1*遺伝子の変化を生まれつきもっていた場合、両親、兄弟、姉妹、子供が同じ変化をもっている確率は50%です。同じ変化をもっているかは、遺伝子検査で確認することができます。
- おば、おじ、いとこなども一定の確率で*同じ遺伝子の変化をもっている*可能性があります。
- 遺伝子の変化についてご家族の方と情報共有することは、ご家族の方の健康管理に役立つ可能性があります。

※生まれつきの*MEN1*遺伝子の変化は、両親いずれかから受け継いだものではなく、新規に起こった変化である可能性が約10%^1)^ (注意：多発性内分泌腫瘍症1型として) といわれています。この場合のご家族への影響については、遺伝専門外来におたずねください。

遺伝カウンセリングについて

遺伝カウンセリングでは、生まれつきの遺伝子の変化と腫瘍との関連、ご家族への影響などについてより詳しくご説明します。お気軽にご相談ください。　注意：遺伝学的検査の保険適用には条件があり、関連する症状を発症していないご家族の遺伝子検査や検診は自費となります。詳しくは担当者におたずね下さい。

＜参考資料表＞

|  | 一般的な日本人 | *MEN1*遺伝子に病気の原因となる変化を生まれつきもっている | | | |
| --- | --- | --- | --- | --- | --- |
|  | 生涯を通して  罹患する確率^2,3,4,5)^ | 生涯を通して  罹患する確率 | | 推奨される対応 | |
|  |  | 女性^6)^ | 男性^6)^ | 女性^6,7)^ | 男性^6,7)^ |
| 原発性副甲状腺機能亢進症 | 4~5千人あたり  1人 | 95% | | 血液検査 (血清カルシウム、インタクトPTH)、尿検査 | |
| 膵・消化管神経内分泌腫瘍 | 10万人あたり  3~5人 | 60 ％ | | 1年ごとに血液検査 (空腹時血糖、インスリン、ガストリン、グルカゴン)  2~3年ごとに腹部CTまたはMRI検査と上部消化管内視鏡検査 | |
| 下垂体腺腫 | 10万人あたり  2~3人 | 50 ％ | | 2~3年ごとに血液検査 (プロラクチン、成長ホルモン、IGF-１)  3~5年ごとに下垂体MRIまたはCT検査 | |
| 副腎皮質腫瘍 | 100万人あたり  1~2人 | 20 % | | 2~3年ごとにMRIまたはCT検査 (通常膵臓の検査時に同時に評価可能) | |

※今回見つかった*MEN1遺伝子*の変化が生まれつきの変化であっても、必ず症状がみられるというわけではありません。

※上記に示す罹患する確率は、良性・境界性の腫瘍を含めた確率であり、悪性腫瘍はその一部です。上表以外にも、皮膚、胸腺等に特徴的な症状がみられることがあります。詳細は遺伝専門外来におたずねください。

※遺伝子の変化をもっていた場合の罹患する確率、推奨される対応の記載は日本の診療ガイドブック^6)^を参照しています。詳細は各施設の遺伝専門外来におたずねください。

※表は2021年時点の記載です。今後、研究が進み推奨内容が変わる可能性があります。

【参考文献】

- GeneReviews Japan: 多発性内分泌腫瘍症1型. ver.2018.10.1 [ref. 1]
- Clinical features of multiple endocrine neoplasia type 1(MEN1) phenocopy without germline MEN1 gene mutations: analysis of 20 Japanese sporadic cases with MEN1. Clin Endocrinol (Oxf), 2000; 52(4): 509. [ref. 2]
- 日本神経内分泌腫瘍研究会. 膵・消化管神経内分泌腫瘍診療ガイドライン第２版作成委員会. 膵・消化管神経内分泌腫瘍(NEN)診療ガイドライン2019年第2版. (2019年9月) [ref. 3]
- 全国がん登録罹患数・率 報告 平成30年 <https://www.mhlw.go.jp/content/10900000/000794199.pdf> [ref. 4]
- がんの統計2021 <https://ganjoho.jp/public/qa_links/report/statistics/pdf/cancer_statistics_2021.pdf> [ref. 5]
- 多発性内分泌腫瘍症診療ガイドブック編集委員会. 多発性内分泌腫瘍症 診療ガイドブック. (2013年4月) [ref. 6]
- MEN1遺伝子の発症前診断に用いる説明文書 <http://men-net.org/medical/child.html> [ref. 7]
- ClinGen Actionability Reports: MEN1 Adult. ver.2.2.3 (2021.10.4)

監修：厚労科研小杉班・Actionability Working Group-Japan

編集： MONSTAR-SCREEN-2遺伝事務局

***MET*遺伝子について**

*がんの発症と関連する遺伝子の変化について*

- - 一般的にがんの5~10%は「生まれつきの遺伝子の変化」が原因といわれています。
  - 「生まれつきの遺伝子の変化」をもっている場合、がんになりやすい体質をもっていると考えられます。
  - がんになりやすい体質は親、子、兄弟、姉妹 などの血のつながった家族と共有している可能性があります。
  - 体質を知ることで、その情報をご自身やご家族の健康管理に役立てることができます。
  - 今回の遺伝子検査でみつかった変化が「生まれつきの遺伝子の変化」かどうかは、血液検査により確認できます。

*MET*遺伝子とは？

- *MET遺伝子*の生まれつきの変化は***遺伝性乳頭状腎細胞がん***の原因となることが知られています。裏面の資料 (表) をご参照ください。

「生まれつきの遺伝子の変化」であるかどうかを知ることはどんなことに役立ちますか？

- 発症リスクが高いがんを知り、それに対する検診を行うことで、早期発見につながる可能性があります。

家族にどんな影響がありますか？
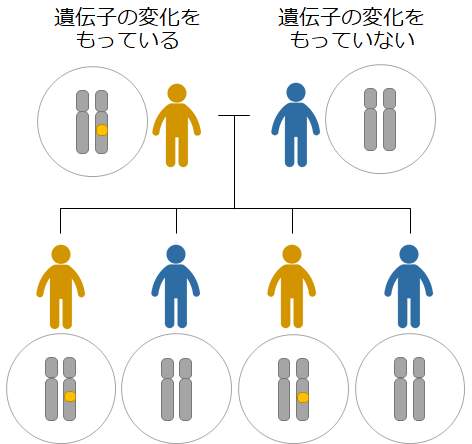


- 人は２つの*MET*遺伝子をそれぞれ両親から1つずつ受け継いでいます。
- *MET*遺伝子の変化を生まれつきもっていた場合、両親、兄弟、姉妹、子どもが同じ変化をもっている確率は50%です。同じ変化をもっているかは、遺伝子検査で確認することができます。
- おば、おじ、いとこなども一定の確率で*同じ遺伝子の変化をもっている*可能性があります。
- 遺伝子の変化についてご家族の方と情報共有することは、ご家族の方の健康管理に役立つ可能性があります。

※生まれつきの*MET*遺伝子の変化は、両親いずれかから受け継いだものではなく、新規に起こった変化である可能性もあります。この場合のご家族への影響については、遺伝専門外来におたずねください。

遺伝カウンセリングについて

遺伝カウンセリングでは、生まれつきの遺伝子の変化と体質との関連、ご家族への影響などについてより詳しくご説明します。お気軽にご相談ください。　注意：遺伝学的検査の保険適用には条件があり、関連する症状を発症していないご家族の遺伝子検査や検診は自費となります。詳しくは担当者におたずね下さい。

＜参考資料表＞

|  | 一般的な日本人 | *MET*遺伝子に病気の原因となる変化を生まれつきもっている | | | |
| --- | --- | --- | --- | --- | --- |
|  | 生涯を通して  罹患する確率^1)^ | 80歳までに  罹患する確率 | | 推奨される対応 | |
|  |  | 女性^2)^ | 男性^2)^ | 女性^3)^ | 男性^3)^ |
| 乳頭状  腎細胞癌 | (腎 (腎盂を除く))  1.2% (男性)  0.4% (女性) | ≦90 % | | 腹部MRI (推奨) またはCT (１～２年に1回)  ：30歳以降 (注) | |

(注) 記載の推奨事項については、本邦での具体的な検診方法は示されていません。詳細は各施設の遺伝専門外来におたずねください。

※*MET遺伝子*の変化が生まれつきの変化であっても、必ずがんを発症するというわけではありません。

※遺伝子の変化をもっていた場合の罹患する確率、推奨される対応の記載は米国のガイドライン (NCCNガイドライン)^3)^ を参照しています。

※表は2021年時点の記載です。今後、研究が進み推奨内容が変わる可能性があります。

【参考文献】

- 厚生労働省健康局がん・疾病対策課. 平成30年全国がん登録 罹患数・率報告 2018 [ref. 1]
- ClinGen Actionability Reports: MET Adult. ver.1.2.1 (2021.9.15) [ref. 2]
- NCCN Guidelines®: Kidney Cancer. ver.4.2022 (2021.12.21) [ref. 3]

監修：厚労科研小杉班・Actionability Working Group-Japan

編集： MONSTAR-SCREEN-2遺伝事務局

***MLH1*遺伝子について**

*がんの発症と関連する遺伝子の変化について*

- - 一般的にがんの5~10%は「生まれつきの遺伝子の変化」が原因といわれています。
  - 「生まれつきの遺伝子の変化」をもっている場合、がんになりやすい体質をもっていると考えられます。
  - がんになりやすい体質は親、子、兄弟、姉妹 などの血のつながった家族と共有している可能性があります。
  - 体質を知ることで、その情報をご自身やご家族の健康管理に役立てることができます。
  - 今回の遺伝子検査でみつかった変化が「生まれつきの遺伝子の変化」かどうかは、血液検査により確認できます。

*MLH1*遺伝子とは？

- *MLH1遺伝子*の生まれつきの変化は**リンチ症候群**の原因となることが知られています。裏面の資料(表) をご参照ください。

「生まれつきの遺伝子の変化」であるかどうかを知ることはどんなことに役立ちますか？

- 発症リスクが高いがんを知り、それに対する検診を行うことで、早期発見につながる可能性があります。

家族にどんな影響がありますか？
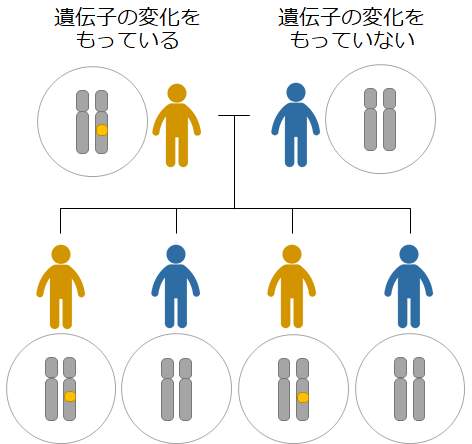


- 人は２つの*MLH1*遺伝子をそれぞれ両親から1つずつ受け継いでいます。
- *MLH1*遺伝子の変化を生まれつきもっていた場合、両親、兄弟、姉妹、子どもが同じ変化をもっている確率は50%です。同じ変化をもっているかは、遺伝子検査で確認することができます。
- おば、おじ、いとこなども一定の確率で*同じ遺伝子の変化をもっている*可能性があります。
- 遺伝子の変化についてご家族の方と情報共有することは、ご家族の方の健康管理に役立つ可能性があります。

※生まれつきの*MLH1*遺伝子の変化は、両親いずれかから受け継いだものではなく、新規に起こった変化である可能性もあります。この場合のご家族への影響については、遺伝専門外来におたずねください。

遺伝カウンセリングについて

遺伝カウンセリングでは、生まれつきの遺伝子の変化と体質との関連、ご家族への影響などについてより詳しくご説明します。お気軽にご相談ください。　注意：遺伝学的検査の保険適用には条件があり、関連する症状を発症していないご家族の遺伝子検査や検診は自費となります。詳しくは担当者におたずね下さい。

＜参考資料表＞

|  | 一般的な日本人 | *MLH1*遺伝子に病気の原因となる変化を生まれつきもっている | | | |
| --- | --- | --- | --- | --- | --- |
|  | 生涯を通して  罹患する確率^1)^ | 80歳までに  罹患する確率 | | 推奨される対応 | |
|  |  | 女性^２)^ | 男性^２)^ | 女性^３)^ | 男性^３)^ |
| 大腸がん | 10.2 ％ (男性)  8.0 ％ (女性) | 46~61 % | | 20~25歳以降：  1~2年ごとに大腸内視鏡検査 | |
| 子宮内膜がん | 2.0% (女性) | 34~54 % | ― | 30~35歳以降：1年ごとに経腟超音波検査、子宮内膜組織診 (または細胞診)、(血清マーカー検査) | |
| 卵巣がん | 1.6% (女性) | 4~20 % | ― |  |  |
| 胃がん | 10.3 ％ (男性)  4.7 ％ (女性) | 5~7 % (注) | | (胃・十二指腸がんの家族歴がある場合に考慮)  30~35歳以降：  ヘリコバクター・ピロリ菌除菌  1~3年ごとに上部消化管内視鏡検査 | |
| 腎盂・尿管がん | 2.2 % (男性)  1.1 % (女性) | 0.2~5 % | | (尿路上皮がんの家族歴がある場合に考慮)  30~35歳以降：  1年ごとに検尿 (または尿細胞診) | |
| 膀胱がん | 2.1 % (男性)  0.7 % (女性) | 2~7 % | |  |  |

(注) 記載した確率は、東アジア人では欧米諸国に比べ高いことが知られています^4)^。リンチ症候群と診断された日本人において60~70歳までに胃がんに罹患する確率は14.5~24%との報告^5,6)^があります。

※*MLH1遺伝子*の変化が生まれつきの変化であっても、必ずがんを発症するというわけではありません。

※遺伝子の変化をもっていた場合の罹患する確率、推奨される対応の記載は日本の診療ガイドライン^3)^および海外のガイドライン (米国)^2)^ を参照しています。推奨事項については、各医療機関において必ずしも実施されているとは限りませんので、詳細については各施設の専門診療科あるいは遺伝専門外来におたずねください。

※表は2021年時点の記載です。今後、研究が進み推奨内容が変わる可能性があります。

【参考文献】

- 国立がん研究センターがん情報サービス「累積がん罹患リスク (2018年データ)」 <https://ganjoho.jp/reg_stat/statistics/stat/summary.html> [ref. 1]
- NCCN Guidelines® Genetic/Familial High-Risk Assessment: Colorectal. ver.1.2021 (2021.5.11) [ref. 2]
- 大腸癌研究会. 遺伝性大腸癌診療ガイドライン 2020年版. (2020年4月) [ref. 3]
- Managing gastric cancer risk in lynch syndrome: controversies and recommendations. Fam Cancer, 2021 (online ahead of print). [ref. 4]
- Tumor development in Japanese patients with Lynch syndrome. PLoS One, 2018; 13(4): e0195572. [ref. 5]
- Comparison of clinical features between suspected familial colorectal cancer type X and Lynch syndrome in Japanese patients with colorectal cancer: a cross-sectional study conducted by the Japanese Society for Cancer of the Colon and Rectum. Jpn J Clin Oncol, 2015; 45(2): 153. [ref. 6]
- GeneReviews Japan: リンチ症候群. ver.2021.4.21
- ClinGen Actionability Reports: MLH1, MSH2, MSH6, PMS2, EPCAM Adult. ver.1.1.2 (2021.9.15)

監修：厚労科研小杉班・Actionability Working Group-Japan

編集： MONSTAR-SCREEN-2遺伝事務局

***MSH2*遺伝子について**

*がんの発症と関連する遺伝子の変化について*

- - 一般的にがんの5~10%は「生まれつきの遺伝子の変化」が原因といわれています。
  - 「生まれつきの遺伝子の変化」をもっている場合、がんになりやすい体質をもっていると考えられます。
  - がんになりやすい体質は親、子、兄弟、姉妹 などの血のつながった家族と共有している可能性があります。
  - 体質を知ることで、その情報をご自身やご家族の健康管理に役立てることができます。
  - 今回の遺伝子検査でみつかった変化が「生まれつきの遺伝子の変化」かどうかは、血液検査により確認できます。

*MSH2*遺伝子とは？

- *MSH2遺伝子*の生まれつきの変化は**リンチ症候群**の原因となることが知られています。裏面の資料 (表) をご参照ください。

「生まれつきの遺伝子の変化」であるかどうかを知ることはどんなことに役立ちますか？

- 発症リスクが高いがんを知り、それに対する検診を行うことで、早期発見につながる可能性があります。


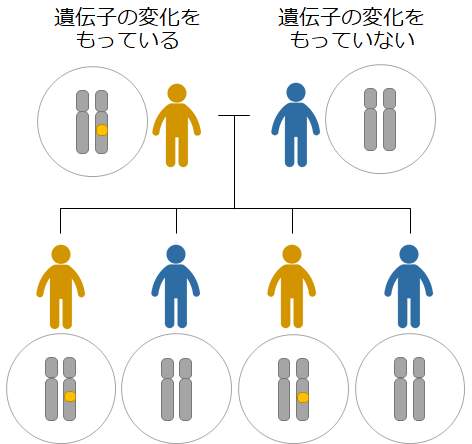
家族にどんな影響がありますか？

- 人は２つの*MSH2*遺伝子をそれぞれ両親から1つずつ受け継いでいます。
- *MSH2*遺伝子の変化を生まれつきもっていた場合、両親、兄弟、姉妹、子どもが同じ変化をもっている確率は50%です。同じ変化をもっているかは、遺伝子検査で確認することができます。
- おば、おじ、いとこなども一定の確率で*同じ遺伝子の変化をもっている*可能性があります。
- 遺伝子の変化についてご家族の方と情報共有することは、ご家族の方の健康管理に役立つ可能性があります。

※生まれつきの*MSH2*遺伝子の変化は、両親いずれかから受け継いだものではなく、新規に起こった変化である可能性もあります。この場合のご家族への影響については、遺伝専門外来におたずねください。

遺伝カウンセリングについて

遺伝カウンセリングでは、生まれつきの遺伝子の変化と体質との関連、ご家族への影響などについてより詳しくご説明します。お気軽にご相談ください。　注意：遺伝学的検査の保険適用には条件があり、関連する症状を発症していないご家族の遺伝子検査や検診は自費となります。詳しくは担当者におたずね下さい。

＜参考資料表＞

|  | 一般的な日本人 | *MSH2*遺伝子に病気の原因となる変化を生まれつきもっている | | | |
| --- | --- | --- | --- | --- | --- |
|  | 生涯を通して  罹患する確率^1)^ | 80歳までに  罹患する確率 | | 推奨される対応 | |
|  |  | 女性^2)^ | 男性^2)^ | 女性^3)^ | 男性^3)^ |
| 大腸がん | 10.2 ％ (男性)  8.0 ％ (女性) | 33~52 % | | 20~25歳以降：  1~2年ごとに大腸内視鏡検査 | |
| 子宮内膜がん | 2.0% (女性) | 21~57 % | ― | 30~35歳以降：1年ごとに経腟超音波検査、子宮内膜組織診 (または細胞診)、(血清マーカー検査) | |
| 卵巣がん | 1.6% (女性) | 8~38 % | ― |  |  |
| 胃がん | 10.3 ％ (男性)  4.7 ％ (女性) | 0.2~9 % (注) | | (胃・十二指腸がんの家族歴がある場合に考慮)  30~35歳以降：  ヘリコバクター・ピロリ菌除菌  1~3年ごとに上部消化管内視鏡検査 | |
| 腎・尿管がん | 2.2 % (男性)  1.1 % (女性) | 2.2~28 %  (男性においてリスクが  高いとされています) | | 30~35歳以降：  1年ごとに検尿 (または尿細胞診) | |
| 膀胱がん | 2.1 % (男性)  0.7 % (女性) | 4.4~12.8 % | |  |  |

(注) 記載した確率は、東アジア人では欧米諸国に比べ高いことが知られています^4)^。リンチ症候群と診断された日本人において60~70歳までに胃がんに罹患する確率は14.5~24%との報告^5,6)^があります。

※*MSH2遺伝子*の変化が生まれつきの変化であっても、必ずがんを発症するというわけではありません。

※遺伝子の変化をもっていた場合の罹患する確率、推奨される対応の記載は日本の診療ガイドライン^3)^および海外のガイドライン (米国)^2)^ を参照しています。推奨事項については、各医療機関において必ずしも実施されているとは限りませんので、詳細については各施設の専門診療科あるいは遺伝専門外来におたずねください。

※表は2021年時点の記載です。今後、研究が進み推奨内容が変わる可能性があります。

【参考文献】

- 国立がん研究センターがん情報サービス「累積がん罹患リスク (2018年データ)」 <https://ganjoho.jp/reg_stat/statistics/stat/summary.html> [ref. 1]
- NCCN Guidelines® Genetic/Familial High-Risk Assessment: Colorectal. ver.1.2021 (2021.5.11) [ref. 2]
- 大腸癌研究会. 遺伝性大腸癌診療ガイドライン 2020年版. (2020年4月) [ref. 3]
- Managing gastric cancer risk in lynch syndrome: controversies and recommendations. Fam Cancer, 2021 (online ahead of print). [ref. 4]
- Tumor development in Japanese patients with Lynch syndrome. PLoS One, 2018; 13(4): e0195572. [ref. 5]
- Comparison of clinical features between suspected familial colorectal cancer type X and Lynch syndrome in Japanese patients with colorectal cancer: a cross-sectional study conducted by the Japanese Society for Cancer of the Colon and Rectum. Jpn J Clin Oncol, 2015; 45(2): 153. [ref. 6]
- GeneReviews Japan: リンチ症候群. ver.2021.4.21
- ClinGen Actionability Reports: MLH1, MSH2, MSH6, PMS2, EPCAM Adult. ver.1.1.2 (2021.9.15)

監修：厚労科研小杉班・Actionability Working Group-Japan

編集： MONSTAR-SCREEN-2遺伝事務局

***MSH6*遺伝子について**

*がんの発症と関連する遺伝子の変化について*

- - 一般的にがんの5~10%は「生まれつきの遺伝子の変化」が原因といわれています。
  - 「生まれつきの遺伝子の変化」をもっている場合、がんになりやすい体質をもっていると考えられます。
  - がんになりやすい体質は親、子、兄弟、姉妹 などの血のつながった家族と共有している可能性があります。
  - 体質を知ることで、その情報をご自身やご家族の健康管理に役立てることができます。
  - 今回の遺伝子検査でみつかった変化が「生まれつきの遺伝子の変化」かどうかは、血液検査により確認できます。

*MSH6*遺伝子とは？

- *MSH6遺伝子*の生まれつきの変化は**リンチ症候群**の原因となることが知られています。裏面の資料 (表) をご参照ください。

「生まれつきの遺伝子の変化」であるかどうかを知ることはどんなことに役立ちますか？

- 発症リスクが高いがんを知り、それに対する検診を行うことで、早期発見につながる可能性があります。

家族にどんな影響がありますか？
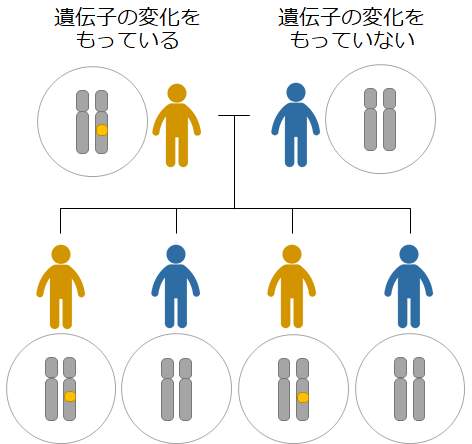


- 人は２つの*MSH6*遺伝子をそれぞれ両親から1つずつ受け継いでいます。
- *MSH6*遺伝子の変化を生まれつきもっていた場合、両親、兄弟、姉妹、子どもが同じ変化をもっている確率は50%です。同じ変化をもっているかは、遺伝子検査で確認することができます。
- おば、おじ、いとこなども一定の確率で*同じ遺伝子の変化をもっている*可能性があります。
- 遺伝子の変化についてご家族の方と情報共有することは、ご家族の方の健康管理に役立つ可能性があります。

※生まれつきの*MSH6*遺伝子の変化は、両親いずれかから受け継いだものではなく、新規に起こった変化である可能性もあります。この場合のご家族への影響については、遺伝専門外来におたずねください。

遺伝カウンセリングについて

遺伝カウンセリングでは、生まれつきの遺伝子の変化と体質との関連、ご家族への影響などについてより詳しくご説明します。お気軽にご相談ください。　注意：遺伝学的検査の保険適用には条件があり、関連する症状を発症していないご家族の遺伝子検査や検診は自費となります。詳しくは担当者におたずね下さい。

＜参考資料表＞

|  | 一般的な日本人 | *MSH6*遺伝子に病気の原因となる変化を生まれつきもっている | | | |
| --- | --- | --- | --- | --- | --- |
|  | 生涯を通して  罹患する確率^1)^ | 80歳までに  罹患する確率 | | 推奨される対応 | |
|  |  | 女性^2)^ | 男性^2)^ | 女性^3)^ | 男性^3)^ |
| 大腸がん | 10.2 ％ (男性)  8.0 ％ (女性) | 10~44 % | | 20~25歳以降：  1~2年ごとに大腸内視鏡検査 | |
| 子宮内膜がん | 2.0% (女性) | 16~49 % | ― | 30~35歳以降：1年ごとに経腟超音波検査、子宮内膜組織診 (または細胞診)、(血清マーカー検査) | |
| 卵巣がん | 1.6% (女性) | ≤1~13 % | ― |  |  |
| 胃がん | 10.3 ％ (男性)  4.7 ％ (女性) | ≤1~7.9 % (注) | | (胃・十二指腸がんの家族歴がある場合に考慮)  30~35歳以降：  ヘリコバクター・ピロリ菌除菌  1~3年ごとに上部消化管内視鏡検査 | |
| 腎盂・尿管がん | 2.2 % (男性)  1.1 % (女性) | 0.7~5.5 % | | (尿路上皮がんの家族歴がある場合に考慮)  30~35歳以降：  1年ごとに検尿 (または尿細胞診) | |
| 膀胱がん | 2.1 % (男性)  0.7 % (女性) | 1.0~8.2 % | |  |  |

(注) 記載した確率は、東アジア人では欧米諸国に比べ高いことが知られています^4)^。リンチ症候群と診断された日本人において60~70歳までに胃がんに罹患する確率は14.5~24%との報告^5,6)^があります。

※*MSH6遺伝子*の変化が生まれつきの変化であっても、必ずがんを発症するというわけではありません。

※遺伝子の変化をもっていた場合の罹患する確率、推奨される対応の記載は日本の診療ガイドライン^3)^および海外のガイドライン (米国)^2)^ を参照しています。推奨事項については、各医療機関において必ずしも実施されているとは限りませんので、詳細については各施設の専門診療科あるいは遺伝専門外来におたずねください。

※表は2021年時点の記載です。今後、研究が進み推奨内容が変わる可能性があります。

【参考文献】

- 国立がん研究センターがん情報サービス「累積がん罹患リスク (2018年データ)」 <https://ganjoho.jp/reg_stat/statistics/stat/summary.html> [ref. 1]
- NCCN Guidelines® Genetic/Familial High-Risk Assessment: Colorectal. ver.1.2021 (2021.5.11) [ref. 2]
- 大腸癌研究会. 遺伝性大腸癌診療ガイドライン 2020年版. (2020年4月) [ref. 3]
- Managing gastric cancer risk in lynch syndrome: controversies and recommendations. Fam Cancer, 2021 (online ahead of print). [ref. 4]
- Tumor development in Japanese patients with Lynch syndrome. PLoS One, 2018; 13(4): e0195572. [ref. 5]
- Comparison of clinical features between suspected familial colorectal cancer type X and Lynch syndrome in Japanese patients with colorectal cancer: a cross-sectional study conducted by the Japanese Society for Cancer of the Colon and Rectum. Jpn J Clin Oncol, 2015; 45(2): 153. [ref. 6]
- GeneReviews Japan: リンチ症候群. ver.2021.4.21
- ClinGen Actionability Reports: MLH1, MSH2, MSH6, PMS2, EPCAM Adult. ver.1.1.2 (2021.9.15)

監修：厚労科研小杉班・Actionability Working Group-Japan

編集： MONSTAR-SCREEN-2遺伝事務局

***MUTYH*遺伝子について**

**※本資料は、 *MUTYH*の遺伝子変化が両アレルに検出された場合に使用する資料になります。**

*がんの発症と関連する遺伝子の変化について*

- - 一般的にがんの5~10%は「生まれつきの遺伝子の変化」が原因といわれています。
  - 「生まれつきの遺伝子の変化」をもっている場合、がんになりやすい体質をもっていると考えられます。
  - がんになりやすい体質は親、子、兄弟、姉妹 などの血のつながった家族と共有している可能性があります。
  - 体質を知ることで、その情報をご自身やご家族の健康管理に役立てることができます。
  - 今回の遺伝子検査でみつかった変化が「生まれつきの遺伝子の変化」かどうかは、血液検査により確認できます。

*MUTYH*遺伝子とは？

- 人は*MUTYH*遺伝子を2つもっています。２つの*MUTYH*遺伝子の両方に病気の原因となる変化を生まれつきもっていると、裏面の表に示した特定のがんを発症する確率が高くなります。
- *MUTYH遺伝子*の生まれつきの変化は***MUTYH*関連ポリポーシス**の原因となることが知られています。裏面の資料 (表) をご参照ください。

「生まれつきの遺伝子の変化」であるかどうかを知ることはどんなことに役立ちますか？

- 発症リスクが高いがんを知り、それに対する検診を行うことで、早期発見につながる可能性があります。

家族にどんな影響がありますか？

- 人は２つの*MUTYH*遺伝子をそれぞれ両親から1つずつ受け継いでいます。
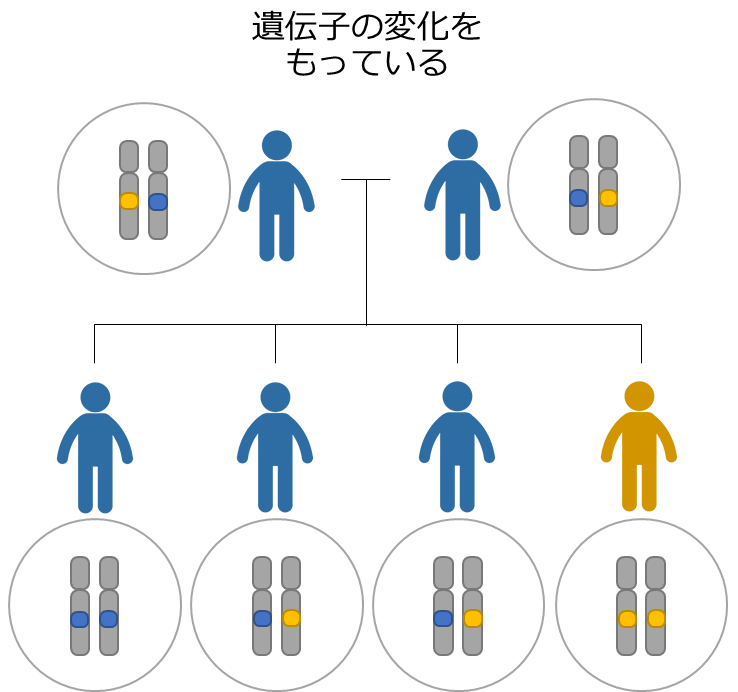

- *MUTYH*遺伝子の両方に変化を生まれつきもっていた場合、両親は変化のある遺伝子をそれぞれ１つずつもっていると考えられます。
- 兄弟、姉妹、子ども、おば、おじ、いとこなども一定の確率で*同じ遺伝子の変化をもっている*可能性がありますが、２つの*MUTYH*遺伝子のうち１つだけに変化をもっていても、健康への影響はないと考えられています。
- 遺伝子の変化についてご家族の方と情報共有することは、ご家族の方の健康管理に役立つ可能性があります。

※生まれつきの*MUTYH*遺伝子の変化は、両親から受け継いだものではなく、新規に起こった変化である可能性もあります。この場合のご家族への影響については、遺伝専門外来におたずねください。

遺伝カウンセリングについて

遺伝カウンセリングでは、生まれつきの遺伝子の変化と体質との関連、ご家族への影響などについてより詳しくご説明します。お気軽にご相談ください。　注意：遺伝学的検査の保険適用には条件があり、関連する症状を発症していないご家族の遺伝子検査や検診は自費となります。詳しくは担当者におたずね下さい。

＜参考資料表＞

|  | 一般的な日本人 | *2つのMUTYH*遺伝子に病気の原因となる変化を生まれつきもっている | | | |
| --- | --- | --- | --- | --- | --- |
|  | 生涯を通して  罹患する確率^1,2)^ | 60歳までに  罹患する確率 | | 推奨される対応 | |
|  |  | 女性^3)^ | 男性^3)^ | 女性^4,5)^ | 男性^4,5)^ |
| 大腸ポリポーシス  (がん化するリスクあり) | (大腸がん)  10.2 ％ (男性)  8.0 ％ (女性) | (大腸がん)  43~100 % | | 25~30歳以降大腸内視鏡検査：  1~3年ごとにポリープの状態に応じて | |
| 十二指腸がん | （小腸がん）  0.2 % (男性)  0.1 % (女性) | リスクが高まる可能性 | | 30~35歳以降：  上部消化管内視鏡検査 (年１回) | |

※*MUTYH遺伝子*の変化が生まれつきの変化であっても、必ずがんを発症するというわけではありません。

※遺伝子の変化をもっていた場合の罹患する確率、推奨される対応の記載は日本の診療ガイドライン^3)^および海外のガイドライン (米国、欧州)^4,5)^ を参照しています。推奨事項については、日本で必ずしも実施されているとは限りませんので、詳細は各施設の遺伝専門外来におたずねください。

※表は2021年時点の記載です。今後、研究が進み推奨内容が変わる可能性があります。

【参考文献】

- 国立がん研究センターがん情報サービス「累積がん罹患リスク (2018年データ)」 <https://ganjoho.jp/reg_stat/statistics/stat/summary.html> [ref. 1]
- 厚生労働省健康局がん・疾病対策課. 平成30年全国がん登録 罹患数・率報告 2018 [ref. 2]
- 大腸癌研究会. 遺伝性大腸癌診療ガイドライン 2020年版. (2020年4月) [ref. 3]
- NCCN Guidelines® Genetic/Familial High-Risk Assessment: Colorectal. ver.1.2021 (2021.5.11) [ref. 4]
- Revised guidelines for the clinical management of Lynch syndrome (HNPCC): recommendations by a group of European experts. Gut, 2013; 62(6): 812. [ref. 5]
- GeneReviews®: *MUTYH* Polyposis. ver.2021.5.27.
- ClinGen Actionability Reports: MUTYH Adult. ver.2.2.2 (2022.1.3)

監修：厚労科研小杉班・Actionability Working Group-Japan

編集： MONSTAR-SCREEN-2遺伝事務局

***NBN*遺伝子について**

**この遺伝子に、「生まれつきの遺伝子の変化」が見つかった場合には、その病的意義の解釈について症例ごとに検討する必要があります。まずは研究事務局にご相談ください。**

*がんの発症と関連する遺伝子の変化について*

- - 一般的にがんの5~10%は「生まれつきの遺伝子の変化」が原因といわれています。
  - 「生まれつきの遺伝子の変化」をもっている場合、がんになりやすい体質をもっていると考えられます。
  - がんになりやすい体質は親、子、兄弟、姉妹 などの血のつながった家族と共有している可能性があります。
  - 体質を知ることで、その情報をご自身やご家族の健康管理に役立てることができます。
  - 今回の遺伝子検査でみつかった変化が「生まれつきの遺伝子の変化」かどうかは、血液検査により確認できます。

*NBN*遺伝子とは？

- *がんを発症するリスクと関連することが示されている遺伝子です。裏面の資料* (*表*) *をご参照ください*。

「生まれつきの遺伝子変化」であるかどうかを知ることはどんなことに役立ちますか？

- 発症リスクが高いがんを知り、それに対する検診を行うことで、早期発見につながる可能性があります。


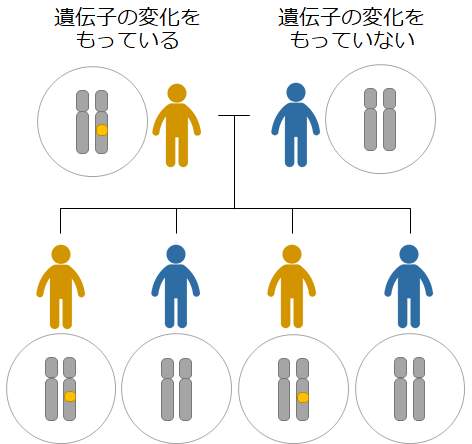
家族にどんな影響がありますか？

- 人は２つの*NBN*遺伝子をそれぞれ両親から1つずつ受け継いでいます。
- *NBN*遺伝子の変化を生まれつきもっていた場合、両親、兄弟、姉妹、子どもが同じ変化をもっている確率は50%です。同じ変化をもっているかは、遺伝子検査で確認することができます。
- おば、おじ、いとこなども一定の確率で*同じ遺伝子の変化をもっている*可能性があります。
- 遺伝子の変化についてご家族の方と情報共有することは、ご家族の方の健康管理に役立つ可能性があります。

※生まれつきの*NBN*遺伝子の変化は、両親いずれかから受け継いだものではなく、新規に起こった変化である可能性もあります。この場合のご家族への影響については、遺伝専門外来におたずねください。

遺伝カウンセリングについて

遺伝カウンセリングでは、生まれつきの遺伝子の変化と体質との関連、ご家族への影響などについてより詳しくご説明します。お気軽にご相談ください。 注意：遺伝学的検査の保険適用には条件があり、関連する症状を発症していないご家族の遺伝子検査や検診は自費となります。詳しくは担当者におたずね下さい。

＜参考資料表＞

|  | 一般的な日本人 | *NBN遺伝子*に病気の原因となる変化を生まれつきもっている | | | |
| --- | --- | --- | --- | --- | --- |
|  | 生涯を通して  罹患する確率^1)^ | 生涯を通して  罹患する確率 | | 推奨される対応 | |
|  |  | 女性^2,3)^ | 男性 | 女性^2)^ | 男性 |
| 卵巣がん | 1.6 % (女性) | リスクが上がる  可能性 | ― | ※家族歴や既往歴によってリスクは異なると考えられています。現時点では検診方法は確立されていません。 | ― |
| 乳がん | 10.9 ％ (女性) | リスクが上がる  可能性 | ― |  | ― |

※*NBN遺伝子*の変化が生まれつきの変化であっても、必ずがんを発症するというわけではありません。

※上表は*NBN*遺伝子の一部の種類の変化におけるデータです。遺伝子の変化の種類に応じて検診の方法や頻度を検討することが推奨されています。

※検診等の推奨について、現状では一般的ながん検診を積極的に受検することが推奨されます。詳細は各施設の遺伝専門外来におたずねください。

※表は2021年時点の記載です。今後、研究が進み推奨内容が変わる可能性があります。

【参考文献】

- 国立がん研究センターがん情報サービス「累積がん罹患リスク (2018年データ)」 https://ganjoho.jp/reg_stat/statistics/stat/summary.html [ref. 1]
- NCCN Guidelines® Genetic/Familial High-Risk Assessment: Breast, Ovarian, and Pancreatic. ver.1.2022 (2021.8.11) [ref. 2]
- GeneReviews®: Nijmegen Breakage Syndrome. ver.2017.2.2 [ref. 3]

監修：厚労科研小杉班・Actionability Working Group-Japan

編集： MONSTAR-SCREEN-2遺伝事務局

***NF1*遺伝子について**

*がんの発症と関連する遺伝子の変化について*

- - 一般的にがんの5~10%は「生まれつきの遺伝子の変化」が原因といわれています。
  - 「生まれつきの遺伝子の変化」をもっている場合、がんになりやすい体質をもっていると考えられます。
  - がんになりやすい体質は親、子、兄弟、姉妹 などの血のつながった家族と共有している可能性があります。
  - 体質を知ることで、その情報をご自身やご家族の健康管理に役立てることができます。
  - 今回の遺伝子検査でみつかった変化が「生まれつきの遺伝子の変化」かどうかは、血液検査により確認できます。

*NF1*遺伝子とは？

- *NF1遺伝子*の生まれつきの変化は**神経線維腫症１型 (レックリングハウゼン病)** の原因となることが知られています。裏面の資料 (表) をご参照ください。

「生まれつきの遺伝子変化」であるかどうかを知ることはどんなことに役立ちますか？

- 発症リスクが高いがんを知り、それに対する検診を行うことで、早期発見につながる可能性があります。

家族にどんな影響がありますか？

-
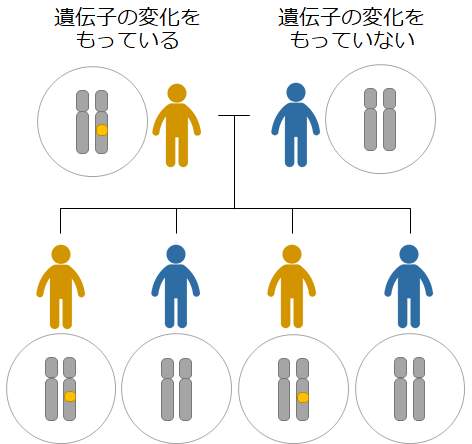
人は２つの*NF1*遺伝子をそれぞれ両親から1つずつ受け継いでいます。
- *NF1*遺伝子の変化を生まれつきもっていた場合、両親、兄弟、姉妹、子どもが同じ変化をもっている確率は50%です。同じ変化をもっているかは、遺伝子検査で確認することができます。
- おば、おじ、いとこなども一定の確率で*同じ遺伝子の変化をもっている*可能性があります。
- 遺伝子の変化についてご家族の方と情報共有することは、ご家族の方の健康管理に役立つ可能性があります。

※生まれつきの*NF1*遺伝子の変化は、両親いずれかから受け継いだものではなく、新規に起こった変化である可能性が約50%^1)^ (ただし神経線維腫症１型として) といわれています。この場合のご家族への影響については、遺伝専門外来におたずねください。

遺伝カウンセリングについて

遺伝カウンセリングでは、生まれつきの遺伝子の変化と体質との関連、ご家族への影響などについてより詳しくご説明します。お気軽にご相談ください。　注意：遺伝学的検査の保険適用には条件があり、関連する症状を発症していないご家族の遺伝子検査や検診は自費となります。詳しくは担当者におたずね下さい。

＜参考資料表＞

|  | 一般的な  日本人 | *NF1遺伝子*に病気の原因となる変化を生まれつきもっている | | | | |
| --- | --- | --- | --- | --- | --- | --- |
|  | 生涯を通して  罹患する確率^2)^ | | 生涯を通して  罹患する確率 | | 推奨される対応 | |
|  |  |  | 女性^3,4,5)^ | 男性^3,4,5)^ | 女性^3,4)^ | 男性^3,4)^ |
| 乳がん | 10.9 ％ | | 15~40 ％ | ― | 30歳以降:年1回のマンモグラフィ  30～50歳以降: 年1回の乳房造影MRI検査 | ― |
| 消化管間質腫瘍 (GIST) | データなし  ※希少がん | | 5～25 % | | 下血や腹痛などの症状が生じた場合、医療機関に相談する | |
| 悪性末梢神経鞘腫 | データなし  ※希少がん | | 2 % | | 急速な腫瘍の増大、突然の神経症状が出現した場合、医療機関に相談する | |

- 上表以外にも、皮膚、神経、眼などに特徴的な症状がみられることがあります。詳細は専門診療科あるいは遺伝専門外来におたずねください。

※*NF1*の変化が生まれつきの変化であっても、必ずがんを発症するというわけではありません。

※遺伝子の変化をもっていた場合の罹患する確率、推奨される対応の記載は日本の診療ガイドライン^3)^および米国のガイドライン (NCCNガイドライン)^4)^ を参照しています。推奨事項については、日本で必ずしも実施されているとは限りませんので、詳細は各施設の専門診療科あるいは遺伝専門外来におたずねください。

※表は2021年時点の記載です。今後、研究が進み推奨内容が変わる可能性があります。

【参考文献】

- GeneReviews Japan: 神経線維腫症1型. ver.2021.1.12 [ref. 1]
- 国立がん研究センターがん情報サービス「累積がん罹患リスク (2018年データ)」https://ganjoho.jp/reg_stat/statistics/stat/summary.html [ref. 2]
- 日本皮膚科学会. 神経線維腫症1型 (レックリングハウゼン病) 診療ガイドライン 2018. 日皮会誌, 2018; 128(1): 17. [ref. 3]
- NCCN Guidelines® Genetic/Familial High-Risk Assessment: Breast, Ovarian, and Pancreatic. ver.1.2022 (2021.8.11) [ref. 4]
- Therapeutic consequences from molecular biology for gastrointestinal stromal tumor patients affected by neurofibromatosis type 1. Clin Cancer Res, 2008; 14(14): 4550. [ref. 5]
- ClinGen Actionability Reports: NF1 Adult. ver.1.2.1 (2020.8.19)

監修：厚労科研小杉班・Actionability Working Group-Japan

編集： MONSTAR-SCREEN-2遺伝事務局

***NF2*遺伝子について**

*腫瘍と関連する遺伝子の変化について*

- 一般的に腫瘍の一部は「生まれつきの遺伝子の変化」が原因といわれています。
  - 「生まれつきの遺伝子の変化」をもっている場合、腫瘍ができやすい体質をもっていると考えられます。
  - 腫瘍ができやすい体質は親、子、兄弟、姉妹などの血のつながった家族と共有している可能性があります。
  - 体質を知ることで、その情報をご自身やご家族の健康管理に役立てることができます。
  - 今回の遺伝子検査でみつかった変化が「生まれつきの遺伝子の変化」かどうかは、血液検査により確認できます。

*NF2*遺伝子とは？

- *NF2遺伝子*の生まれつきの変化は**神経線維腫症2型**の原因となることが知られています。裏面の資料 (表) をご参照ください。

「生まれつきの遺伝子の変化」であるかどうかを知ることはどんなことに役立ちますか？

- 発症リスクが高い症状を知り、それに対する検診を行うことで、早期発見につながる可能性があります。

家族にどんな影響がありますか？
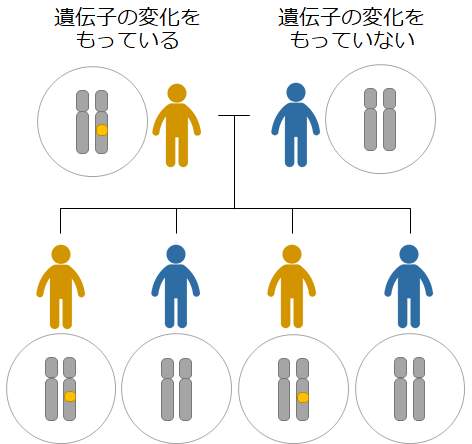


- 人は２つの*NF2*遺伝子をそれぞれ両親から1つずつ受け継いでいます。
- *NF2*遺伝子の変化を生まれつきもっていた場合、両親、兄弟、姉妹、子供が同じ変化をもっている確率は50%です。同じ変化をもっているかは、遺伝子検査で確認することができます。
- おば、おじ、いとこなども一定の確率で*同じ遺伝子の変化をもっている*可能性があります。
- 遺伝子の変化についてご家族の方と情報共有することは、ご家族の方の健康管理に役立つ可能性があります。

※生まれつきの*NF2*遺伝子の変化は、両親いずれかから受け継いだものではなく、新規に起こった変化である可能性が約50%^1,2)^ (ただし神経線維腫症2型として) といわれています。この場合のご家族への影響については、遺伝専門外来におたずねください。

遺伝カウンセリングについて

遺伝カウンセリングでは、生まれつきの遺伝子の変化と体質との関連、ご家族への影響などについてより詳しくご説明します。お気軽にご相談ください。　注意：遺伝学的検査の保険適用には条件があり、関連する症状を発症していないご家族の遺伝子検査や検診は自費となります。詳しくは担当者におたずね下さい。

＜参考資料表＞

|  | 一般的な日本人 | *NF2*遺伝子に病気の原因となる変化を生まれつきもっている | | | |
| --- | --- | --- | --- | --- | --- |
|  | 生涯を通して  罹患する確率 | 生涯を通して  罹患する確率 | | 推奨される対応 | |
|  |  | 女性^2,3)^ | 男性^2,3)^ | 女性^1,2)^ | 男性^1,2)^ |
| 前庭神経鞘腫  (聴神経鞘腫) | データなし | ほぼ100 % | | 10～12歳頃から40歳まで1年ごとのMRI検査、聴力検査 | |
| 脊髄腫瘍 | データなし | 約33 % | |  |  |
| 髄膜腫 | データなし | 50~80 % | |  |  |

- 上表以外にも、脳、神経、眼、皮膚などに症状がみられることがあります。詳細は専門診療科あるいは遺伝専門外来におたずねください。

※今回見つかった*NF2遺伝子*の変化が生まれつきの変化であっても、必ず症状がみられるというわけではありません。

※遺伝子の変化をもっていた場合の罹患する確率、推奨される対応は、海外の資料を参照しています。日本で必ずしも実施されているとは限りませんので、詳細については各施設の専門診療科あるいは遺伝専門外来におたずねください。

【参考文献】

- 厚生労働科学研究費補助金（難治性疾患政策研究事業）「神経皮膚症候群に関する診療科横断的な診療体制の確立」研究班. 神経線維腫症２型 (NF2) 治療指針 (2016年10月)　[ref. 1]
- GeneReviews®: Neurofibromatosis 2. ver.2018.3.15 [ref. 2]
- ClinGen Actionability Reports: NF2 Adult. ver.2.0.2 (2021.5.5) [ref. 3]

監修：厚労科研小杉班・Actionability Working Group-Japan

編集： MONSTAR-SCREEN-2遺伝事務局

***PALB2*遺伝子について**

*がんの発症と関連する遺伝子の変化について*

- - 一般的にがんの5~10%は「生まれつきの遺伝子の変化」が原因といわれています。
  - 「生まれつきの遺伝子の変化」をもっている場合、がんになりやすい体質をもっていると考えられます。
  - がんになりやすい体質は親、子、兄弟、姉妹 などの血のつながった家族と共有している可能性があります。
  - 体質を知ることで、その情報をご自身やご家族の健康管理に役立てることができます。
  - 今回の遺伝子検査でみつかった変化が「生まれつきの遺伝子の変化」かどうかは、血液検査により確認できます。

*PALB2*遺伝子とは？

- *がんを発症するリスクと関連があることが示されている遺伝子です。裏面の資料* (表) *をご参照ください。*

「生まれつきの遺伝子変化」であるかどうかを知ることはどんなことに役立ちますか？

- 発症リスクが高いがんを知り、それに対する検診を行うことで、早期発見につながる可能性があります。

家族にどんな影響がありますか？
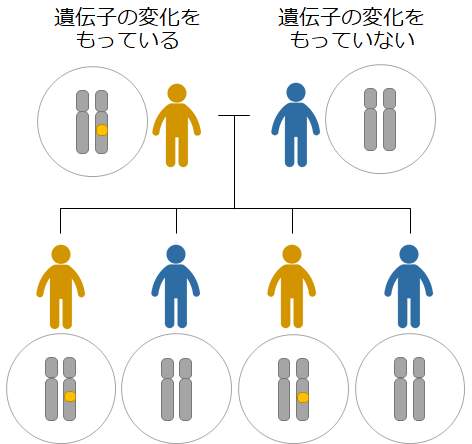


- 人は２つの*PALB2*遺伝子をそれぞれ両親から1つずつ受け継いでいます。
- *PALB2*遺伝子の変化を生まれつきもっていた場合、両親、兄弟、姉妹、子どもが同じ変化をもっている確率は50%です。同じ変化をもっているかは、遺伝子検査で確認することができます。
- おば、おじ、いとこなども一定の確率で*同じ遺伝子の変化をもっている*可能性があります。
- 遺伝子の変化についてご家族の方と情報共有することは、ご家族の方の健康管理に役立つ可能性があります。

※生まれつきの*PALB2*遺伝子の変化は、両親いずれかから受け継いだものではなく、新規に起こった変化である可能性もあります。この場合のご家族への影響については、遺伝専門外来におたずねください。

遺伝カウンセリングについて

遺伝カウンセリングでは、生まれつきの遺伝子の変化と体質との関連、ご家族への影響などについてより詳しくご説明します。お気軽にご相談ください。 注意：遺伝学的検査の保険適用には条件があり、関連する症状を発症していないご家族の遺伝子検査や検診は自費となります。詳しくは担当者におたずね下さい。

＜参考資料表＞

|  | 一般的な日本人 | *PALB2遺伝子*に病気の原因となる変化を生まれつきもっている | | | |
| --- | --- | --- | --- | --- | --- |
|  | 生涯を通して  罹患する確率^1)^ | 生涯を通して  罹患する確率 | | 推奨される対応 | |
|  |  | 女性^2)^ | 男性^2)^ | 女性^2)^ | 男性^2)^ |
| 乳がん | 10.9 ％ (女性) | 41～60 ％ | ― | 30歳以降: 年1回のマンモグラフィと乳房造影MRI検査。 (注) | ― |
| 卵巣がん | 1.6 % (女性) | 3～5 % | ― | ※現時点では検診方法は確立されていません。家族歴や既往歴によってリスクは異なると考えられています。 | ― |
| 膵がん | 2.6 % (男女) | 5～10 % | | ※現時点では検診方法は確立されていません。 | |

(注) 記載の推奨事項については、本邦での具体的な検診方法は示されていません。詳細は各施設の遺伝専門外来におたずねください。

※*PALB2遺伝子*の変化が生まれつきの変化であっても、必ずがんを発症するというわけではありません。

※遺伝子の変化をもっていた場合の罹患する確率、推奨される対応の記載 (注) は米国のガイドライン(NCCNガイドライン)^2)^ を参照しています。

※表は2021年時点の記載です。今後、研究が進み推奨内容が変わる可能性があります。

【参考文献】

- 国立がん研究センターがん情報サービス「累積がん罹患リスク (2018年データ)」 https://ganjoho.jp/reg_stat/statistics/stat/summary.html [ref. 1]
- NCCN Guidelines® Genetic/Familial High-Risk Assessment: Breast, Ovarian, and Pancreatic. ver.1.2022 (2021.8.11) [ref. 2]
- ClinGen Actionability Reports: PALB2 Adult. ver.2.2.1 (2021.9.15)

監修：厚労科研小杉班・Actionability Working Group-Japan

編集： MONSTAR-SCREEN-2遺伝事務局

***PMS2*遺伝子について**

*がんの発症と関連する遺伝子の変化について*

- - 一般的にがんの5~10%は「生まれつきの遺伝子の変化」が原因といわれています。
  - 「生まれつきの遺伝子の変化」をもっている場合、がんになりやすい体質をもっていると考えられます。
  - がんになりやすい体質は親、子、兄弟、姉妹 などの血のつながった家族と共有している可能性があります。
  - 体質を知ることで、その情報をご自身やご家族の健康管理に役立てることができます。
  - 今回の遺伝子検査でみつかった変化が「生まれつきの遺伝子の変化」かどうかは、血液検査により確認できます。

*PMS2*遺伝子とは？

- *PMS2遺伝子*の生まれつきの変化は**リンチ症候群**の原因となることが知られています。裏面の資料 (表) をご参照ください。

「生まれつきの遺伝子の変化」であるかどうかを知ることはどんなことに役立ちますか？

- 発症リスクが高いがんを知り、それに対する検診を行うことで、早期発見につながる可能性があります。


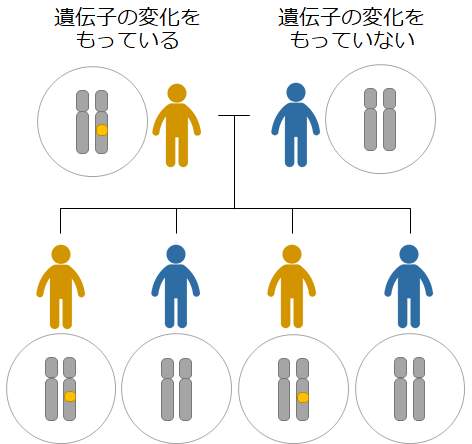
家族にどんな影響がありますか？

- 人は２つの*PMS2*遺伝子をそれぞれ両親から1つずつ受け継いでいます。
- *PMS2*遺伝子の変化を生まれつきもっていた場合、両親、兄弟、姉妹、子どもが同じ変化をもっている確率は50%です。同じ変化をもっているかは、遺伝子検査で確認することができます。
- おば、おじ、いとこなども一定の確率で*同じ遺伝子の変化をもっている*可能性があります。
- 遺伝子の変化についてご家族の方と情報共有することは、ご家族の方の健康管理に役立つ可能性があります。

※生まれつきの*PMS2*遺伝子の変化は、両親いずれかから受け継いだものではなく、新規に起こった変化である可能性もあります。この場合のご家族への影響については、遺伝専門外来におたずねください。

遺伝カウンセリングについて

遺伝カウンセリングでは、生まれつきの遺伝子の変化と体質との関連、ご家族への影響などについてより詳しくご説明します。お気軽にご相談ください。　注意：遺伝学的検査の保険適用には条件があり、関連する症状を発症していないご家族の遺伝子検査や検診は自費となります。詳しくは担当者におたずね下さい。

＜参考資料表＞

|  | 一般的な日本人 | *PMS2*遺伝子に病気の原因となる変化を生まれつきもっている | | | |
| --- | --- | --- | --- | --- | --- |
|  | 生涯を通して  罹患する確率^1)^ | 80歳までに  罹患する確率 | | 推奨される対応 | |
|  |  | 女性^2)^ | 男性^2)^ | 女性^3)^ | 男性^3)^ |
| 大腸がん | 10.2 ％ (男性)  8.0 ％ (女性) | 8.7~20 % | | 20~25歳以降：  1~2年ごとに大腸内視鏡検査 | |
| 子宮内膜がん | 2.0% (女性) | 13~26 % | ― | 30~35歳以降：1年ごとに経腟超音波検査、子宮内膜組織診（または細胞診）、（血清マーカー検査） | |
| 卵巣がん | 1.6% (女性) | 1.3~3 % | ― |  |  |
| 胃がん | 10.3 ％ (男性)  4.7 ％ (女性) | データなし (注) | | (胃・十二指腸がんの家族歴がある場合に考慮)  30~35歳以降：  ヘリコバクター・ピロリ菌除菌  1~3年ごとに上部消化管内視鏡検査 | |
| 腎盂・尿管がん | 2.2 % (男性)  1.1 % (女性) | ≤1%~3.7 % | | (尿路上皮がんの家族歴がある場合に考慮)  30~35歳以降：  1年ごとに検尿 (または尿細胞診) | |
| 膀胱がん | 2.1 % (男性)  0.7 % (女性) | ≤1~2.4 % | |  |  |

(注) 記載した確率は、東アジア人では欧米諸国に比べ高いことが知られています^4)^。リンチ症候群と診断された日本人において60~70歳までに胃がんに罹患する確率は14.5~24%との報告^5,6)^があります。

※*PMS2遺伝子*の変化が生まれつきの変化であっても、必ずがんを発症するというわけではありません。

※遺伝子の変化をもっていた場合の罹患する確率、推奨される対応の記載は日本の診療ガイドライン^3)^および海外のガイドライン (米国)^2)^ を参照しています。推奨事項については、各医療機関において必ずしも実施されているとは限りませんので、詳細については各施設の専門診療科あるいは遺伝専門外来におたずねください。

※表は2021年時点の記載です。今後、研究が進み推奨内容が変わる可能性があります。

【参考文献】

- 国立がん研究センターがん情報サービス「累積がん罹患リスク (2018年データ)」<https://ganjoho.jp/reg_stat/statistics/stat/summary.html> [ref. 1]
- NCCN Guidelines® Genetic/Familial High-Risk Assessment: Colorectal. ver.1.2021 (2021.5.11) [ref. 2]
- 大腸癌研究会. 遺伝性大腸癌診療ガイドライン 2020年版. (2020年4月) [ref. 3]
- Managing gastric cancer risk in lynch syndrome: controversies and recommendations. Fam Cancer, 2021 (online ahead of print). [ref. 4]
- Tumor development in Japanese patients with Lynch syndrome. PLoS One, 2018; 13(4): e0195572. [ref. 5]
- Comparison of clinical features between suspected familial colorectal cancer type X and Lynch syndrome in Japanese patients with colorectal cancer: a cross-sectional study conducted by the Japanese Society for Cancer of the Colon and Rectum. Jpn J Clin Oncol, 2015; 45(2): 153. [ref. 6]
- GeneReviews Japan: リンチ症候群. ver.2021.4.21
- ClinGen Actionability Reports: MLH1, MSH2, MSH6, PMS2, EPCAM Adult. ver.1.1.2 (2021.9.15)

監修：厚労科研小杉班・Actionability Working Group-Japan

編集： MONSTAR-SCREEN-2遺伝事務局

***POLD1*遺伝子について**

*がんの発症と関連する遺伝子の変化について*

- - 一般的にがんの5~10%は「生まれつきの遺伝子の変化」が原因といわれています。
  - 「生まれつきの遺伝子の変化」をもっている場合、がんになりやすい体質をもっていると考えられます。
  - がんになりやすい体質は親、子、兄弟、姉妹 などの血のつながった家族と共有している可能性があります。
  - 体質を知ることで、その情報をご自身やご家族の健康管理に役立てることができます。
  - 今回の遺伝子検査でみつかった変化が「生まれつきの遺伝子の変化」かどうかは、血液検査により確認できます。

*POLD1*遺伝子とは？

- *POLD1遺伝子*の生まれつきの変化は**ポリメラーゼ校正関連ポリポーシス**の原因となることが知られています。裏面の資料 (表) をご参照ください。

「生まれつきの遺伝子の変化」であるかどうかを知ることはどんなことに役立ちますか？

- 発症リスクが高いがんを知り、それに対する検診を行うことで、早期発見につながる可能性があります。


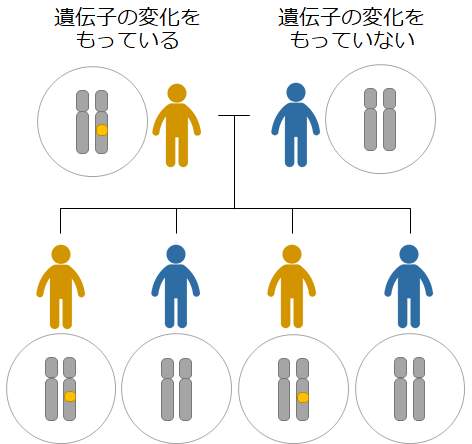
家族にどんな影響がありますか？

- 人は２つの*POLD1*遺伝子をそれぞれ両親から1つずつ受け継いでいます。
- *POLD1*遺伝子の変化を生まれつきもっていた場合、両親、兄弟、姉妹、子どもが同じ変化をもっている確率は50%です。同じ変化をもっているかは、遺伝子検査で確認することができます。
- おば、おじ、いとこなども一定の確率で*同じ遺伝子の変化をもっている*可能性があります。
- 遺伝子の変化についてご家族の方と情報共有することは、ご家族の方の健康管理に役立つ可能性があります。

※生まれつきの*POLD1*遺伝子の変化は、両親から受け継いだものではなく、新規に起こった変化である可能性もあります。この場合のご家族への影響については、遺伝専門外来におたずねください。

遺伝カウンセリングについて

遺伝カウンセリングでは、生まれつきの遺伝子の変化と体質との関連、ご家族への影響などについてより詳しくご説明します。お気軽にご相談ください。　注意：遺伝学的検査の保険適用には条件があり、関連する症状を発症していないご家族の遺伝子検査や検診は自費となります。詳しくは担当者におたずね下さい。

＜参考資料表＞

|  | 一般的な日本人 | *POLD1*遺伝子に病気の原因となる変化を生まれつきもっている | | | | |
| --- | --- | --- | --- | --- | --- | --- |
|  | 生涯を通して罹患する確率^1,2)^ | 生涯を通して  罹患する確率 | | 推奨される対応 | | |
|  |  | 女性^3,4)^ | 男性^3,4)^ | 女性^5)^ | | 男性^5)^ |
| 大腸がん | 10.2 ％ (男性)  8.0 ％ (女性) | 52 % | 63 % | 25~30歳以降：  2~3年ごとに大腸内視鏡検査 (注) | | |
| 子宮内膜がん | 2.0% (女性) | リスクが上がる  可能性 | ― | 現時点で検診の推奨事項は確立されていません。 | ― | |
| 乳がん | 10.9% (女性) | リスクが上がる  可能性 | ― |  |  |  |
| 脳腫瘍 | 0.3 ％ (男性)  0.2 ％ (女性) | リスクが上がる可能性 | | 現時点で検診の推奨事項は確立されていません。 | | |

(注) 記載の推奨事項については、本邦での具体的な検診方法は示されていません。詳細は各施設の遺伝専門外来におたずねください。

※*POLD1遺伝子*の変化が生まれつきの変化であっても、必ずがんを発症するというわけではありません。

※検診等の推奨については、現状では一般的ながん検診を積極的に受検することが推奨されます。

※表は2021年時点の記載です。今後、研究が進み推奨内容が変わる可能性があります。

【参考文献】

- 国立がん研究センターがん情報サービス「累積がん罹患リスク (2018年データ)」[https://ganjoho.jp/reg_stat/statistics/stat/summary.htmll](https://ganjoho.jp/reg_stat/statistics/stat/summary.html) [ref. 1]
- 厚生労働省健康局がん・疾病対策課. 平成30年全国がん登録 罹患数・率報告 2018. [ref. 2]
- Risk of colorectal cancer for carriers of a germ-line mutation in POLE or POLD1. Genet Med. 2018; 20(8): 890. [ref. 3]
- 大腸癌研究会. 遺伝性大腸癌診療ガイドライン 2020年版. (2020年4月) [ref. 4]
- NCCN Guidelines® Genetic/Familial High-Risk Assessment: Colorectal. ver.1.2021 (2021.5.11) [ref. 5]
- ClinGen Actionability Reports: POLE, POLD1 Adult. ver.1.1.3 (2021.11.15)

監修：厚労科研小杉班・Actionability Working Group-Japan

編集： MONSTAR-SCREEN-2遺伝事務局

***POLE*遺伝子について**

*がんの発症と関連する遺伝子の変化について*

- - 一般的にがんの5~10%は「生まれつきの遺伝子の変化」が原因といわれています。
  - 「生まれつきの遺伝子の変化」をもっている場合、がんになりやすい体質をもっていると考えられます。
  - がんになりやすい体質は親、子、兄弟、姉妹 などの血のつながった家族と共有している可能性があります。
  - 体質を知ることで、その情報をご自身やご家族の健康管理に役立てることができます。
  - 今回の遺伝子検査でみつかった変化が「生まれつきの遺伝子の変化」かどうかは、血液検査により確認できます。

*POLE*遺伝子とは？

- *POLE遺伝子*の生まれつきの変化は**ポリメラーゼ校正関連ポリポーシス**の原因となることが知られています。裏面の資料 (表) をご参照ください

「生まれつきの遺伝子の変化」であるかどうかを知ることはどんなことに役立ちますか？

- 発症リスクが高いがんを知り、それに対する検診を行うことで、早期発見につながる可能性があります。


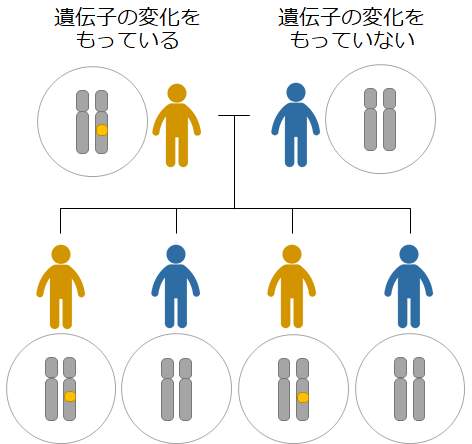


家族にどんな影響がありますか？

- 人は２つの*POLE*遺伝子をそれぞれ両親から1つずつ受け継いでいます。
- *POLE*遺伝子の変化を生まれつきもっていた場合、両親、兄弟、姉妹、子どもが同じ変化をもっている確率は50%です。同じ変化をもっているかは、遺伝子検査で確認することができます。
- おば、おじ、いとこなども一定の確率で*同じ遺伝子の変化をもっている*可能性があります。
- 遺伝子の変化についてご家族の方と情報共有することは、ご家族の方の健康管理に役立つ可能性があります。

※生まれつきの*POLE*遺伝子の変化は、両親から受け継いだものではなく、新規に起こった変化である可能性もあります。この場合のご家族への影響については、遺伝専門外来におたずねください。

遺伝カウンセリングについて

遺伝カウンセリングでは、生まれつきの遺伝子の変化と体質との関連、ご家族への影響などについてより詳しくご説明します。お気軽にご相談ください。　注意：遺伝学的検査の保険適用には条件があり、関連する症状を発症していないご家族の遺伝子検査や検診は自費となります。詳しくは担当者におたずね下さい。

＜参考資料表＞

|  | 一般的な日本人 | *POLE*遺伝子に病気の原因となる変化を生まれつきもっている | | | |
| --- | --- | --- | --- | --- | --- |
|  | 生涯を通して罹患する確率^1,2)^ | 生涯を通して  罹患する確率 | | 推奨される対応 | |
|  |  | 女性^3,4)^ | 男性^3,4)^ | 女性^5)^ | 男性^5)^ |
| 大腸がん | 10.2 ％ (男性)  8.0 ％ (女性) | 32 % | 40 % | 25~30歳以降：  2~3年ごとに大腸内視鏡検査 (注) | |
| 十二指腸がん | （小腸がん）  0.2 % (男性)  0.1 % (女性) | リスクが上がる可能性 | | 現時点で検診の推奨事項は確立されていません。 | |
| 脳腫瘍 | 0.3 ％ (男性)  0.2 ％ (女性) | リスクが上がる可能性 | | 現時点で検診の推奨事項は確立されていません。 | |

(注) 記載の推奨事項については、本邦での具体的な検診方法は示されていません。詳細は各施設の遺伝専門外来におたずねください。

※*POLE遺伝子*の変化が生まれつきの変化であっても、必ずがんを発症するというわけではありません。

※検診等の推奨については、現状では一般的ながん検診を積極的に受検することが推奨されます。

※表は2021年時点の記載です。今後、研究が進み推奨内容が変わる可能性があります。

【参考文献】

- 国立がん研究センターがん情報サービス「累積がん罹患リスク (2018年データ)」 <https://ganjoho.jp/reg_stat/statistics/stat/summary.html> [ref. 1]
- 厚生労働省健康局がん・疾病対策課. 平成30年全国がん登録 罹患数・率報告 2018. [ref. 2]
- Risk of colorectal cancer for carriers of a germ-line mutation in POLE or POLD1. Genet Med, 2018; 20(8): 890. [ref. 3]
- 大腸癌研究会. 遺伝性大腸癌診療ガイドライン 2020年版. (2020年4月) [ref. 4]
- NCCN Guidelines® Genetic/Familial High-Risk Assessment: Colorectal. ver.1.2021 (2021.5.11) [ref. 5]
- ClinGen Actionability Reports: POLE, POLD1 Adult.ver.1.1.3 (2021.11.15)

監修：厚労科研小杉班・Actionability Working Group-Japan

編集： MONSTAR-SCREEN-2遺伝事務局

***POT1*遺伝子について**

*がんの発症と関連する遺伝子の変化について*

- - 一般的にがんの5~10%は「生まれつきの遺伝子の変化」が原因といわれています。
  - 「生まれつきの遺伝子の変化」をもっている場合、がんになりやすい体質をもっていると考えられます。
  - がんになりやすい体質は親、子、兄弟、姉妹 などの血のつながった家族と共有している可能性があります。
  - 体質を知ることで、その情報をご自身やご家族の健康管理に役立てることができます。
  - 今回の遺伝子検査でみつかった変化が「生まれつきの遺伝子の変化」かどうかは、血液検査により確認できます。

*POT1*遺伝子とは？

- がんを発症するリスクと関連があることが示されている遺伝子です^1)^。裏面の資料 (表) をご参照ください。

「生まれつきの遺伝子の変化」であるかどうかを知ることはどんなことに役立ちますか？

- 発症リスクが高いがんを知り、それに対する検診を行うことで、早期発見につながる可能性があります。

家族にどんな影響がありますか？
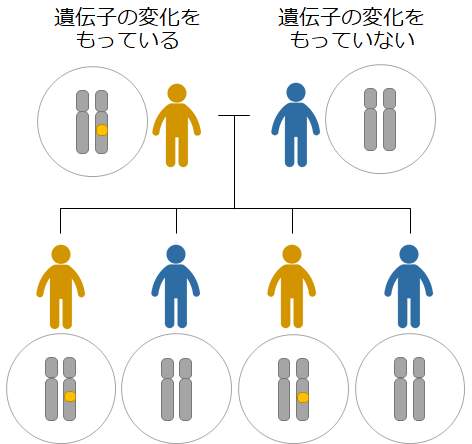


- 人は２つの*POT1*遺伝子をそれぞれ両親から1つずつ受け継いでいます。
- *POT1*遺伝子の変化を生まれつきもっていた場合、両親、兄弟、姉妹、子どもが同じ変化をもっている確率は50%です。同じ変化をもっているかは、遺伝子検査で確認することができます。
- おば、おじ、いとこなども一定の確率で*同じ遺伝子の変化をもっている*可能性があります。
- 遺伝子の変化についてご家族の方と情報共有することは、ご家族の方の健康管理に役立つ可能性があります。

※生まれつきの*POT1*遺伝子の変化は、両親いずれかから受け継いだものではなく、新規に起こった変化である可能性もあります。この場合のご家族への影響については、遺伝専門外来におたずねください。

遺伝カウンセリングについて

遺伝カウンセリングでは、生まれつきの遺伝子の変化と体質との関連、ご家族への影響などについてより詳しくご説明します。お気軽にご相談ください。　注意：遺伝学的検査の保険適用には条件があり、関連する症状を発症していないご家族の遺伝子検査や検診は自費となります。詳しくは担当者におたずね下さい。

＜参考資料表＞

|  | 一般的な日本人 | *POT1*遺伝子に病気の原因となる変化を生まれつきもっている | | | |
| --- | --- | --- | --- | --- | --- |
|  | 生涯を通して罹患する確率^2)^ | 生涯を通して  罹患する確率 | | 推奨される対応 | |
|  |  | 女性^3)^ | 男性^3)^ | 女性^4)^ | 男性^4)^ |
| 悪性黒色腫 (注1) | 0.1 % (男性)  0.1 % (女性) | リスクが高まる可能性 | | 皮膚、頭皮、口腔粘膜、生殖器の皮膚科での精密検査（半年に1回、母斑が安定している場合は年１回) (注2)  月１回の母斑自己検診 (注2) | |

- 上記症状以外にも、血液、神経などに症状がみられることがあります。詳細は遺伝専門外来におたずねください。

(注1) 悪性黒色腫の罹病率 (年) は、白人が 24.3 人/10 万人、アジア人が 1.7 人/10 万人との報告があり^5)^、人種、地域、その他の遺伝的要因によってリスクは異なると考えられています。

(注2) 記載の推奨事項については、本邦での具体的な検診方法は示されていません。詳細は各施設の遺伝専門外来におたずねください

※*POT1遺伝子*の変化が生まれつきの変化であっても、必ずがんを発症するというわけではありません。

※遺伝子の変化をもっていた場合の罹患する確率、推奨される対応は海外の資料^3,4)^ を参照しています。

※表は2021年時点の記載です。今後、研究が進み推奨内容が変わる可能性があります。

【参考文献】

- *CDKN2A* genetic testing in melanoma-prone families in Sweden in the years 2015-2020: implications for novel national recommendations. Acta Oncologica, 2021; 60(7): 888. [ref. 1]
- 厚生労働省健康局がん・疾病対策課. 平成30年全国がん登録 罹患数・率報告 2018 [ref. 2]
- GeneReviews® *POT1* Tumor Predisposition. ver.2020.10.29 [ref. 3]
- ClinGen Actionability Reports: CDKN2A Adult. ver.1.2.1 (2020.8.19) [ref. 4]
- 日本皮膚科学会. 皮膚悪性腫瘍ガイドライン第 3 版 メラノーマ診療ガイドライン 2019. 日皮会誌, 2019; 129(9):1759. [ref. 5]

監修：厚労科研小杉班・Actionability Working Group-Japan

編集： MONSTAR-SCREEN-2遺伝事務局

***PTEN*遺伝子について**

*がんの発症と関連する遺伝子の変化について*

- - 一般的にがんの5~10%は「生まれつきの遺伝子の変化」が原因といわれています。
  - 「生まれつきの遺伝子の変化」をもっている場合、がんになりやすい体質をもっていると考えられます。
  - がんになりやすい体質は親、子、兄弟、姉妹 などの血のつながった家族と共有している可能性があります。
  - 体質を知ることで、その情報をご自身やご家族の健康管理に役立てることができます。
  - 今回の遺伝子検査でみつかった変化が「生まれつきの遺伝子の変化」かどうかは、血液検査により確認できます。

*PTEN*遺伝子とは？

- *PTEN遺伝子*の生まれつきの変化は***PTEN*過誤腫症候群 (カウデン症候群)** の原因となることが知られています。裏面の (表) をご参照ください。

「生まれつきの遺伝子の変化」であるかどうかを知ることはどんなことに役立ちますか？

- 発症リスクが高いがんを知り、それに対する検診を行うことで、早期発見につながる可能性があります。


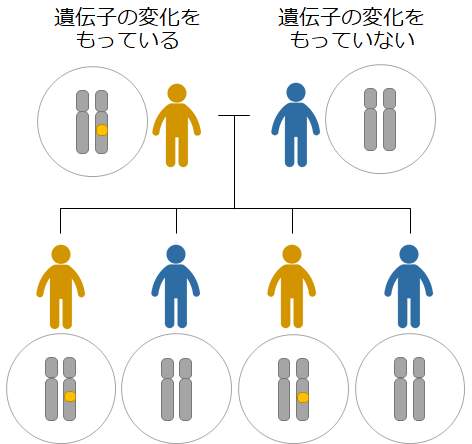
家族にどんな影響がありますか？

- 人は２つの*PTEN*遺伝子をそれぞれ両親から1つずつ受け継いでいます。
- *PTEN*遺伝子の変化を生まれつきもっていた場合、両親、兄弟、姉妹、子どもが同じ変化をもっている確率は50%です。同じ変化をもっているかは、遺伝子検査で確認することができます。
- おば、おじ、いとこなども一定の確率で*同じ遺伝子の変化をもっている*可能性があります。
- 遺伝子の変化についてご家族の方と情報共有することは、ご家族の方の健康管理に役立つ可能性があります。

※生まれつきの*PTEN*遺伝子の変化は、両親から受け継いだものではなく、新規に起こった変化である可能性もあります。この場合のご家族への影響については、遺伝専門外来におたずねください。

遺伝カウンセリングについて

遺伝カウンセリングでは、生まれつきの遺伝子の変化と体質との関連、ご家族への影響などについてより詳しくご説明します。お気軽にご相談ください。　注意：遺伝学的検査の保険適用には条件があり、関連する症状を発症していないご家族の遺伝子検査や検診は自費となります。詳しくは担当者におたずね下さい。

＜参考資料表＞

|  | 一般的な  日本人 | *PTEN*遺伝子に病気の原因となる変化を生まれつきもっている | | | |
| --- | --- | --- | --- | --- | --- |
|  | 生涯で  罹患する確率^1)^ | 生涯を通して  罹患する確率 | | 推奨される対応 | |
|  |  | 女性^2)^ | 男性^2)^ | 女性^2,3)^ | 男性^2,3)^ |
| 乳腺良性病変/  乳がん | (乳がん)  10.9 ％  (女性) | (過誤腫性病変)  一般に比べ罹患率が高いとのエビデンスはない  (乳がん)  25~85 ％^3)^ | ＊症例報告レベル明らかなリスク増加につながるとのエビデンスはない | 18歳以降：自己乳房検診  25歳以降**：半年～1年ごとに医師による視触診  35歳以降**：年1回の乳房マンモグラフィ、乳房造影MRI検査  **家系員の最も若い乳がん診断年齢より5~10歳早い年齢 | |
| 甲状腺良性腫瘍/がん | (甲状腺がん)  10.8 ％  (男女) | (良性甲状腺) 30~68 %  (甲状腺がん) 10~35 ％^1)^ | | 7歳または診断時以降：年１回の甲状腺超音波検査 | |
| 子宮内膜がん | (子宮がん)  2.0%  (女性) | (子宮内膜がん)  19~28 % | ― | 35歳以降：1~2年ごとの子宮内膜細胞診  閉経後：経腟超音波検査を考慮  出産完了後：子宮摘出の選択肢について話し合うことができる | |
| 大腸ポリープ/  大腸がん | (大腸がん)  8.1 %  (女性)　10.3 % (男性) | (消化管ポリープ) 頻度90 %以上  (がん) 9~16 % | | 35歳以降***：5年ごとの大腸内視鏡検査  ***40歳以前に診断された家系員がいる場合、診断年齢より5~10歳早い年齢 | |
| 腎細胞がん | 6人  /10万人 | 34% | | 40歳以降：1~2年ごとの腎超音波検査 | |

- 上表以外にも、皮膚、神経、血管などに症状がみられることがあります。詳細は専門診療科あるいは遺伝専門外来におたずねください。

※*PTEN遺伝子*の変化が生まれつきの変化であっても、必ずがんを発症するというわけではありません。

※遺伝子の変化をもっていた場合の罹患する確率、推奨される対応の記載は日本の診療ガイドライン^2)^および米国のガイドライン (NCCNガイドライン)^3)^ を参照しています。推奨事項については、日本で必ずしも実施されているとは限りませんので、詳細については各施設の専門診療科あるいは遺伝専門外来におたずねください。

※表は2021年時点の記載です。今後、研究が進み推奨内容が変わる可能性があります。

【参考文献】

- 国立がん研究センターがん情報サービス「累積がん罹患リスク (2018年データ)」<https://ganjoho.jp/reg_stat/statistics/stat/summary.html> [ref. 1]
- 小児・成人のためのCowden症候群/PTEN過誤腫症候群診療ガイドライン 2020年版. 遺伝性腫瘍, 2020; 20(2): 93. [ref. 2]
- NCCN Guidelines® Genetic/Familial High-Risk Assessment: Breast, Ovarian, and Pancreatic. ver.1.2022 (2021.8.11) [ref. 3]
- GeneRevies Japan: *PTEN*過誤腫症候群. ver.2021.3.1.
- ClinGen Actionability Reports: PTEN Adult. ver.2.1.2 (2021.6.21)

監修：厚労科研小杉班・Actionability Working Group-Japan

編集： MONSTAR-SCREEN-2遺伝事務局

***RAD51C*遺伝子について**

*がんの発症と関連する遺伝子の変化について*

- - 一般的にがんの5~10%は「生まれつきの遺伝子の変化」が原因といわれています。
  - 「生まれつきの遺伝子の変化」をもっている場合、がんになりやすい体質をもっていると考えられます。
  - がんになりやすい体質は親、子、兄弟、姉妹 などの血のつながった家族と共有している可能性があります。
  - 体質を知ることで、その情報をご自身やご家族の健康管理に役立てることができます。
  - 今回の遺伝子検査でみつかった変化が「生まれつきの遺伝子の変化」かどうかは、血液検査により確認できます。

*RAD51C*遺伝子とは？

- *がんを発症するリスクと関連があることが示されている遺伝子です。裏面の資料* (*表*) *をご参照ください。*

「生まれつきの遺伝子変化」であるかどうかを知ることはどんなことに役立ちますか？

- 発症リスクが高いがんを知り、それに対する検診を行うことで、早期発見につながる可能性があります。

家族にどんな影響がありますか？
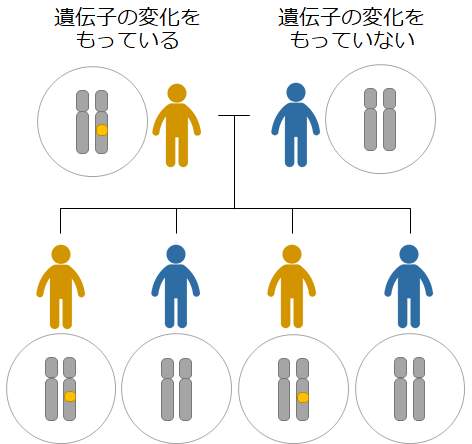


- 人は２つの*RAD51C*遺伝子をそれぞれ両親から1つずつ受け継いでいます。
- *RAD51C*遺伝子の変化を生まれつきもっていた場合、両親、兄弟、姉妹、子どもが同じ変化をもっている確率は50%です。同じ変化をもっているかは、遺伝子検査で確認することができます。
- おば、おじ、いとこなども一定の確率で*同じ遺伝子の変化をもっている*可能性があります。
- 遺伝子の変化についてご家族の方と情報共有することは、ご家族の方の健康管理に役立つ可能性があります。

※生まれつきの*RAD51C*遺伝子の変化は、両親いずれかから受け継いだものではなく、新規に起こった変化である可能性もあります。この場合のご家族への影響については、遺伝専門外来におたずねください。

遺伝カウンセリングについて

遺伝カウンセリングでは、生まれつきの遺伝子の変化と体質との関連、ご家族への影響などについてより詳しくご説明します。お気軽にご相談ください。 注意：遺伝学的検査の保険適用には条件があり、関連する症状を発症していないご家族の遺伝子検査や検診は自費となります。詳しくは担当者におたずね下さい。

＜参考資料表＞

|  | 一般的な日本人 | *RAD51C遺伝子*に病気の原因となる変化を生まれつきもっている場合 | | | |
| --- | --- | --- | --- | --- | --- |
|  | 生涯を通して  罹患する確率^1)^ | 生涯を通して  罹患する確率 | | 推奨される対応 | |
|  |  | 女性^2)^ | 男性 | 女性^2)^ | 男性 |
| 卵巣がん | 1.6 % (女性) | >10 % | ― | 45~50歳以降:リスク低減卵管卵巣摘出術 (RRSO) を考慮。(注)  ※家族歴や既往歴によってリスクは異なると考えられています。現時点では検診方法は確立されていません。 | ― |
| 乳がん | 10.9 ％ (女性) | 15～40 ％ | ― | ※家族歴や既往歴によってリスクは異なると考えられています。現時点では検診方法は確立されていません。 | ― |

(注) 記載の推奨事項については、本邦での具体的な検診方法は示されていません。詳細は各施設の遺伝専門外来におたずねください。

※*RAD51C遺伝子*の変化が生まれつきの変化であっても、必ずがんを発症するというわけではありません。

※遺伝子の変化をもっていた場合の罹患する確率、推奨される対応の記載は米国のガイドライン (NCCNガイドライン)^2)^ を参照しています。

※表は2021年時点の記載です。今後、研究が進み推奨内容が変わる可能性があります。

【参考文献】

- 国立がん研究センターがん情報サービス「累積がん罹患リスク (2018年データ)」https://ganjoho.jp/reg_stat/statistics/stat/summary.html [ref. 1]
- NCCN Guidelines® Genetic/Familial High-Risk Assessment: Breast, Ovarian, and Pancreatic. ver.1.2022 (2021.8.11) [ref. 2]
- ClinGen Actionability Reports: BRIP1, RAD51C, RAD51D Adult. ver.1.1.1 (2020.7.13)

監修：厚労科研小杉班・Actionability Working Group-Japan

編集： MONSTAR-SCREEN-2遺伝事務局

***RAD51D*遺伝子について**

*がんの発症と関連する遺伝子の変化について*

- - 一般的にがんの5~10%は「生まれつきの遺伝子の変化」が原因といわれています。
  - 「生まれつきの遺伝子の変化」をもっている場合、がんになりやすい体質をもっていると考えられます。
  - がんになりやすい体質は親、子、兄弟、姉妹 などの血のつながった家族と共有している可能性があります。
  - 体質を知ることで、その情報をご自身やご家族の健康管理に役立てることができます。
  - 今回の遺伝子検査でみつかった変化が「生まれつきの遺伝子の変化」かどうかは、血液検査により確認できます。

*RAD51D*遺伝子とは？

- がんを*発症するリスクと関連があることが示されている遺伝子です。裏面の資料* (*表*) *をご参照ください。*

「生まれつきの遺伝子変化」であるかどうかを知ることはどんなことに役立ちますか？

- 発症リスクが高いがんを知り、それに対する検診を行うことで、早期発見につながる可能性があります。


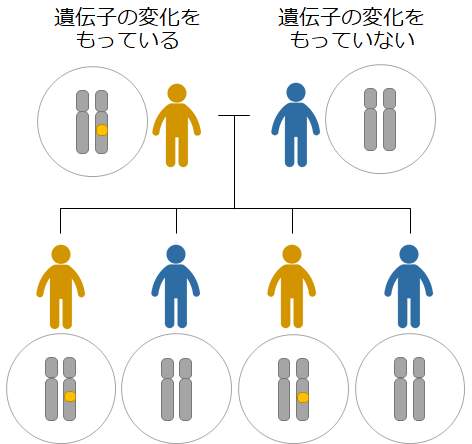
家族にどんな影響がありますか？

- 人は２つの*RAD51D*遺伝子をそれぞれ両親から1つずつ受け継いでいます。
- *RAD51D*遺伝子の変化を生まれつきもっていた場合、両親、兄弟、姉妹、子どもが同じ変化をもっている確率は50%です。同じ変化をもっているかは、遺伝子検査で確認することができます。
- おば、おじ、いとこなども一定の確率で*同じ遺伝子の変化をもっている*可能性があります。
- 遺伝子の変化についてご家族の方と情報共有することは、ご家族の方の健康管理に役立つ可能性があります。

※生まれつきの*RAD51D*遺伝子の変化は、両親いずれかから受け継いだものではなく、新規に起こった変化である可能性もあります。この場合のご家族への影響については、遺伝専門外来におたずねください。

遺伝カウンセリングについて

遺伝カウンセリングでは、生まれつきの遺伝子の変化と体質との関連、ご家族への影響などについてより詳しくご説明します。お気軽にご相談ください。 注意：遺伝学的検査の保険適用には条件があり、関連する症状を発症していないご家族の遺伝子検査や検診は自費となります。詳しくは担当者におたずね下さい。

＜参考資料表＞

|  | 一般的な日本人 | *RAD51D遺伝子*に病気の原因となる変化を生まれつきもっている場合 | | | |
| --- | --- | --- | --- | --- | --- |
|  | 生涯を通して  罹患する確率^1)^ | 生涯を通して  罹患する確率 | | 推奨される対応 | |
|  |  | 女性^2)^ | 男性 | 女性^2)^ | 男性 |
| 卵巣がん | 1.6 % (女性) | >10 % | ― | 45~50歳以降:リスク低減卵管卵巣摘出術(RRSO) を考慮。(注)  ※家族歴や既往歴によってリスクは異なると考えられています。現時点では検診方法は確立されていません。 | ― |
| 乳がん | 10.9 ％ (女性) | 15～40 ％ | ― | ※家族歴や既往歴によってリスクは異なると考えられています。現時点では検診方法は確立されていません。 | ― |

(注) 記載の推奨事項については、本邦での具体的な検診方法は示されていません。詳細は各施設の遺伝専門外来におたずねください。

※*RAD51D遺伝子*の変化が生まれつきの変化であっても、必ずがんを発症するというわけではありません。

※遺伝子の変化をもっていた場合の罹患する確率、推奨される対応の記載は米国のガイドライン (NCCNガイドライン)^2)^ を参照しています。

※表は2021年時点の記載です。今後、研究が進み推奨内容が変わる可能性があります。

【参考文献】

- 国立がん研究センターがん情報サービス 「累積がん罹患リスク (2018年データ)」 https://ganjoho.jp/reg_stat/statistics/stat/summary.html [ref. 1]
- NCCN Guidelines® Genetic/Familial High-Risk Assessment: Breast, Ovarian, and Pancreatic. ver.1.2022 (2021.8.11) [ref. 2]
- ClinGen Actionability Reports: BRIP1, RAD51C, RAD51D Adult. ver.1.1.1 (2020.7.13)

監修：厚労科研小杉班・Actionability Working Group-Japan

編集： MONSTAR-SCREEN-2遺伝事務局

***RB1*遺伝子について**

*がんの発症と関連する遺伝子の変化について*

- 一般的にがんの5~10%は「生まれつきの遺伝子の変化」が原因といわれています。
  - 「生まれつきの遺伝子の変化」をもっている場合、がんになりやすい体質をもっていると考えられます。
  - がんになりやすい体質は親、子、兄弟、姉妹などの血のつながった家族と共有している可能性があります。
  - 体質を知ることで、その情報をご自身やご家族の健康管理に役立てることができます。
  - 今回の遺伝子検査でみつかった変化が「生まれつきの遺伝子の変化」かどうかは、血液検査により確認できます。

*RB1*遺伝子とは？

- *RB1*遺伝子*の生まれつきの変化*は**網膜芽細胞腫***の原因となることが知られています。*裏面の資料 (表) をご参照ください。

「生まれつきの遺伝子の変化」であるかどうかを知ることはどんなことに役立ちますか？

- 発症リスクが高い腫瘍を知り、それに対する検診を行うことで、ご自身とご家族の体質に合わせた検診につながる可能性があります。

家族にどんな影響がありますか？
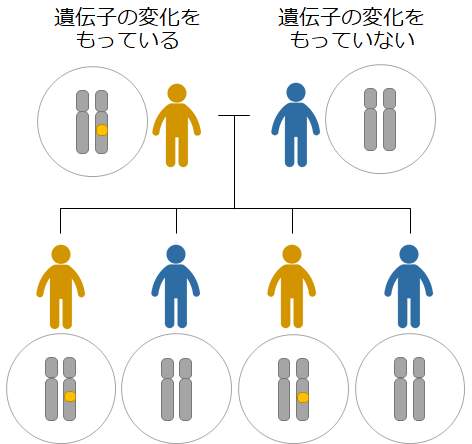


- 人は２つの*RB1*遺伝子をそれぞれ両親から1つずつ受け継いでいます。
- *RB1*遺伝子の変化を生まれつきもっていた場合、両親、兄弟、姉妹、子供が同じ変化をもっている確率は50%です。同じ変化をもっているかは、遺伝子検査で確認することができます。
- おば、おじ、いとこなども一定の確率で*同じ遺伝子の変化をもっている*可能性があります。
- 遺伝子の変化についてご家族の方と情報共有することは、ご家族の方の健康管理に役立つ可能性があります。

※生まれつきの*RB1*遺伝子の変化は、両親いずれかから受け継いだものではなく、新規に起こった変化である可能性が約80%^1)^ (注意：網膜芽細胞腫として) といわれています。この場合のご家族への影響については、遺伝専門外来におたずねください。

遺伝カウンセリングについて

遺伝カウンセリングでは、生まれつきの遺伝子の変化と体質との関連、ご家族への影響などについてより詳しくご説明します。お気軽にご相談ください。　注意：遺伝学的検査の保険適用には条件があり、関連する症状を発症していないご家族の遺伝子検査や検診は自費となります。詳しくは担当者におたずね下さい。

＜参考資料表＞

|  | 一般的な日本人 | *RB1*遺伝子に病気の原因となる変化を生まれつきもっている | | | |
| --- | --- | --- | --- | --- | --- |
|  | 生涯を通して  罹患する確率^2)^ | 生涯を通して  罹患する確率 | | 推奨される対応 | |
|  |  | 女性^2)^ | 男性^2)^ | 女性^2)^ | 男性^2)^ |
| 網膜芽細胞腫 | データなし  ※小児がんの2.5~4％ | 90% | | 3~4歳まで3~4か月ごと、5~6歳まで6か月ごとに眼底検査 | |

※記載は日本の診療ガイドライン^2)^を参照しています。詳細は各施設の専門診療科あるいは遺伝専門外来におたずねください。

※表は2021年時点の記載です。今後、研究が進み推奨内容が変わる可能性があります。

【参考文献】

- Retinoblastoma and Neuroblastoma Predisposition and Surveillance. Clin Cancer Res, 2017; 23(13): e98. [ref. 1]
- 日本小児血液がん学会. 小児がん診療ガイドライン (2016年版). (2016年8月) [ref. 2]
- GeneReviews®: Retinoblastoma ver.2018.11.21.
- ClinGen Actionability Reports: RB1 Pediatric. ver.1.3.2 (2021.10.18)

監修：厚労科研小杉班・Actionability Working Group-Japan

編集： MONSTAR-SCREEN-2遺伝事務局

***RET*遺伝子について**

*がんの発症と関連する遺伝子の変化について*

- 一般的にがんの5~10%は「生まれつきの遺伝子の変化」が原因といわれています。
  - 「生まれつきの遺伝子の変化」をもっている場合、がんになりやすい体質をもっていると考えられます。
  - がんになりやすい体質は親、子、兄弟、姉妹などの血のつながった家族と共有している可能性があります。
  - 体質を知ることで、その情報をご自身やご家族の健康管理に役立てることができます。
  - 今回の遺伝子検査でみつかった変化が「生まれつきの遺伝子の変化」かどうかは、血液検査により確認できます。

*RET*遺伝子とは？

- *RET*の生まれつきの変化は**多発性内分泌腫瘍症2型**の原因となることが知られています。裏面の資料 (表) をご参照ください。

「生まれつきの遺伝子の変化」であるかどうかを知ることはどんなことに役立ちますか？

- 発症リスクが高い症状を知り、それに対する検診を行うことで、早期発見につながる可能性があります。

家族にどんな影響がありますか？
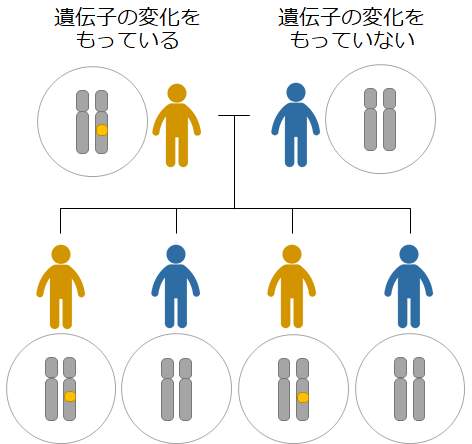


- 人は２つの*RET*遺伝子をそれぞれ両親から1つずつ受け継いでいます。
- *RET*遺伝子の変化を生まれつきもっていた場合両親、兄弟、姉妹、子供が同じ変化をもっている確率は50%です。同じ変化をもっているかは、遺伝子検査で確認することができます。
- おば、おじ、いとこなども一定の確率で*同じ遺伝子の変化をもっている*可能性があります。
- 遺伝子の変化についてご家族の方と情報共有することは、ご家族の方の健康管理に役立つ可能性があります。

※生まれつきの*RET*遺伝子の変化は、両親いずれかから受け継いだものではなく、新規に起こった変化である可能性もあります。この場合のご家族への影響については、遺伝専門外来におたずねください。

遺伝カウンセリングについて

遺伝カウンセリングでは、生まれつきの遺伝子の変化と体質との関連、ご家族への影響などについてより詳しくご説明します。お気軽にご相談ください。　注意：遺伝学的検査の保険適用には条件があり、関連する症状を発症していないご家族の遺伝子検査や検診は自費となります。詳しくは担当者におたずね下さい。

＜参考資料表＞

|  | 一般的な日本人 | *RET*遺伝子に病気の原因となる変化を生まれつきもっている | | | |
| --- | --- | --- | --- | --- | --- |
|  | 生涯を通して  罹患する確率^2,3,4)^ | 生涯を通して  罹患する確率 | | 推奨される対応 | |
|  |  | 女性^5)^ | 男性^5)^ | 女性^5,6)^ | 男性^5,6)^ |
| 甲状腺  髄様がん | 0.09 ％ | 90 ％ | | 1年ごとに血液検査 (カルシトニン) と頸部超音波検査  ※検査開始年齢は、遺伝子の変化の箇所により異なります。詳しくは遺伝専門外来にておたずねください。 | |
| 副腎  褐色細胞腫 | 0.01~0.02 ％ | 30~60 % | | 2~3年ごとに血中遊離メタネフリンと腹部単純CTまたはMRI検査  ※検査開始年齢は、遺伝子変化の箇所により異なります。詳しくは遺伝専門外来にておたずねください。 | |
| 原発性副甲状腺機能亢進症 | 4~5千人あたり  1人 | 8.1 ％ | | 血液検査 (血清カルシウム、インタクトPTH), 尿検査 | |

- 上表の症状以外にも、特徴的な症状がみられることがあります。詳細は遺伝専門外来におたずねください。

※今回見つかった*RET遺伝子*の変化が生まれつきの変化であっても、必ず症状がみられるというわけではありません。

※記載は日本の診療ガイドブック^5)^を参照しています。詳細は各施設の遺伝専門外来におたずねください。

※表は2021年時点の記載です。今後、研究が進み推奨内容が変わる可能性があります。

【参考文献】

- GeneReviews Japan: 多発性内分泌腫瘍症2型. ver.2021.9.24 [ref. 1]
- 全国がん登録罹患数・率 報告 平成30年 <https://www.mhlw.go.jp/content/10900000/000794199.pdf> [ref. 2]
- 日本内分泌学会. 褐色細胞腫・パラガングリオーマ診療ガイドライン2018. (2018年7月) [ref. 3]
- Clinical features of multiple endocrine neoplasia type 1 (MEN1) phenocopy without germline MEN1 gene mutations: analysis of 20 Japanese sporadic cases with MEN1. Clin Endocrinol (Oxf), 2000; 52(4): 509. [ref. 4]
- 多発性内分泌腫瘍症診療ガイドブック編集委員会. 多発性内分泌腫瘍症 診療ガイドブック. (2013年4月) [ref. 5]
- RET遺伝子の発症前診断に用いる説明文書 <http://men-net.org/medical/child.html> [ref. 6]
- ClinGen Actionability Reports: RET Adult. ver.2.0.2 (2021.10.18)

監修：厚労科研小杉班・Actionability Working Group-Japan

編集： MONSTAR-SCREEN-2遺伝事務局

***SDHA*遺伝子について**

*腫瘍と関連する遺伝子の変化について*

- 一般的に腫瘍の一部は「生まれつきの遺伝子の変化」が原因といわれています。
  - 「生まれつきの遺伝子の変化」をもっている場合、腫瘍ができやすい体質をもっていると考えられます。
  - 腫瘍ができやすい体質は親、子、兄弟、姉妹などの血のつながった家族と共有している可能性があります。
  - 体質を知ることで、その情報をご自身やご家族の健康管理に役立てることができます。
  - 今回の遺伝子検査でみつかった変化が「生まれつきの遺伝子の変化」かどうかは、血液検査により確認できます。

*SDHA*遺伝子とは？

- 腫瘍を発症するリスクと関連があることが示されている遺伝子です。裏面の資料 (表) をご参照ください。

「生まれつきの遺伝子の変化」であるかどうかを知ることはどんなことに役立ちますか？

- 発症リスクが高い腫瘍を知り、それに対する検診を行うことで、早期発見につながる可能性があります。

家族にどんな影響がありますか？
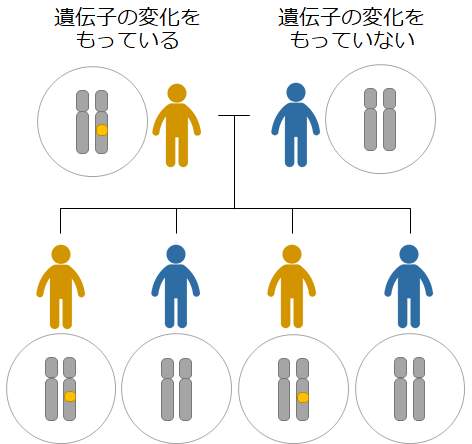


- 人は２つの*SDHA*遺伝子をそれぞれ両親から1つずつ受け継いでいます。
- *SDHA*遺伝子の変化を生まれつきもっていた場合、両親、兄弟、姉妹、子供が同じ変化をもっている確率は50%です。同じ変化をもっているかは、遺伝子検査で確認することができます。
- おば、おじ、いとこなども一定の確率で*同じ遺伝子の変化をもっている*可能性があります。
- 遺伝子の変化についてご家族の方と情報共有することは、ご家族の方の健康管理に役立つ可能性があります。

※生まれつきの*SDHA*遺伝子の変化は、両親いずれかから受け継いだものではなく、新規に起こった変化である可能性もあります。この場合のご家族への影響については、遺伝専門外来におたずねください。

遺伝カウンセリングについて

遺伝カウンセリングでは、生まれつきの遺伝子の変化と体質との関連、ご家族への影響などについてより詳しくご説明します。お気軽にご相談ください。　注意：遺伝学的検査の保険適用には条件があり、関連する症状を発症していないご家族の遺伝子検査や検診は自費となります。詳しくは担当者におたずね下さい。

＜参考資料表＞

|  | 一般的な日本人 | *SDHA*遺伝子に病気の原因となる変化を生まれつきもっている | | | |
| --- | --- | --- | --- | --- | --- |
|  | 生涯を通して  罹患する確率^1,2,3)^ | 生涯を通して  罹患する確率 | | 推奨される対応 | |
|  |  | 女性^3)^ | 男性^3)^ | 女性^3)^ | 男性^3)^ |
| パラガングリ  オーマ | 患者数：全国で  約1500人/年  ※米国データ：100万人に2人 | リスクが上がる  可能性 | | 1年ごとの血液検査(血中遊離メタネフリン)  2年ごとのCTまたはMRI検査  3年ごとのI-MIBGシンチグラフィ(画像検査) | |
| 副腎  褐色細胞腫 | 0.01~0.02 ％ | リスクが上がる  可能性 | |  |  |

※今回見つかった*SDHA遺伝子*の変化が生まれつきの変化であっても、必ず症状がみられるというわけではありません。

※上記に示す罹患する確率は、良性・境界性の腫瘍を含めた確率であり、悪性腫瘍はその一部です。

※上表に示したほか、消化管の腫瘍が生じることがあります。詳細は遺伝専門外来におたずねください。

※記載は日本の診療ガイドライン^3)^を参照しています。詳細については各施設の遺伝専門外来におたずねください。

※表は2021年時点の記載です。今後、研究が進み推奨内容が変わる可能性があります。

【参考文献】

- 国立がん研究センター希少がんセンター: パラガングリオーマ [https://www.ncc.go.jp/jp/rcc/about/paraganguriouma/index.html](https://www.ncc.go.jp/jp/rcc/about/paraganguriouma/index.html%C2%A0) [ref. 1]
- National Cancer Institute Center for Cancer Research <https://www.cancer.gov/pediatric-adult-rare-tumor/rare-tumors/rare-endocrine-tumor/paraganglioma> [ref. 2]
- 日本内分泌学会. 褐色細胞腫・パラガングリオーマ診療ガイドライン2018. (2018年7月) [ref. 3]
- GeneReviews Japan: 遺伝性パラガングリオーマ・褐色細胞腫症候群. ver.2020.7.15
- ClinGen Actionability Reports: MAX, SDHA, SDHAF2, SDHB, SDHC, SDHD, TMEM127 Adult. ver.1.1.3 (2022.1.3)

監修：厚労科研小杉班・Actionability Working Group-Japan

編集： MONSTAR-SCREEN-2遺伝事務局

***SDHAF2*遺伝子について**

腫瘍*と関連する遺伝子の変化について*

- 一般的に腫瘍の一部は「生まれつきの遺伝子の変化」が原因といわれています。
  - 「生まれつきの遺伝子の変化」をもっている場合、生まれつき腫瘍ができやすい体質をもっていると考えられます。
  - 腫瘍ができやすい体質は親、子、兄弟、姉妹などの血のつながった家族と共有している可能性があります。
  - 体質を知ることで、その情報をご自身やご家族の健康管理に役立てることができます。
  - 今回の遺伝子検査でみつかった変化が「生まれつきの遺伝子の変化」かどうかは、血液検査により確認できます。

*SDHAF2*遺伝子とは？

- 腫瘍を発症するリスクと関連があることが示されている遺伝子です。裏面の資料 (表) をご参照ください。

「生まれつきの遺伝子の変化」であるかどうかを知ることはどんなことに役立ちますか？

- 発症リスクが高い腫瘍を知り、それに対する検診を行うことで、早期発見につながる可能性があります。

家族にどんな影響がありますか？
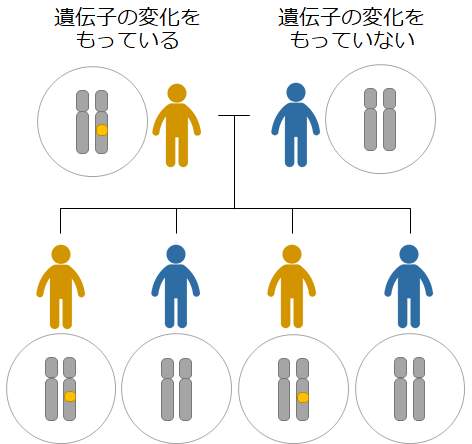


- 人は２つの*SDHAF2*遺伝子をそれぞれ両親から1つずつ受け継いでいます。
- *SDHAF2*遺伝子の変化を生まれつきもっていた場合、両親、兄弟、姉妹、子供が同じ変化をもっている確率は50%です。同じ変化をもっているかは、遺伝子検査で確認することができます。
- おば、おじ、いとこなども一定の確率で*同じ遺伝子の変化をもっている*可能性があります。
- 遺伝子の変化についてご家族の方と情報共有することは、ご家族の方の健康管理に役立つ可能性があります。

※生まれつきの*SDHAF2*遺伝子の変化は、両親いずれかから受け継いだものではなく、新規に起こった変化である可能性もあります。この場合のご家族への影響については、遺伝専門外来におたずねください。

遺伝カウンセリングについて

遺伝カウンセリングでは、生まれつきの遺伝子の変化と体質との関連、ご家族への影響などについてより詳しくご説明します。お気軽にご相談ください。　注意：遺伝学的検査の保険適用には条件があり、関連する症状を発症していないご家族の遺伝子検査や検診は自費となります。詳しくは担当者におたずね下さい。

＜参考資料表＞

|  | 一般的な日本人 | *SDHAF2*遺伝子に病気の原因となる変化を生まれつきもっている | | | |
| --- | --- | --- | --- | --- | --- |
|  | 生涯を通して  罹患する確率^1,2,3)^ | 生涯を通して  罹患する確率 | | 推奨される対応 | |
|  |  | 女性^3)^ | 男性^3)^ | 女性^3)^ | 男性^3)^ |
| パラガングリ  オーマ | 患者数：全国で  約1500人/年  ※米国データ：100万人に2人 | リスクが上がる  可能性 | | 1年ごとの血液検査 (血中遊離メタネフリン)  2年ごとのCTまたはMRI検査  3年ごとのI-MIBGシンチグラフィ (画像検査) | |
| 副腎  褐色細胞腫 | 0.01~0.02 ％ | リスクが上がる  可能性 | |  |  |

※今回見つかった*SDHAF2遺伝子*の変化が生まれつきの変化であっても、必ず症状がみられるというわけではありません。

※上記に示す罹患する確率は、良性・境界性の腫瘍を含めた確率であり、悪性腫瘍はその一部です。

※記載は日本の診療ガイドライン^3)^を参照しています。詳細は各施設の遺伝専門外来におたずねください。

※表は2021年時点の記載です。今後、研究が進み推奨内容が変わる可能性があります。

【参考文献】

- 国立がん研究センター希少がんセンター: パラガングリオーマ <https://www.ncc.go.jp/jp/rcc/about/paraganguriouma/index.htmll> [ref. 1]
- National Cancer Institute Center for Cancer Research <https://www.cancer.gov/pediatric-adult-rare-tumor/rare-tumors/rare-endocrine-tumor/paraganglioma> [ref. 2]
- 日本内分泌学会. 褐色細胞腫・パラガングリオーマ診療ガイドライン2018. (2018年7月) [ref. 3]
- GeneReviews Japan: 遺伝性パラガングリオーマ・褐色細胞腫症候群. ver.2020.7.15
- ClinGen Actionability Reports: MAX, SDHA, SDHAF2, SDHB, SDHC, SDHD, TMEM127 Adult. ver.1.1.3 (2022.1.3)

監修：厚労科研小杉班・Actionability Working Group-Japan

編集： MONSTAR-SCREEN-2遺伝事務局

***SDHB*遺伝子について**

腫瘍*と関連する遺伝子の変化について*

- 一般的に腫瘍の一部は「生まれつきの遺伝子の変化」が原因といわれています。
  - 「生まれつきの遺伝子の変化」をもっている場合、腫瘍ができやすい体質をもっていると考えられます。
  - 腫瘍ができやすい体質は親、子、兄弟、姉妹などの血のつながった家族と共有している可能性があります。
  - 体質を知ることで、その情報をご自身やご家族の健康管理に役立てることができます。
  - 今回の遺伝子検査でみつかった変化が「生まれつきの遺伝子の変化」かどうかは、血液検査により確認できます。

*SDHB*遺伝子とは？

- 腫瘍を発症するリスクと関連があることが示されている遺伝子です。裏面の資料 (表) をご参照ください。

「生まれつきの遺伝子の変化」であるかどうかを知ることはどんなことに役立ちますか？

- 発症リスクが高い腫瘍を知り、それに対する検診を行うことで、早期発見につながる可能性があります。

家族にどんな影響がありますか？
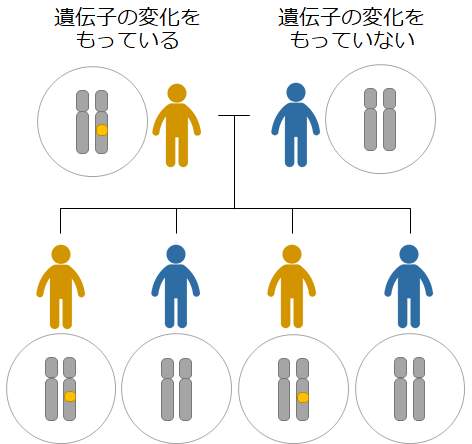


- 人は２つの*SDHB*遺伝子をそれぞれ両親から1つずつ受け継いでいます。
- *SDHB*遺伝子の変化を生まれつきもっていた場合、両親、兄弟、姉妹、子供が同じ変化をもっている確率は50%です。同じ変化をもっているかは、遺伝子検査で確認することができます。
- おば、おじ、いとこなども一定の確率で*同じ遺伝子の変化をもっている*可能性があります。
- 遺伝子の変化についてご家族の方と情報共有することは、ご家族の方の健康管理に役立つ可能性があります。

※生まれつきの*SDHB*遺伝子の変化は、両親いずれかから受け継いだものではなく、新規に起こった変化である可能性もあります。この場合のご家族への影響については、遺伝専門外来におたずねください。

遺伝カウンセリングについて

遺伝カウンセリングでは、生まれつきの遺伝子の変化と体質との関連、ご家族への影響などについてより詳しくご説明します。お気軽にご相談ください。　注意：遺伝学的検査の保険適用には条件があり、関連する症状を発症していないご家族の遺伝子検査や検診は自費となります。詳しくは担当者におたずね下さい。

＜参考資料表＞

|  | 一般的な日本人 | *SDHB*遺伝子に病気の原因となる変化を生まれつきもっている | | | |
| --- | --- | --- | --- | --- | --- |
|  | 生涯を通して  罹患する確率^1,2,3)^ | 生涯を通して  罹患する確率 | | 推奨される対応 | |
|  |  | 女性^3)^ | 男性^3)^ | 女性^3)^ | 男性^3)^ |
| パラガングリ  オーマ | 患者数：全国で  約1500人/年  ※米国データ：100万人に2人 | 30 ％ | | 1年ごとの血液検査(血中遊離メタネフリン)  2年ごとのCTまたはMRI検査  3年ごとのI-MIBGシンチグラフィ(画像診断) | |
| 副腎  褐色細胞腫 | 0.01~0.02 ％ |  |  |  |  |

※今回見つかった*SDHB遺伝子*の変化が生まれつきの変化であっても、必ず症状がみられるというわけではありません。

※上記に示す罹患する確率は、良性・境界性の腫瘍を含めた確率であり、悪性腫瘍はその一部です。*SDHB*遺伝子に変化をもっている方の腫瘍では34～97%の悪性化のリスクがあるといわれています^3)^。

※上表に示したほか、消化管の腫瘍が生じることがあります。詳細は遺伝専門外来におたずねください。

※記載は日本の診療ガイドライン^3)^を参照しています。詳細は各施設の遺伝専門外来におたずねください。

※表は2021年時点の記載です。今後、研究が進み推奨内容が変わる可能性があります。

【参考文献】

- 国立がん研究センター希少がんセンター: パラガングリオーマ <https://www.ncc.go.jp/jp/rcc/about/paraganguriouma/index.html> [ref. 1]
- National Cancer Institute Center for Cancer Research <https://www.cancer.gov/pediatric-adult-rare-tumor/rare-tumors/rare-endocrine-tumor/paraganglioma> [ref. 2]
- 日本内分泌学会. 褐色細胞腫・パラガングリオーマ診療ガイドライン2018. (2018年7月) [ref. 3]
- GeneReviews Japan: 遺伝性パラガングリオーマ・褐色細胞腫症候群. ver.2020.7.15
- ClinGen Actionability Reports: MAX, SDHA, SDHAF2, SDHB, SDHC, SDHD, TMEM127 Adult. ver.1.1.3 (2022.1.3)

監修：厚労科研小杉班・Actionability Working Group-Japan

編集： MONSTAR-SCREEN-2遺伝事務局

***SDHC*遺伝子について**

腫瘍*と関連する遺伝子の変化について*

- 一般的に腫瘍の一部は「生まれつきの遺伝子の変化」が原因といわれています。
  - 「生まれつきの遺伝子の変化」をもっている場合、腫瘍ができやすい体質をもっていると考えられます。
  - 腫瘍ができやすい体質は親、子、兄弟、姉妹などの血のつながった家族と共有している可能性があります。
  - 体質を知ることで、その情報をご自身やご家族の健康管理に役立てることができます。
  - 今回の遺伝子検査でみつかった変化が「生まれつきの遺伝子の変化」かどうかは、血液検査により確認できます。

*SDHC*遺伝子とは？

- 腫瘍を発症するリスクと関連があることが示されている遺伝子です。裏面の資料 (表) をご参照ください。

「生まれつきの遺伝子の変化」であるかどうかを知ることはどんなことに役立ちますか？

- 発症リスクが高い腫瘍を知り、それに対する検診を行うことで、早期発見につながる可能性があります。

家族にどんな影響がありますか？
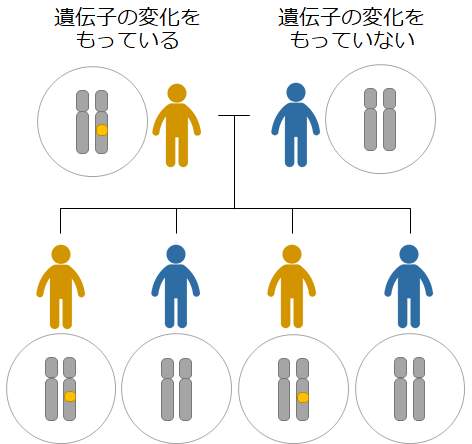


- 人は２つの*SDHC*遺伝子をそれぞれ両親から1つずつ受け継いでいます。
- *SDHC*遺伝子の変化を生まれつきもっていた場合、両親、兄弟、姉妹、子供が同じ変化をもっている確率は50%です。同じ変化をもっているかは、遺伝子検査で確認することができます。
- おば、おじ、いとこなども一定の確率で*同じ遺伝子の変化をもっている*可能性があります。
- 遺伝子の変化についてご家族の方と情報共有することは、ご家族の方の健康管理にも役立つ可能性があります。

※生まれつきの*SDHC*遺伝子の変化は、両親いずれかから受け継いだものではなく、新規に起こった変化である可能性もあります。この場合のご家族への影響については、遺伝専門外来におたずねください。

遺伝カウンセリングについて

遺伝カウンセリングでは、生まれつきの遺伝子の変化とがんとの関連、ご家族への影響などについてより詳しくご説明します。お気軽にご相談ください。　注意：遺伝学的検査の保険適用には条件があり、関連する症状を発症していないご家族の遺伝子検査や検診は自費となります。詳しくは担当者におたずね下さい。

＜参考資料表＞

|  | 一般的な日本人 | *SDHC*遺伝子に病気の原因となる変化を生まれつきもっている | | | |
| --- | --- | --- | --- | --- | --- |
|  | 生涯を通して  罹患する確率^1,2,3)^ | 生涯を通して  罹患する確率 | | 推奨される対応 | |
|  |  | 女性^3)^ | 男性^3)^ | 女性^3)^ | 男性^3)^ |
| パラガングリ  オーマ | 患者数：全国で  約1500人/年  ※米国データ：100万人に2人 | リスクが上がる  可能性 | | 1年ごとの血液検査(血中遊離メタネフリン)  2年ごとのCTまたはMRI検査  3年ごとのI-MIBGシンチグラフィ(画像検査) | |
| 副腎  褐色細胞腫 | 0.01~0.02 ％ | リスクが上がる  可能性 | |  |  |

※今回見つかった*SDHC遺伝子*の変化が生まれつきの変化であっても、必ず症状がみられるというわけではありません。

※上記に示す罹患する確率は、良性・境界性の腫瘍を含めた確率であり、悪性腫瘍はその一部です。

※上表に示したほか、消化管の腫瘍が生じることがあります。詳細は遺伝専門外来におたずねください。

※記載は日本の診療ガイドライン^3)^を参照しています。詳細は各施設の遺伝専門外来におたずねください。

※表は2021年時点の記載です。今後、研究が進み推奨内容が変わる可能性があります。

【参考文献】

- 国立がん研究センター希少がんセンター: パラガングリオーマ <https://www.ncc.go.jp/jp/rcc/about/paraganguriouma/index.html> [ref. 1]
- National Cancer Institute Center for Cancer Research <https://www.cancer.gov/pediatric-adult-rare-tumor/rare-tumors/rare-endocrine-tumor/paraganglioma> [ref. 2]
- 日本内分泌学会. 褐色細胞腫・パラガングリオーマ診療ガイドライン2018. (2018年7月) [ref. 3]
- GeneReviews Japan: 遺伝性パラガングリオーマ・褐色細胞腫症候群. ver.2020.7.15
- ClinGen Actionability Reports: MAX, SDHA, SDHAF2, SDHB, SDHC, SDHD, TMEM127 Adult. ver.1.1.3 (2022.1.3)

監修：厚労科研小杉班・Actionability Working Group-Japan

編集： MONSTAR-SCREEN-2遺伝事務局

***SDHD*遺伝子について**

腫瘍*と関連する遺伝子の変化について*

- 一般的に腫瘍の一部は「生まれつきの遺伝子の変化」が原因といわれています。
  - 「生まれつきの遺伝子の変化」をもっている場合、腫瘍ができやすい体質をもっていると考えられます。
  - 腫瘍ができやすい体質は親、子、兄弟、姉妹などの血のつながった家族と共有している可能性があります。
  - 体質を知ることで、その情報をご自身やご家族の健康管理に役立てることができます。
  - 今回の遺伝子検査でみつかった変化が「生まれつきの遺伝子の変化」かどうかは、血液検査により確認できます。

*SDHD*遺伝子とは？

- 腫瘍を発症するリスクと関連があることが示されている遺伝子です。裏面の資料 (表) をご参照ください。

「生まれつきの遺伝子の変化」であるかどうかを知ることはどんなことに役立ちますか？

- 発症リスクが高い腫瘍を知り、それに対する検診を行うことで、早期発見につながる可能性があります。

家族にどんな影響がありますか？
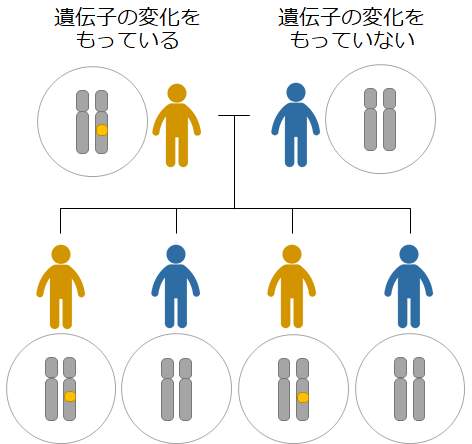


- 人は２つの*SDHD*遺伝子をそれぞれ両親から1つずつ受け継いでいます。
- *SDHD*遺伝子の変化を生まれつきもっていた場合、両親、兄弟、姉妹、子供が同じ変化をもっている確率は50%です。同じ変化をもっているかは、遺伝子検査で確認することができます。
- おば、おじ、いとこなども一定の確率で*同じ遺伝子の変化をもっている*可能性があります。
- 遺伝子の変化についてご家族の方と情報共有することは、ご家族の方の健康管理に役立つ可能性があります。

※生まれつきの*SDHD*遺伝子の変化は、両親いずれかから受け継いだものではなく、新規に起こった変化である可能性もあります。この場合のご家族への影響については、遺伝専門外来におたずねください。

遺伝カウンセリングについて

遺伝カウンセリングでは、生まれつきの遺伝子の変化と体質との関連、ご家族への影響などについてより詳しくご説明します。お気軽にご相談ください。　注意：遺伝学的検査の保険適用には条件があり、関連する症状を発症していないご家族の遺伝子検査や検診は自費となります。詳しくは担当者におたずね下さい。

＜参考資料表＞

|  | 一般的な日本人 | *SDHD*遺伝子に病気の原因となる変化を生まれつき持っている | | | |
| --- | --- | --- | --- | --- | --- |
|  | 生涯を通して  罹患する確率^1,2,3)^ | 生涯を通して  罹患する確率 | | 推奨される対応 | |
|  |  | 女性^3)^ | 男性^3)^ | 女性^3)^ | 男性^3)^ |
| パラガングリ  オーマ | 患者数：全国で  約1500人/年  ※米国データ：100万人に2人 | リスクが上がる  可能性 | | 1年ごとの血液検査(血中遊離メタネフリン)  2年ごとのCTまたはMRI検査  3年ごとのI-MIBGシンチグラフィ | |
| 副腎  褐色細胞腫 | 0.01~0.02 ％ | リスクが上がる  可能性 | |  |  |

※今回見つかった*SDHD遺伝子*の変化が生まれつきの変化であっても、必ず症状がみられるというわけではありません。

※上記に示す罹患する確率は、良性・境界性の腫瘍を含めた確率であり、悪性腫瘍はその一部です。

※上表に示したほか、消化管の腫瘍が生じることがあります。詳細は遺伝専門外来におたずねください。

※記載は日本の診療ガイドライン^3)^を参照しています。詳細については各施設の遺伝専門外来におたずねください。

※表は2021年時点の記載です。今後、研究が進み推奨内容が変わる可能性があります。

【参考文献】

- 国立がん研究センター希少がんセンター: パラガングリオーマ <https://www.ncc.go.jp/jp/rcc/about/paraganguriouma/index.html> [ref. 1]
- National Cancer Institute Center for Cancer Research <https://www.cancer.gov/pediatric-adult-rare-tumor/rare-tumors/rare-endocrine-tumor/paraganglioma> [ref. 2]
- 日本内分泌学会. 褐色細胞腫・パラガングリオーマ診療ガイドライン2018. (2018年7月) [ref. 3]
- GeneReviews Japan: 遺伝性パラガングリオーマ・褐色細胞腫症候群. ver.2020.7.15
- ClinGen Actionability Reports: MAX, SDHA, SDHAF2, SDHB, SDHC, SDHD, TMEM127 Adult. ver.1.1.3 (2022.1.3)

監修：厚労科研小杉班・Actionability Working Group-Japan

編集： MONSTAR-SCREEN-2遺伝事務局

***SMAD3*遺伝子について**

*SMAD3*遺伝子とは？

- 心疾患のリスクと関連があることが示されている遺伝子です。
- *SMAD3遺伝子*の生まれつきの変化は**ロイス・ディーツ症候群**の原因となることが知られています。裏面の資料 (表) をご参照ください。
- 今回の遺伝子検査でみつかった変化が「生まれつきの遺伝子の変化」かどうかは、血液検査により確認できます。

「生まれつきの遺伝子の変化」であるかどうかを知ることはどんなことに役立ちますか？

- 発症リスクが高い症状を知り、それに対する検診を行うことで、早期発見につながる可能性があります。

家族にどんな影響がありますか？
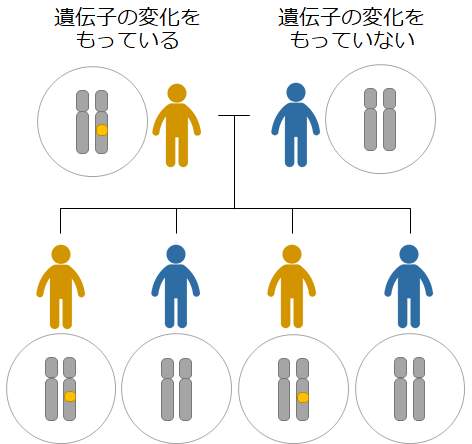


- 人は２つの*SMAD3*遺伝子をそれぞれ両親から1つずつ受け継いでいます。
- *SMAD3*遺伝子の変化を生まれつきもっていた場合、両親、兄弟、姉妹、子供が同じ変化をもっている確率は50%です。同じ変化をもっているかは、遺伝子検査で確認することができます。
- おば、おじ、いとこなども一定の確率で*同じ遺伝子の変化をもっている*可能性があります。
- 遺伝子の変化についてご家族の方と情報共有することは、ご家族の方の健康管理にも役立つ可能性があります。

※生まれつきの*SMAD3*遺伝子の変化は、両親いずれかから受け継いだものではなく、新規に起こった変化である可能性が約75%^2)^ (注意：ロイス・ディーツ症候群として) といわれています。この場合のご家族への影響については、遺伝専門外来におたずねください。

遺伝カウンセリングについて

遺伝カウンセリングでは、生まれつきの遺伝子の変化と体質との関連、ご家族への影響などについてより詳しくご説明します。お気軽にご相談ください。　注意：遺伝学的検査の保険適用には条件があり、関連する症状を発症していないご家族の遺伝子検査や検診は自費となります。詳しくは担当者におたずね下さい。

＜参考資料表＞

|  | 一般的な日本人 | *SMAD3*遺伝子に病気の原因となる変化を生まれつきもっている | |
| --- | --- | --- | --- |
|  | 発症する確率^1)^ | 生涯を通して  罹患する確率^2)^ | 推奨される対応^1)^ |
| 大動脈瘤・解離 | 10万人あたり  年間3~10人発症 | 95% | 心血管超音波検査、画像検査 (MRA, CTA)  大動脈基部拡張が認められた場合は大動脈基部置換術を検討  アンジオテンシンⅡ受容体拮抗薬 (ARB) あるいはβ遮断薬による降圧剤治療 |

- 他に骨格や皮膚に特徴的な症状が見られることがあります。

※*SMAD3遺伝子*の変化が生まれつきの変化であっても、必ず症状がみられるというわけではありません。

※記載は日本の診療ガイドライン^1)^を参照しています。詳細については各施設の専門診療科あるいは遺伝専門外来におたずねください。

【参考文献】

- 日本循環器学会・日本心臓血管外科学会・日本胸部外科学会・日本血管外科学会合同ガイドライン. 大動脈瘤・大動脈解離診療ガイドライン 2020年改訂版. (2020年7月) [ref. 1]
- GeneReviews Japan: ロイス・ディーツ症候群. ver.2021.4.30 [ref. 2]
- ClinGen Actionability Reports: SMAD3, TGFB2, TGFB3, TGFBR1, TGFBR2 Adult. ver.3.0.4 (2021.8.24)

監修：厚労科研小杉班・Actionability Working Group-Japan

編集： MONSTAR-SCREEN-2遺伝事務局

***SMAD4*遺伝子について**

*がんの発症と関連する遺伝子の変化について*

- - 一般的にがんの5~10%は「生まれつきの遺伝子の変化」が原因といわれています。
  - 「生まれつきの遺伝子の変化」をもっている場合、がんになりやすい体質をもっていると考えられます。
  - がんになりやすい体質は親、子、兄弟、姉妹 などの血のつながった家族と共有している可能性があります。
  - 体質を知ることで、その情報をご自身やご家族の健康管理に役立てることができます。
  - 今回の遺伝子検査でみつかった変化が「生まれつきの遺伝子の変化」かどうかは、血液検査により確認できます。

*SMAD4*遺伝子とは？

- *SMAD4遺伝子*の生まれつきの変化は**若年性ポリポーシス症候群**の原因となることが知られています。裏面の資料 (表) をご参照ください。

「生まれつきの遺伝子の変化」であるかどうかを知ることはどんなことに役立ちますか？

- 発症リスクが高いがんを知り、それに対する検診を行うことで、早期発見につながる可能性があります。

家族にどんな影響がありますか？
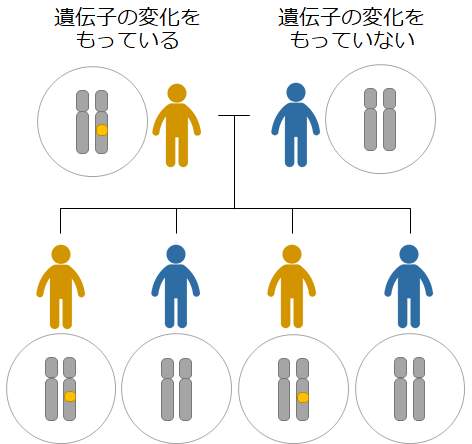


- 人は２つの*SMAD4*遺伝子をそれぞれ両親から1つずつ受け継いでいます。
- *SMAD4*遺伝子の変化を生まれつきもっていた場合、両親、兄弟、姉妹、子どもが同じ変化をもっている確率は50%です。同じ変化をもっているかは、遺伝子検査で確認することができます。
- おば、おじ、いとこなども一定の確率で*同じ遺伝子の変化をもっている*可能性があります。
- 遺伝子の変化についてご家族の方と情報共有することは、ご家族の方の健康管理に役立つ可能性があります。

※生まれつきの*SMAD4*遺伝子の変化は、両親いずれかから受け継いだものではなく、新規に起こった変化である可能性もあります。この場合のご家族への影響については、遺伝専門外来におたずねください。

遺伝カウンセリングについて

遺伝カウンセリングでは、生まれつきの遺伝子の変化と体質との関連、ご家族への影響などについてより詳しくご説明します。お気軽にご相談ください。　注意：遺伝学的検査の保険適用には条件があり、関連する症状を発症していないご家族の遺伝子検査や検診は自費となります。詳しくは担当者におたずね下さい。

＜参考資料表＞

|  | 一般的な日本人 | *SMAD4*遺伝子に病気の原因となる変化を生まれつきもっている | | | |
| --- | --- | --- | --- | --- | --- |
|  | 生涯を通して  罹患する確率^1,2)^ | 生涯を通して  罹患する確率 | | 推奨される対応 | |
|  |  | 女性^3,4)^ | 男性^3,4)^ | 女性^3,5)^ | 男性^3,5)^ |
| 大腸ポリープ(がん化するリスクあり) | (大腸がん)  10.2 ％ (男性)  8.0 ％ (女性) | (大腸ポリープ) 97 %  (大腸がん) ≦50 % | | 上部・下部消化管内視鏡検査  1~3 年おきに検査を行う | |
| 胃ポリープ  (がん化するリスクあり) | (胃がん)  10.3 ％ (男性)  4.7 ％ (女性) | (胃ポリープ) 68 %  (胃がん) ≦21 % | |  |  |
| 小腸ポリープ | (小腸がん)  0.2 % (男性)  0.1 % (女性) | がん化のリスクは低い | |  |  |

- ほとんどの若年性ポリポーシス症候群の患者さんは20歳までに何個かのポリープを認めることがわかっています。
- がん以外にも、心血管系に異常がみられることがあります。詳細は専門診療科あるいは遺伝専門外来におたずねください。

※*SMAD4遺伝子*の変化が生まれつきの変化であっても、必ずがんを発症するというわけではありません。

※遺伝子の変化をもっていた場合の罹患する確率、推奨される対応の記載は日本の診療ガイドライン^3)^ および米国のガイドライン (NCCNガイドライン)^5)^を参照しています。推奨事項については、各医療機関において必ずしも実施されているとは限りませんので、詳細については各施設の専門診療科あるいは遺伝専門外来におたずねください。

※表は2021年時点の記載です。今後、研究が進み推奨内容が変わる可能性があります。

【参考文献】

- 国立がん研究センターがん情報サービス「累積がん罹患リスク (2018年データ)」<https://ganjoho.jp/reg_stat/statistics/stat/summary.html> [ref. 1]
- 厚生労働省健康局がん・疾病対策課. 平成30年全国がん登録 罹患数・率報告 2018 [ref. 2]
- 小児・成人のための若年性ポリポーシス 症候群診療ガイドライン 2020年版. 遺伝性腫瘍, 2020; 20(2): 79. [ref. 3]
- Appreciating the broad clinical features of SMAD4 mutation carriers: multicenter chart review. Genet Med, 2014; 16(8): 588. [ref. 4]
- NCCN Guidelines® Genetic/Familial High-Risk Assessment: Colorectal. ver.1.2021 (2021.5.11) [ref. 5]
- GeneReviews Japan: 若年性ポリポーシス症候群. ver.2014.3.3.
- ClinGen Actionability Reports: SMAD4, BMPR1A Adult. ver.1.1.1 (2021.1.19)

監修：厚労科研小杉班・Actionability Working Group-Japan

編集： MONSTAR-SCREEN-2遺伝事務局

***SMARCB1*遺伝子について**

*腫瘍と関連する遺伝子の変化について*

- 一般的に腫瘍の一部は「生まれつきの遺伝子の変化」が原因といわれています。
  - 「生まれつきの遺伝子の変化」をもっている場合、生まれつき腫瘍ができやすい体質をもっていると考えられます。
  - 生まれつき腫瘍ができやすい体質は親、子、兄弟、姉妹などの血のつながった家族と共有している可能性があります。
  - 体質を知ることで、その情報をご自身やご家族の健康管理に役立てることができます。
  - 今回の遺伝子検査でみつかった変化が「生まれつきの遺伝子の変化」かどうかは、血液検査により確認できます。

*SMARCB1*遺伝子とは？

- 小児期に腫瘍が発生する**ラブドイド腫瘍好発症候群１型** (Rhabdoid tumor predisposition syndrome 1, RTPS1) のリスクと関連があることが示されている遺伝子です。裏面の資料 (表) をご参照ください。

「生まれつきの遺伝子の変化」であるかどうかを知ることはどんなことに役立ちますか？

- 発症リスクが高い腫瘍を知り、それに対する検診を行うことで、早期発見につながる可能性があります。

家族にどんな影響がありますか？
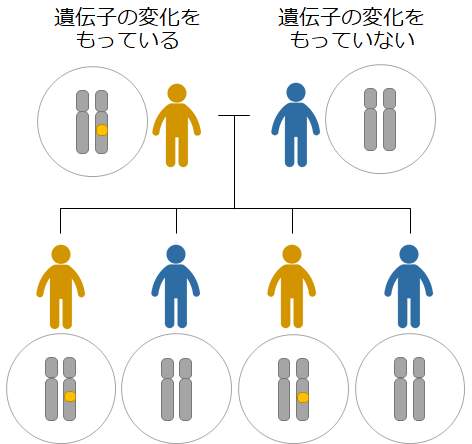


- 人は２つの*SMARCB1*遺伝子をそれぞれ両親から1つずつ受け継いでいます。
- *SMARCB1*遺伝子の変化を生まれつきもっていた場合、両親、兄弟、姉妹、子供が同じ変化をもっている確率は50%です。同じ変化をもっているかは、遺伝子検査で確認することができます。
- おば、おじ、いとこなども一定の確率で*同じ遺伝子の変化をもっている*可能性があります。
- 遺伝子の変化についてご家族の方と情報共有することは、ご家族の方の健康管理にも役立つ可能性があります。

※多くの場合、生まれつきの*SMARCB1*遺伝子の変化は、両親いずれかから受け継いだものではなく、新規に起こった変化であるといわれています^1,2)^。この場合のご家族への影響については、遺伝専門外来におたずねください。

遺伝カウンセリングについて

遺伝カウンセリングでは、生まれつきの遺伝子の変化と体質との関連、ご家族への影響などについてより詳しくご説明します。お気軽にご相談ください。　注意：遺伝学的検査の保険適用には条件があり、関連する症状を発症していないご家族の遺伝子検査や検診は自費となります。詳しくは担当者におたずね下さい。

＜参考資料表＞

|  | 一般的な日本人 | *SMARCB1*遺伝子に病気の原因となる変化を生まれつきもっている | | | |
| --- | --- | --- | --- | --- | --- |
|  | 生涯を通して  罹患する確率^2)^ | 罹患する確率 | | 推奨される対応 | |
|  |  | 女性^2)^ | 男性^2)^ | 女性^2)^ | 男性^2)^ |
| 脳腫瘍  (ラブドイド腫瘍) | 1万人に1~3人/年 | データなし | | 出生後～5歳まで3か月ごとの脳MRI検査（注） | |
| 腎腫瘍  (ラブドイド腫瘍) | 15歳以下の子ども  1万人に1~2人/年 | データなし | | 出生後～5歳まで3か月ごとの腹部超音波(注) | |
| 軟骨肉腫  (ラブドイド腫瘍) | データなし | データなし | | 出生後～5歳まで全身MRI検査 (注) | |

(注) 記載の推奨事項については、本邦での具体的な検診方法は示されていません。詳細は各施設の遺伝専門外来におたずねください。

※*SMARCB1遺伝子*の変化が生まれつきの変化であっても、必ず症状がみられるというわけではありません。

※表は2021年時点の記載です。今後、研究が進み推奨内容が変わる可能性があります。

【参考文献】

- GeneReviews: Rhabdoid Tumor Predisposition Syndrome. ver.2017.12.7 [ref. 1]
- ClinGen Actionability Reports: SMARCA4, SMARCB1 Adult. ver.1.0.0 (2021.8.2) [ref. 2]

監修：厚労科研小杉班・Actionability Working Group-Japan

編集： MONSTAR-SCREEN-2遺伝事務局

***STK11*遺伝子について**

*がんの発症と関連する遺伝子の変化について*

- - 一般的にがんの5~10%は「生まれつきの遺伝子の変化」が原因といわれています。
  - 「生まれつきの遺伝子の変化」をもっている場合、がんになりやすい体質をもっていると考えられます。
  - がんになりやすい体質は親、子、兄弟、姉妹 などの血のつながった家族と共有している可能性があります。
  - 体質を知ることで、その情報をご自身やご家族の健康管理に役立てることができます。
  - 今回の遺伝子検査でみつかった変化が「生まれつきの遺伝子の変化」かどうかは、血液検査により確認できます。

*STK11*遺伝子とは？

- *STK11遺伝子*の生まれつきの変化は**ポイツ・ジェガース症候群**の原因となることが知られています。裏面の資料 (表) をご参照ください。

「生まれつきの遺伝子の変化」であるかどうかを知ることはどんなことに役立ちますか？

- 発症リスクが高いがんを知り、それに対する検診を行うことで、早期発見につながる可能性があります。


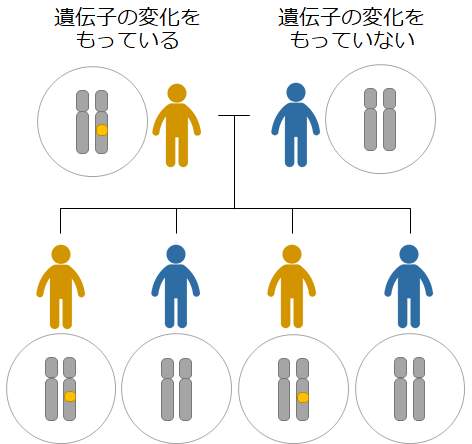
家族にどんな影響がありますか？

- 人は２つの*STK11*遺伝子をそれぞれ両親から1つずつ受け継いでいます。
- *STK11*遺伝子の変化を生まれつきもっていた場合、両親、兄弟、姉妹、子どもが同じ変化をもっている確率は50%です。同じ変化をもっているかは、遺伝子検査で確認することができます。
- おば、おじ、いとこなども一定の確率で*同じ遺伝子の変化をもっている*可能性があります。
- 遺伝子の変化についてご家族の方と情報共有することは、ご家族の方の健康管理に役立つ可能性があります。

※生まれつきの*STK11*遺伝子の変化は、両親から受け継いだものではなく、新規に起こった変化である可能性もあります。この場合のご家族への影響については、遺伝専門外来におたずねください。

遺伝カウンセリングについて

遺伝カウンセリングでは、生まれつきの遺伝子の変化と体質との関連、ご家族への影響などについてより詳しくご説明します。お気軽にご相談ください。　※遺伝学的検査の保険適用には条件があり、関連する症状を発症していない家族の遺伝子検査や検診は自費となります。詳細は担当者におたずね下さい。

＜参考資料表＞

|  | 一般的な日本人 | *STK11*遺伝子に病気の原因となる変化を生まれつきもっている | | | |
| --- | --- | --- | --- | --- | --- |
|  | 生涯を通して  罹患する確率^1,2)^ | 生涯を通して  罹患する確率 | | 推奨される対応 | |
|  |  | 女性^3,4)^ | 男性^3,4)^ | 女性^3,4)^ | 男性^3,4)^ |
| 大腸ポリープ/がん | (大腸がん)  10.2 ％ (男性)  8.0 ％ (女性) | (ポリープ) 24~27 %  (がん) 36.4 % | | 上部消化管、全大腸内視鏡検査および  小腸カプセル内視鏡検査  18 歳以降：3 年おきに検査を行う  50歳以降：1~2 年おきに検査を行う | |
| 胃ポリープ/  がん | (胃がん)  10.3 ％ (男性)  4.7 ％ (女性) | (ポリープ) 24 %  (がん) 24 % | |  |  |
| 小腸ポリープ/がん | (小腸がん)  0.2 ％ (男性)  0.1 ％ (女性) | (ポリープ) 96 %  （がん）13.8 % | |  |  |
| 乳がん | 10.9 ％ (女性) | 19.3 % | ― | 18歳以降：自己検診 (年１回)  25歳以降：乳房MRI/超音波 (年１回)  50歳以降：マンモグラフィー (年１回) | ― |
| 子宮頸がん | 1.3 % (女性) | (子宮がん)  46.5% | ― | 18~25歳以降：1~3年おきに子宮頸部細胞診 | ― |
| 子宮内膜がん | 2.0% (女性) |  | ― | 18~25歳以降：内診/経腟超音波検査 (年１回) | ― |
| 卵巣がん | 1.6% (女性) | 10.1 % | ― |  |  |
| 膵がん | 2.6 ％ (男性)  2.6 ％ (女性) | 29.4 % | | 30歳以降：1~2 年おきにMRCP  (MR胆管膵管撮影) /EUS (超音波内視鏡) | |
| 精巣腫瘍 | 0.3% (男性) | ― | 9 % | 誕生～成年：触診/超音波 (触診で異常がみられた場合) (年１回) | |
| 肺がん | 9.9 ％ (男性)  4.9 ％ (女性) | 7.6 % | | 一般的な検診より早期の開始を考慮 | |

※*STK11遺伝子*の変化が生まれつきの変化であっても、必ずがんを発症するというわけではありません。

※遺伝子の変化をもっていた場合の罹患率、推奨される対応は日本の診療ガイドライン^3)^および米国のガイドライン^4)^ を参照しています。検診内容がすべて実施できるとは限りませんので、詳細は各施設の専門診療科あるいは遺伝専門外来におたずねください。

※表は2021年時点の記載です。今後、研究が進み推奨内容が変わる可能性があります。

【参考文献】

- 国立がん研究センターがん情報サービス「累積がん罹患リスク (2018年データ)」 <https://ganjoho.jp/reg_stat/statistics/stat/summary.html> [ref. 1]
- 厚生労働省健康局がん・疾病対策課. 平成30年全国がん登録 罹患数・率報告 2018 [ref. 2]
- 小児・成人のためのPeutz-Jeghers症候群診療ガイドライン 2020年版. 遺伝性腫瘍, 2020; 20(2): 59. [ref. 3]
- NCCN Guidelines® Genetic/Familial High-Risk Assessment: Colorectal. ver.1.2021 (2021.5.11) [ref. 4]
- GeneReviews Japan: Peutz-Jeghers症候群. ver.2021.11.9 [ref. 5]
- ClinGen Actionability Reports: STK11 Adult. ver.2.0.1 (2021.3.29) [ref. 6]

監修：厚労科研小杉班・Actionability Working Group-Japan

編集： MONSTAR-SCREEN-2遺伝事務局

***TERF2IP*遺伝子について**

*がんの発症と関連する遺伝子の変化について*

- - 一般的にがんの5~10%は「生まれつきの遺伝子の変化」が原因といわれています。
  - 「生まれつきの遺伝子の変化」をもっている場合、がんになりやすい体質をもっていると考えられます。
  - がんになりやすい体質は親、子、兄弟、姉妹 などの血のつながった家族と共有している可能性があります。
  - 体質を知ることで、その情報をご自身やご家族の健康管理に役立てることができます。
  - 今回の遺伝子検査でみつかった変化が「生まれつきの遺伝子の変化」かどうかは、血液検査により確認できます。

*TERF2IP*遺伝子とは？

- がんを発症するリスクと関連があることが示されている遺伝子です^1,2)^。裏面の資料 (表) をご参照ください。

「生まれつきの遺伝子の変化」であるかどうかを知ることはどんなことに役立ちますか？

- 発症リスクが高いがんを知り、それに対する検診を行うことで、早期発見につながる可能性があります。


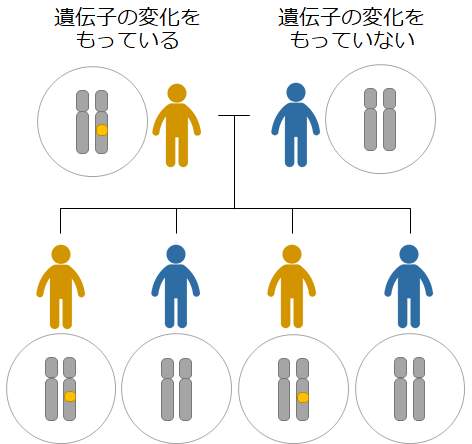


家族にどんな影響がありますか？

- 人は２つの*TERF2IP*遺伝子をそれぞれ両親から1つずつ受け継いでいます。
- *TERF2IP*遺伝子の変化を生まれつきもっていた場合、両親、兄弟、姉妹、子どもが同じ変化をもっている確率は50%です。同じ変化をもっているかは、遺伝子検査で確認することができます。
- おば、おじ、いとこなども一定の確率で*同じ遺伝子の変化をもっている*可能性があります。
- 遺伝子の変化についてご家族の方と情報共有することは、ご家族の方の健康管理にも役立つ可能性があります。

※生まれつきの*TERF2IP*遺伝子の変化は、両親いずれかから受け継いだものではなく、新規に起こった変化である可能性もあります。この場合のご家族への影響については、遺伝専門外来におたずねください。

遺伝カウンセリングについて

遺伝カウンセリングでは、生まれつきの遺伝子の変化と体質との関連、ご家族への影響などについてより詳しくご説明します。お気軽にご相談ください。　注意：遺伝学的検査の保険適用には条件があり、関連する症状を発症していないご家族の遺伝子検査や検診は自費となります。詳しくは担当者におたずね下さい。

＜参考資料表＞

|  | 一般的な日本人 | *TERF2IP*遺伝子に病気の原因となる変化を生まれつきもっている | | | |
| --- | --- | --- | --- | --- | --- |
|  | 生涯を通して罹患する確率^3)^ | 生涯を通して  罹患する確率 | | 推奨される対応 | |
|  |  | 女性^1)^ | 男性^1)^ | 女性^4)^ | 男性^4)^ |
| 悪性黒色腫  (注1) | 0.1 % (男性)  0.1 % (女性) | リスクが高まる可能性が  示唆されています。 | | 皮膚、頭皮、口腔粘膜、生殖器の皮膚科での精密検査 (半年に1回、母斑が安定している場合は年１回) (注2)  月１回の母斑自己検診 (注2) | |

(注1) 悪性黒色腫の罹病率 (年) は、白人が 24.3 人/10 万人、アジア人が 1.7 人/10 万人との報告があり^5)^、人種、地域、その他の遺伝的要因によってリスクは異なると考えられています。

(注2) 記載の推奨事項については、本邦での具体的な検診方法は示されていません。詳細は各施設の遺伝専門外来におたずねください

※*TERF2IP遺伝子*の変化が生まれつきの変化であっても、必ずがんを発症するというわけではありません。

※遺伝子の変化をもっていた場合の罹患する確率、推奨される対応の記載は海外の資料^1,4)^ を参照しています。

※表は2021年時点の記載です。今後、研究が進み推奨内容が変わる可能性があります。

【参考文献】

- *CDKN2A* genetic testing in melanoma-prone families in Sweden in the years 2015-2020: implications for novel national recommendations. Acta Oncologica, 2021; 60(7): 888. [ref. 1]
- Guidelines for reporting secondary findings of genome sequencing in cancer genes: the SFMPP recommendations. Eur J Hum Genet, 2018; 26(12): 1732. [ref. 2]
- 厚生労働省健康局がん・疾病対策課. 平成30年全国がん登録 罹患数・率報告 2018 [ref. 3]
- ClinGen Actionability Reports: CDKN2A Adult. ver.1.2.1 (2020.8.19) [ref. 4]
- 日本皮膚科学会. 皮膚悪性腫瘍ガイドライン第 3 版 メラノーマ診療ガイドライン 2019. 日皮会誌, 2019; 129(9):1759. [ref. 5]

監修：厚労科研小杉班・Actionability Working Group-Japan

編集： MONSTAR-SCREEN-2遺伝事務局

***TERT*遺伝子について**

**この遺伝子に、「生まれつきの遺伝子の変化」が見つかった場合には、その病的意義の解釈について症例ごとに検討する必要があります。まずは研究事務局にご相談ください。**

*がんの発症と関連する遺伝子の変化について*

- - 一般的にがんの5~10%は「生まれつきの遺伝子の変化」が原因といわれています。
  - 「生まれつきの遺伝子の変化」をもっている場合、がんになりやすい体質をもっていると考えられます。
  - がんになりやすい体質は親、子、兄弟、姉妹 などの血のつながった家族と共有している可能性があります。
  - 体質を知ることで、その情報をご自身やご家族の健康管理に役立てることができます。
  - 今回の遺伝子検査でみつかった変化が「生まれつきの遺伝子の変化」かどうかは、血液検査により確認できます。

*TERT*遺伝子とは？

- *TERT*遺伝子の生まれつきの変化は先天性角化不全症 (Dyskeratosis congenita: DC) の原因となることが知られています^1,2)^。
- DCは爪の萎縮、口腔内白斑、皮膚色素沈着を３徴とする先天性造血不全症候群です。
- DC患者では、悪性腫瘍の罹患率が健常人に比較して 11 倍とされています (肺や頭頚部の扁平上皮癌、消化管の腺癌、骨髄異形成症候群、骨髄性白血病など)。

遺伝カウンセリングについて

遺伝カウンセリングでは、生まれつきの遺伝子の変化と体質との関連、ご家族への影響などについてより詳しくご説明します。お気軽にご相談ください。

【参考文献】

- 厚生労働科学研究費補助金　難治性疾患等政策研究事業　特発性造血障害に関する調査研究班. 先天性角化不全症診療の参照ガイド 令和 1 年改訂版. (2019年12月) [ref. 1]
- GeneReviews®: Dyskeratosis Congenita. ver.2019.11.21 [ref. 2]

監修：厚労科研小杉班・Actionability Working Group-Japan

編集： MONSTAR-SCREEN-2遺伝事務局

***TGFBR1*遺伝子について**

*TGFBR1*遺伝子とは？

- 心疾患のリスクと関連があることが示されている遺伝子です。
- *TGFBR1遺伝子*の生まれつきの変化は**ロイス・ディーツ症候群**の原因となることが知られています。裏面の資料 (表) をご参照ください。
- 今回の遺伝子検査でみつかった変化が「生まれつきの遺伝子の変化」かどうかは、血液検査により確認できます。

「生まれつきの遺伝子の変化」であるかどうかを知ることはどんなことに役立ちますか？

- 発症リスクが高い症状を知り、それに対する検診を行うことで、早期発見につながる可能性があります。

家族にどんな影響がありますか？
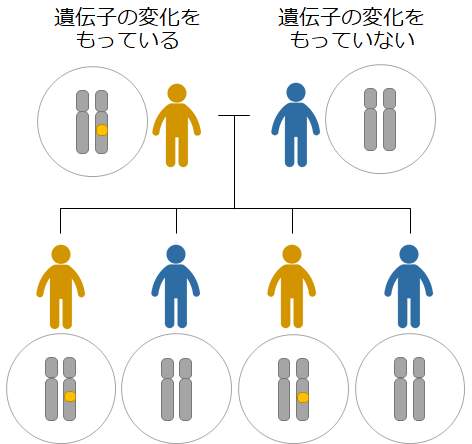


- 人は２つの*TGFBR1*遺伝子をそれぞれ両親から1つずつ受け継いでいます。
- *TGFBR1*遺伝子の変化を生まれつきもっていた場合、両親、兄弟、姉妹、子供が同じ変化をもっている確率は50%です。同じ変化をもっているかは、遺伝子検査で確認することができます。
- おば、おじ、いとこなども一定の確率で*同じ遺伝子の変化をもっている*可能性があります。
- 遺伝子の変化についてご家族の方と情報共有することは、ご家族の方の健康管理に役立つ可能性があります。

※生まれつきの*TGFR1*遺伝子の変化は、両親いずれかから受け継いだものではなく、新規に起こった変化である可能性が約75%^2)^ (注意：ロイス・ディーツ症候群として) といわれています。この場合のご家族への影響については、遺伝専門外来におたずねください。

遺伝カウンセリングについて

遺伝カウンセリングでは、生まれつきの遺伝子の変化とがんとの関連、ご家族への影響などについてより詳しくご説明します。お気軽にご相談ください。　注意：遺伝学的検査の保険適用には条件があり、関連する症状を発症していないご家族の遺伝子検査や検診は自費となる可能性があります。詳しくは担当者におたずね下さい。

＜参考資料表＞

|  | 一般的な日本人 | *TGFBR1*遺伝子に病気の原因となる変化を生まれつき持っている | |
| --- | --- | --- | --- |
|  | 発症する確率^1)^ | 生涯を通して  罹患する確率^2)^ | 推奨される対応^1)^ |
| 大動脈瘤・解離 | 10万人あたり  年間3~10人発症 | 95% | 心血管超音波検査、画像検査 (MRA, CTA)  大動脈基部拡張が認められた場合は大動脈基部置換術を検討  アンジオテンシンⅡ受容体拮抗薬 (ARB) あるいはβ遮断薬による降圧剤治療 |

- 他に骨格や皮膚に特徴的な症状が見られることがあります。

※*TGFBR1遺伝子*の変化が生まれつきの変化であっても、必ず症状がみられるというわけではありません。

※記載は日本の診療ガイドライン^1)^を参照しています。詳細については各施設の専門診療科あるいは遺伝専門外来におたずねください。

【参考文献】

- 日本循環器学会・日本心臓血管外科学会・日本胸部外科学会・日本血管外科学会合同ガイドライン. 大動脈瘤・大動脈解離診療ガイドライン 2020年改訂版. (2020年7月) [ref. 1]
- GeneReviews Japan: ロイス・ディーツ症候群. ver.2021.4.30 [ref. 2]
- ClinGen Actionability Reports: SMAD3, TGFB2, TGFB3, TGFBR1, TGFBR2 Adult. ver.3.0.4 (2021.8.24)

監修：厚労科研小杉班・Actionability Working Group-Japan

編集： MONSTAR-SCREEN-2遺伝事務局

***TGFBR2*遺伝子について**

*TGFBR2*遺伝子とは？

- 心疾患のリスクと関連があることが示されている遺伝子です。
  - *TGFBR2遺伝子*の生まれつきの変化は**ロイス・ディーツ症候群**の原因となることが知られています。裏面の資料 (表) をご参照ください。
  - 今回の遺伝子検査でみつかった変化が「生まれつきの遺伝子の変化」かどうかは、血液検査により確認できます。

「生まれつきの遺伝子の変化」であるかどうかを知ることはどんなことに役立ちますか？

- 発症リスクが高い症状を知り、それに対する検診を行うことで、早期発見につながる可能性があります。

家族にどんな影響がありますか？
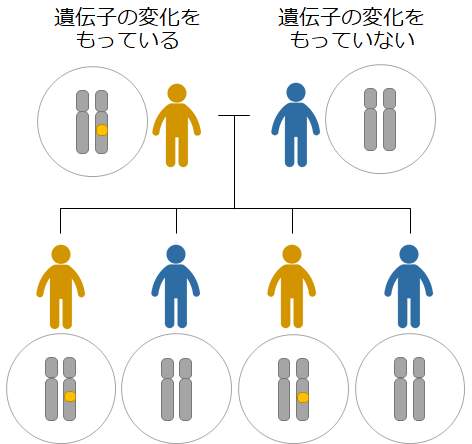


- 人は２つの*TGFBR2*遺伝子をそれぞれ両親から1つずつ受け継いでいます。
- *TGFBR2*遺伝子の変化を生まれつきもっていた場合、両親、兄弟、姉妹、子供が同じ変化をもっている確率は50%です。同じ変化をもっているかは、遺伝子検査で確認することができます。
- おば、おじ、いとこなども一定の確率で*同じ遺伝子の変化をもっている*可能性があります。
- 遺伝子の変化についてご家族の方と情報共有することは、ご家族の方の健康管理に役立つ可能性があります。

※生まれつきの*TGFR2*遺伝子の変化は、両親いずれかから受け継いだものではなく、新規に起こった変化である可能性が約75%^2)^ (注意：ロイス・ディーツ症候群として) といわれています。この場合のご家族への影響については、遺伝専門外来にておたずねください。

遺伝カウンセリングについて

遺伝カウンセリングでは、生まれつきの遺伝子の変化と体質との関連、ご家族への影響などについてより詳しくご説明します。お気軽にご相談ください。　注意：遺伝学的検査の保険適用には条件があり、関連する症状を発症していないご家族の遺伝子検査や検診は自費となる可能性があります。詳しくは担当者におたずね下さい。

＜参考資料表＞

|  | 一般的な日本人 | *TGFBR2*遺伝子に病気の原因となる変化を生まれつきもっている | |
| --- | --- | --- | --- |
|  | 発症する確率^1)^ | 生涯を通して  罹患する確率^2)^ | 推奨される対応^1)^ |
| 大動脈瘤・解離 | 10万人あたり  年間3~10人発症 | 95% | 心血管超音波検査、画像検査 (MRA, CTA)  大動脈基部拡張が認められた場合は大動脈基部置換術を検討  アンジオテンシンⅡ受容体拮抗薬 (ARB) あるいはβ遮断薬による降圧剤治療 |

- 他に骨格や皮膚に特徴的な症状が見られることがあります。

※*TGFBR2遺伝子*の変化が生まれつきの変化であっても、必ず症状がみられるというわけではありません。

※記載は日本の診療ガイドライン^1)^を参照しています。詳細については各施設の専門診療科あるいは遺伝専門外来におたずねください。

【参考文献】

- 日本循環器学会・日本心臓血管外科学会・日本胸部外科学会・日本血管外科学会合同ガイドライン. 大動脈瘤・大動脈解離診療ガイドライン 2020年改訂版. (2020年7月) [ref. 1]
- GeneReviews Japan: ロイス・ディーツ症候群. ver.2021.4.30 [ref. 2]
- ClinGen Actionability Reports: SMAD3, TGFB1, TGFB3, TGFBR2, TGFBR2 Adult. ver.3.0.4 (2021.8.24)

監修：厚労科研小杉班・Actionability Working Group-Japan

編集： MONSTAR-SCREEN-2遺伝事務局

***TMEM127*遺伝子について**

腫瘍*と関連する遺伝子の変化について*

- 一般的に腫瘍の一部は「生まれつきの遺伝子の変化」が原因といわれています。
  - 「生まれつきの遺伝子の変化」をもっている場合、腫瘍ができやすい体質をもっていると考えられます。
  - 腫瘍ができやすい体質は親、子、兄弟、姉妹などの血のつながった家族と共有している可能性があります。
  - 体質を知ることで、その情報をご自身やご家族の健康管理に役立てることができます。
  - 今回の遺伝子検査でみつかった変化が「生まれつきの遺伝子の変化」かどうかは、血液検査により確認できます。

*TMEM127*遺伝子とは？

- 腫瘍を発症するリスクと関連があることが示されている遺伝子です。裏面の資料 (表) をご参照ください。

「生まれつきの遺伝子の変化」であるかどうかを知ることはどんなことに役立ちますか？

- 発症リスクが高い腫瘍を知り、それに対する検診を行うことで、早期発見につながる可能性があります。

家族にどんな影響がありますか？
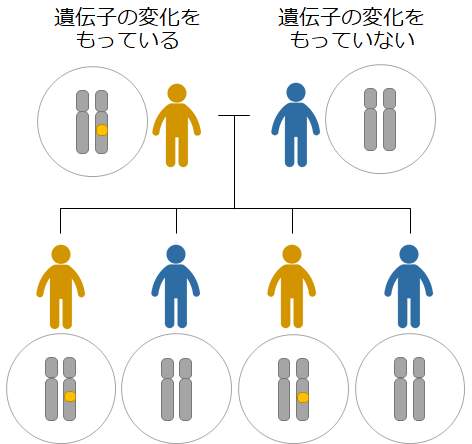


- 人は２つの*TMEM127*遺伝子をそれぞれ両親から1つずつ受け継いでいます。
- *TMEM127*遺伝子の変化を生まれつきもっていた場合、両親、兄弟、姉妹、子供が同じ変化をもっている確率は50%です。同じ変化をもっているかは、遺伝子検査で確認することができます。
- おば、おじ、いとこなども一定の確率で*同じ遺伝子の変化をもっている*可能性があります。
- 遺伝子の変化についてご家族の方と情報共有することは、ご家族の方の健康管理に役立つ可能性があります。

※生まれつきの*TMEM127*遺伝子の変化は、両親いずれかから受け継いだものではなく、新規に起こった変化である可能性もあります。この場合のご家族への影響については、遺伝専門外来におたずねください。

遺伝カウンセリングについて

遺伝カウンセリングでは、生まれつきの遺伝子の変化と体質との関連、ご家族への影響などについてより詳しくご説明します。お気軽にご相談ください。　注意：遺伝学的検査の保険適用には条件があり、関連する症状を発症していないご家族の遺伝子検査や検診は自費となります。詳しくは担当者におたずね下さい。

＜参考資料表＞

|  | 一般的な日本人 | *TMEM127*遺伝子に病気の原因となる変化を生まれつきもっている | | | |
| --- | --- | --- | --- | --- | --- |
|  | 生涯を通して  罹患する確率^1,2,3)^ | 生涯を通して  罹患する確率 | | 推奨される対応 | |
|  |  | 女性^3)^ | 男性^3)^ | 女性^3)^ | 男性^3)^ |
| パラガングリ  オーマ | 患者数：全国で  約1500人/年  ※米国データ：100万人に2人 | リスクが上がる  可能性 | | 1年ごとの血液検査(血中遊離メタネフリン)  2年ごとのCTまたはMRI検査  3年ごとのI-MIBGシンチグラフィ(画像検査) | |
| 副腎  褐色細胞腫 | 0.01～0.02 ％ | リスクが上がる  可能性 | |  |  |

※今回見つかった*TMEM127遺伝子*の変化が生まれつきの変化であっても、必ず症状がみられるというわけではありません。

※上記に示す罹患する確率は、良性・境界性の腫瘍を含めた確率であり、悪性腫瘍はその一部です。

※記載は日本の診療ガイドライン^3)^を参照しています。詳細は各施設の遺伝専門外来におたずねください。

※表は2021年時点の記載です。今後、研究が進み推奨内容が変わる可能性があります。

【参考文献】

- 国立がん研究センター希少がんセンター: パラガングリオーマ <https://www.ncc.go.jp/jp/rcc/about/paraganguriouma/index.html> [ref. 1]
- National Cancer Institute Center for Cancer Research <https://www.cancer.gov/pediatric-adult-rare-tumor/rare-tumors/rare-endocrine-tumor/paraganglioma> [ref. 2]
- 日本内分泌学会. 褐色細胞腫・パラガングリオーマ診療ガイドライン2018. (2018年7月) [ref. 3]
- GeneReviews Japan: 遺伝性パラガングリオーマ・褐色細胞腫症候群. ver.2020.7.15
- ClinGen Actionability Reports: MAX, SDHA, SDHAF2, SDHB, SDHC, SDHD, TMEM127 Adult. ver.1.1.3 (2022.1.3)

監修：厚労科研小杉班・Actionability Working Group-Japan

編集： MONSTAR-SCREEN-2遺伝事務局

***TP53*遺伝子について**

*がんの発症と関連する遺伝子の変化について*

- - 一般的にがんの5~10%は「生まれつきの遺伝子の変化」が原因といわれています。
  - 「生まれつきの遺伝子の変化」をもっている場合、がんになりやすい体質をもっていると考えられます。
  - がんになりやすい体質は親、子、兄弟、姉妹 などの血のつながった家族と共有している可能性があります。
  - 体質を知ることで、その情報をご自身やご家族の健康管理に役立てることができます。
  - 今回の遺伝子検査でみつかった変化が「生まれつきの遺伝子の変化」かどうかは、血液検査により確認できます。

*TP53*遺伝子とは？

- *TP53遺伝子*の生まれつきの変化は**リー・フラウメニ (Li-Fraumeni) 症候群**の原因となることが知られています。裏面の資料 (表) をご参照ください。

「生まれつきの遺伝子変化」であるかどうかを知ることはどんなことに役立ちますか？

- 発症リスクが高いがんを知り、それに対する検診を行うことで、早期発見につながる可能性があります。

家族にどんな影響がありますか？
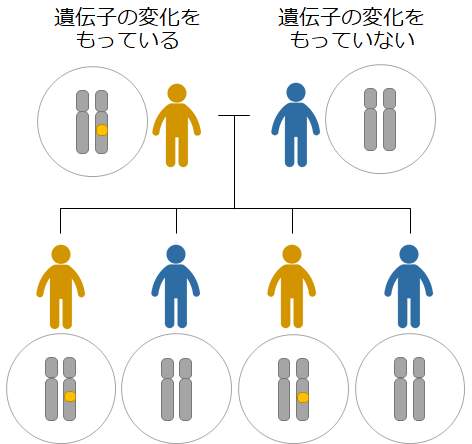


- 人は２つの*TP53*遺伝子をそれぞれ両親から1つずつ受け継いでいます。
- *TP53*遺伝子の変化を生まれつきもっていた場合、両親、兄弟、姉妹、子どもが同じ変化をもっている確率は50%です。同じ変化をもっているかは、遺伝子検査で確認することができます。
- おば、おじ、いとこなども一定の確率で*同じ遺伝子の変化をもっている*可能性があります。
- 遺伝子の変化についてご家族の方と情報共有することは、ご家族の方の健康管理に役立つ可能性があります。

※生まれつきの*TP53*遺伝子の変化は、両親いずれかから受け継いだものではなく、新規に起こった変化である可能性が約25%^1)^ (注意：リー・フラウメニ症候群として) といわれています。この場合のご家族への影響については、遺伝専門外来におたずねください。

遺伝カウンセリングについて

遺伝カウンセリングでは、生まれつきの遺伝子の変化と体質との関連、ご家族への影響などについてより詳しくご説明します。お気軽にご相談ください。 注意：遺伝学的検査の保険適用には条件があり、関連する症状を発症していないご家族の遺伝子検査や検診は自費となります。詳しくは担当者におたずね下さい。

＜参考資料表＞

|  | 一般的な日本人 | *TP53遺伝子*に病気の原因となる変化を生まれつきもっている | | | |
| --- | --- | --- | --- | --- | --- |
|  | 生涯を通して  がんに罹患する確率^2,3,4,5)^ | 生涯を通して  がんに罹患する確率^1)^ | | 成人に推奨される対応^1)^ | |
|  |  | 女性 | 男性 | 女性 | 男性 |
| 乳がん | 10.9 ％ (女性) | 25.0~59.6 ％ | ― | 18歳以降:自己乳房検診  20歳以降:半年ごとに医師による診察  20~75歳:年1回の乳房MRI検査  リスク低減乳房切除術（RRM）を考慮 | ― |
| 骨肉腫 | 発生頻度：500~800例/年 | 6.3~15.5 % | | 年１回の全身MRI  年1 回の腹部骨盤エコー | |
| 軟部肉腫 | 発生頻度：1500例/年 | 14.3~26.7 ％ | |  |  |
| 脳腫瘍 | 0.3 % (男性)  0.2 % (女性) | 5.4~13.0 % | | 年１回の脳MRI | |
| 副腎皮質  がん | 100万人当たり  0.72人程度  (米国のデータ) | 1.7~13.0 % | | 現時点で検診方法は確立されていません | |

- 表に示した内容の他に、消化管、皮膚等の定期的な検診、全身の身体診察等が推奨されています。小児 (出生～18歳) には別途対応が推奨されています。また、がん (二次がんを含む) の発生リスクを抑えるために、がんの放射線療法や日光浴、喫煙等を避けるべきであるとされています^1,6,7)^。

※*TP53遺伝子*の変化が生まれつきの変化であっても、必ずこれらのがんを発症するというわけではありませんが、生涯になんらかのがんを発症する確率は、女性でほぼ100％、男性で約75％となっています^1)^。

※記載は日本の診療ガイドライン^1)^を参照しています。各検診の詳細については各施設の遺伝専門外来におたずねください。

※表は2021年時点の記載です。今後、研究が進み推奨内容が変わる可能性があります。

【参考文献】

- 厚⽣労働省科学研究費補助⾦（がん政策研究事業）⼩児期に発症する遺伝性腫瘍に対するがんゲノム医療体制実装のための研究. リー・フラウメニ症候群の診療ガイドライン 2019 年度版. ver.1.1 (2020年3月) [ref. 1]
- 国立がん研究センターがん情報サービス 「累積がん罹患リスク(2018年データ)」 <https://ganjoho.jp/reg_stat/statistics/stat/summary.html> [ref. 2]
- 国立がん研究センター希少がんセンター: 骨の肉腫 https://www.ncc.go.jp/jp/rcc/about/bone_sarcomas/index.html [ref. 3]
- 厚生労働省健康局がん・疾病対策課. 平成30年全国がん登録 罹患数・率報告 2018 [ref. 4]
- Adrenocortical carcinoma in the United States: treatment utilization and prognostic factors. Cancer, 2008; 113(11): 3130. [ref. 5]
- NCCN Guidelines® Genetic/Familial High-Risk Assessment: Breast, Ovarian, and Pancreatic. ver.1.2022 (2021.8.11) [ref. 6]
- ClinGen Actionability Reports: TP53 Adult. ver.3.0.0 (2021.7.21) [ref. 7]
- GeneReviews Japan: リ・フラウメニ症候群. ver.2020.3.6

監修：厚労科研小杉班・Actionability Working Group-Japan

編集： MONSTAR-SCREEN-2遺伝事務局

***TSC1*遺伝子について**

*腫瘍と関連する遺伝子の変化について*

- 一般的に腫瘍の一部は「生まれつきの遺伝子の変化」が原因といわれています。
  - 「生まれつきの遺伝子の変化」をもっている場合、腫瘍ができやすい体質をもっていると考えられます。
  - 生まれつき腫瘍ができやすい体質は親、子、兄弟、姉妹などの血のつながった家族と共有している可能性があります。
  - 体質を知ることで、その情報をご自身やご家族の健康管理に役立てることができます。
  - 今回の遺伝子検査でみつかった変化が「生まれつきの遺伝子の変化」かどうかは、血液検査により確認できます。

*TSC1*遺伝子とは？

- *TSC1遺伝子*の生まれつきの変化は**結節性硬化症**の原因となることが知られています。裏面の資料 (表) をご参照ください。

「生まれつきの遺伝子の変化」であるかどうかを知ることはどんなことに役立ちますか？

- 発症リスクが高い症状を知り、それに対する検診を行うことで、早期発見につながる可能性があります。

家族にどんな影響がありますか？
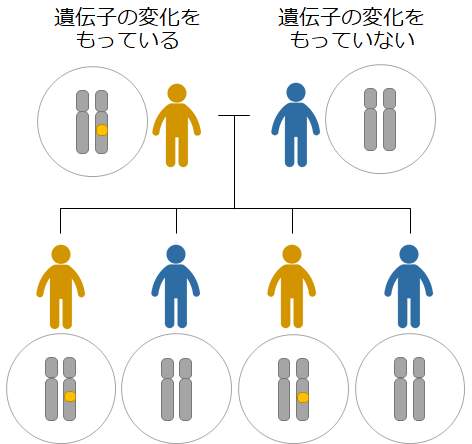


- 人は２つの*TSC1*遺伝子をそれぞれ両親から1つずつ受け継いでいます。
- *TSC1*遺伝子の変化を生まれつきもっていた場合、両親、兄弟、姉妹、子どもが同じ変化をもっている確率は50%です。同じ変化をもっているかは、遺伝子検査で確認することができます。
- おば、おじ、いとこなども一定の確率で*同じ遺伝子の変化をもっている*可能性があります。
- 遺伝子の変化についてご家族の方と情報共有することは、ご家族の方の健康管理にも役立つ可能性があります。

※生まれつきの*TSC1*遺伝子の変化は、両親いずれかから受け継いだものではなく、新規に起こった変化である可能性が約6割^1,2,3)^ (注意：結節性硬化症として) といわれています。この場合のご家族への影響については、遺伝専門外来におたずねください。

遺伝カウンセリングについて

遺伝カウンセリングでは、生まれつきの遺伝子の変化と体質との関連、ご家族への影響などについてより詳しくご説明します。お気軽にご相談ください。　注意：遺伝学的検査の保険適用には条件があり、関連する症状を発症していないご家族の遺伝子検査や検診は自費となります。詳しくは担当者におたずね下さい。

＜参考資料表＞

|  | 一般的な日本人 | *TSC1*遺伝子に病気の原因となる変化を生まれつきもっている | | | |
| --- | --- | --- | --- | --- | --- |
|  | 生涯を通して  罹患する  確率^4,5,6)^ | 生涯を通して  罹患する確率 | | 推奨される対応 | |
|  |  | 女性^1,2)^ | 男性^1,2)^ | 女性^1,2,7)^ | 男性^1,2,7)^ |
| 顔面  血管線維腫 | データなし | 5歳以上の80 % | | レーザーアブレーションや切除術  mTORC1阻害剤の内服や塗布 | |
| 横紋筋腫 | データなし | 47~67 % | | 1-3年ごとの心エコー検査、心電図検査 | |
| てんかん | 1000人に  5~8人 | 84 % | | 乳幼児期にてんかんを発症する可能性が予測される患者:  生後1カ月までに脳波の検査を考慮  小児の患者:てんかんの有無に関わらず脳波検査を行うのが望ましい  点頭てんかん症状：vigabatrin  その他症状に応じた治療 | |
| 脳室上衣下巨細胞性星状細胞腫 (SEGA) | データなし | 5~15 % | | 2-3年ごとの頭部画像診断 (MRIまたはCT) 検査 | |
| リンパ脈管  筋腫症 (LAM) | 100万人あたり  1.9-4.5人 | 30~40 % | 10~12 % | 5-10年ごとに精密肺機能検査，  6間歩行テスト、 高分解能CT | |
| 腎細胞がん | 10万人あたり  10.1人 (男性)  3.7人 (女性) | 2~4 % | | 1-2年ごとのMRI検査または腹部超音波検査 | |

- 上記症状以外にも、脳、心臓、肺、皮膚、歯などに症状がみられることがあります。詳細は遺伝専門外来におたずねください。

※*TSC1遺伝子*の変化が生まれつきの変化であっても、必ず症状がみられるというわけではありません。

※上記に示す罹患する確率は、良性・境界性の腫瘍を含めた確率であり、悪性腫瘍はその一部です。

※記載は日本の診療ガイドライン^1,7)^を参照しています。詳細については各施設の専門診療科あるいは遺伝専門外来におたずねください。

※表は2021年時点の記載です。今後、研究が進み推奨内容が変わる可能性があります。

【参考文献】

- 日本皮膚科学会. 結節性硬化症の診断基準及び治療ガイドライン 改訂版. 日皮会誌, 2018; 128(1): 1. [ref. 1]
- GeneReviews Japan: 結節性硬化症. ver.2020.9.29 [ref. 2]
- ClinGen Actionability Reports: TSC1, TSC2 Adult. ver.1.1.2 (2020.4.29) [ref. 3]
- 厚生労働省 てんかん対策 <https://www.mhlw.go.jp/stf/seisakunitsuite/bunya/0000070789_00008.html> [ref. 4]
- 林ら. 肺リンパ脈管筋腫症に関する全国疫学調査 追跡調査および第2回目全国横断調査．厚生労働省難治性疾患呼吸不全に関する調査研究 平成19年度総括・分担研究報告書．2008 [ref. 5]
- 国立がん研究センターがん情報サービス「がん統計」(全国がん登録) 2016年～2018年 [ref. 6]
- 日本脳腫瘍学会. 脳腫瘍診療ガイドライン 2019年版 小児脳腫瘍編 上衣下巨細胞性星細胞腫 (SEGA) 診療ガイドライン. (2019年5月) [ref. 7]

監修：厚労科研小杉班・Actionability Working Group-Japan

編集： MONSTAR-SCREEN-2遺伝事務局

***TSC2*遺伝子について**

*腫瘍と関連する遺伝子の変化について*

- 一般的に腫瘍の一部は「生まれつきの遺伝子の変化」が原因といわれています。
  - 「生まれつきの遺伝子の変化」をもっている場合、腫瘍ができやすい体質をもっていると考えられます。
  - 生まれつき腫瘍ができやすい体質は親、子、兄弟、姉妹などの血のつながった家族と共有している可能性があります。
  - 体質を知ることで、その情報をご自身やご家族の健康管理に役立てることができます。
  - 今回の遺伝子検査でみつかった変化が「生まれつきの遺伝子の変化」かどうかは、血液検査により確認できます。

*TSC2*遺伝子とは？

- 腫瘍が発生するリスクと関連があることが示されている遺伝子です。
- *TSC2遺伝子*の生まれつきの変化は**結節性硬化症**の原因となることが知られています。裏面の資料 (表) をご参照ください。

「生まれつきの遺伝子の変化」であるかどうかを知ることはどんなことに役立ちますか？

- 発症リスクが高い症状を知り、それに対する検診を行うことで、早期発見につながる可能性があります。

家族にどんな影響がありますか？
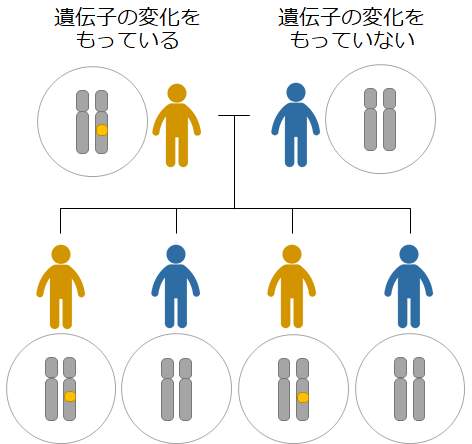


- 人は２つの*TSC2*遺伝子をそれぞれ両親から1つずつ受け継いでいます。
- *TSC2*遺伝子の変化を生まれつきもっていた場合、両親、兄弟、姉妹、子どもが同じ変化をもっている確率は50%です。同じ変化をもっているかは、遺伝子検査で確認することができます。
- おば、おじ、いとこなども一定の確率で*同じ遺伝子の変化をもっている*可能性があります。
- 遺伝子の変化についてご家族の方と情報共有することは、ご家族の方の健康管理にも役立つ可能性があります。

※生まれつきの*TSC2*遺伝子の変化は、両親いずれかから受け継いだものではなく、新規に起こった変化である可能性が約6割^1,2,3)^ (注意：結節性硬化症として) といわれています。この場合のご家族への影響については、遺伝専門外来におたずねください。

遺伝カウンセリングについて

遺伝カウンセリングでは、生まれつきの遺伝子の変化と体質との関連、ご家族への影響などについてより詳しくご説明します。お気軽にご相談ください。　注意：遺伝学的検査の保険適用には条件があり、関連する症状を発症していないご家族の遺伝子検査や検診は自費となる可能性があります。詳しくは担当者におたずね下さい。

＜参考資料表＞

|  | 一般的な日本人 | *TSC2*遺伝子に病気の原因となる変化を生まれつきもっている | | | |
| --- | --- | --- | --- | --- | --- |
|  | 生涯を通して  罹患する  確率^4,5,6)^ | 生涯を通して  罹患する確率 | | 推奨される対応 | |
|  |  | 女性^1,2)^ | 男性^1,2)^ | 女性^1,2,7)^ | 男性^1,2,7)^ |
| 顔面  血管線維腫 | データなし | 5歳以上の80 % | | レーザーアブレーションや切除術  mTORC1阻害剤の内服 | |
| 横紋筋腫 | データなし | 47~67% | | 1-3年ごとの心エコー検査、心電図検査 | |
| てんかん | 1000人に  5~8人 | 84 % | | 乳幼児期にてんかんを発症する可能性が予測される患者:  生後1カ月までに脳波の検査を考慮  小児の患者:てんかんの有無に関わらず脳波検査を行うのが望ましい  点頭てんかん症状：vigabatrin  その他症状に応じた治療 | |
| 脳室上衣下巨細胞性星状細胞腫 (SEGA) | データなし | 5~15% | | 2-3年ごとの頭部画像診断 (MRIまたはCT) 検査 | |
| リンパ脈管  筋腫症 (LAM) | 100万人あたり  1.9-4.5人 | 30~40 % | 10~12 % | 5-10年ごとに精密肺機能検査，  6 分間歩行テスト、高分解能CT | |
| 腎細胞がん | 10万人あたり  10.1人（男性）  3.7人（女性） | 2~4 % | | 1-２年ごとのMRI検査または腹部超音波検査 | |

- 上記症状以外にも、脳、心臓、肺、皮膚、歯などに症状がみられることがあります。詳細は遺伝専門外来におたずねください。

※*TSC2遺伝子*の変化が生まれつきの変化であっても、必ず症状がみられるというわけではありません。

※上記に示す罹患する確率は、良性・境界性の腫瘍を含めた確率であり、悪性腫瘍はその一部です。

※記載は日本の診療ガイドライン^1,7)^を参照しています。詳細については各施設の専門診療科あるいは遺伝専門外来におたずねください。

※表は2021年時点の記載です。今後、研究が進み推奨内容が変わる可能性があります。

【参考文献】

- 日本皮膚科学会. 結節性硬化症の診断基準及び治療ガイドライン 改訂版. 日皮会誌, 2018; 128(1): 1. [ref. 1]
- GeneReviews Japan: 結節性硬化症. ver.2020.9.29 [ref. 2]
- ClinGen Actionability Reports: TSC1, TSC2 Adult. ver.1.1.2 (2020.4.29) [ref. 3]
- 厚生労働省 てんかん対策 <https://www.mhlw.go.jp/stf/seisakunitsuite/bunya/0000070789_00008.html> [ref. 4]
- 林ら.　肺リンパ脈管筋腫症に関する全国疫学調査 追跡調査および第2回目全国横断調査．厚生労働省難治性疾患呼吸不全に関する調査研究 平成19年度総括・分担研究報告書. 2008 [ref. 5]
- 国立がん研究センターがん情報サービス「がん統計」(全国がん登録) 2016年～2018年 [ref. 6]
- 日本脳腫瘍学会. 脳腫瘍診療ガイドライン 2019年版 小児脳腫瘍編 上衣下巨細胞性星細胞腫 (SEGA) 診療ガイドライン. (2019年5月) [ref. 7]

監修：厚労科研小杉班・Actionability Working Group-Japan

編集： MONSTAR-SCREEN-2遺伝事務局

***VHL*遺伝子について**

*がんの発症と関連する遺伝子の変化について*

- 一般的にがんの5~10%は「生まれつきの遺伝子の変化」が原因といわれています。
  - 「生まれつきの遺伝子の変化」をもっている場合、がんになりやすい体質をもっていると考えられます。
  - がんになりやすい体質は親、子、兄弟、姉妹などの血のつながった家族と共有している可能性があります。
  - 体質を知ることで、その情報をご自身やご家族の健康管理に役立てることができます。
  - 今回の遺伝子検査でみつかった変化が「生まれつきの遺伝子の変化」かどうかは、血液検査により確認できます。

*VHL*遺伝子とは？

- *VHL遺伝子*の生まれつきの変化は**フォン・ヒッペル・リンドウ病** (Von Hippel-Lindau Syndrome) の原因となることが知られています。裏面の資料 (表) をご参照ください。

「生まれつきの遺伝子の変化」であるかどうかを知ることはどんなことに役立ちますか？

- 発症リスクが高い症状を知り、それに対する検診を行うことで、早期発見につながる可能性があります。

家族にどんな影響がありますか？
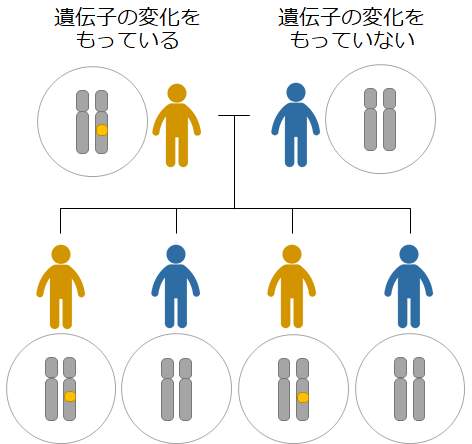


- 人は２つの*VHL*遺伝子をそれぞれ両親から1つずつ受け継いでいます。
- *VHL*遺伝子の変化を生まれつきもっていた場合、両親、兄弟、姉妹、子供が同じ変化をもっている確率は50%です。同じ変化をもっているかは、遺伝子検査で確認することができます。
- おば、おじ、いとこなども一定の確率で*同じ遺伝子の変化をもっている*可能性があります。
- 遺伝子の変化についてご家族の方と情報共有することは、ご家族の方の健康管理に役立つ可能性があります。

※生まれつきの*VHL*遺伝子の変化は、両親いずれかから受け継いだものではなく、新規に起こった変化である可能性が約20%^1)^ (注意：フォン・ヒッペル・リンドウ病として) といわれています。この場合のご家族への影響については、遺伝専門外来におたずねください。

遺伝カウンセリングについて

遺伝カウンセリングでは、生まれつきの遺伝子の変化と体質との関連、ご家族への影響などについてより詳しくご説明します。お気軽にご相談ください。　注意：遺伝学的検査の保険適用には条件があり、関連する症状を発症していないご家族の遺伝子検査や検診は自費となります。詳しくは担当者におたずね下さい。

＜参考資料表＞

|  | 一般的な日本人 | *VHL*遺伝子に病気の原因となる変化を生まれつきもっている | | | |
| --- | --- | --- | --- | --- | --- |
|  | 生涯を通して  罹患する確率^2,3,4,5,6)^ | 生涯を通して  罹患する確率 | | 推奨される対応 | |
|  |  | 女性^7)^ | 男性^7)^ | 女性^7)^ | 男性^7)^ |
| 中枢神経  血管芽腫 | 1000万人あたり  5~6人 | 60-80 ％ | | 2 年ごとに造影 MRI 検査 | |
| 網膜血管腫 | データなし | 40-70 ％ | | 3年ごとに眼底検査 | |
| 膵嚢胞 | 0.21~13.5% | 17-61 ％ | | 嚢胞径が大きくなり他臓器の圧迫症状などの臨床症状が出現するまで、経過観察あるいは治療の必要はない | |
| 腎嚢胞 | データなし | 60-80 ％ | | 嚢胞径に関わらず経過観察が推奨される | |
| 腎細胞がん | 10万人あたり  10.1人 (男性)  3.7人 (女性) | 25-50 % | | 1年ごとに超音波と単純MRI 検査を交互に実施 | |
| 膵・消化管  神経内分泌腫瘍 | 10万人あたり  2~3人 | 8-17 ％ | | 2~3年ごとの診察とCT検査  膵神経内分泌腫瘍が認められない場合は、1年ごとに腹部超音波と腹部単純MRI検査を実施 | |
| 副腎  褐色細胞腫 | 0.01~0.02 ％ | 10-20 % | | 蓄尿検査 (尿中メタネフリン、尿中ノルメタネフリン、アドレナリン、ノルアドレナリン) と血液検査(血中カテコールアミン) を開始、1年ごとに画像検査 | |

- 上表の症状以外にも、精巣、子宮、内耳等に症状がみられることがあります。詳細は遺伝専門外来におたずねください。

※今回見つかった*VHL遺伝子*の変化が生まれつきの変化であっても、必ず症状がみられるというわけではありません。

※上記に示す罹患する確率は、良性の腫瘍を含めた確率であり、悪性腫瘍はその一部です。

※記載は日本の診療ガイドライン^7)^を参照しています。 詳細は各施設の専門診療科あるいは遺伝専門外来におたずねください。

※表は2021年時点の記載です。今後、研究が進み推奨内容が変わる可能性があります。

【参考文献】

- GeneReviews Japan: フォンヒッペル・リンドウ病. ver.2018.8.25 [ref. 1]
- 全国がん登録罹患数・率 報告 平成30年 <https://www.mhlw.go.jp/content/10900000/000794199.pdf> [ref. 2]
- 中井. 膵嚢胞性腫瘍ガイドラインをめぐって〜超音波内視鏡診断・治療の役割〜. 埼玉医科大学雑誌, 2017; 43(2): 129. [ref. 3]
- 国立がん研究センターがん情報サービス「がん統計」(全国がん登録) 2016年～2018年 [ref. 4]
- 日本神経内分泌腫瘍研究会. 膵・消化管神経内分泌腫瘍診療ガイドライン第2版作成委員会. 膵・消化管神経内分泌腫瘍 (NEN) 診療ガイドライン 2019年第2版. (2019年9月) [ref. 5]
- 日本内分泌学会. 褐色細胞腫・パラガングリオーマ診療ガイドライン2018. (2018年7月) [ref. 6]
- 「多彩な内分泌異常を生じる遺伝性疾患(多発性内分泌腫瘍症およびフォン・ヒッペル・リンドウ病)の実態把握と診療標準化の研究」班. フォン・ヒッペル・リンドウ (VHL) 病 診療ガイドライン2017年版. (2017年) [ref. 7]
- ClinGen Actionability Reports: VHL Adult. ver.1.2.1 (2020.12.23) [ref. 8]

監修：厚労科研小杉班・Actionability Working Group-Japan

編集： MONSTAR-SCREEN-2遺伝事務局

***WT1*遺伝子について**

*腫瘍と関連する遺伝子の変化について*

- - 一般的に腫瘍の一部は「生まれつきの遺伝子の変化」が原因といわれています。
  - 「生まれつきの遺伝子の変化」をもっている場合、腫瘍ができやすい体質をもっていると考えられます。
  - 腫瘍ができやすい体質は親、子、兄弟、姉妹などの血のつながった家族と共有している可能性があります。
  - 体質を知ることで、その情報をご自身やご家族の健康管理に役立てることができます。
  - 今回の遺伝子検査でみつかった変化が「生まれつきの遺伝子の変化」かどうかは、血液検査により確認できます。

*WT1*遺伝子とは？

- *WT1遺伝子*の生まれつきの変化は***WT1関連ウィルムス腫瘍***の原因となることが知られています。裏面の資料 (表) をご参照ください。

「生まれつきの遺伝子の変化」であるかどうかを知ることはどんなことに役立ちますか？

- 発症リスクが高い腫瘍を知り、それに対する検診を行うことで、早期発見につながる可能性があります。

家族にどんな影響がありますか？
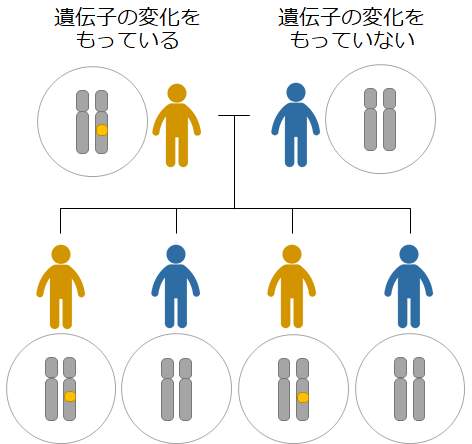


- 人は２つの*WT1*遺伝子をそれぞれ両親から1つずつ受け継いでいます。
- *WT1*遺伝子の変化を生まれつきもっていた場合、両親、兄弟、姉妹、子どもが同じ変化をもっている確率は50%です。同じ変化をもっているかは、遺伝子検査で確認することができます。
- おば、おじ、いとこなども一定の確率で*同じ遺伝子の変化をもっている*可能性があります。
- 遺伝子の変化についてご家族の方と情報共有することは、ご家族の方の健康管理に役立つ可能性があります。

※生まれつきの*WT1*遺伝子の変化は、両親いずれかから受け継いだものではなく、新規に起こった変化である可能性もあります。この場合のご家族への影響については、遺伝専門外来におたずねください。

遺伝カウンセリングについて

遺伝カウンセリングでは、生まれつきの遺伝子の変化と体質との関連、ご家族への影響などについてより詳しくご説明します。お気軽にご相談ください。　注意：遺伝学的検査の保険適用には条件があり、関連する症状を発症していないご家族の遺伝子検査や検診は自費となります。詳しくは担当者におたずね下さい。

＜参考資料表＞

|  | 一般的な日本人 | *WT1*遺伝子に病気の原因となる変化を生まれつきもっている | | | |
| --- | --- | --- | --- | --- | --- |
|  | 生涯を通して罹患する確率^1)^ | 生涯を通して  罹患する確率^2,3)^ | | 推奨される対応^4)^ | |
|  |  | 女性 | 男性 | 女性 | 男性 |
| ウィルムス腫瘍(腎芽腫) | データなし  日本では年間70~100例 | 38 %  ３歳前後に多く発症 | | 定期的な腹部超音波検査 (注) | |

(注) 記載の推奨事項については、本邦での具体的な検診方法は示されていません。詳細は各施設の遺伝専門外来におたずねください。

※記載は海外の資料を参照しています^2,3,4)^。

※表は2021年時点の記載です。今後、研究が進み推奨内容が変わる可能性があります。

【参考文献】

- 国立がん研究センターがん情報サービス 「累積がん罹患リスク (2018年データ)」 <https://ganjoho.jp/reg_stat/statistics/stat/summary.html> [ref. 1]
- Genotype-phenotype associations in WT1 glomerulopathy. Kidney Int, 2014; 85(5): 1169. [ref. 2]
- ClinGen Actionability Reports: WT1 Adult. ver.1.0.0 (2021.10.18) [ref. 3]
- GeneReviews Japan: ウィルムス腫瘍易罹患性. ver.2018.8.22 [ref. 4]

監修：厚労科研小杉班・Actionability Working Group-Japan

編集： MONSTAR-SCREEN-2遺伝事務局
