# Supplementary material for: Establishment of a comprehensive set of fact sheets for cancer predisposition genes for medical oncologists practicing cancer genome profiling
Source: Int J Clin Oncol. 2025 Apr 4;30(5):827–36. doi: 10.1007/s10147-025-02746-w (PMC12014809; doi:10.1007/s10147-025-02746-w)
Supplement: Supplementary file 2 — Supplementary file2 (DOCX 569 KB) [file 10147_2025_2746_MOESM2_ESM.docx]

***APC***

**Genetic Change and Hereditary Cancer Syndrome**

- - If genetic changes at birth are responsible for the hereditary cancer syndrome, the patient is considered to be predisposed to cancer.
  - In general, 5-10% of cancers are caused by genetic changes at birth.
  - Cancer susceptibility may be shared by relatives such as parents, children, siblings.
  - Understanding genetic risks allows better health management.
  - A blood test confirms whether the changes observed in genomic testing (tumor profiling) are hereditary.

**What Is *APC* Related To?**

- Genetic changes in the *APC* gene causes Familial Adenomatous Polyposis (see Table 1).

**The Benefit of Confirmatory Testing**

- It is important to know the cancer risk for prevention and early detection of cancer.

**
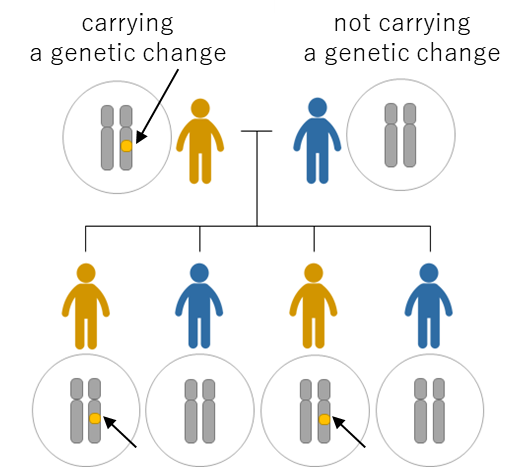
 Risk of Family Members**

- There are two copies of *APC*. One and another copy is inherited from the mother, and the other from the father, respectively.
- Each child, brother, sister, and parent have a 50% chance of inheriting genetic changes in *APC*. Genetic testing can be performed to examine if similar changes are exhibited.
- Aunts, uncles, and cousins may share genetic changes and risks.
- Sharing your genetic information with your relatives may help them manage their health.

※ There is a 20-25%^1)^ probability that individuals diagnosed with Familial Adenomatous Polyposis did not inherit it. This indicates that genetic changes appear in a family for the first time without an exact genetic change in the parents. Please consult your genetic specialist for further information about the impact of such a case on your family.

**What Is Genetic Counseling?**

Genetic Counseling provides information on how genetic conditions may affect you and/or your family　and how to manage it. Please feel free to contact your genetic specialist if you have any relevant questions. ※Genetic testing(s) are not always covered by public health insurance in Japan. Genetic testing and follow-up care for healthy relatives who are unaffected by cancer are not covered by insurance. Please ask Genetic Services for details.

Table 1. Lifetime Risk and Recommended Management

|  | Japanese | Carrier of *APC* pathogenic / likely pathogenic variant | | | |
| --- | --- | --- | --- | --- | --- |
|  | Lifetime risk^2,3)^ | Lifetime risk | | Recommended management | |
|  |  | Women^1,4,5,6)^ | Men^1,4,5,6)^ | Women^1,4)^ | Men^1,4)^ |
| Colorectal Polyposis  (Risk of cancer) | (Colorectal cancer)  10.2 ％ (Men)  8.0 ％ (Women) | （Colon polyps）  Until 35y: 95 %  （Colorectal cancer）  Until 40-55y: 50 % | | ・Risk-reduction surgery for colorectal cancer: the timing is generally in the 20s, but it depends upon symptoms and social background  ・Colonoscopy: every 1-3 years depending on polyp burden | |
| Gastric Fundic Gland Polyposis  ・Gastric Adenoma  (Risk of cancer) | (Gastric cancer)  10.3 ％ (Men)  4.7 ％ (Women) | Until 50y  （Gastric adenoma）  21.8 %  (Gastric cancer）  3.8 ％ | | ・Annual upper endoscopy after the age 20-25y | |
| Duodenal Polyposis  (Risk of cancer) | (Small intestinal cancer)  0.2% (Men)  0.1% (Women) | (Duodenal cancer)  4-12 % | |  |  |
| Desmoid Tumor | No data available | 10-15 % | | ・Annual abdominal examination：palpation, CT or MRI | |
| Thyroid Cancer | 0.6 ％ (Men)  1.7 ％ (Women) | (Papillary thyroid carcinoma、particularly for women)  1-12% | | ・Annual thyroid palpation and thyroid ultrasound(Particularly for women) | |
| Brain Tumor | 0.3 ％ (Men)  0.2 ％ (Women) | Potential increased risk | | ・Annual examination | |

- Other conditions, such as hepatoblastoma and adrenal tumors, may occur in association with the genetic change in the *APC* gene. Please contact specialists and Genetic Services for further details.

※It is important to note that not all carriers who inherit genetic changes in *APC* develop cancer.

※The likelihood of being symptomatic if carrying a genetic change and recommended management is based on the Japanese guideline^1)^ and the National Comprehensive Cancer Network guideline^4)^. However, these recommendations are not always implemented in Japan. Please contact specialists and Genetic Services if management is provided at the hospital.

※Table1 is based on information as of 2021. Recommendations may be revised as research progresses.

【References】

- 大腸癌研究会. 遺伝性大腸癌診療ガイドライン 2020年版. (2020年4月) [ref. 1]
- 国立がん研究センターがん情報サービス「累積がん罹患リスク(2018年データ)」<https://ganjoho.jp/reg_stat/statistics/stat/summary.html> [ref. 2]
- 厚生労働省健康局がん・疾病対策課. 平成30年全国がん登録 罹患数・率報告 2018 [ref. 3]
- NCCN Guidelines® Genetic/Familial High-Risk Assessment: Colorectal. ver.1.2021 (2021.5.11) [ref. 4]
- GeneReviews Japan: *APC関連ポリポーシス*. ver.2018.5.6 [ref. 5]
- Upper gastrointestinal tumours in Japanese familial adenomatous polyposis patients. Jpn J Clin Oncol, 2016; 46(4): 310. [ref. 6]
- ClinGen Actionability Reports: APC Adult. ver.3.0.4 (2021.5.6)

supervising editor： Research Group for the Research Project on Ethical, Legal, and Social Issues Supported by the Health, Labour and Welfare Sciences Research Grants “Extraction of ethical and social issues and improvement of social environment toward the realization of a society where people can benefit from genome medicine without anxiety,” Actionability Working Group-Japan

Edited by MONSTAR-SCREEN-2　Medical Genetic Office

***ATM***

**Genetic Change and Hereditary Cancer Syndrome**

- - If genetic changes at birth are responsible for the hereditary cancer syndrome, the patient is considered to be predisposed to cancer.
  - In general, 5-10% of cancers are caused by genetic changes at birth.
  - Cancer susceptibility may be shared by relatives such as parents, children, siblings.
  - Understanding genetic risks allows better health management.
  - A blood test confirms whether the changes observed in genomic testing (tumor profiling) are hereditary.

**What Is *ATM* Related To?**

- *ATM* has been shown to be associated with an increased risk of cancer (see Table 1).

**The Benefit of Confirmatory Testing**

- It is important to know the cancer risk for prevention and early detection of cancer.


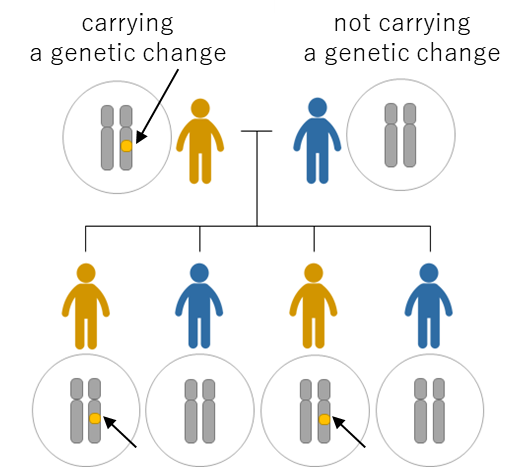
 **Risk of Family Members**

- There are two copies of *ATM*. One and another copy is inherited from the mother, and the other from the father, respectively.
- Each child, brother, sister, and parent have a 50% chance of inheriting genetic changes in *ATM*. Genetic testing can be performed to examine if similar changes are exhibited.
- Aunts, uncles, and cousins may share genetic changes and risks.
- Sharing your genetic information with your relatives may help them manage their health.

※ It is possible that the *ATM* gene change is not inherited from either parent but is a *de* *novo* change. Please consult your genetic specialist for information about the impact of such a case on your family.

**What Is Genetic Counseling?**

Genetic Counseling provides information on how genetic conditions may affect you and/or your family　and how to manage it. Please feel free to contact your genetic specialist if you have any relevant questions. ※Genetic testing(s) are not always covered by public health insurance in Japan. Genetic testing and follow-up care for healthy relatives who are unaffected by cancer are not covered by insurance. Please ask Genetic Services for details.

Table 1. Lifetime Risk and Recommended Management

|  | Japanese | Carrier of *ATM* pathogenic / likely pathogenic variant | | | |
| --- | --- | --- | --- | --- | --- |
|  | Lifetime risk^1)^ | Lifetime risk | | Recommended management | |
|  |  | Women^2)^ | Men^2)^ | Women^2)^ | Men^2)^ |
| Breast Cancer | 10.9 ％  (Women) | 15ー40 ％ | ― | ・Starting at age 40y: annual mammogram and breast MRI screening with contrast* | ― |
| Pancreatic Cancer | 2.6 % (Women)  2.6 % (Men) | ≦10 % | | ※Currently, there is no established management. | |
| Ovarian Cancer | 1.6 % (Women) | < 3 % | ― | ※Currently, there is no established management. Risks are considered to vary depending on family and medical history. | ― |
| Prostate Cancer | 10.8 ％ (Men) | ― | Insufficient evidence | ― | ― |

* The recommended management in Table 1 is not specifically presented in Japan. Please contact the genetic specialist at your institution for further details.

※It is important to note that not all carriers who inherit genetic changes in *ATM* develop cancer.

※The likelihood of being symptomatic if carrying a genetic change and recommended management is based on the National Comprehensive Cancer Network guideline ^2)^.

※Table1 is based on information as of 2021. Recommendations may be revised as research progresses.

【References】

- 国立がん研究センターがん情報サービス「累積がん罹患リスク (2018年データ)」https://ganjoho.jp/reg_stat/statistics/stat/summary.html [ref. 1]
- NCCN Guidelines® Genetic/Familial High-Risk Assessment: Breast, Ovarian, and Pancreatic. ver.1.2022 (2021.8.11) [ref. 2]
- ClinGen Actionability Reports: ATM, CHEK2 Adult. ver.1.1.1 (2020.4.16)

supervising editor： Research Group for the Research Project on Ethical, Legal, and Social Issues Supported by the Health, Labour and Welfare Sciences Research Grants “Extraction of ethical and social issues and improvement of social environment toward the realization of a society where people can benefit from genome medicine without anxiety,” Actionability Working Group-Japan

Edited by MONSTAR-SCREEN-2　Medical Genetic Office

***BAP1***

**Genetic Change and Hereditary Cancer Syndrome**

- - If genetic changes at birth are responsible for the hereditary cancer syndrome, the patient is considered to be predisposed to cancer.
  - In general, 5-10% of cancers are caused by genetic changes at birth.
  - Cancer susceptibility may be shared by relatives such as parents, children, siblings.
  - Understanding genetic risks allows better health management.
  - A blood test confirms whether the changes observed in genomic testing (tumor profiling) are hereditary.

**What Is *BAP1* Related To?**

- Genetic changes in the *BAP1* gene causes *BAP1* tumor predisposition syndrome (see Table 1).

**The Benefit of Confirmatory Testing**

- It is important to know the cancer risk for prevention and early detection of cancer.


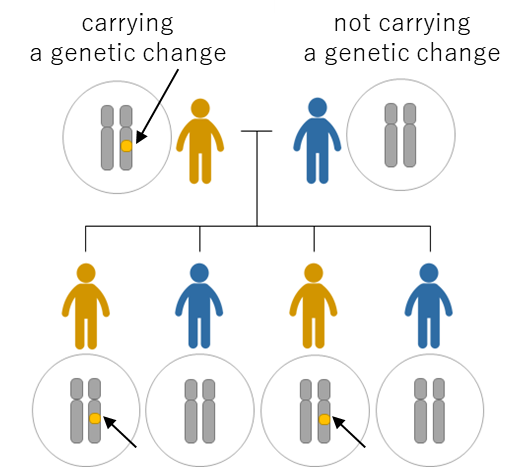
 **Risk of Family Members**

- There are two copies of *BAP1*. One and another copy is inherited from the mother, and the other from the father, respectively.
- Each child, brother, sister, and parent have a 50% chance of inheriting genetic changes in *BAP1*. Genetic testing can be performed to examine if similar changes are exhibited.
- Aunts, uncles, and cousins may share genetic changes and risks.
- Sharing your genetic information with your relatives may help them manage their health.

※ It is possible that the *BAP1* gene change is not inherited from either parent but is a *de* *novo* change. Please consult your genetic specialist for information about the impact of such a case on your family.

**What Is Genetic Counseling?**

Genetic Counseling provides information on how genetic conditions may affect you and/or your family　and how to manage it. Please feel free to contact your genetic specialist if you have any relevant questions. ※Genetic testing(s) are not always covered by public health insurance in Japan. Genetic testing and follow-up care for healthy relatives who are unaffected by cancer are not covered by insurance. Please ask Genetic Services for details.

Table 1. Lifetime Risk and Recommended Management

|  | Japanese | | Carrier of *BAP1* pathogenic / likely pathogenic variant | |
| --- | --- | --- | --- | --- |
|  | Lifetime risk^1,2)^ | | Lifetime risk^3)^ | Recommended management^3,4,5)^ |
|  | Women | Men |  |  |
| Atypical Spitz Tumor | Unknown | | 76 % | ・Starting after the age 20y: annual dermatologic examinations ** |
| Cutaneous Melanoma* | 0.1 % | 0.1 % | 13 % |  |
| Basal Cell Carcinoma | (Skin cancer) | | 6.3 ％ |  |
|  | 0.5 % | 0.6 % |  |  |
| Uveal Malignant　Melanoma | ※Frequency of occurrence:  50 cases/year | | 31 % | ・Starting after the age 11y: annual eye examinations** |
| Malignant Mesothelioma | (Mesothelioma) | | 39 % | ・Currently, there is no established management. |
|  | 0.0 % | 0.1 % |  |  |
| Renal Cell Carcinoma | (Renal cancer (except renal pelvis cancer)) | | 10 % | ・Starting after the age 30y: abdominal MRI (recommended) or CT every 2 years** |
|  | 0.4 % | 1.2 % |  |  |

* The annual prevalence of malignant melanoma is reported to be 24.3 in 100,000 people for Caucasians and 1.7 in 100,000 people for Asians^6)^, and the risk is considered to vary according to race, region, and other genetic factors.

** The recommended management in Table 1 is not specifically presented in Japan. Please contact the genetic specialist at your institution for further details.

※It is important to note that not all carriers who inherit genetic changes in *BAP1* develop cancer.

※The likelihood of being symptomatic if carrying a genetic change and recommended management is based on the international data^3,4,5)^.

※Table1 is based on information as of 2021. Recommendations may be revised as research progresses.

【References】

- 厚生労働健康局がん・疾病対策課. 平成30年全国がん登録 罹患数・率報告 2018 [ref. 1]
- 国立がん研究センター希少がんセンター<https://www.ncc.go.jp/jp/rcc/index.html> [ref. 2]
- ClinGen Actionability Reports: BAP1 Adult. ver.1.2.1 (2020.4.22) [ref. 3]
- NCCN Guidelines®: Kidney Cancer. ver.4.2022 (2021.12.21) [ref. 4]
- GeneReviews Japan: *BAP1*腫瘍素因症候群. ver.2019.3.22 [ref. 5]

- 日本皮膚科学会. 皮膚悪性腫瘍ガイドライン第 3 版 メラノーマ診療ガイドライン 2019. 日皮会誌, 2019; 129(9): 1759. [ref. 6]

supervising editor： Research Group for the Research Project on Ethical, Legal, and Social Issues Supported by the Health, Labour and Welfare Sciences Research Grants “Extraction of ethical and social issues and improvement of social environment toward the realization of a society where people can benefit from genome medicine without anxiety,” Actionability Working Group-Japan

Edited by MONSTAR-SCREEN-2　Medical Genetic Office

***BARD1***

**Genetic Change and Hereditary Cancer Syndrome**

- - If genetic changes at birth are responsible for the hereditary cancer syndrome, the patient is considered to be predisposed to cancer.
  - In general, 5-10% of cancers are caused by genetic changes at birth.
  - Cancer susceptibility may be shared by relatives such as parents, children, siblings.
  - Understanding genetic risks allows better health management.
  - A blood test confirms whether the changes observed in genomic testing (tumor profiling) are hereditary.

**What Is *BARD1* Related To?**

- *BARD1* has been shown to be associated with an increased risk of cancer (see Table 1).

**The Benefit of Confirmatory Testing**

- It is important to know the cancer risk for prevention and early detection of cancer.


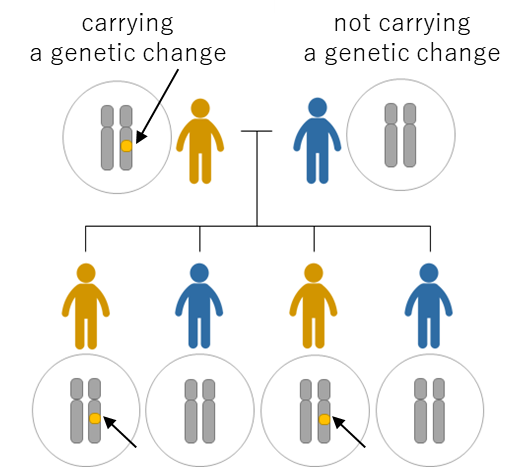
 **Risk of Family Members**

- There are two copies of *BARD1*. One and another copy is inherited from the mother, and the other from the father, respectively.
- Each child, brother, sister, and parent have a 50% chance of inheriting genetic changes in *BARD1*. Genetic testing can be performed to examine if similar changes are exhibited.
- Aunts, uncles, and cousins may share genetic changes and risks.
- Sharing your genetic information with your relatives may help them manage their health.

※ It is possible that the *BARD1* gene change is not inherited from either parent but is a *de* *novo* change. Please consult your genetic specialist for information about the impact of such a case on your family.

**What Is Genetic Counseling?**

Genetic Counseling provides information on how genetic conditions may affect you and/or your family　and how to manage it. Please feel free to contact your genetic specialist if you have any relevant questions. ※Genetic testing(s) are not always covered by public health insurance in Japan. Genetic testing and follow-up care for healthy relatives who are unaffected by cancer are not covered by insurance. Please ask Genetic Services for details.

Table 1. Lifetime Risk and Recommended Management

|  | Japanese | Carrier of *BARD1* pathogenic / likely pathogenic variant | | | | |
| --- | --- | --- | --- | --- | --- | --- |
|  | Lifetime risk^1)^ | | Lifetime risk | | Recommended management | |
|  |  |  | Women^2)^ | Men | Women^2)^ | Men |
| Breast Cancer | 10.9 ％（women） | | 15-40 ％ | ― | ・Starting at age 40y: annual mammogram and breast MRI screening with contrast* | ― |

* The recommended management in Table 1 is not specifically presented in Japan. Please contact the genetic specialist at your institution for further details.

※It is important to note that not all carriers who inherit genetic changes in *BARD1* develop cancer.

※The likelihood of being symptomatic if carrying a genetic change and recommended management is based on the National Comprehensive Cancer Network guideline^2)^.

※Table1 is based on information as of 2021. Recommendations may be revised as research progresses.

【References】

- 国立がん研究センターがん情報サービス「累積がん罹患リスク (2018年データ)」 https://ganjoho.jp/reg_stat/statistics/stat/summary.html [ref. 1]
- NCCN Guidelines® Genetic/Familial High-Risk Assessment: Breast, Ovarian, and Pancreatic. ver.1.2022 (2021.8.11) [ref. 2]

supervising editor： Research Group for the Research Project on Ethical, Legal, and Social Issues Supported by the Health, Labour and Welfare Sciences Research Grants “Extraction of ethical and social issues and improvement of social environment toward the realization of a society where people can benefit from genome medicine without anxiety,” Actionability Working Group-Japan

Edited by MONSTAR-SCREEN-2　Medical Genetic Office

***BMPR1A***

**Genetic Change and Hereditary Cancer Syndrome**

- - If genetic changes at birth are responsible for the hereditary cancer syndrome, the patient is considered to be predisposed to cancer.
  - In general, 5-10% of cancers are caused by genetic changes at birth.
  - Cancer susceptibility may be shared by relatives such as parents, children, siblings.
  - Understanding genetic risks allows better health management.
  - A blood test confirms whether the changes observed in genomic testing (tumor profiling) are hereditary.

**What Is *BMPR1A* Related To?**

- Genetic changes in the *BMPR1A* gene causes Juvenile Polyposis Syndrome (see Table 1).

**The Benefit of Confirmatory Testing**

- It is important to know the cancer risk for prevention and early detection of cancer.


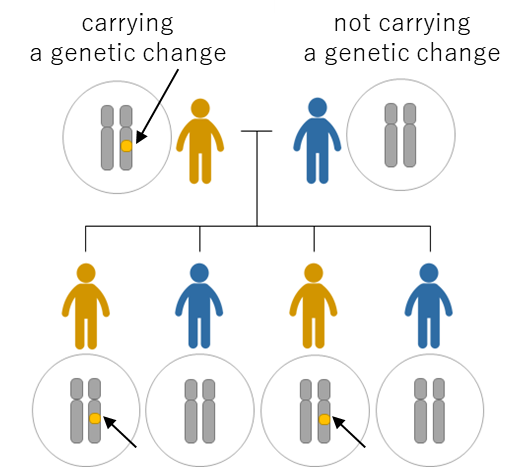
 **Risk of Family Members**

- There are two copies of *BMPR1A*. One and another copy is inherited from the mother, and the other from the father, respectively.
- Each child, brother, sister, and parent have a 50% chance of inheriting genetic changes in *BMPR1A*. Genetic testing can be performed to examine if similar changes are exhibited.
- Aunts, uncles, and cousins may share genetic changes and risks.
- Sharing your genetic information with your relatives may help them manage their health.

※ It is possible that the *BMPR1A* gene change is not inherited from either parent but is a *de* *novo* change. Please consult your genetic specialist for information about the impact of such a case on your family.

**What Is Genetic Counseling?**

Genetic Counseling provides information on how genetic conditions may affect you and/or your family　and how to manage it. Please feel free to contact your genetic specialist if you have any relevant questions. ※Genetic testing(s) are not always covered by public health insurance in Japan. Genetic testing and follow-up care for healthy relatives who are unaffected by cancer are not covered by insurance. Please ask Genetic Services for details.

Table 1. Lifetime Risk and Recommended Management

|  | Japanese | Carrier of *BMPR1A* pathogenic / likely pathogenic variant | | | |
| --- | --- | --- | --- | --- | --- |
|  | Lifetime risk^1,2)^ | Lifetime risk | | Recommended management | |
|  |  | Women^3,4)^ | Men^3,4)^ | Women^3,5)^ | Men^3,5)^ |
| Colon Polyp  (Risk of cancer) | (Colorectal cancer)  10.2 ％ (Men)  8.0 ％ (Women) | （Polyps）  ≦90 %  (Colorectal cancer)  ≦68 %  (Gastric・Small intestinal cancer)  ≦21 %* | | ・Upper endoscopy and colonoscopy every 1-3 years | |
| Gastric Polyp (Risk of cancer) | (Gastric cancer)  10.3 ％ (Men)  4.7 ％ (Women) |  |  |  |  |
| Small intestine Polyp | (Small intestinal cancer)  0.2% (Men)  0.1% (Women) |  |  |  |  |

* The prevalence of a juvenile polyposis.

※It is important to note that not all carriers who inherit genetic changes in *BMPR1A* develop cancer. Most patients with juvenile polyposis syndrome are found to have several polyps by the age of 20. Please contact specialists and Genetic Services for further details.

※The likelihood of being symptomatic if carrying a genetic change and recommended management is based on the Japanese guideline^3)^ and the National Comprehensive Cancer Network guideline^5)^. However, these recommendations are not always implemented in Japan. Please contact specialists and Genetic Services if management is provided at the hospital.

※Table1 is based on information as of 2021. Recommendations may be revised as research progresses.

【References】

- 国立がん研究センターがん情報サービス「累積がん罹患リスク (2018年データ)」<https://ganjoho.jp/reg_stat/statistics/stat/summary.html> [ref. 1]
- 厚生労働省健康局がん・疾病対策課 平成30年全国がん登録 罹患数・率報告 2018 [ref. 2]
- 小児・成人のための若年性ポリポーシス 症候群診療ガイドライン 2020年版. 遺伝性腫瘍, 2020; 20(2): 79. [ref. 3]
- ClinGen Actionability Reports: SMAD4, BMPR1A Adult. ver.1.1.1 (2021.1.19) [ref. 4]
- NCCN Guidelines® Genetic/Familial High-Risk Assessment: Colorectal. ver.1.2021 (2021.5.11) [ref. 5]
- GeneReviews Japan: 若年性ポリポーシス症候群. ver.2014.3.3.

supervising editor： Research Group for the Research Project on Ethical, Legal, and Social Issues Supported by the Health, Labour and Welfare Sciences Research Grants “Extraction of ethical and social issues and improvement of social environment toward the realization of a society where people can benefit from genome medicine without anxiety,” Actionability Working Group-Japan

Edited by MONSTAR-SCREEN-2　Medical Genetic Office

***BRCA1***

**Genetic Change and Hereditary Cancer Syndrome**

- - If genetic changes at birth are responsible for the hereditary cancer syndrome, the patient is considered to be predisposed to cancer.
  - In general, 5-10% of cancers are caused by genetic changes at birth.
  - Cancer susceptibility may be shared by relatives such as parents, children, siblings.
  - Understanding genetic risks allows better health management.
  - A blood test confirms whether the changes observed in genomic testing (tumor profiling) are hereditary.

**What Is *BRCA1* Related To?**

- Genetic changes in the *BRCA1* gene causes Hereditary Breast and Ovarian Cancer (see Table 1).

**The Benefit of Confirmatory Testing**

- It is important to know the cancer risk for prevention and early detection of cancer.


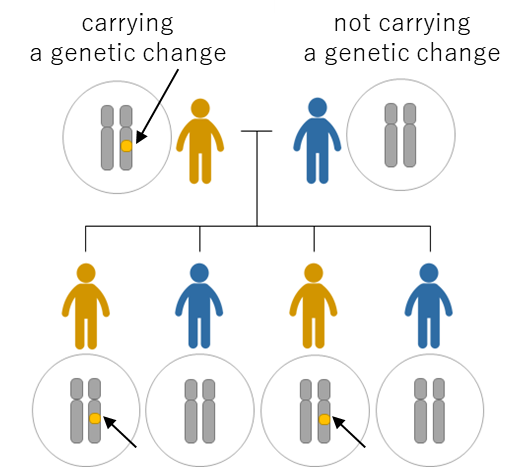
 **Risk of Family Members**

- There are two copies of *BRCA1*. One and another copy is inherited from the mother, and the other from the father, respectively.
- Each child, brother, sister, and parent have a 50% chance of inheriting genetic changes in *BRCA1*. Genetic testing can be performed to examine if similar changes are exhibited.
- Aunts, uncles, and cousins may share genetic changes and risks.
- Sharing your genetic information with your relatives may help them manage their health.

※ There is a 0.1%^1)^ probability that an individual with a *BRCA1* genetic change will not inherit the mutation. This indicates that genetic changes appear in a family for the first time without an exact genetic change in the parents. Please consult your genetic specialist for further information about the impact of such a case on your family.

**What Is Genetic Counseling?**

Genetic Counseling provides information on how genetic conditions may affect you and/or your family　and how to manage it. Please feel free to contact your genetic specialist if you have any relevant questions. ※Genetic testing(s) are not always covered by public health insurance in Japan. Genetic testing and follow-up care for healthy relatives who are unaffected by cancer are not covered by insurance. Please ask Genetic Services for details.

Table 1. Lifetime Risk and Recommended Management

|  | Japanese | Carrier of *BRCA1* pathogenic / likely pathogenic variant | | | |
| --- | --- | --- | --- | --- | --- |
|  | Lifetime risk^2,3)^ | Lifetime risk | | Recommended management | |
|  |  | Women^3,4,5)^ | Men^3,6)^ | Women^3,4,5,8)^ | Men^3,6,8)^ |
| Breast Cancer | 10.9 ％  (Women)  0.1 ％  (Men) | 57-72 ％ | 1.2 ％ | ・Starting at age 18y: breast awareness  ・Starting at age 25y: clinical breast exam every 6-12 months  ・Starting after the age 25-29y: annual breast MRI screening with contrast  ・Starting after the age 30-75y: annual mammogram and breast MRI screening with contrast  ・Discuss the option of risk-reducing mastectomy | ・Starting at age 35y: Breast self-exam, clinical breast exam, every year  ・ (men with gynecomastia) Starting at age 50y: annual mammogram |
| Ovarian Cancer | 1.6 %  (Women) | 40-44 % | ― | ・Age 35-40y: Recommend risk-reducing salpingo-oophorectpmy (RRSO)  ・Starting after the age 30-35y:　Consider transvaginal ultrasound combined with serum CA-125 | ― |
| Prostate Cancer | 10.8 ％  (Men) | ― | Until 65y　8.6 ％* | ― | ・Starting at　age 40y: consider prostate cancer screening (PSA) |
| Pancreatic Cancer | 2.6 %  (Men and Women) | ≦5% | | ・Consider screening using MRI and/or endoscopic ultrasound. | |

*　For under 65y carriers, it is a 1.8 to 3.8 fold increased risk compared to the general population.^6,7)^

※It is important to note that not all carriers who inherit genetic changes in *BRCA1* develop cancer.

※The likelihood of being symptomatic if carrying a genetic change and recommended management is based on the Japanese guideline^3)^ and the National Comprehensive Cancer Network guideline^8)^. However, these recommendations are not always implemented in Japan. Please contact specialists and Genetic Services if management is provided at the hospital.

※Table1 is based on information as of 2021. Recommendations may be revised as research progresses.

【References】

- Breast and ovarian cancer predisposition due to de novo BRCA1 and BRCA2 mutations. Oncogene, 2016; 35(10): 1324. [ref. 1]
- 国立がん研究センターがん情報サービス「累積がん罹患リスク (2018年データ)」 <https://ganjoho.jp/reg_stat/statistics/stat/summary.html> [ref. 2]
- 日本遺伝性乳癌卵巣癌総合診療精度機構. 遺伝性乳癌卵巣癌 (HBOC) 診療ガイドライン2021年版. (2021年7月) [ref. 3]
- Meta-analysis of BRCA2 and BRCA2 penetrance. J Clin Oncol, 2007; 25(11): 1329. [ref. 4]
- Risks of Breast, Ovarian, and Contralateral Breast Cancer for BRCA2 and BRCA2 Mutation Carriers. JAMA, 2017; 317(23): 2402. [ref. 5]
- Germline BRCA1 mutations increase prostate cancer risk. Br J Cancer, 2012; 106(10): 1697. [ref. 6]
- Cancer incidence in BRCA1 mutation carriers. J Natl Cancer Inst, 2002; 94(18): 1358. [ref. 7]
- NCCN Guidelines® Genetic/Familial High-Risk Assessment: Breast, Ovarian, and Pancreatic. ver.1.2022 (2021.8.11) [ref. 8]
- GeneReviews Japan: *BRCA1*および*BRCA2*関連遺伝性乳癌卵巣癌. ver.2016.12.25 (minor revision; 2017.2.20)
- ClinGen Actionability Reports: BRCA1, BRCA2 Adult. ver.1.1.3 (2021.9.15)

supervising editor： Research Group for the Research Project on Ethical, Legal, and Social Issues Supported by the Health, Labour and Welfare Sciences Research Grants “Extraction of ethical and social issues and improvement of social environment toward the realization of a society where people can benefit from genome medicine without anxiety,” Actionability Working Group-Japan

Edited by MONSTAR-SCREEN-2　Medical Genetic Office

***BRCA2***

**Genetic Change and Hereditary Cancer Syndrome**

- - If genetic changes at birth are responsible for the hereditary cancer syndrome, the patient is considered to be predisposed to cancer.
  - In general, 5-10% of cancers are caused by genetic changes at birth.
  - Cancer susceptibility may be shared by relatives such as parents, children, siblings.
  - Understanding genetic risks allows better health management.
  - A blood test confirms whether the changes observed in genomic testing (tumor profiling) are hereditary.

**What Is *BRCA2* Related To?**

- Genetic changes in the *BRCA2* gene causes Hereditary Breast and Ovarian Cancer (see Table 1).

**The Benefit of Confirmatory Testing**

- It is important to know the cancer risk for prevention and early detection of cancer.


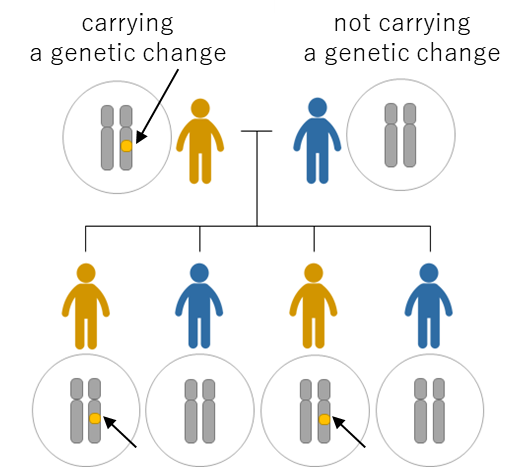
 **Risk of Family Members**

- There are two copies of *BRCA2*. One and another copy is inherited from the mother, and the other from the father, respectively.
- Each child, brother, sister, and parent have a 50% chance of inheriting genetic changes in *BRCA2*. Genetic testing can be performed to examine if similar changes are exhibited.
- Aunts, uncles, and cousins may share genetic changes and risks.
- Sharing your genetic information with your relatives may help them manage their health.

※ There is a 0.7%^1)^ probability that an individual with a *BRCA2* genetic change will not inherit the mutation. This indicates that genetic changes appear in a family for the first time without an exact genetic change in the parents. Please consult your genetic specialist for further information about the impact of such a case on your family.

**What Is Genetic Counseling?**

Genetic Counseling provides information on how genetic conditions may affect you and/or your family　and how to manage it. Please feel free to contact your genetic specialist if you have any relevant questions. ※Genetic testing(s) are not always covered by public health insurance in Japan. Genetic testing and follow-up care for healthy relatives who are unaffected by cancer are not covered by insurance. Please ask Genetic Services for details.

Table 1. Lifetime Risk and Recommended Management

|  | Japanese | Carrier of *BRCA2* pathogenic / likely pathogenic variant | | | | |
| --- | --- | --- | --- | --- | --- | --- |
|  | Lifetime risk^2,3)^ | Lifetime risk | | | Recommended management | |
|  |  | Women^3,4,5)^ | Men^3,6,7,8,9)^ | Women^3,10)^ | | Men^3,10)^ |
| Breast Cancer | 10.9 ％  (Women)  0.1 ％  (Men) | 49-69 ％ | 7-8 ％ | ・Starting at age 18y: breast awareness  ・Starting at age 25y: clinical breast exam every 6-12 months  Starting after the age 25-29y: annual breast MRI screening with contrast  ・Starting after the age 30-75y: annual mammogram and breast MRI screening with contrast  ・Discuss the option of risk-reducing mastectomy | | ・Starting at age 35y: Breast self-exam, clinical breast exam, every year  ・ (men with gynecomastia) Starting at age 50y: annual mammogram |
| Ovarian Cancer | 1.6 %  (Women) | 17-18 % | ― | ・Starting after the age 35-40y: Recommend risk-reducing salpingo-oophorectpmy (RRSO)  ・Starting after the age 30-35y: Consider transvaginal ultrasound combined with serum CA-125 | | ― |
| Prostate Cancer | 10.8 ％  (Men) | ― | 7.5-15 ％* | ― | | ・Starting at age 40y: consider prostate cancer screening (PSA) |
| Pancreatic Cancer | 2.6 %  (Men and Women) | 5-10 % | | ・Consider screening using MRI and/or endoscopic ultrasound. | | |

*　For under 65y to 70y carriers, it is a 7.3 to 8.6 fold increased risk compared to the general population^8,9)^ .

※It is important to note that not all carriers who inherit genetic changes in *BRCA2* develop cancer.

※The likelihood of being symptomatic if carrying a genetic change and recommended management is based on the Japanese guideline^3)^ and the National Comprehensive Cancer Network guideline^9)^. However, these recommendations are not always implemented in Japan. Please contact specialists and Genetic Services if management is provided at the hospital.

※Table1 is based on information as of 2021. Recommendations may be revised as research progresses.

【References】

- Breast and ovarian cancer predisposition due to de novo BRCA1 and BRCA2 mutations. Oncogene, 2016; 35(10): 1324. [ref. 1]
- 国立がん研究センターがん情報サービス「累積がん罹患リスク (2018年データ)」https://ganjoho.jp/reg_stat/statistics/stat/summary.html [ref. 2]
- 日本遺伝性乳癌卵巣癌総合診療精度機構. 遺伝性乳癌卵巣癌 (HBOC) 診療ガイドライン2021年版. (2021年7月) [ref. 3]
- Meta-analysis of BRCA2 and BRCA2 penetrance. J Clin Oncol, 2007; 25(11): 1329. [ref. 4]
- Risks of Breast, Ovarian, and Contralateral Breast Cancer for BRCA2 and BRCA2 Mutation Carriers. JAMA, 2017; 317(23): 2402. [ref. 5]
- Breast cancer risk among male BRCA1 and BRCA2 mutation carriers. J Natl Cancer Ins, 2007; 99(23): 1811. [ref. 6]
- Risk of breast cancer in male BRCA2 carriers. J Med Genet, 2010; 47(10): 710. [ref. 7]
- Germline BRCA mutations are associated with higher risk of nodal 11 involvement, distant metastasis, and poor survival outcomes in prostate cancer. J Clin Oncol, 2013; 31(14): 1748. [ref. 8]
- Breast Cancer Linkage Consortium Cancer risks in BRCA2 mutation carriers. J Natl Cancer Inst, 1999; 91(15): 1310. [ref. 9]
- NCCN Guidelines® Genetic/Familial High-Risk Assessment: Breast, Ovarian, and Pancreatic. ver.1.2022 (2021.8.11) [ref. 10]
- GeneReviews Japan: BRCA1およびBRCA2関連遺伝性乳癌卵巣癌. ver.2016.12.25 (minor revision; 2017.2.20)
- ClinGen Actionability Reports: BRCA1, BRCA2 Adult. ver.1.1.3 (2020.9.15)

supervising editor： Research Group for the Research Project on Ethical, Legal, and Social Issues Supported by the Health, Labour and Welfare Sciences Research Grants “Extraction of ethical and social issues and improvement of social environment toward the realization of a society where people can benefit from genome medicine without anxiety,” Actionability Working Group-Japan

Edited by MONSTAR-SCREEN-2　Medical Genetic Office

***BRIP1***

**Genetic Change and Hereditary Cancer Syndrome**

- - If genetic changes at birth are responsible for the hereditary cancer syndrome, the patient is considered to be predisposed to cancer.
  - In general, 5-10% of cancers are caused by genetic changes at birth.
  - Cancer susceptibility may be shared by relatives such as parents, children, siblings.
  - Understanding genetic risks allows better health management.
  - A blood test confirms whether the changes observed in genomic testing (tumor profiling) are hereditary.

**What Is *BRIP1* Related To?**

- *BRIP1* has been shown to be associated with an increased risk of cancer (see Table 1).

**The Benefit of Confirmatory Testing**

- It is important to know the cancer risk for prevention and early detection of cancer.


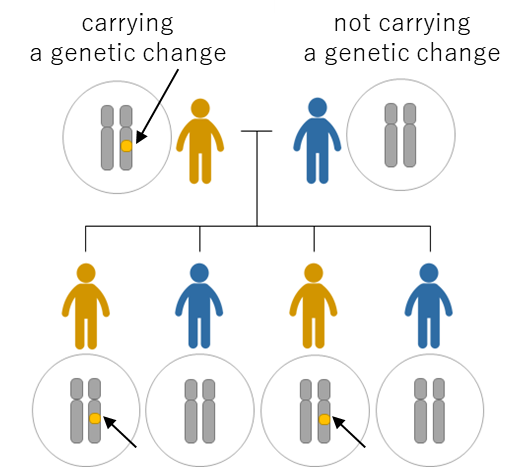
 **Risk of Family Members**

- There are two copies of *BRIP1*. One and another copy is inherited from the mother, and the other from the father, respectively.
- Each child, brother, sister, and parent have a 50% chance of inheriting genetic changes in *BRIP1*. Genetic testing can be performed to examine if similar changes are exhibited.
- Aunts, uncles, and cousins may share genetic changes and risks.
- Sharing your genetic information with your relatives may help them manage their health.

※ It is possible that the *BRIP1* gene change is not inherited from either parent but is a *de* *novo* change. Please consult your genetic specialist for information about the impact of such a case on your family.

**What Is Genetic Counseling?**

Genetic Counseling provides information on how genetic conditions may affect you and/or your family　and how to manage it. Please feel free to contact your genetic specialist if you have any relevant questions. ※Genetic testing(s) are not always covered by public health insurance in Japan. Genetic testing and follow-up care for healthy relatives who are unaffected by cancer are not covered by insurance. Please ask Genetic Services for details.

Table 1. Lifetime Risk and Recommended Management

|  | Japanese | Carrier of *BRIP1* pathogenic / likely pathogenic variant | | | |
| --- | --- | --- | --- | --- | --- |
|  | Lifetime risk^1)^ | Lifetime risk | | Recommended management | |
|  |  | Women^2)^ | Men | Women^2)^ | Men |
| Ovarian Cancer | 1.6% (Women) | >10 % | ― | ・Starting after the age 45-50y: Consider risk-reducing salpingo-oophorectpmy (RRSO)  ※Risks are considered to vary depending on family and medical history. | ― |
| Breast Cancer | 10.9 ％ (Women) | Potential for increased risk | ― | ※Risks are considered to vary depending on family and medical history. | ― |

* The recommended management in Table 1 is not specifically presented in Japan. Please contact the genetic specialist at your institution for further details.

※It is important to note that not all carriers who inherit genetic changes in *BRIP1* develop cancer.

※The likelihood of being symptomatic if carrying a genetic change and recommended management is based on the National Comprehensive Cancer Network guideline^2)^.

※Table1 is based on information as of 2021. Recommendations may be revised as research progresses.

【References】

- 国立がん研究センターがん情報サービス「累積がん罹患リスク (2018年データ)」https://ganjoho.jp/reg_stat/statistics/stat/summary.html [ref. 1]
- NCCN Guidelines® Genetic/Familial High-Risk Assessment: Breast, Ovarian, and Pancreatic. ver.1.2022 (2021.8.11) [ref. 2]
- ClinGen Actionability Reports: BRIP1, RAD51C, RAD51D Adult. ver.1.1.1 (2020.7.13)

supervising editor： Research Group for the Research Project on Ethical, Legal, and Social Issues Supported by the Health, Labour and Welfare Sciences Research Grants “Extraction of ethical and social issues and improvement of social environment toward the realization of a society where people can benefit from genome medicine without anxiety,” Actionability Working Group-Japan

Edited by MONSTAR-SCREEN-2　Medical Genetic Office

***CDH1***

**Genetic Change and Hereditary Cancer Syndrome**

- - If genetic changes at birth are responsible for the hereditary cancer syndrome, the patient is considered to be predisposed to cancer.
  - In general, 5-10% of cancers are caused by genetic changes at birth.
  - Cancer susceptibility may be shared by relatives such as parents, children, siblings.
  - Understanding genetic risks allows better health management.
  - A blood test confirms whether the changes observed in genomic testing (tumor profiling) are hereditary.

**What Is this *CDH1* Related To?**

- Genetic changes in the *CDH1* gene causes Hereditary Diffuse Gastric Cancer (see Table 1).

**The Benefit of Confirmatory Testing**

- It is important to know the cancer risk for prevention and early detection of cancer.


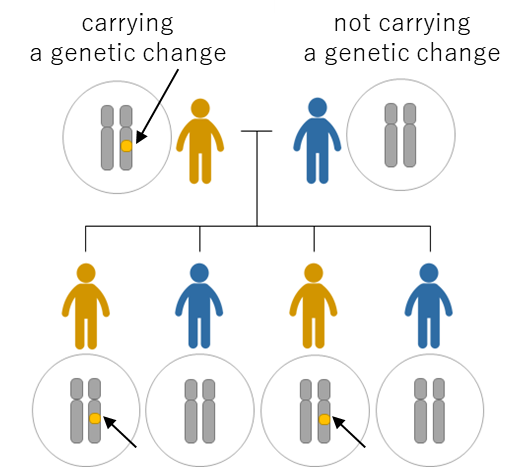
 **Risk of Family Members**

- There are two copies of *CDH1*. One and another copy is inherited from the mother, and the other from the father, respectively.
- Each child, brother, sister, and parent have a 50% chance of inheriting genetic changes in *CDH1*. Genetic testing can be performed to examine if similar changes are exhibited.
- Aunts, uncles, and cousins may share genetic changes and risks.
- Sharing your genetic information with your relatives may help them manage their health.

※ It is possible that the *CDH1* gene change is not inherited from either parent but is a *de* *novo* change. Please consult your genetic specialist for information about the impact of such a case on your family.

**What Is Genetic Counseling?**

Genetic Counseling provides information on how genetic conditions may affect you and/or your family　and how to manage it. Please feel free to contact your genetic specialist if you have any relevant questions. ※Genetic testing(s) are not always covered by public health insurance in Japan. Genetic testing and follow-up care for healthy relatives who are unaffected by cancer are not covered by insurance. Please ask Genetic Services for details.

Table 1. Lifetime Risk and Recommended Management

|  | Japanese | Carrier of *CDH1* pathogenic / likely pathogenic variant | | | |
| --- | --- | --- | --- | --- | --- |
|  | Lifetime risk^1)^ | Lifetime risk | | Recommended management | |
|  |  | Women^2,3)^ | Men^2,3)^ | Women^2,3)^ | Men^2,3)^ |
| Breast Cancer | 10.9 ％ (Women) | 39-52 % | ― | ・Starting at age 30y：annual mammogram and breast MRI screening with contrast* | ― |
| Gastric Cancer | 10.3 ％  (Men)  4.7 ％ (Women) | Until 80y  83 % | Until 80y  67 % | ・Upper endoscopy with multiple random biopsies every 6-12 months*  ・Starting between ages 18-40y: Prophylactic total gastrectomy (※Depending on the age of onset of family members)*  ・Risks are considered to vary depending on family and medical history. | |

* The recommended management in Table 1 is not specifically presented in Japan. Please contact the genetic specialist at your institution for further details.

※It is important to note that not all carriers who inherit genetic changes in *CDH1* develop cancer.

※The likelihood of being symptomatic if carrying a genetic change and recommended management is based on the National Comprehensive Cancer Network guidelines ^2,3)^.

※Table1 is based on information as of 2021. Recommendations may be revised as research progresses.

【References】

- 国立がん研究センターがん情報サービス「累積がん罹患リスク (2018年データ)」<https://ganjoho.jp/reg_stat/statistics/stat/summary.html> [ref. 1]
- NCCN Guidelines® Genetic/Familial High-Risk Assessment: Breast, Ovarian, and Pancreatic. ver.1.2022 (2021.8.11) [ref. 2]
- NCCN Guidelines® Gastric cancer. ver.2.2022 (2022.1.11) [ref. 3]
- GeneReviews®: Hereditary Diffuse Gastric Cancer. ver.2018.3.22.
- ClinGen Actionability Reports: CDH1 Adult. ver.1.1.3 (2021.9.3)

supervising editor： Research Group for the Research Project on Ethical, Legal, and Social Issues Supported by the Health, Labour and Welfare Sciences Research Grants “Extraction of ethical and social issues and improvement of social environment toward the realization of a society where people can benefit from genome medicine without anxiety,” Actionability Working Group-Japan

Edited by MONSTAR-SCREEN-2　Medical Genetic Office

***CDK4***

**Genetic Change and Hereditary Cancer Syndrome**

- - If genetic changes at birth are responsible for the hereditary cancer syndrome, the patient is considered to be predisposed to cancer.
  - In general, 5-10% of cancers are caused by genetic changes at birth.
  - Cancer susceptibility may be shared by relatives such as parents, children, siblings.
  - Understanding genetic risks allows better health management.
  - A blood test confirms whether the changes observed in genomic testing (tumor profiling) are hereditary.

**What Is *CDK4* Related To?**

- *CDK4* has been shown to be associated with an increased risk of cancer^1,2)^ (see Table 1).

**The Benefit of Confirmatory Testing**

- It is important to know the cancer risk for prevention and early detection of cancer.


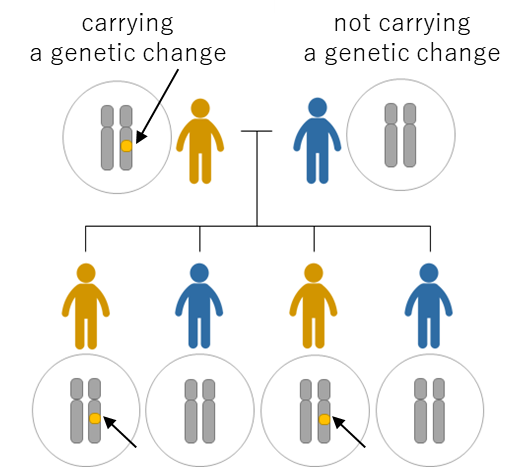
 **Risk of Family Members**

- There are two copies of *CDK4*. One and another copy is inherited from the mother, and the other from the father, respectively.
- Each child, brother, sister, and parent have a 50% chance of inheriting genetic changes in *CDK4*. Genetic testing can be performed to examine if similar changes are exhibited.
- Aunts, uncles, and cousins may share genetic changes and risks.
- Sharing your genetic information with your relatives may help them manage their health.

※ It is possible that the *CDK4* gene change is not inherited from either parent but is a *de* *novo* change. Please consult your genetic specialist for information about the impact of such a case on your family.

**What Is Genetic Counseling?**

Genetic Counseling provides information on how genetic conditions may affect you and/or your family　and how to manage it. Please feel free to contact your genetic specialist if you have any relevant questions. ※Genetic testing(s) are not always covered by public health insurance in Japan. Genetic testing and follow-up care for healthy relatives who are unaffected by cancer are not covered by insurance. Please ask Genetic Services for details.

Table 1. Lifetime Risk and Recommended Management

|  | Japanese | Carrier of *CDK4* pathogenic / likely pathogenic variant | | | |
| --- | --- | --- | --- | --- | --- |
|  | Lifetime risk^3,4)^ | Lifetime risk | | Recommended management | |
|  |  | Women^1,2)^ | Men^1,2)^ | Women^5,6)^ | Men^5,6)^ |
| Melanoma* | 0.1 % (Men)  0.1 % (Women) | Potential increased risk | | ・Comprehensive skin examination by a dermatologist, including total body skin, scalp, oral mucosa, and genitals every 6 months (if the nevus is stable: every 12 month) **  ・Monthly nevus self-examination** | |
| Pancreatic Cancer | 2.6 %  (Men and Women) | Potential increased risk | | ・Starting at age 40y or 10 years earlier than the youngest onset of pancreatic cancer in the family: contrast-enhanced MRI and/or endoscopic ultrasound** | |

* The annual prevalence of malignant melanoma is reported to be 24.3 in 100,000 people per for Caucasians and 1.7 in 100,000 people for Asians^6)^, and the risk is considered to vary according to race, region, and other genetic factors.

** The recommended management in Table 1 is not specifically presented in Japan. Please contact the genetic specialist at your institution for further details.

※It is important to note that not all carriers who inherit genetic changes in *CDK4* develop cancer.

※The likelihood of being symptomatic if carrying a genetic change and recommended management is based on international data.^1,2,5,6)^

※Table1 is based on information as of 2021. Recommendations may be revised as research progresses.

【References】

- Hereditary melanoma: update on syndrome and management -Genetics of familial atypical multiple mole melanoma syndrome. J Am Acad Dernatol, 2016; 74(3): 395. [ref. 1]
- Genotype-phenotype relationships in U.S. melanoma-prone families with *CDKN2A* and *CDK4* mutations. J Natl Cancer Inst, 2000; 92(12): 1006. [ref. 2]
- 厚生労働省健康局がん・疾病対策課. 平成30年全国がん登録 罹患数・率報告 2018 [ref. 3]
- 国立がん研究センターがん情報サービス「累積がん罹患リスク (2018年データ)」 <https://ganjoho.jp/reg_stat/statistics/stat/summary.html> [ref. 4]
- ClinGen Actionability Reports: CDKN2A Adult. ver.1.2.1 (2020.8.19) [ref. 5]
- NCCN Guidelines® Genetic/Familial High-Risk Assessment: Breast, Ovarian, and Pancreatic. ver.1.2022 (2021.8.11) [ref. 6]
- 日本皮膚科学会. 皮膚悪性腫瘍ガイドライン第 3 版 メラノーマ診療ガイドライン 2019. 日皮会誌, 2019; 129(9): 1759. [ref. 7]

supervising editor： Research Group for the Research Project on Ethical, Legal, and Social Issues Supported by the Health, Labour and Welfare Sciences Research Grants “Extraction of ethical and social issues and improvement of social environment toward the realization of a society where people can benefit from genome medicine without anxiety,” Actionability Working Group-Japan

Edited by MONSTAR-SCREEN-2　Medical Genetic Office

***CDKN2A***

**Genetic Change and Hereditary Cancer Syndrome**

- - If genetic changes at birth are responsible for the hereditary cancer syndrome, the patient is considered to be predisposed to cancer.
  - In general, 5-10% of cancers are caused by genetic changes at birth.
  - Cancer susceptibility may be shared by relatives such as parents, children, siblings.
  - Understanding genetic risks allows better health management.
  - A blood test confirms whether the changes observed in genomic testing (tumor profiling) are hereditary.

**What Is *CDKN2A* Related To?**

- Genetic change in the *CDKN2A* gene causes Melanoma-pancreatic cancer syndrome (see Table 1).

**The Benefit of Confirmatory Testing**

- It is important to know the cancer risk for prevention and early detection of cancer.


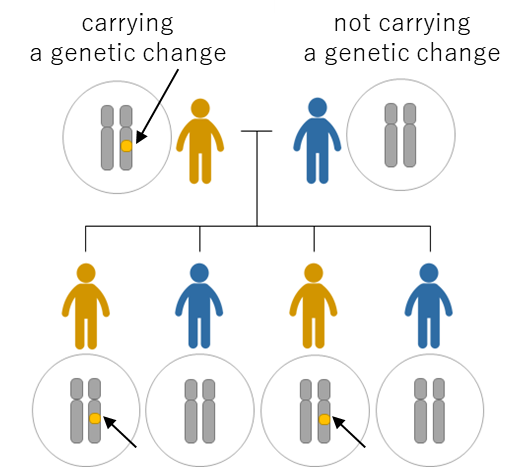
 **Risk of Family Members**

- There are two copies of *CDKN2A*. One and another copy is inherited from the mother, and the other from the father, respectively.
- Each child, brother, sister, and parent have a 50% chance of inheriting genetic changes in *CDKN2A*. Genetic testing can be performed to examine if similar changes are exhibited.
- Aunts, uncles, and cousins may share genetic changes and risks.
- Sharing your genetic information with your relatives may help them manage their health.

※ It is possible that the *CDKN2A* gene change is not inherited from either parent but is a *de* *novo* change. Please consult your genetic specialist for information about the impact of such a case on your family.

**What Is Genetic Counseling?**

Genetic Counseling provides information on how genetic conditions may affect you and/or your family　and how to manage it. Please feel free to contact your genetic specialist if you have any relevant questions. ※Genetic testing(s) are not always covered by public health insurance in Japan. Genetic testing and follow-up care for healthy relatives who are unaffected by cancer are not covered by insurance. Please ask Genetic Services for details.

Table 1. Lifetime Risk and Recommended Management

|  | Japanese | Carrier of *CDKN2A* pathogenic / likely pathogenic variant | | | |
| --- | --- | --- | --- | --- | --- |
|  | Lifetime risk^1,2)^ | Lifetime risk | | Recommended management | |
|  |  | Women^3,4)^ | Men^3,4)^ | Women^3,4,5)^ | Men^3,4,5)^ |
| Pancreatic Cancer | 2.6 %  （Men and Women） | >15 %  （20-47 times higher risk than general population） | | ・Starting at age 40y or 10 years earlier than the youngest onset of pancreatic cancer in the family: MRI and/or endoscopic ultrasound** | |
| Malignant Melanoma* | 0.1 % (Men)  0.1 % (Women) | 28-76％ | | ・Comprehensive skin examination by a dermatologist, including total body skin, scalp, oral mucosa, and genitals every 6 months (if the nevus is stable: every 12 month) **  ・Monthly nevus self-examination** | |

* The annual prevalence of malignant melanoma is reported to be 24.3 in 100,000 people for Caucasians and 1.7 in 100,000 people for Asians^6)^, and the risk is considered to vary according to race, region, and other genetic factors.

** The recommended management in Table 1 is not specifically presented in Japan. Please contact the genetic specialist at your institution for further details.

※It is important to note that not all carriers who inherit genetic changes in *CDKN2A* develop cancer.

※The likelihood of being symptomatic if carrying a genetic change and recommended management is based on the international data^3,4,5)^.

※Table1 is based on information as of 2021. Recommendations may be revised as research progresses.

【References】

- 国立がん研究センターがん情報サービス「累積がん罹患リスク (2018年データ)」 <https://ganjoho.jp/reg_stat/statistics/stat/summary.html> [ref. 1]
- 厚生労働省健康局がん・疾病対策課 平成30年全国がん登録 罹患数・率報告 2018 [ref. 2]
- NCCN Guidelines® Genetic/Familial High-Risk Assessment: Breast, Ovarian, and Pancreatic. ver.1.2022 (2021.8.11) [ref. 3]
- NCCN Guidelines®: Pancreatic Adenocarcinoma. ver.2.2021 (2021.2.25) [ref. 4]
- ClinGen Actionability Reports: CDKN2A Adult. ver.1.2.1 (2020.8.19) [ref. 5]
- 日本皮膚科学会. 皮膚悪性腫瘍ガイドライン第 3 版 メラノーマ診療ガイドライン 2019. 日皮会誌, 2019; 129(9):1759. [ref. 6]

supervising editor： Research Group for the Research Project on Ethical, Legal, and Social Issues Supported by the Health, Labour and Welfare Sciences Research Grants “Extraction of ethical and social issues and improvement of social environment toward the realization of a society where people can benefit from genome medicine without anxiety,” Actionability Working Group-Japan

Edited by MONSTAR-SCREEN-2　Medical Genetic Office

***CHEK2***

**Genetic Change and Hereditary Cancer Syndrome**

- - If genetic changes at birth are responsible for the hereditary cancer syndrome, the patient is considered to be predisposed to cancer.
  - In general, 5-10% of cancers are caused by genetic changes at birth.
  - Cancer susceptibility may be shared by relatives such as parents, children, siblings.
  - Understanding genetic risks allows better health management.
  - A blood test confirms whether the changes observed in genomic testing (tumor profiling) are hereditary.

**What Is *CHEK2* Related To?**

- *CHEK2* has been shown to be associated with an increased risk of cancer^1,2)^ (see Table 1).

**The Benefit of Confirmatory Testing**

- It is important to know the cancer risk for prevention and early detection of cancer.


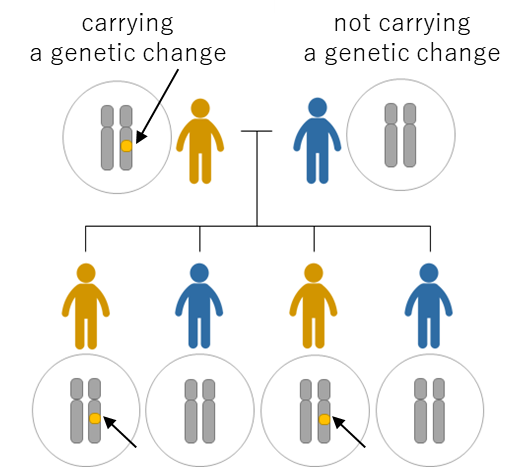
 **Risk of Family Members**

- There are two copies of *CHEK2*. One and another copy is inherited from the mother, and the other from the father, respectively.
- Each child, brother, sister, and parent have a 50% chance of inheriting genetic changes in *CHEK2*. Genetic testing can be performed to examine if similar changes are exhibited.
- Aunts, uncles, and cousins may share genetic changes and risks.
- Sharing your genetic information with your relatives may help them manage their health.

※ It is possible that the *CHEK2* gene change is not inherited from either parent but is a *de* *novo* change. Please consult your genetic specialist for information about the impact of such a case on your family.

**What Is Genetic Counseling?**

Genetic Counseling provides information on how genetic conditions may affect you and/or your family　and how to manage it. Please feel free to contact your genetic specialist if you have any relevant questions. ※Genetic testing(s) are not always covered by public health insurance in Japan. Genetic testing and follow-up care for healthy relatives who are unaffected by cancer are not covered by insurance. Please ask Genetic Services for details.

Table 1. Lifetime Risk and Recommended Management

|  | Japanese | Carrier of *CHEK2* pathogenic / likely pathogenic variant | | | | |
| --- | --- | --- | --- | --- | --- | --- |
|  | Lifetime risk^1)^ | | Lifetime risk | | Recommended management | |
|  |  |  | Women^2,3)^ | Men^2,3)^ | Women^2,3)^ | Men^2,3)^ |
| Breast Cancer | 10.9 ％  (Women) | | 15-40 ％ | ― | ・Starting at age 40y: annual mammogram and breast MRI screening with contrast* | ― |
| Colorectal Cancer | 8.0 % (Women)　10.2 % (Men) | | Potential for increases risk | | ・Starting at age 40y (or 10y prior to age of first-degree relative’s age at colorectal cancer diagnosis.): colonoscopy every 5 years* | |

* The recommended management in Table 1 is not specifically presented in Japan. Please contact the genetic specialist at your institution for further details.

※It is important to note that not all carriers who inherit genetic changes in *CHEK2* develop cancer.

※The likelihood of being symptomatic if carrying a genetic change and recommended management is based on the National Comprehensive Cancer Network guidelines^2,3)^. However, these recommendations are not always implemented in Japan. Please contact specialists and Genetic Services if management is provided at the hospital.

※Table1 is based on information as of 2021. Recommendations may be revised as research progresses.

【References】

- 国立がん研究センターがん情報サービス「累積がん罹患リスク (2018年データ)」https://ganjoho.jp/reg_stat/statistics/stat/summary.html [ref. 1]
- NCCN Guidelines® Genetic/Familial High-Risk Assessment: Breast, Ovarian, and Pancreatic. ver.1.2022 (2021.8.11) [ref. 2]
- NCCN Guidelines® Genetic/Familial High-Risk Assessment: Colorectal. ver.1.2021 (2021.5.11) [ref. 3]
- ClinGen Actionability Reports: ATM, CHEK2 Adult. ver.1.1.1 (2020.4.16)

supervising editor： Research Group for the Research Project on Ethical, Legal, and Social Issues Supported by the Health, Labour and Welfare Sciences Research Grants “Extraction of ethical and social issues and improvement of social environment toward the realization of a society where people can benefit from genome medicine without anxiety,” Actionability Working Group-Japan

Edited by MONSTAR-SCREEN-2　Medical Genetic Office

***EPCAM***

**Genetic Change and Hereditary Cancer Syndrome**

- - If genetic changes at birth are responsible for the hereditary cancer syndrome, the patient is considered to be predisposed to cancer.
  - In general, 5-10% of cancers are caused by genetic changes at birth.
  - Cancer susceptibility may be shared by relatives such as parents, children, siblings.
  - Understanding genetic risks allows better health management.
  - A blood test confirms whether the changes observed in genomic testing (tumor profiling) are hereditary.

**What Is *EPCAM* Related To?**

- Genetic changes in the *EPCAM* gene causes Lynch Syndrome (see Table 1).

**The Benefit of Confirmatory Testing**

- It is important to know the cancer risk for prevention and early detection of cancer.


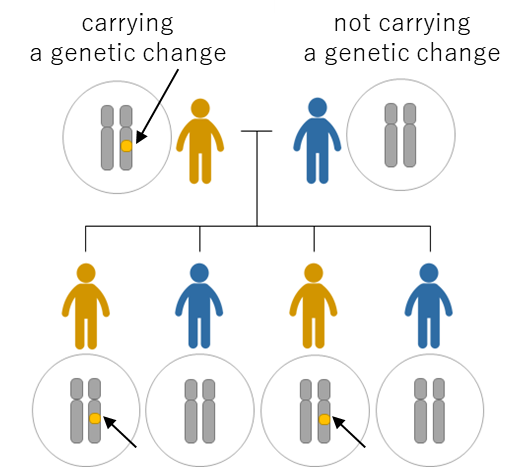
 **Risk of Family Members**

- There are two copies of *EPCAM*. One and another copy is inherited from the mother, and the other from the father, respectively.
- Each child, brother, sister, and parent have a 50% chance of inheriting genetic changes in *EPCAM*. Genetic testing can be performed to examine if similar changes are exhibited.
- Aunts, uncles, and cousins may share genetic changes and risks.
- Sharing your genetic information with your relatives may help them manage their health.

※ It is possible that the *EPCAM* gene change is not inherited from either parent but is a *de* *novo* change. Please consult your genetic specialist for information about the impact of such a case on your family.

**What Is Genetic Counseling?**

Genetic Counseling provides information on how genetic conditions may affect you and/or your family　and how to manage it. Please feel free to contact your genetic specialist if you have any relevant questions. ※Genetic testing(s) are not always covered by public health insurance in Japan. Genetic testing and follow-up care for healthy relatives who are unaffected by cancer are not covered by insurance. Please ask Genetic Services for details.

Table 1. Lifetime Risk and Recommended Management

|  | Japanese | Carrier of *EPCAM* pathogenic / likely pathogenic variant | | | |
| --- | --- | --- | --- | --- | --- |
|  | Lifetime risk^1)^ | Risk up to 80y | | Recommended management | |
|  |  | Women^２)^ | Men^２)^ | Women^３)^ | Men^３)^ |
| Colorectal Cancer | 10.2 ％ (Men)  8.0 ％ (Women) | 33-52 % | | ・Starting at ages 20-25y: colonoscopy every 1-2 years | |
| Endometrial Cancer | 2.0% (Women) | 21-57 % | ― | ・Starting at ages 30-35y: annual transvaginal ultrasound, endometrial biopsy, (CA-125 screening) | ― |
| Ovarian Cancer | 1.6% (Women) | 8-38 % | ― |  |  |
| Gastric Cancer | 10.3 ％ (Men)  4.7 ％ (Women) | 0.2-9 %* | | (Consider in those with a family history of gastric or duodenal cancer.)  ・Starting at ages 30-35y: Eradication of H.pylori  Upper endoscopy every 1-3 years | |
| Ureter and Renal Pelvis Cancer | 2.2 % (Men)  1.1 % (Women) | 2.2-28 % | | (Consider in those with a family history of urothelial cancer)  ・Starting at ages 30-35y: annual urine test (or urine cytology) | |
| Bladder Cancer | 2.1 % (Men)  0.7 % (Women) | 4.4-12.8 % | |  |  |

* The risk listed in Table 1 is based on the data from other countries. The risk of gastric cancer is higher in East Asian populations than that in Western populations ^4)^.　It has been reported the risk of gastric cancer in Japanese patients diagnosed with Lynch syndrome is 14.5-24% by the age of 60-70 years^5,6)^.

※It is important to note that not all carriers who inherit genetic changes in *EPCAM* develop cancer.

※The likelihood of being symptomatic if carrying genetic change and recommended management is based on the Japanese guideline^3)^ and the National Comprehensive Cancer Network guideline^2)^. However, these recommendations are not always implemented in Japan. Please contact specialists and Genetic Services if management is provided at the hospital.

※Table1 is based on information as of 2021. Recommendations may be revised as research progresses.

【References】

- 国立がん研究センターがん情報サービス「累積がん罹患リスク (2018年データ)」 <https://ganjoho.jp/reg_stat/statistics/stat/summary.html> [ref. 1]
- NCCN Guidelines® Genetic/Familial High-Risk Assessment: Colorectal. ver.1.2021 (2021.5.11) [ref. 2]
- 大腸癌研究会. 遺伝性大腸癌診療ガイドライン 2020年版. (2020年4月) [ref. 3]
- Managing gastric cancer risk in lynch syndrome: controversies and recommendations. Fam Cancer, 2021 (online ahead of print). [ref. 4]
- Tumor development in Japanese patients with Lynch syndrome. PLoS One, 2018; 13(4): e0195572. [ref. 5]
- Comparison of clinical features between suspected familial colorectal cancer type X and Lynch syndrome in Japanese patients with colorectal cancer: a cross-sectional study conducted by the Japanese Society for Cancer of the Colon and Rectum. Jpn J Clin Oncol, 2015; 45(2): 153. [ref. 6]
- GeneReviews Japan: リンチ症候群. ver.2021.4.21
- ClinGen Actionability Reports: MLH1, MSH2, MSH6, PMS2, EPCAM Adult. ver.1.1.2 (2021.9.15)

supervising editor： Research Group for the Research Project on Ethical, Legal, and Social Issues Supported by the Health, Labour and Welfare Sciences Research Grants “Extraction of ethical and social issues and improvement of social environment toward the realization of a society where people can benefit from genome medicine without anxiety,” Actionability Working Group-Japan

Edited by MONSTAR-SCREEN-2　Medical Genetic Office

***FH***

**Genetic Change and Hereditary Cancer Syndrome**

- - If genetic changes at birth are responsible for the hereditary cancer syndrome, the patient is considered to be predisposed to cancer.
  - In general, 5-10% of cancers are caused by genetic changes at birth.
  - Cancer susceptibility may be shared by relatives such as parents, children, siblings.
  - Understanding genetic risks allows better health management.
  - A blood test confirms whether the changes observed in genomic testing (tumor profiling) are hereditary.

**What Is *FH* Related To?**

- Genetic changes in the *FH* gene causes Hereditary Leiomyomatosis and Renal Cell Cancer (see Table 1).

**The Benefit of Confirmatory Testing**

- It is important to know the cancer risk for prevention and early detection of cancer.


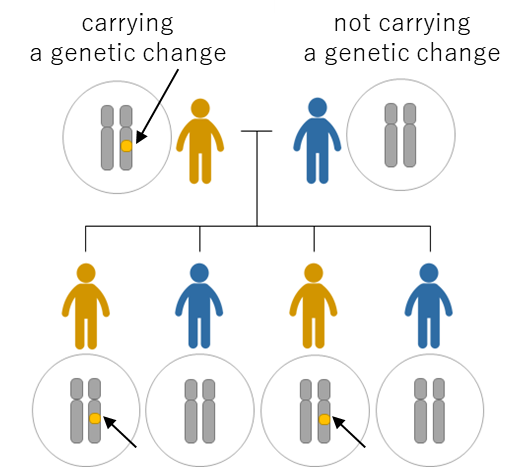
 **Risk of Family Members**

- There are two copies of *FH*. One and another copy is inherited from the mother, and the other from the father, respectively.
- Each child, brother, sister, and parent have a 50% chance of inheriting genetic changes in *FH*. Genetic testing can be performed to examine if similar changes are exhibited.
- Aunts, uncles, and cousins may share genetic changes and risks.
- Sharing your genetic information with your relatives may help them manage their health.

※ It is possible that the *FH* gene change is not inherited from either parent, but is a *de* *novo* change. Please consult your genetic specialist for information about the impact of such a case on your family.

**What Is Genetic Counseling?**

Genetic Counseling gives you information about how genetic conditions might affect you and/or your family. Please feel free to contact your genetic specialist if you have any questions. ※Genetic testing(s) are not always covered by public health insurance in Japan. Genetic testing and follow-up care for healthy relatives, unaffected by cancer, will not be covered by the insurance. Please ask Genetic Services for details.

Table 1. Lifetime Risk and Recommended Management

|  | Japanese | Carrier of *FH* pathogenic / likely pathogenic variant | | | |
| --- | --- | --- | --- | --- | --- |
|  | Lifetimerisk^1,2,3,4)^ | Lifetime risk | | Recommended management | |
|  |  | Women^5,6)^ | Men^5,6)^ | Women^5,7,8,9)^ | Men^5,7,8,9)^ |
| Cutaneous Leiomyoma  Cutaneous Leiomyosarcoma | ※Malignant soft tissue sarcomas occur at 40 cases/million/year | (Cutaneous leiomyoma）  76 % | | Dermatologic examination every 1-2 years* | |
| Uterine Leiomyomas  Uterine Leiomyosarcoma | (Endometrial cancer)  ２％ | （Uterine leiomyomas）77 % | ― | ・Starting at age 20y: annual gynecologic examination (start earlier before 20y if symptomatic) * | ― |
| Renal Cell Cancer | (Renal cancer (except renal pelvis cancer))  1.2% (Men)  0.4% (Women) | 10-16 %  ※Estimates ranging from 1.7% to 5.8% also exist | | ・Starting at ages 8-10y: annual renal ultrasound and MRI* | |
| Paraganglioma/  Pheochromocytoma | ※Approximately  1,500 /year nationwide | Potential for increased risk | | ・Consider baseline whole-body MRI and plasma-free metanephrines for measurement with a relevant personal/family history of paraganglioma/pheochromocytoma* | |

* The recommended management in Table 1 is not specifically presented in Japan. Please contact the genetic specialist at your institution for further details.

※It is important to note that not all carriers who inherit genetic changes in *FH* develop cancer.

※The likelihood of being symptomatic if carrying a genetic change and recommended management is based on the International data^4)^.

※Table1 is based on information as of 2021. Recommendations may be revised as research progresses.

【References】

- 日本整形外科学会. 軟部腫瘍診療ガイドライン2020 改訂第3版. (2020年7月) [ref. 1]
- 国立がん研究センターがん情報サービス「累積がん罹患リスク (2018年データ)」 <https://ganjoho.jp/reg_stat/statistics/stat/summary.html> [ref. 2]
- 厚生労働省健康局がん・疾病対策課. 平成30年全国がん登録 罹患数・率報告 2018. [ref. 3]
- 国立がん研究センター希少がんセンター: パラガングリオーマ <https://www.ncc.go.jp/jp/rcc/about/paraganguriouma/index.html> [ref. 4]
- ClinGen Actionability Reports: FH Adult. ver.1.2.1 (2020.4.27) [ref. 5]
- Estimation of the carrier frequency of fumarase hydratase alterations and implications for kidney cancer risk in hereditary leiomyomatosis and renal cancer. Cancer, 2020; 126(16): 3657. [ref. 6]
- NCCN Guidelines®: Kidney Cancer. ver.4.2022 (2021.12.21) [ref. 7]
- GeneReviews Japan: *FH*腫瘍易罹患性症候群. ver.2021.5.1 [ref. 8]
- 日本内分泌学会. 褐色細胞腫・パラガングリオーマ診療ガイドライン2018. (2018年7月) [ref. 9]

supervising editor： Research Group for the Research Project on Ethical, Legal, and Social Issues Supported by the Health, Labour and Welfare Sciences Research Grants “Extraction of ethical and social issues and improvement of social environment toward the realization of a society where people can benefit from genome medicine without anxiety,” Actionability Working Group-Japan

Edited by MONSTAR-SCREEN-2　Medical Genetic Office

***FLCN***

**Genetic Change and Hereditary Cancer Syndrome**

- - If genetic changes at birth are responsible for the hereditary cancer syndrome, the patient is considered to be predisposed to cancer.
  - In general, 5-10% of cancers are caused by genetic changes at birth.
  - Cancer susceptibility may be shared by relatives such as parents, children, siblings.
  - Understanding genetic risks allows better health management.
  - A blood test confirms whether the changes observed in genomic testing (tumor profiling) are hereditary.

**What Is *FLCN* Related To?**

- Genetic changes in the *FLCN* gene causes Birt-Hogg-Dubé Syndrome (see Table 1).

**The Benefit of Confirmatory Testing**

- It is important to know the cancer risk for prevention and early detection of cancer.


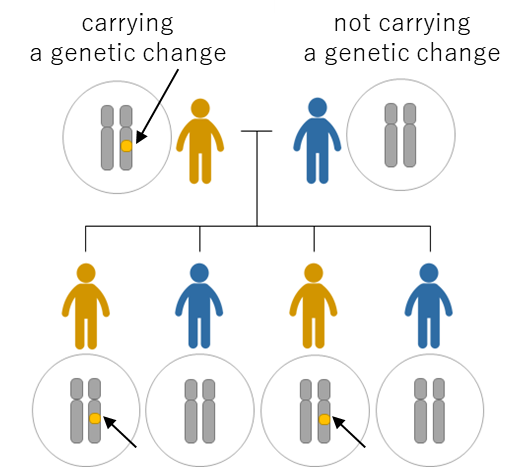
 **Risk of Family Members**

- There are two copies of *FLCN*. One and another copy is inherited from the mother, and the other from the father, respectively.
- Each child, brother, sister, and parent have a 50% chance of inheriting genetic changes in *FLCN*. Genetic testing can be performed to examine if similar changes are exhibited.
- Aunts, uncles, and cousins may share genetic changes and risks.
- Sharing your genetic information with your relatives may help them manage their health.

※ It is possible that the *FLCN* gene change is not inherited from either parent but is a *de* *novo* change. Please consult your genetic specialist for information about the impact of such as case on your family.

**What Is Genetic Counseling?**

Genetic Counseling provides information on how genetic conditions may affect you and/or your family　and how to manage it. Please feel free to contact your genetic specialist if you have any relevant questions. ※Genetic testing(s) are not always covered by public health insurance in Japan. Genetic testing and follow-up care for healthy relatives who are unaffected by cancer are not covered by insurance. Please ask Genetic Services for details.

Table 1. Lifetime Risk and Recommended Management

|  | Japanese | Carrier of *FLCN* pathogenic / likely pathogenic variant | | | |
| --- | --- | --- | --- | --- | --- |
|  | Lifetime risk^1)^ | Lifetime risk | | Recommended management | |
|  |  | Women^2)^ | Men^2)^ | Women^3,4)^ | Men^3,4)^ |
| Lung Cyst | Unknown | 89 % | | ・HRCT or CT of the chest for visualization of pulmonary cysts*: when signs of pneumothorax are present, appropriate follow-up should be carried out. | |
| Renal Tumor | (Renal cancer (except renal pelvis cancer))  1.2% (Men)  0.4% (Women) | 6.5-34 % | | ・Starting at age 20y: abdominal MRI (recommended) or CT every 3 years* | |
| Fibrofolliculoma | Unknown | 73-85 % | | ・Regular dermatologic examination* | |

* The recommended management in Table 1 is not specifically presented in Japan. Please contact the genetic specialist at your institution for further details.

※It is important to note that not all carriers who inherit genetic changes in *FLCN* develop cancer.

※The likelihood of being symptomatic if carrying a genetic change and recommended management is based on the International data^13)^.

※Table1 is based on information as of 2021. Recommendations may be revised as research progresses.

【References】

- 厚生労働省健康局がん・疾病対策課. 平成30年全国がん登録 罹患数・率報告 2018 [ref. 1]
- ClinGen Actionability Reports: FLCN Adult. ver.1.2.1 (2020.5.2) [ref. 2]
- GeneReviews Japan: Birt-Hogg-Dubé症候群. ver.2018.8.22 [ref. 3]
- NCCN Guidelines®: Kidney Cancer. ver.4.2022 (2021.12.21) [ref. 4]

supervising editor： Research Group for the Research Project on Ethical, Legal, and Social Issues Supported by the Health, Labour and Welfare Sciences Research Grants “Extraction of ethical and social issues and improvement of social environment toward the realization of a society where people can benefit from genome medicine without anxiety,” Actionability Working Group-Japan

Edited by MONSTAR-SCREEN-2　Medical Genetic Office

***HNF1A***

**What Is *HNF1A* Related To?**

- - *HNF1A* is a gene that has been shown to be associated with risk of endocrine disease.
  - A genetic change in the *HNF1A* gene causes Maturity-onset diabetes of the young type 3. (see Table 1)
  - A blood test will confirm whether the changes found in the genetic testing (tumor profiling) are hereditary.

**The Benefit of Confirmatory Testing**

- It is important to know the risk of diabetes for early detection.


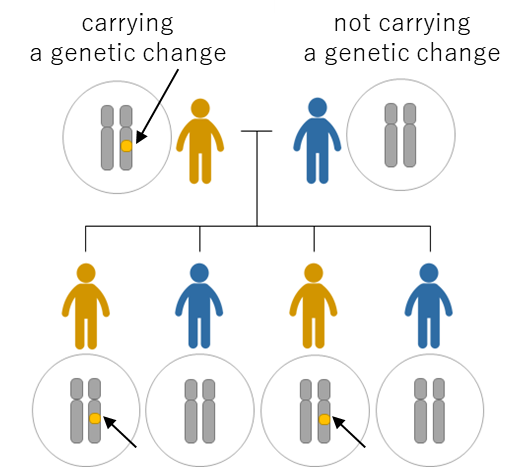
 **Risk of Family Members**

- There are two copies of *HNF1A*. One and another copy is inherited from the mother, and the other from the father, respectively.
- Each child, brother, sister, and parent have a 50% chance of inheriting genetic changes in *HNF1A*. Genetic testing can be performed to examine if similar changes are exhibited.
- Aunts, uncles, and cousins may share genetic changes and risks.
- Sharing your genetic information with your relatives may help them manage their health.

※ It is possible that the *HNF1A* gene change is not inherited from either parent but is a *de* *novo* change. Please consult your genetic specialist for information about the impact of such a case on your family.

**What Is Genetic Counseling?**

Genetic Counseling provides information on how genetic conditions may affect you and/or your family　and how to manage it. Please feel free to contact your genetic specialist if you have any relevant questions. ※Genetic testing(s) are not always covered by public health insurance in Japan. Genetic testing and follow-up care for healthy relatives who are unaffected by cancer are not covered by insurance. Please ask Genetic Services for details.

Table 1. Lifetime Risk and Recommended Management

|  | Japanese | Carrier of *HNF1A* pathogenic / likely pathogenic variant | | | |
| --- | --- | --- | --- | --- | --- |
|  | People with strongly suspected of diabetes* ^1)^ | Lifetime risk^2)^ | | Recommended management^3)^ | |
|  |  | Women | Men | Women | Men |
| Insulin Secretion Deficiency Type Diabetes | 19.7 % (Men)*  10.8 % (Women)* | Risk up to 25y 63%  Risk up to 55y 96% | | ・Standard of care for general diabetes (diet, exercise, pharmacotherapy) and management of complications | |

* Include diabetes types other than insulin secretion deficiency.

※It is important to note that not all carriers who inherit genetic changes in *HNF1A* are symptomatic.

※Please contact specialists and Genetic Services for further details.

【References】

- 厚生労働省. 令和元 年国民健康・栄養調査結果の概要. [ref. 1]
- GeneReviews®: Maturity-Onset Diabetes of the Young Overview. ver.2018.5.24 [ref. 2]
- Maturity-onset diabetes of the young as a model for elucidating the multifactorial origin of type 2 diabetes mellitus. J Diabetes Investig, 2018; 9(4): 704. [ref. 3]
- ClinGen Actionability Reports: HNF1A Adult. ver.1.2.1 (2020.4.27)

supervising editor： Research Group for the Research Project on Ethical, Legal, and Social Issues Supported by the Health, Labour and Welfare Sciences Research Grants “Extraction of ethical and social issues and improvement of social environment toward the realization of a society where people can benefit from genome medicine without anxiety,” Actionability Working Group-Japan

Edited by MONSTAR-SCREEN-2　Medical Genetic Office

***MAX***

**Genetic Change and Hereditary Cancer Syndrome**

- - If genetic changes at birth are responsible for the hereditary cancer syndrome, the patient is considered to be predisposed to cancer.
  - In general, 5-10% of cancers are caused by genetic changes at birth.
  - Cancer susceptibility may be shared by relatives such as parents, children, siblings.
  - Understanding genetic risks allows better health management.
  - A blood test confirms whether the changes observed in genomic testing (tumor profiling) are hereditary.

**What Is *MAX* Related To?**

- *MAX* has been shown to be associated with an increased risk of cancer (see Table 1).

**The Benefit of Confirmatory Testing**

- It is important to know the cancer risk for prevention and early detection of cancer.


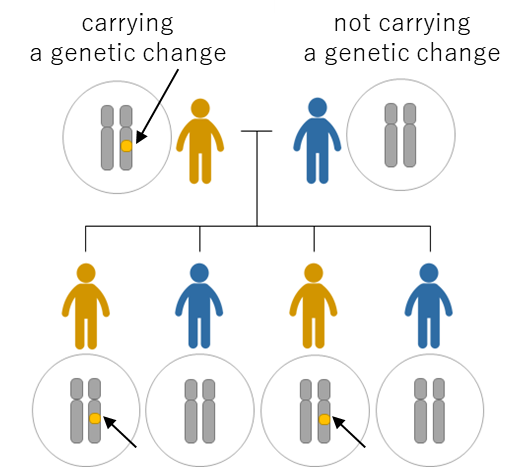
 **Risk of Family Members**

- There are two copies of *MAX*. One and another copy is inherited from the mother, and the other from the father, respectively.
- Each child, brother, sister, and parent have a 50% chance of inheriting genetic changes in *MAX*. Genetic testing can be performed to examine if similar changes are exhibited.
- Aunts, uncles, and cousins may share genetic changes and risks.
- Sharing your genetic information with your relatives may help them manage their health.

※ It is possible that the *MAX* gene change is not inherited from either parent but is a *de* *novo* change. Please consult your genetic specialist for information about the impact of such a case on your family.

**What Is Genetic Counseling?**

Genetic Counseling provides information on how genetic conditions may affect you and/or your family　and how to manage it. Please feel free to contact your genetic specialist if you have any relevant questions. ※Genetic testing(s) are not always covered by public health insurance in Japan. Genetic testing and follow-up care for healthy relatives who are unaffected by cancer are not covered by insurance. Please ask Genetic Services for details.

Table 1. Lifetime Risk and Recommended Management

|  | Japanese | Carrier of *MAX* pathogenic / likely pathogenic variant | | | |
| --- | --- | --- | --- | --- | --- |
|  | Lifetime risk^1,2,3)^ | Lifetime risk | | Recommended management | |
|  |  | Women^3)^ | Men^3)^ | Women^3)^ | Men^3)^ |
| Paraganglioma | Japan: 1,500 patients/year  (USA: 2 patients/million) | Potential for increased risk | | ・Annual Blood test (Plasma-free metanephrines)  ・CT or MRI every 2 years  ・I-MIBG scintigraphy every 3 years | |
| Adrenal Pheochromocytoma | 0.01-0.02 ％ | Potential for increased risk | |  |  |

※It is important to note that not all carriers who inherit genetic changes in *MAX* are symptomatic.

※The probabilities shown in Table 1 include benign, borderline malignant, and malignant tumors.

※The likelihood of being symptomatic if carrying a genetic change and recommended management is based on the Japanese guideline^3)^. Please contact specialists and Genetic Services if management is provided at the hospital.

※Table1 is based on information as of 2021. Recommendations may be revised as research progresses.

【References】

- 国立がん研究センター希少がんセンター: パラガングリオーマ <https://www.ncc.go.jp/jp/rcc/about/paraganguriouma/index.html> [ref. 1]
- National Cancer Institute Center for Cancer Research <https://www.cancer.gov/pediatric-adult-rare-tumor/rare-tumors/rare-endocrine-tumor/paraganglioma> [ref. 2]
- 日本内分泌学会. 褐色細胞腫・パラガングリオーマ診療ガイドライン2018. (2018年7月) [ref. 3]
- GeneReviews Japan: 遺伝性パラガングリオーマ・褐色細胞腫症候群. ver.2020.7.15
- ClinGen Actionability Reports: MAX, SDHA, SDHAF2, SDHB, SDHC, SDHD, TMEM127 Adult. ver.1.1.3 (2022.1.3)

supervising editor： Research Group for the Research Project on Ethical, Legal, and Social Issues Supported by the Health, Labour and Welfare Sciences Research Grants “Extraction of ethical and social issues and improvement of social environment toward the realization of a society where people can benefit from genome medicine without anxiety,” Actionability Working Group-Japan

Edited by MONSTAR-SCREEN-2　Medical Genetic Office

***MEN1***

**Genetic Change and Hereditary Cancer Syndrome**

- - If genetic changes at birth are responsible for the hereditary cancer syndrome, the patient is considered to be predisposed to cancer.
  - In general, 5-10% of cancers are caused by genetic changes at birth.
  - Cancer susceptibility may be shared by relatives such as parents, children, siblings.
  - Understanding genetic risks allows better health management.
  - A blood test confirms whether the changes observed in genomic testing (tumor profiling) are hereditary.

**What Is *MEN1* Related To?**

- Genetic changes in the *MEN1* gene causes Multiple Endocrine Neoplasia Type 1 (see Table 1).

**The Benefit of Confirmatory Testing**

- It is important to know the cancer risk for prevention and early detection of cancer.


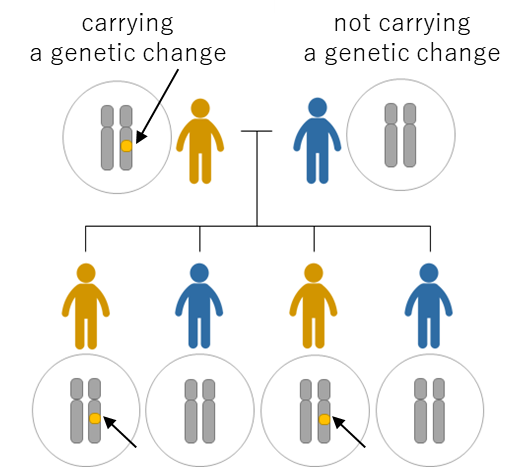
 **Risk of Family Members**

- There are two copies of *MEN1*. One and another copy is inherited from the mother, and the other from the father, respectively.
- Each child, brother, sister, and parent have a 50% chance of inheriting genetic changes in *MEN1*. Genetic testing can be performed to examine if similar changes are exhibited.
- Aunts, uncles, and cousins may share genetic changes and risks.
- Sharing your genetic information with your relatives may help them manage their health.

※ There is an approximately 10%^1)^ probability that individuals diagnosed with Multiple Endocrine Neoplasia Type 1 did not inherit it. This indicates that the genetic change appears in a family for the first time without the exact genetic change in the parents. Please consult your genetic specialist for information about the impact of such a case on your family.

**What Is Genetic Counseling?**

Genetic Counseling provides information on how genetic conditions may affect you and/or your family　and how to manage it. Please feel free to contact your genetic specialist if you have any relevant questions. ※Genetic testing(s) are not always covered by public health insurance in Japan. Genetic testing and follow-up care for healthy relatives who are unaffected by cancer are not covered by insurance. Please ask Genetic Services for details.

Table 1. Lifetime Risk and Recommended Management

|  | Japanese | Carrier of *MEN1* pathogenic / likely pathogenic variant | | | |
| --- | --- | --- | --- | --- | --- |
|  | Lifetime risk^2,3,4,5)^ | Lifetime risk | | Recommended management | |
|  |  | Women^6)^ | Men^6)^ | Women^6,7)^ | Men^6,7)^ |
| Primary Hyperparathyroidism | 1 in 4,000-5,000 people | 95% | | ・Blood test (serum calcium、serum intact PTH), urine test | |
| Gastroenteropancreatic Neuroendocrine Neoplasm | 3-5 in 100,000 people | 60 ％ | | ・Annual blood test (fasting glucose, insulin, gastrin, glucagon)  ・Abdominal CT or MRI, and upper endoscopy every 2-3 years | |
| Pituitary Tumors | 2-3 in 100,000 people | 50 ％ | | ・Blood test (prolactin、growth hormone、IGF-１) every 2-3 years  ・Head MRI or CT every 3-5 years | |
| Adrenocortical Tumor | 1-2 in 1000,000 people | 20 % | | ・MRI or CT (Can usually be evaluated at the same time as the pancreas examination) every 2-3 years | |

※ It is important to note that not all carriers who inherit genetic changes in *MEN1* are symptomatic.

※ The probabilities shown in Table 1 include benign, borderline malignant, and malignant tumors. In addition to the symptoms listed in Table 1, other skin or thymus-related characteristics may be detected. Please contact specialists and Genetic Services for further details.

※The likelihood of being symptomatic if carrying a genetic change and recommended management is based on the Japanese guideline^6)^. Please contact specialists and Genetic Services if management is provided at the hospital.

※Table1 is based on information as of 2021. Recommendations may be revised as research progresses.

【References】

- GeneReviews Japan: 多発性内分泌腫瘍症1型. ver.2018.10.1 [ref. 1]
- Clinical features of multiple endocrine neoplasia type 1(MEN1) phenocopy without germline MEN1 gene mutations: analysis of 20 Japanese sporadic cases with MEN1. Clin Endocrinol (Oxf), 2000; 52(4): 509. [ref. 2]
- 日本神経内分泌腫瘍研究会. 膵・消化管神経内分泌腫瘍診療ガイドライン第２版作成委員会. 膵・消化管神経内分泌腫瘍(NEN)診療ガイドライン2019年第2版. (2019年9月) [ref. 3]
- 全国がん登録罹患数・率 報告 平成30年 <https://www.mhlw.go.jp/content/10900000/000794199.pdf> [ref. 4]
- がんの統計2021 <https://ganjoho.jp/public/qa_links/report/statistics/pdf/cancer_statistics_2021.pdf> [ref. 5]
- 多発性内分泌腫瘍症診療ガイドブック編集委員会. 多発性内分泌腫瘍症 診療ガイドブック. (2013年4月) [ref. 6]
- MEN1遺伝子の発症前診断に用いる説明文書 <http://men-net.org/medical/child.html> [ref. 7]
- ClinGen Actionability Reports: MEN1 Adult. ver.2.2.3 (2021.10.4)

supervising editor： Research Group for the Research Project on Ethical, Legal, and Social Issues Supported by the Health, Labour and Welfare Sciences Research Grants “Extraction of ethical and social issues and improvement of social environment toward the realization of a society where people can benefit from genome medicine without anxiety,” Actionability Working Group-Japan

Edited by MONSTAR-SCREEN-2　Medical Genetic Office

***MET***

**Genetic Change and Hereditary Cancer Syndrome**

- - If genetic changes at birth are responsible for the hereditary cancer syndrome, the patient is considered to be predisposed to cancer.
  - In general, 5-10% of cancers are caused by genetic changes at birth.
  - Cancer susceptibility may be shared by relatives such as parents, children, siblings.
  - Understanding genetic risks allows better health management.
  - A blood test confirms whether the changes observed in genomic testing (tumor profiling) are hereditary.

**What Is *MET* Related To?**

- Genetic changes in the *MET* gene causes Hereditary Papillary Renal Cancer (see Table 1).

**The Benefit of Confirmatory Testing**

- It is important to know the cancer risk for prevention and early detection of cancer.


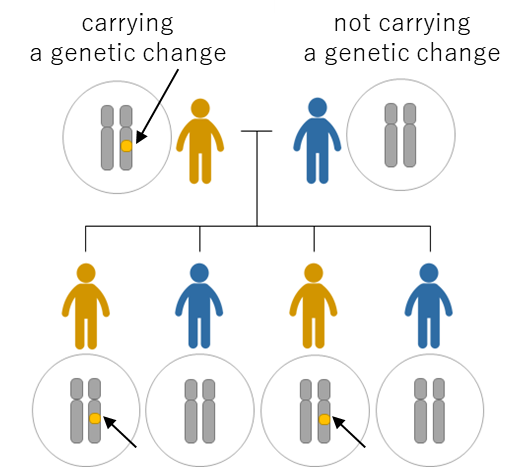
 **Risk of Family Members**

- There are two copies of *MET*. One and another copy is inherited from the mother, and the other from the father, respectively.
- Each child, brother, sister, and parent have a 50% chance of inheriting genetic changes in *MET*. Genetic testing can be performed to examine if similar changes are exhibited.
- Aunts, uncles, and cousins may share genetic changes and risks.
- Sharing your genetic information with your relatives may help them manage their health.

※ It is possible that the *MET* gene change is not inherited from either parent but is a *de* *novo* change. Please consult your genetic specialist for information about the impact of such a case on your family.

**What Is Genetic Counseling?**

Genetic Counseling provides information on how genetic conditions may affect you and/or your family　and how to manage it. Please feel free to contact your genetic specialist if you have any relevant questions. ※Genetic testing(s) are not always covered by public health insurance in Japan. Genetic testing and follow-up care for healthy relatives who are unaffected by cancer are not covered by insurance. Please ask Genetic Services for details.

Table 1. Lifetime Risk and Recommended Management

|  | Japanese | Carrier of *MET* pathogenic / likely pathogenic variant | | | |
| --- | --- | --- | --- | --- | --- |
|  | Lifetime risk^1)^ | Risk up to 80y | | Recommended management | |
|  |  | Women^2)^ | Men^2)^ | Women^3)^ | Men^3)^ |
| Papillary Renal Cancer | (Renal cancer (except renal pelvis cancer))  1.2% (Men)  0.4% (Women) | ≦90 % | | ・Starting at age 30y: abdominal MRI (recommended) or CT every 1-2 years* | |

* The recommended management in Table 1 is not specifically presented in Japan. Please contact the genetic specialist at your institution for further details.

※It is important to note that not all carriers who inherit genetic changes in *MET* develop cancer.

※The likelihood of being symptomatic if carrying a genetic change and recommended management is based on the National Comprehensive Cancer Network guideline^3)^.

※Table1 is based on information as of 2021. Recommendations may be revised as research progresses.

【References】

- 厚生労働省健康局がん・疾病対策課. 平成30年全国がん登録 罹患数・率報告 2018 [ref. 1]
- ClinGen Actionability Reports: MET Adult. ver.1.2.1 (2021.9.15) [ref. 2]
- NCCN Guidelines®: Kidney Cancer. ver.4.2022 (2021.12.21) [ref. 3]

supervising editor： Research Group for the Research Project on Ethical, Legal, and Social Issues Supported by the Health, Labour and Welfare Sciences Research Grants “Extraction of ethical and social issues and improvement of social environment toward the realization of a society where people can benefit from genome medicine without anxiety,” Actionability Working Group-Japan

Edited by MONSTAR-SCREEN-2　Medical Genetic Office

***MLH1***

**Genetic Change and Hereditary Cancer Syndrome**

- - If genetic changes at birth are responsible for the hereditary cancer syndrome, the patient is considered to be predisposed to cancer.
  - In general, 5-10% of cancers are caused by genetic changes at birth.
  - Cancer susceptibility may be shared by relatives such as parents, children, siblings.
  - Understanding genetic risks allows better health management.
  - A blood test confirms whether the changes observed in genomic testing (tumor profiling) are hereditary.

**What Is *MLH1* Related To?**

- Genetic changes in the *MLH1* gene causes Lynch Syndrome (see Table 1).

**The Benefit of Confirmatory Testing**

- It is important to know the cancer risk for prevention and early detection of cancer.


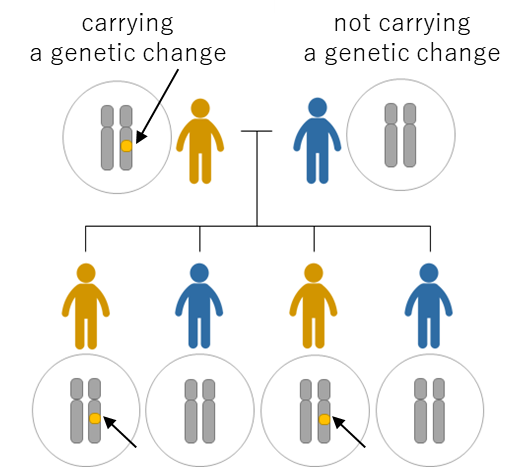
 **Risk of Family Members**

- There are two copies of *MLH1*. One and another copy is inherited from the mother, and the other from the father, respectively.
- Each child, brother, sister, and parent have a 50% chance of inheriting genetic changes in *MLH1*. Genetic testing can be performed to examine if similar changes are exhibited.
- Aunts, uncles, and cousins may share genetic changes and risks.
- Sharing your genetic information with your relatives may help them manage their health.

※ It is possible that the *MLH1* gene change is not inherited from either parent but is a *de* *novo* change. Please consult your genetic specialist for information about the impact of such a case on your family.

**What Is Genetic Counseling?**

Genetic Counseling provides information on how genetic conditions may affect you and/or your family　and how to manage it. Please feel free to contact your genetic specialist if you have any relevant questions. ※Genetic testing(s) are not always covered by public health insurance in Japan. Genetic testing and follow-up care for healthy relatives who are unaffected by cancer are not covered by insurance. Please ask Genetic Services for details.

Table 1. Lifetime Risk and Recommended Management

|  | Japanese | Carrier of *MLH1* pathogenic / likely pathogenic variant | | | |
| --- | --- | --- | --- | --- | --- |
|  | Lifetime risk^1)^ | Risk up to 80y | | Recommended management | |
|  |  | Women^２)^ | Men^２)^ | Women^３)^ | Men^３)^ |
| Colorectal Cancer | 10.2 ％ (Men)  8.0 ％ (Women) | 46-61 % | | ・Starting at ages 20-25y: colonoscopy every 1-2 years | |
| Endometrial Cancer | 2.0% (Women) | 34-54 % | ― | ・Starting at ages 30-35y: annual transvaginal ultrasound, endometrial biopsy, (CA-125 screening) | |
| Ovarian Cancer | 1.6% (Women) | 4-20 % | ― |  |  |
| Gastric Cancer | 10.3 ％ (Men)  4.7 ％ (Women) | 5-7 %* | | (Consider those with a family history of gastric or duodenal cancer.)  ・Starting at ages 30-35y: Eradication of H.pylori  ・Upper endoscopy every 1-3 years | |
| Ureter and Renal Pelvis Cancer | 2.2 % (Men)  1.1 % (Women) | 0.2-5 % | | (Consider in those with a family history of urothelial cancer)  ・Starting at ages 30-35y: annual urine test (or urine cytology) | |
| Bladder Cancer | 2.1 % (Men)  0.7 % (Women) | 2-7 % | |  |  |

* The risk listed in Table 1 is based on the data from other countries. The risk of gastric cancer is higher in East Asian populations than that in Western populations^4)^.　It has been reported the risk of gastric cancer in Japanese patients diagnosed with Lynch syndrome is 14.5-24% by the age of 60-70 years^5,6)^.

※It is important to note that not all carriers who inherit genetic changes in *MLH1* develop cancer.

※The likelihood of being symptomatic if carrying a genetic change and recommended management is based on the Japanese guideline^3)^ and the National Comprehensive Cancer Network guideline^2)^. However, these recommendations are not always implemented in Japan. Please contact specialists and Genetic Services if management is provided at the hospital.

※Table1 is based on information as of 2021. Recommendations may be revised as research progresses.

【References】

- 国立がん研究センターがん情報サービス「累積がん罹患リスク (2018年データ)」 <https://ganjoho.jp/reg_stat/statistics/stat/summary.html> [ref. 1]
- NCCN Guidelines® Genetic/Familial High-Risk Assessment: Colorectal. ver.1.2021 (2021.5.11) [ref. 2]
- 大腸癌研究会. 遺伝性大腸癌診療ガイドライン 2020年版. (2020年4月) [ref. 3]
- Managing gastric cancer risk in lynch syndrome: controversies and recommendations. Fam Cancer, 2021 (online ahead of print). [ref. 4]
- Tumor development in Japanese patients with Lynch syndrome. PLoS One, 2018; 13(4): e0195572. [ref. 5]
- Comparison of clinical features between suspected familial colorectal cancer type X and Lynch syndrome in Japanese patients with colorectal cancer: a cross-sectional study conducted by the Japanese Society for Cancer of the Colon and Rectum. Jpn J Clin Oncol, 2015; 45(2): 153. [ref. 6]
- GeneReviews Japan: リンチ症候群. ver.2021.4.21
- ClinGen Actionability Reports: MLH1, MSH2, MSH6, PMS2, EPCAM Adult. ver.1.1.2 (2021.9.15)

supervising editor： Research Group for the Research Project on Ethical, Legal, and Social Issues Supported by the Health, Labour and Welfare Sciences Research Grants “Extraction of ethical and social issues and improvement of social environment toward the realization of a society where people can benefit from genome medicine without anxiety,” Actionability Working Group-Japan

Edited by MONSTAR-SCREEN-2　Medical Genetic Office

***MSH2***

**Genetic Change and Hereditary Cancer Syndrome**

- - If genetic changes at birth are responsible for the hereditary cancer syndrome, the patient is considered to be predisposed to cancer.
  - In general, 5-10% of cancers are caused by genetic changes at birth.
  - Cancer susceptibility may be shared by relatives such as parents, children, siblings.
  - Understanding genetic risks allows better health management.
  - A blood test confirms whether the changes observed in genomic testing (tumor profiling) are hereditary.

**What Is *MSH2* Related To?**

- Genetic changes in the *MSH2* gene causes Lynch Syndrome (see Table 1).

**The Benefit of Confirmatory Testing**

- It is important to know the cancer risk for prevention and early detection of cancer.


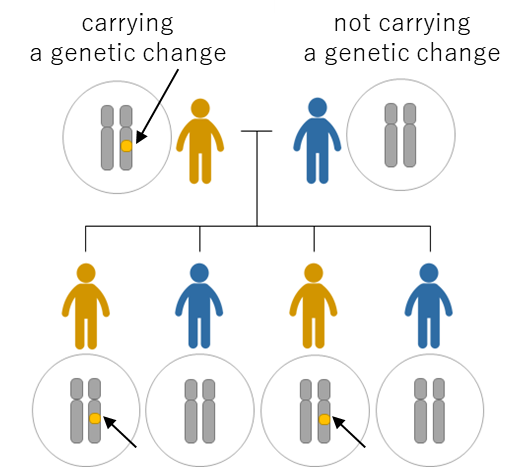
 **Risk of Family Members**

- There are two copies of *MSH2*. One and another copy is inherited from the mother, and the other from the father, respectively.
- Each child, brother, sister, and parent have a 50% chance of inheriting genetic changes in *MSH2*. Genetic testing can be performed to examine if similar changes are exhibited.
- Aunts, uncles, and cousins may share genetic changes and risks.
- Sharing your genetic information with your relatives may help them manage their health.

※ It is possible that the *MSH2* gene change is not inherited from either parent but is a *de* *novo* change. Please consult your genetic specialist for information about the impact of such a case on your family.

**What Is Genetic Counseling?**

Genetic Counseling provides information on how genetic conditions may affect you and/or your family　and how to manage it. Please feel free to contact your genetic specialist if you have any relevant questions. ※Genetic testing(s) are not always covered by public health insurance in Japan. Genetic testing and follow-up care for healthy relatives who are unaffected by cancer are not covered by insurance. Please ask Genetic Services for details.

Table 1. Lifetime Risk and Recommended Management

|  | Japanese | Carrier of *MSH2* pathogenic / likely pathogenic variant | | | |
| --- | --- | --- | --- | --- | --- |
|  | Lifetime risk^1)^ | Risk up to 80y | | Recommended management | |
|  |  | Women^２)^ | Men^２)^ | Women^３)^ | Men^３)^ |
| Colorectal Cancer | 10.2 ％ (Men)  8.0 ％ (Women) | 33-52 % | | ・Starting at ages 20-25y: colonoscopy every 1-2 years | |
| Endometrial Cancer | 2.0% (Women) | 21-57 % | ― | ・Starting at ages 30-35y: annual transvaginal ultrasound, endometrial biopsy, (CA-125 screening) | |
| Ovarian Cancer | 1.6% (Women) | 8-38 % | ― |  |  |
| Gastric Cancer | 10.3 ％ (Men)  4.7 ％ (Women) | 0.2-9 %* | | (Consider in those with a family history of gastric or duodenal cancer.)  ・Starting at ages 30-35y: Eradication of H.pylori  ・Upper endoscopy every 1-3 years | |
| Ureter and Renal Pelvis Cancer | 2.2 % (Men)  1.1 % (Women) | 2.2-28 %  (Risk is higher in men) | | (Consider in those with a family history of urothelial cancer)  ・Starting at ages 30-35y: annual urine test (or urine cytology) | |
| Bladder Cancer | 2.1 % (Men)  0.7 % (Women) | 4.4-12.8 % | |  |  |

* The risk listed in Table 1 is based on the data from other countries. The risk of gastric cancer is higher in East Asian populations than that in Western populations ^4)^.　It has been reported the risk of gastric cancer in Japanese patients diagnosed with Lynch syndrome is 14.5-24% by the age of 60-70 years^5,6)^.

※It is important to note that not all carriers who inherit genetic changes in *MSH2* develop cancer.

※The likelihood of being symptomatic if carrying a genetic change and recommended management is based on the Japanese guideline^3)^ and the National Comprehensive Cancer Network guideline^2)^. However, these recommendations are not always implemented in Japan. Please contact specialists and Genetic Services if management is provided at the hospital.

※Table1 is based on information as of 2021. Recommendations may be revised as research progresses.

【References】

- 国立がん研究センターがん情報サービス「累積がん罹患リスク (2018年データ)」 <https://ganjoho.jp/reg_stat/statistics/stat/summary.html> [ref. 1]
- NCCN Guidelines® Genetic/Familial High-Risk Assessment: Colorectal. ver.1.2021 (2021.5.11) [ref. 2]
- 大腸癌研究会. 遺伝性大腸癌診療ガイドライン 2020年版. (2020年4月) [ref. 3]
- Managing gastric cancer risk in lynch syndrome: controversies and recommendations. Fam Cancer, 2021 (online ahead of print). [ref. 4]
- Tumor development in Japanese patients with Lynch syndrome. PLoS One, 2018; 13(4): e0195572. [ref. 5]
- Comparison of clinical features between suspected familial colorectal cancer type X and Lynch syndrome in Japanese patients with colorectal cancer: a cross-sectional study conducted by the Japanese Society for Cancer of the Colon and Rectum. Jpn J Clin Oncol, 2015; 45(2): 153. [ref. 6]
- GeneReviews Japan: リンチ症候群. ver.2021.4.21
- ClinGen Actionability Reports: MLH1, MSH2, MSH6, PMS2, EPCAM Adult. ver.1.1.2 (2021.9.15)

supervising editor： Research Group for the Research Project on Ethical, Legal, and Social Issues Supported by the Health, Labour and Welfare Sciences Research Grants “Extraction of ethical and social issues and improvement of social environment toward the realization of a society where people can benefit from genome medicine without anxiety,” Actionability Working Group-Japan

Edited by MONSTAR-SCREEN-2　Medical Genetic Office

***MSH6***

**Genetic Change and Hereditary Cancer Syndrome**

- - If genetic changes at birth are responsible for the hereditary cancer syndrome, the patient is considered to be predisposed to cancer.
  - In general, 5-10% of cancers are caused by genetic changes at birth.
  - Cancer susceptibility may be shared by relatives such as parents, children, siblings.
  - Understanding genetic risks allows better health management.
  - A blood test confirms whether the changes observed in genomic testing (tumor profiling) are hereditary.

**What Is *MSH6* Related To?**

- Genetic changes in the *MSH6* gene causes Lynch Syndrome (see Table 1).

**The Benefit of Confirmatory Testing**

- It is important to know the cancer risk for prevention and early detection of cancer.


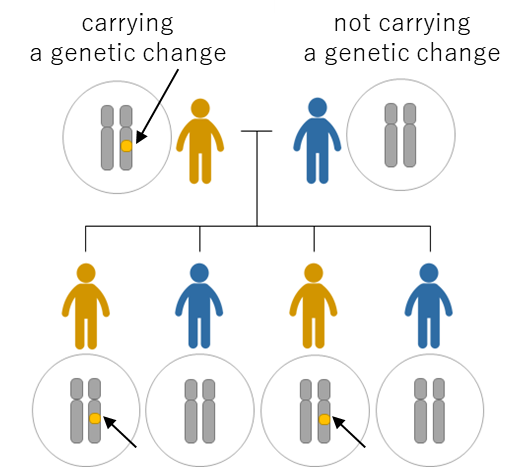
 **Risk of Family Members**

- There are two copies of *MSH6*. One and another copy is inherited from the mother, and the other from the father, respectively.
- Each child, brother, sister, and parent have a 50% chance of inheriting genetic changes in *MSH6*. Genetic testing can be performed to examine if similar changes are exhibited.
- Aunts, uncles, and cousins may share genetic changes and risks.
- Sharing your genetic information with your relatives may help them manage their health.

※ It is possible that the *MSH6* gene change is not inherited from either parent but is a *de* *novo* change. Please consult your genetic specialist for information about the impact of such a case on your family.

**What Is Genetic Counseling?**

Genetic Counseling provides information on how genetic conditions may affect you and/or your family　and how to manage it. Please feel free to contact your genetic specialist if you have any relevant questions. ※Genetic testing(s) are not always covered by public health insurance in Japan. Genetic testing and follow-up care for healthy relatives who are unaffected by cancer are not covered by insurance. Please ask Genetic Services for details.

Table 1. Lifetime Risk and Recommended Management

|  | Japanese | Carrier of *MSH6* pathogenic / likely pathogenic variant | | | |
| --- | --- | --- | --- | --- | --- |
|  | Lifetime risk^1)^ | Risk up to 80y | | Recommended management | |
|  |  | Women^２)^ | Men^２)^ | Women^３)^ | Men^３)^ |
| Colorectal Cancer | 10.2 ％ (Men)  8.0 ％ (Women) | 10-44 % | | ・Starting at ages 20-25y: colonoscopy every 1-2 years | |
| Endometrial Cancer | 2.0% (Women) | 16-49 % | ― | ・Starting at ages 30-35y: annual transvaginal ultrasound, endometrial biopsy, (CA-125 screening) | |
| Ovarian Cancer | 1.6% (Women) | ≦1-13 % | ― |  |  |
| Gastric Cancer | 10.3 ％ (Men)  4.7 ％ (Women) | ≦1-7.9 %* | | (Consider those with a family history of gastric or duodenal cancer.)  ・Starting after 30-35y: Eradication of H.pylori  ・Upper endoscopy every 1-3 years | |
| Ureter and Renal Pelvis Cancer | 2.2 % (Men)  1.1 % (Women) | 0.7-5.5 % | | (Consider in those with a family history of urothelial cancer)  ・Starting at ages　30-35y: annual urine test (or urine cytology) | |
| Bladder Cancer | 2.1 % (Men)  0.7 % (Women) | 1.0-8.2 % | |  |  |

* The risk listed in Table 1 is based on the data from other countries. The risk of gastric cancer is higher in East Asian populations than that in Western populations ^4)^.　It has been reported the risk of gastric cancer in Japanese patients diagnosed with Lynch syndrome is 14.5-24% by the age of 60-70 years^5,6)^.

※It is important to note that not all carriers who inherit genetic changes in *MSH6* develop cancer.

※The likelihood of being symptomatic if carrying a genetic change and recommended management is based on the Japanese guideline^3)^ and the National Comprehensive Cancer Network guideline^2)^. However, these recommendations are not always implemented in Japan. Please contact specialists and Genetic Services if management is provided at the hospital.

※Table1 is based on information as of 2021. Recommendations may be revised as research progresses.

【References】

- 国立がん研究センターがん情報サービス「累積がん罹患リスク (2018年データ)」 <https://ganjoho.jp/reg_stat/statistics/stat/summary.html> [ref. 1]
- NCCN Guidelines® Genetic/Familial High-Risk Assessment: Colorectal. ver.1.2021 (2021.5.11) [ref. 2]
- 大腸癌研究会. 遺伝性大腸癌診療ガイドライン 2020年版. (2020年4月) [ref. 3]
- Managing gastric cancer risk in lynch syndrome: controversies and recommendations. Fam Cancer, 2021 (online ahead of print). [ref. 4]
- Tumor development in Japanese patients with Lynch syndrome. PLoS One, 2018; 13(4): e0195572. [ref. 5]
- Comparison of clinical features between suspected familial colorectal cancer type X and Lynch syndrome in Japanese patients with colorectal cancer: a cross-sectional study conducted by the Japanese Society for Cancer of the Colon and Rectum. Jpn J Clin Oncol, 2015; 45(2): 153. [ref. 6]
- GeneReviews Japan: リンチ症候群. ver.2021.4.21
- ClinGen Actionability Reports: MLH1, MSH2, MSH6, PMS2, EPCAM Adult. ver.1.1.2 (2021.9.15)

supervising editor： Research Group for the Research Project on Ethical, Legal, and Social Issues Supported by the Health, Labour and Welfare Sciences Research Grants “Extraction of ethical and social issues and improvement of social environment toward the realization of a society where people can benefit from genome medicine without anxiety,” Actionability Working Group-Japan

Edited by MONSTAR-SCREEN-2　Medical Genetic Office

***MUTYH***

**※This fact sheet will be used when genetic changes in *MUTYH* are detected in both alleles.**

**Genetic Change and Hereditary Cancer Syndrome**

- - If genetic changes at birth are responsible for the hereditary cancer syndrome, the patient is considered to be predisposed to cancer.
  - In general, 5-10% of cancers are caused by genetic changes at birth.
  - Cancer susceptibility may be shared by relatives such as parents, children, siblings.
  - Understanding genetic risks allows better health management.
  - A blood test confirms whether the changes observed in genomic testing (tumor profiling) are hereditary.

**What Is *MUTYH* Related To?**

- People carry two *MUTYH* genes and being born with pathogenic genetic changes in both *MUTYH* genes increases the risk of cancer (Table 1).
- Two (biallelic) genetic changes in the *MUTYH* gene cause *MUTYH*-associated polyposis. (see Table 1)

**The Benefit of Confirmatory Testing**

- It is important to know the cancer risk for prevention and early detection of cancer.


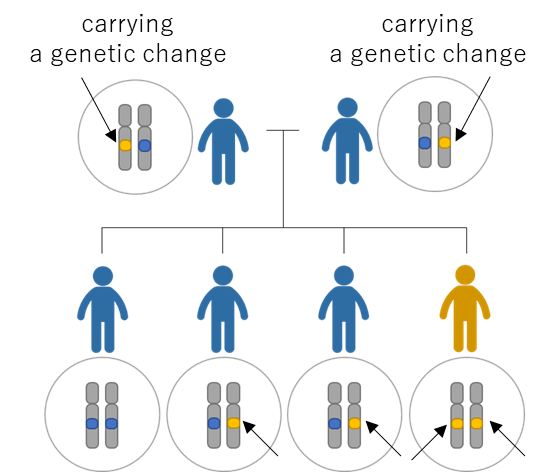
 **Risk of Family Members**

- You have two copies of the *MUTYH* gene. One copy is inherited from the mother and the other inherited from the father, respectively.
- If you were born with changes in both *MUTYH* copies, you may have inherited the changes in each gene from your parents.
- Children, brothers, siblings, uncles, and cousins may also share genetic changes. However, if the genetic changes were only on one side, they would not affect the patient’s health condition.
- Sharing your genetic information with your relatives may help them manage their health.

※ It is possible that the *MUTYH* gene change at birth is not inherited from either parent, but is a *de* *novo* change. Please consult your genetic specialist for information about the impact of this case on your family.

**What Is Genetic Counseling?**

Genetic Counseling provides information on how genetic conditions may affect you and/or your family　and how to manage it. Please feel free to contact your genetic specialist if you have any relevant questions. ※Genetic testing(s) are not always covered by public health insurance in Japan. Genetic testing and follow-up care for healthy relatives who are unaffected by cancer are not covered by insurance. Please ask Genetic Services for details.

Table 1. Lifetime Risk and Recommended Management

|  | Japanese | Biallelic carrier of *MUTYH* pathogenic / likely pathogenic variant | | | |
| --- | --- | --- | --- | --- | --- |
|  | Lifetime risk^1,2)^ | Risk up to 60y | | Recommended management | |
|  |  | Women^3)^ | Men^3)^ | Women^4,5)^ | Men^4,5)^ |
| Colorectal Polyposis  (Risk of cancer) | (Colon cancer)  10.2 ％ (Men)  8.0 ％ (Women) | (Colon cancer)  43-100 % | | ・Colonoscopy: starting at age 25-30y, repeat every 1-3 years depending on polyp burden | |
| Duodenal Cancer | (Small intestinal cancer)  0.2% (Men)  0.1% (Women) | Potential  increased risk | | ・Starting at age 30-35y：Annual upper endoscopy | |

※It is important to note that not all carriers who inherit genetic changes in the *MUTYH* gene develop cancer.

※The likelihood of being symptomatic if carrying a genetic change and recommended management is based on the Japanese guideline^3)^ and the International (U.S. and Europe) guidelines^4,5)^. The recommendations may not always be implemented in Japan. Please contact specialists and Genetic Services if management is provided at the hospital.

※Table1 is based on information as of 2021. Recommendations may be revised as research progresses.

【References】

- 国立がん研究センターがん情報サービス「累積がん罹患リスク (2018年データ)」 <https://ganjoho.jp/reg_stat/statistics/stat/summary.html> [ref. 1]
- 厚生労働省健康局がん・疾病対策課. 平成30年全国がん登録 罹患数・率報告 2018 [ref. 2]
- 大腸癌研究会. 遺伝性大腸癌診療ガイドライン 2020年版. (2020年4月) [ref. 3]
- NCCN Guidelines® Genetic/Familial High-Risk Assessment: Colorectal. ver.1.2021 (2021.5.11) [ref. 4]
- Revised guidelines for the clinical management of Lynch syndrome (HNPCC): recommendations by a group of European experts. Gut, 2013; 62(6): 812. [ref. 5]
- GeneReviews®: *MUTYH* Polyposis. ver.2021.5.27.
- ClinGen Actionability Reports: MUTYH Adult. ver.2.2.2 (2022.1.3)

supervising editor： Research Group for the Research Project on Ethical, Legal, and Social Issues Supported by the Health, Labour and Welfare Sciences Research Grants “Extraction of ethical and social issues and improvement of social environment toward the realization of a society where people can benefit from genome medicine without anxiety,” Actionability Working Group-Japan

Edited by MONSTAR-SCREEN-2　Medical Genetic Office

***NBN***

**If germline genetic changes are found in this gene, the pathogenicity should be discussed on a case-by-case basis. Please consult the Research Office first.**

**Genetic Change and Hereditary Cancer Syndrome**

- - If genetic changes at birth are responsible for the hereditary cancer syndrome, the patient is considered to be predisposed to cancer.
  - In general, 5-10% of cancers are caused by genetic changes at birth.
  - Cancer susceptibility may be shared by relatives such as parents, children, siblings.
  - Understanding genetic risks allows better health management.
  - A blood test confirms whether the changes observed in genomic testing (tumor profiling) are hereditary.

**What Is *NBN* Related To?**

- Certain genetic changes in *NBN* can increase cancer.^1,2)^ (see Table 1)

**The Benefit of Confirmatory Testing**

- It is important to know the cancer risk for prevention and early detection of cancer.


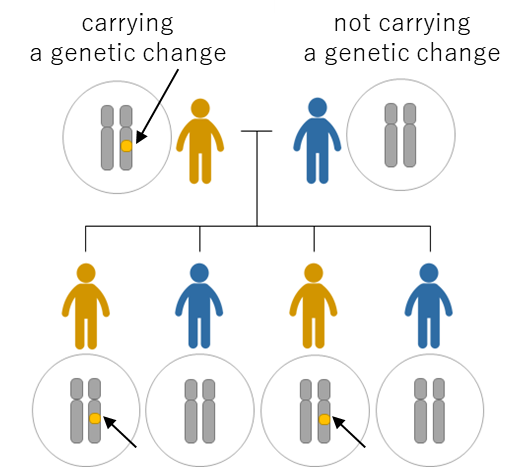
 **Risk of Family Members**

- There are two copies of *NBN*. One and another copy is inherited from the mother, and the other from the father, respectively.
- Each child, brother, sister, and parent have a 50% chance of inheriting genetic changes in *NBN*. Genetic testing can be performed to examine if similar changes are exhibited.
- Aunts, uncles, and cousins may share genetic changes and risks.
- Sharing your genetic information with your relatives may help them manage their health.

※ It is possible that the *NBN* gene change is not inherited from either parent but is a *de* *novo* change. Please consult your genetic specialist for information about the impact of such a case on your family.

**What Is Genetic Counseling?**

Genetic Counseling provides information on how genetic conditions may affect you and/or your family　and how to manage it. Please feel free to contact your genetic specialist if you have any relevant questions. ※Genetic testing(s) are not always covered by public health insurance in Japan. Genetic testing and follow-up care for healthy relatives who are unaffected by cancer are not covered by insurance. Please ask Genetic Services for details.

Table 1. Lifetime Risk and Recommended Management

|  | Japanese | Carrier of *NBN* pathogenic / likely pathogenic variant | | | |
| --- | --- | --- | --- | --- | --- |
|  | Lifetime risk^1)^ | Lifetime risk | | Recommended management | |
|  |  | Women^2,3)^ | Men | Women^2)^ | Men |
| Ovarian Cancer | 1.6 % (Women) | Potential for increased risk | ― | ※Risks are considered to vary depending on family and medical history. Currently, there is no established management. | ― |
| Breast Cancer | 10.9 ％ (Women) | Potential for increased risk | ― |  | ― |

※It is important to note that not all carriers who inherit genetic changes in *NBN* develop cancer.

※Table 1 shows the data for certain types of *NBN* genetic changes. Management should be considered based on the type of genetic change.

※Recommended management for screening is currently recommended to actively perform general cancer screening. Please contact the genetic specialist at your institution for further details.

※Table1 is based on information as of 2021. Recommendations may be revised as research progresses.

【References】

- 国立がん研究センターがん情報サービス「累積がん罹患リスク (2018年データ)」 https://ganjoho.jp/reg_stat/statistics/stat/summary.html [ref. 1]
- NCCN Guidelines® Genetic/Familial High-Risk Assessment: Breast, Ovarian, and Pancreatic. ver.1.2022 (2021.8.11) [ref. 2]
- GeneReviews®: Nijmegen Breakage Syndrome. ver.2017.2.2 [ref. 3]

supervising editor： Research Group for the Research Project on Ethical, Legal, and Social Issues Supported by the Health, Labour and Welfare Sciences Research Grants “Extraction of ethical and social issues and improvement of social environment toward the realization of a society where people can benefit from genome medicine without anxiety,” Actionability Working Group-Japan

Edited by MONSTAR-SCREEN-2　Medical Genetic Office

***NF1***

**Genetic Change and Hereditary Cancer Syndrome**

- - If genetic changes at birth are responsible for the hereditary cancer syndrome, the patient is considered to be predisposed to cancer.
  - In general, 5-10% of cancers are caused by genetic changes at birth.
  - Cancer susceptibility may be shared by relatives such as parents, children, siblings.
  - Understanding genetic risks allows better health management.
  - A blood test confirms whether the changes observed in genomic testing (tumor profiling) are hereditary.

**What Is *NF1* Related To?**

- Genetic changes in the *NF1* gene causes Neurofibromatosis 1 (see Table 1).

**The Benefit of Confirmatory Testing**

- It is important to know the cancer risk for prevention and early detection of cancer.


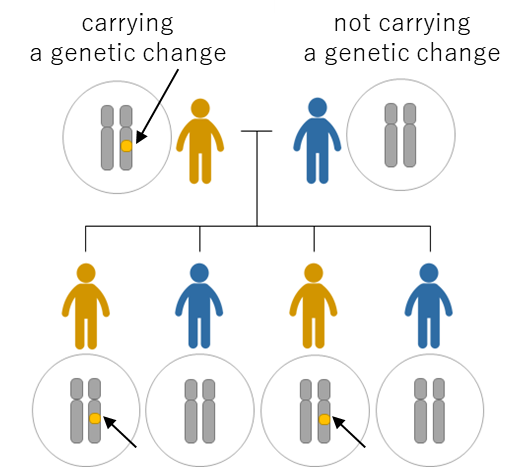
 **Risk of Family Members**

- There are two copies of *NF1*. One and another copy is inherited from the mother, and the other from the father, respectively.
- Each child, brother, sister, and parent have a 50% chance of inheriting genetic changes in *NF1*. Genetic testing can be performed to examine if similar changes are exhibited.
- Aunts, uncles, and cousins may share genetic changes and risks.
- Sharing your genetic information with your relatives may help them manage their health.

※ There is an approximately 50%^1)^ probability that individuals diagnosed with Neurofibromatosis 1 did not inherit it. This indicates that the genetic change appears in a family for the first time without the exact genetic change in the parents. Please consult your genetic specialist for information about the impact of such a case on your family.

**What Is Genetic Counseling?**

Genetic Counseling provides information on how genetic conditions may affect you and/or your family　and how to manage it. Please feel free to contact your genetic specialist if you have any relevant questions. ※Genetic testing(s) are not always covered by public health insurance in Japan. Genetic testing and follow-up care for healthy relatives who are unaffected by cancer are not covered by insurance. Please ask Genetic Services for details.

Table 1. Lifetime Risk and Recommended Management

|  | Japanese | Carrier of *NF1* pathogenic / likely pathogenic variant | | | |
| --- | --- | --- | --- | --- | --- |
|  | Lifetime risk^2)^ | Lifetime risk | | Recommended management | |
|  |  | Women^3,4,5)^ | Men^3,4,5)^ | Women^3,4)^ | Men^3,4)^ |
| Breast Cancer | 10.9 ％ | 15-40 ％ | ― | ・Starting at age 30y: annual mammogram  ・Starting at age 30-50y: annual breast MRI screening with contrast | ― |
| Gastrointestinal Stromal Tumor (GIST) | No data available  ※Rare cancer | 5-25 % | | ・If symptoms such as blood in stools or abdominal pain occur, consult a health care provider. | |
| Malignant Peripheral Nerve Sheath Tumor | No data available  ※Rare cancer | 2 % | | ・If there is rapid tumor growth or sudden onset of neurologic symptoms, consult a health care provider. | |

- In addition to the symptoms listed in Table 1, other skin, nerve, or eye-related characteristics may be detected. Please contact specialists and Genetic Services for details.

※It is important to note that not all carriers who inherit genetic changes in *NF1* develop cancer.

※The likelihood of being symptomatic if carrying a genetic change and recommended management is based on the Japanese guideline^3)^ and the National Comprehensive Cancer Network guideline^4)^. However, these recommendations are not always implemented in Japan. Please contact specialists and Genetic Services if management is provided at the hospital.

※Table1 is based on information as of 2021. Recommendations may be revised as research progresses.

【References】

- GeneReviews Japan: 神経線維腫症1型. ver.2021.1.12 [ref. 1]
- 国立がん研究センターがん情報サービス「累積がん罹患リスク (2018年データ)」https://ganjoho.jp/reg_stat/statistics/stat/summary.html [ref. 2]
- 日本皮膚科学会. 神経線維腫症1型 (レックリングハウゼン病) 診療ガイドライン 2018. 日皮会誌, 2018; 128(1): 17. [ref. 3]
- NCCN Guidelines® Genetic/Familial High-Risk Assessment: Breast, Ovarian, and Pancreatic. ver.1.2022 (2021.8.11) [ref. 4]
- Therapeutic consequences from molecular biology for gastrointestinal stromal tumor patients affected by neurofibromatosis type 1. Clin Cancer Res, 2008; 14(14): 4550. [ref. 5]
- ClinGen Actionability Reports: NF1 Adult. ver.1.2.1 (2020.8.19)

supervising editor： Research Group for the Research Project on Ethical, Legal, and Social Issues Supported by the Health, Labour and Welfare Sciences Research Grants “Extraction of ethical and social issues and improvement of social environment toward the realization of a society where people can benefit from genome medicine without anxiety,” Actionability Working Group-Japan

Edited by MONSTAR-SCREEN-2　Medical Genetic Office

***NF2***

**Genetic Change and Hereditary Cancer syndrome**

- - If genetic changes at birth are responsible for the hereditary cancer syndrome, the patient is considered to be predisposed to cancer.
  - In general, 5-10% of cancers are caused by genetic changes at birth.
  - Cancer susceptibility may be shared by relatives such as parents, children, siblings.
  - Understanding genetic risks allows better health management.
  - A blood test confirms whether the changes observed in genomic testing (tumor profiling) are hereditary.

**What Is *NF2* Related To?**

- Genetic changes in the *NF2* gene causes Neurofibromatosis 2 (see Table 1).

**The Benefit of Confirmatory Testing**

- It is important to know the cancer risk for prevention and early detection of cancer.


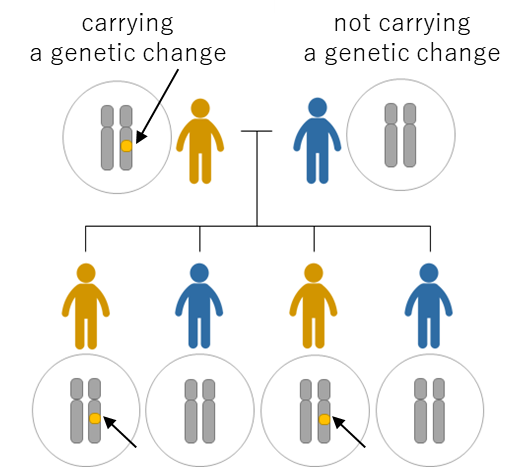
 **Risk of Family Members**

- There are two copies of *NF2*. One and another copy is inherited from the mother, and the other from the father, respectively.
- Each child, brother, sister, and parent have a 50% chance of inheriting genetic changes in *NF2*. Genetic testing can be performed to examine if similar changes are exhibited.
- Aunts, uncles, and cousins may share genetic changes and risks.
- Sharing your genetic information with your relatives may help them manage their health.

※ There is an approximately 50%^1,2)^ probability that individuals diagnosed with Neurofibromatosis 2 did not inherit it. This indicates that the genetic change appears in a family for the first time without the exact genetic change in the parents. Please consult your genetic specialist for information about the impact of such a case on your family.

※ It is possible that the *NF2* gene change is not inherited from either parent but is a *de* *novo* change. Please consult your genetic specialist for information about the impact of such a case on your family.

**What Is Genetic Counseling?**

Genetic Counseling provides information on how genetic conditions may affect you and/or your family　and how to manage it. Please feel free to contact your genetic specialist if you have any relevant questions. ※Genetic testing(s) are not always covered by public health insurance in Japan. Genetic testing and follow-up care for healthy relatives who are unaffected by cancer are not covered by insurance. Please ask Genetic Services for details.

Table 1. Lifetime Risk and Recommended Management

|  | Japanese | Carrier of *NF2* pathogenic / likely pathogenic variant | | | |
| --- | --- | --- | --- | --- | --- |
|  | Lifetime risk | Lifetime risk | | Recommended management | |
|  |  | Women^2,3)^ | Men^2,3)^ | Women^1,2)^ | Men^1,2)^ |
| Vestibular Schwannomas  (Acoustic neuroma) | No data available | Almost 100 % | | ・Starting between ages 10-12y to 40y: annual MRI and hearing test | |
| Spinal Cord Tumor | No data available | Approximately 33 % | |  |  |
| Meningioma | No data available | 50-80 % | |  |  |

- In addition to the symptoms listed in Table 1, other brain, nerve, eye, or skin-related characteristics may be detected. Please contact specialists and Genetic Services for details.

※It is important to note that not all carriers who inherit genetic changes in *NF2* develop cancer.

※The likelihood of being symptomatic if carrying a genetic change and recommended management is based on the international data. However, these recommendations are not always implemented in Japan. Please contact specialists and Genetic Services if management is provided at the hospital.

【References】

- 厚生労働科学研究費補助金（難治性疾患政策研究事業）「神経皮膚症候群に関する診療科横断的な診療体制の確立」研究班. 神経線維腫症２型 (NF2) 治療指針 (2016年10月)　[ref. 1]
- GeneReviews®: Neurofibromatosis 2. ver.2018.3.15 [ref. 2]
- ClinGen Actionability Reports: NF2 Adult. ver.2.0.2 (2021.5.5) [ref. 3]

supervising editor： Research Group for the Research Project on Ethical, Legal, and Social Issues Supported by the Health, Labour and Welfare Sciences Research Grants “Extraction of ethical and social issues and improvement of social environment toward the realization of a society where people can benefit from genome medicine without anxiety,” Actionability Working Group-Japan

Edited by MONSTAR-SCREEN-2　Medical Genetic Office

***PALB2***

**Genetic Change and Hereditary Cancer Syndrome**

- - If genetic changes at birth are responsible for the hereditary cancer syndrome, the patient is considered to be predisposed to cancer.
  - In general, 5-10% of cancers are caused by genetic changes at birth.
  - Cancer susceptibility may be shared by relatives such as parents, children, siblings.
  - Understanding genetic risks allows better health management.
  - A blood test confirms whether the changes observed in genomic testing (tumor profiling) are hereditary.

**What Is *PALB2* Related To?**

- *PALB2* has been shown to be associated with an increased risk of cancer (see Table 1).

**The Benefit of Confirmatory Testing**

- It is important to know the cancer risk for prevention and early detection of cancer.


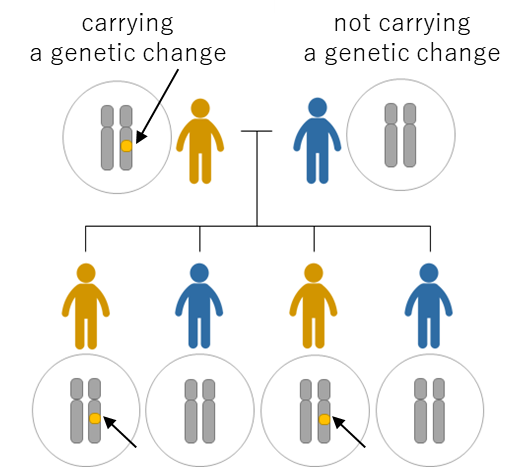
 **Risk of Family Members**

- There are two copies of *PALB2*. One and another copy is inherited from the mother, and the other from the father, respectively.
- Each child, brother, sister, and parent have a 50% chance of inheriting genetic changes in *PALB2*. Genetic testing can be performed to examine if similar changes are exhibited.
- Aunts, uncles, and cousins may share genetic changes and risks.
- Sharing your genetic information with your relatives may help them manage their health.

※ It is possible that the *PALB2* gene change is not inherited from either parent but is a *de* *novo* change. Please consult your genetic specialist for information about the impact of such a case on your family.

**What Is Genetic Counseling?**

Genetic Counseling provides information on how genetic conditions may affect you and/or your family　and how to manage it. Please feel free to contact your genetic specialist if you have any relevant questions. ※Genetic testing(s) are not always covered by public health insurance in Japan. Genetic testing and follow-up care for healthy relatives who are unaffected by cancer are not covered by insurance. Please ask Genetic Services for details.

Table 1. Lifetime Risk and Recommended Management

|  | Japanese | Carrier of *PALB2* pathogenic / likely pathogenic variant | | | |
| --- | --- | --- | --- | --- | --- |
|  | Lifetime risk^1)^ | Lifetime risk | | Recommended management | |
|  |  | Women^2)^ | Men^2)^ | Women^2)^ | Men^2)^ |
| Breast Cancer | 10.9 ％  (Women) | 41-60 ％ | ― | ・Starting at age 30y: annual mammogram and breast MRI screening with contrast* | ― |
| Ovarian Cancer | 1.6 % (Women) | 3-5 % | ― | ※Currently, there is no established management. Risks are considered to vary depending on family and medical history. | ― |
| Pancreatic Cancer | 2.6 %  (Men and Women) | 5-10 % | | ※Currently, there is no established management. | |

* The recommended management in Table 1 is not specifically presented in Japan. Please contact the genetic specialist at your institution for further details.

※It is important to note that not all carriers who inherit genetic changes in *PALB2* develop cancer.

※The likelihood of being symptomatic if carrying a genetic change and recommended management is based on the National Comprehensive Cancer Network guideline^2)^.

※Table1 is based on information as of 2021. Recommendations may be revised as research progresses.

【References】

- 国立がん研究センターがん情報サービス「累積がん罹患リスク (2018年データ)」 https://ganjoho.jp/reg_stat/statistics/stat/summary.html [ref. 1]
- NCCN Guidelines® Genetic/Familial High-Risk Assessment: Breast, Ovarian, and Pancreatic. ver.1.2022 (2021.8.11) [ref. 2]
- ClinGen Actionability Reports: PALB2 Adult. ver.2.2.1 (2021.9.15)

supervising editor： Research Group for the Research Project on Ethical, Legal, and Social Issues Supported by the Health, Labour and Welfare Sciences Research Grants “Extraction of ethical and social issues and improvement of social environment toward the realization of a society where people can benefit from genome medicine without anxiety,” Actionability Working Group-Japan

Edited by MONSTAR-SCREEN-2　Medical Genetic Office

***PMS2***

**Genetic Change and Hereditary Cancer Syndrome**

- - If genetic changes at birth are responsible for the hereditary cancer syndrome, the patient is considered to be predisposed to cancer.
  - In general, 5-10% of cancers are caused by genetic changes at birth.
  - Cancer susceptibility may be shared by relatives such as parents, children, siblings.
  - Understanding genetic risks allows better health management.
  - A blood test confirms whether the changes observed in genomic testing (tumor profiling) are hereditary.

**What Is *PMS2* Related To?**

- Genetic changes in the *PMS2* gene causes Lynch Syndrome (see Table 1).

**The Benefit of Confirmatory Testing**

- It is important to know the cancer risk for prevention and early detection of cancer.


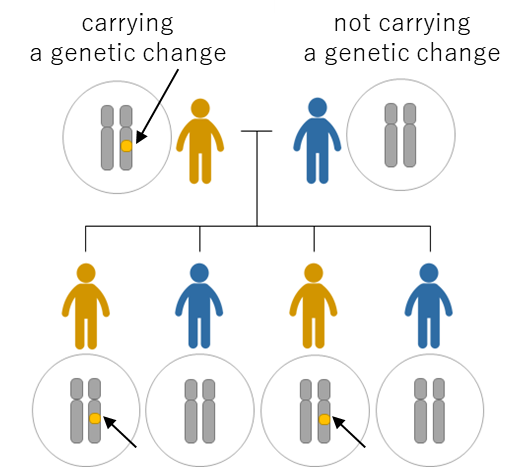
 **Risk of Family Members**

- There are two copies of *PMS2*. One and another copy is inherited from the mother, and the other from the father, respectively.
- Each child, brother, sister, and parent have a 50% chance of inheriting genetic changes in *PMS2*. Genetic testing can be performed to examine if similar changes are exhibited.
- Aunts, uncles, and cousins may share genetic changes and risks.
- Sharing your genetic information with your relatives may help them manage their health.

※ It is possible that the *PMS2* gene change is not inherited from either parent but is a *de* *novo* change. Please consult your genetic specialist for information about the impact of such a case on your family.

**What Is Genetic Counseling?**

Genetic Counseling provides information on how genetic conditions may affect you and/or your family　and how to manage it. Please feel free to contact your genetic specialist if you have any relevant questions. ※Genetic testing(s) are not always covered by public health insurance in Japan. Genetic testing and follow-up care for healthy relatives who are unaffected by cancer are not covered by insurance. Please ask Genetic Services for details.

Table 1. Lifetime Risk and Recommended Management

|  | Japanese | Carrier of *PMS2* pathogenic / likely pathogenic variant | | | |
| --- | --- | --- | --- | --- | --- |
|  | Lifetime risk^1)^ | Risk up to 80y | | Recommended management | |
|  |  | Women^２)^ | Men^２)^ | Women^３)^ | Men^３)^ |
| Colorectal Cancer | 10.2 ％ (Men)  8.0 ％ (Women) | 8.7-20 % | | ・Starting at ages 20-25y: colonoscopy every 1-2 years | |
| Endometrial Cancer | 2.0% (Women) | 13-26 % | ― | ・Starting at ages 30-35y: annual transvaginal ultrasound, endometrial biopsy, (CA-125 screening) | |
| Ovarian Cancer | 1.6% (Women) | 1.3-3 % | ― |  |  |
| Gastric Cancer | 10.3 ％ (Men)  4.7 ％ (Women) | No data available* | | (Consider those with a family history of gastric or duodenal cancer.)  ・Starting at ages 30-35y: Eradication of H.pylori  ・Upper endoscopy every 1-3 years | |
| Ureter and Renal Pelvis Cancer | 2.2 % (Men)  1.1 % (Women) | ≦1-3.7 % | | (Consider in those with a family history of urothelial cancer)  ・Starting at ages 30-35y: annual urine test (or urine cytology) | |
| Bladder Cancer | 2.1 % (Men)  0.7 % (Women) | ≦1-2.4 % | |  |  |

* The risk listed in Table 1 is based on the data from other countries. The risk of gastric cancer is higher in East Asian populations than that in Western populations ^4)^.　It has been reported the risk of gastric cancer in Japanese patients diagnosed with Lynch syndrome is 14.5-24% by the age of 60-70 years^5,6)^.

※It is important to note that not all carriers who inherit genetic changes in *PMS2* develop cancer.

※The likelihood of being symptomatic if carrying a genetic change and recommended management is based on the Japanese guideline^3)^ and the National Comprehensive Cancer Network guideline^2)^. However, these recommendations are not always implemented in Japan. Please contact specialists and Genetic Services if management is provided at the hospital.

※Table1 is based on information as of 2021. Recommendations may be revised as research progresses.

【References】

- 国立がん研究センターがん情報サービス「累積がん罹患リスク (2018年データ)」 <https://ganjoho.jp/reg_stat/statistics/stat/summary.html> [ref. 1]
- NCCN Guidelines® Genetic/Familial High-Risk Assessment: Colorectal. ver.1.2021 (2021.5.11) [ref. 2]
- 大腸癌研究会. 遺伝性大腸癌診療ガイドライン 2020年版. (2020年4月) [ref. 3]
- Managing gastric cancer risk in lynch syndrome: controversies and recommendations. Fam Cancer, 2021 (online ahead of print). [ref. 4]
- Tumor development in Japanese patients with Lynch syndrome. PLoS One, 2018; 13(4): e0195572. [ref. 5]
- Comparison of clinical features between suspected familial colorectal cancer type X and Lynch syndrome in Japanese patients with colorectal cancer: a cross-sectional study conducted by the Japanese Society for Cancer of the Colon and Rectum. Jpn J Clin Oncol, 2015; 45(2): 153. [ref. 6]
- GeneReviews Japan: リンチ症候群. ver.2021.4.21
- ClinGen Actionability Reports: MLH1, MSH2, MSH6, PMS2, EPCAM Adult. ver.1.1.2 (2021.9.15)

supervising editor： Research Group for the Research Project on Ethical, Legal, and Social Issues Supported by the Health, Labour and Welfare Sciences Research Grants “Extraction of ethical and social issues and improvement of social environment toward the realization of a society where people can benefit from genome medicine without anxiety,” Actionability Working Group-Japan

Edited by MONSTAR-SCREEN-2　Medical Genetic Office

***POLD1***

**Genetic Change and Hereditary Cancer Syndrome**

- - If genetic changes at birth are responsible for the hereditary cancer syndrome, the patient is considered to be predisposed to cancer.
  - In general, 5-10% of cancers are caused by genetic changes at birth.
  - Cancer susceptibility may be shared by relatives such as parents, children, siblings.
  - Understanding genetic risks allows better health management.
  - A blood test confirms whether the changes observed in genomic testing (tumor profiling) are hereditary.

**What Is *POLD1* Related To?**

- Genetic changes in the *POLD1* gene causes Polymerase proofreading-associated polyposis (see Table 1).

**The Benefit of Confirmatory Testing**

- It is important to know the cancer risk for prevention and early detection of cancer.


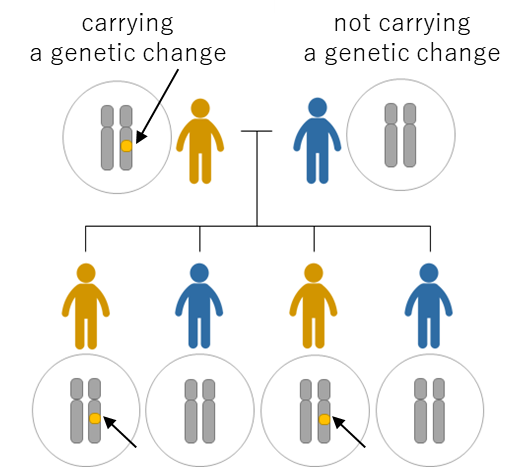
 **Risk of Family Members**

- There are two copies of *POLD1*. One and another copy is inherited from the mother, and the other from the father, respectively.
- Each child, brother, sister, and parent have a 50% chance of inheriting genetic changes in *POLD1*. Genetic testing can be performed to examine if similar changes are exhibited.
- Aunts, uncles, and cousins may share genetic changes and risks.
- Sharing your genetic information with your relatives may help them manage their health.

※ It is possible that the *POLD1* gene change is not inherited from either parent but is a *de* *novo* change. Please consult your genetic specialist for information about the impact of such a case on your family.

**What Is Genetic Counseling?**

Genetic Counseling provides information on how genetic conditions may affect you and/or your family　and how to manage it. Please feel free to contact your genetic specialist if you have any relevant questions. ※Genetic testing(s) are not always covered by public health insurance in Japan. Genetic testing and follow-up care for healthy relatives who are unaffected by cancer are not covered by insurance. Please ask Genetic Services for details.

Table 1. Lifetime Risk and Recommended Management

|  | Japanese | Carrier of *POLD1* pathogenic / likely pathogenic variant | | | | |
| --- | --- | --- | --- | --- | --- | --- |
|  | Lifetime risk^1,2)^ | Lifetime risk | | Recommended management | | |
|  |  | Women^3,4)^ | Men^3,4)^ | Women^5)^ | | Men^5)^ |
| Colorectal Cancer | 10.2 ％ (Men)  8.0 ％ (Women) | 52 % | 63 % | ・Start ages at 25-30y: Colonoscopy every 1-3 years* | | |
| Endometrial Cancer | 2.0% (Women) | Potential for increased risk | ― | ・Currently, there is no established management. | ― | |
| Breast Cancer | 10.9% (Women) | Potential for increased risk | ― |  |  |  |
| Brain Tumor | 0.3 ％ (Men)  0.2 ％ (Women) | Potential for increased risk | | ・Currently, there is no established management. | | |

* The recommended management in Table 1 is not specifically presented in Japan. Please contact the genetic specialist at your institution for further details.

※It is important to note that not all carriers who inherit genetic changes in *POLD1* develop cancer.

※Recommended management for screening is currently recommended to actively perform general cancer screening.

※Table1 is based on information as of 2021. Recommendations may be revised as research progresses.

【References】

- 国立がん研究センターがん情報サービス「累積がん罹患リスク (2018年データ)」[https://ganjoho.jp/reg_stat/statistics/stat/summary.htmll](https://ganjoho.jp/reg_stat/statistics/stat/summary.html) [ref. 1]
- 厚生労働省健康局がん・疾病対策課. 平成30年全国がん登録 罹患数・率報告 2018. [ref. 2]
- Risk of colorectal cancer for carriers of a germ-line mutation in POLE or POLD1. Genet Med. 2018; 20(8): 890. [ref. 3]
- 大腸癌研究会. 遺伝性大腸癌診療ガイドライン 2020年版. (2020年4月) [ref. 4]
- NCCN Guidelines® Genetic/Familial High-Risk Assessment: Colorectal. ver.1.2021 (2021.5.11) [ref. 5]
- ClinGen Actionability Reports: POLE, POLD1 Adult. ver.1.1.3 (2021.11.15)

supervising editor： Research Group for the Research Project on Ethical, Legal, and Social Issues Supported by the Health, Labour and Welfare Sciences Research Grants “Extraction of ethical and social issues and improvement of social environment toward the realization of a society where people can benefit from genome medicine without anxiety,” Actionability Working Group-Japan

Edited by MONSTAR-SCREEN-2　Medical Genetic Office

***POLE***

**Genetic Change and Hereditary Cancer Syndrome**

- - If genetic changes at birth are responsible for the hereditary cancer syndrome, the patient is considered to be predisposed to cancer.
  - In general, 5-10% of cancers are caused by genetic changes at birth.
  - Cancer susceptibility may be shared by relatives such as parents, children, siblings.
  - Understanding genetic risks allows better health management.
  - A blood test confirms whether the changes observed in genomic testing (tumor profiling) are hereditary.

**What Is *POLE* Related To?**

- Genetic changes in the *POLE* gene causes Polymerase proofreading-associated polyposis (see Table 1).

**The Benefit of Confirmatory Testing**

- It is important to know the cancer risk for prevention and early detection of cancer.


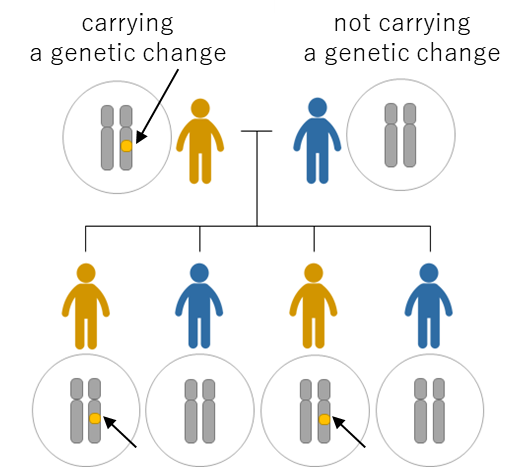
 **Risk of Family Members**

- There are two copies of *POLE*. One and another copy is inherited from the mother, and the other from the father, respectively.
- Each child, brother, sister, and parent have a 50% chance of inheriting genetic changes in *POLE*. Genetic testing can be performed to examine if similar changes are exhibited.
- Aunts, uncles, and cousins may share genetic changes and risks.
- Sharing your genetic information with your relatives may help them manage their health.

※ It is possible that the *POLE* gene change is not inherited from either parent but is a *de* *novo* change. Please consult your genetic specialist for information about the impact of such a case on your family.

**What Is Genetic Counseling?**

Genetic Counseling provides information on how genetic conditions may affect you and/or your family　and how to manage it. Please feel free to contact your genetic specialist if you have any relevant questions. ※Genetic testing(s) are not always covered by public health insurance in Japan. Genetic testing and follow-up care for healthy relatives who are unaffected by cancer are not covered by insurance. Please ask Genetic Services for details.

Table 1. Lifetime Risk and Recommended Management

|  | Japanese | Carrier of *POLE* pathogenic / likely pathogenic variant | | | |
| --- | --- | --- | --- | --- | --- |
|  | Lifetime risk^1,2)^ | Lifetime risk | | Recommended management | |
|  |  | Women^3,4)^ | Men^3,4)^ | Women^5)^ | Men^5)^ |
| Colorectal Cancer | 10.2 ％ (Men)  8.0 ％ (Women) | 32 % | 40 % | ・Start ages at 25-30y:Colonoscopy every 1-3 years* | |
| Duodenal Cancer | (Small intestinal cancer)  0.2% (Men)  0.1% (Women) | Potential for increased risk | | ・Currently, there is no established management. | |
| Brain Tumor | 0.3 ％ (Men)  0.2 ％ (Women) | Potential for increased risk | | ・Currently, there is no established management. | |

* The recommended management in Table 1 is not specifically presented in Japan. Please contact the genetic specialist at your institution for further details.

※It is important to note that not all carriers who inherit genetic changes in *POLE* develop cancer.

※Recommended management for screening is currently recommended to actively perform general cancer screening.

※Table1 is based on information as of 2021. Recommendations may be revised as research progresses.

【References】

- 国立がん研究センターがん情報サービス「累積がん罹患リスク (2018年データ)」[https://ganjoho.jp/reg_stat/statistics/stat/summary.htmll](https://ganjoho.jp/reg_stat/statistics/stat/summary.html) [ref. 1]
- 厚生労働省健康局がん・疾病対策課. 平成30年全国がん登録 罹患数・率報告 2018. [ref. 2]
- Risk of colorectal cancer for carriers of a germ-line mutation in POLE or POLD1. Genet Med. 2018; 20(8): 890. [ref. 3]
- 大腸癌研究会. 遺伝性大腸癌診療ガイドライン 2020年版. (2020年4月) [ref. 4]
- NCCN Guidelines® Genetic/Familial High-Risk Assessment: Colorectal. ver.1.2021 (2021.5.11) [ref. 5]
- ClinGen Actionability Reports: POLE, POLD1 Adult. ver.1.1.3 (2021.11.15)

supervising editor： Research Group for the Research Project on Ethical, Legal, and Social Issues Supported by the Health, Labour and Welfare Sciences Research Grants “Extraction of ethical and social issues and improvement of social environment toward the realization of a society where people can benefit from genome medicine without anxiety,” Actionability Working Group-Japan

Edited by MONSTAR-SCREEN-2　Medical Genetic Office

***POT1***

**Genetic Change and Hereditary Cancer Syndrome**

- - If genetic changes at birth are responsible for the hereditary cancer syndrome, the patient is considered to be predisposed to cancer.
  - In general, 5-10% of cancers are caused by genetic changes at birth.
  - Cancer susceptibility may be shared by relatives such as parents, children, siblings.
  - Understanding genetic risks allows better health management.
  - A blood test confirms whether the changes observed in genomic testing (tumor profiling) are hereditary.

**What Is *POT1* Related To?**

- *POT1* has been shown to be associated with an increased risk of cancer^1)^ (see Table 1).

**The Benefit of Confirmatory Testing**

- It is important to know the cancer risk for prevention and early detection of cancer.


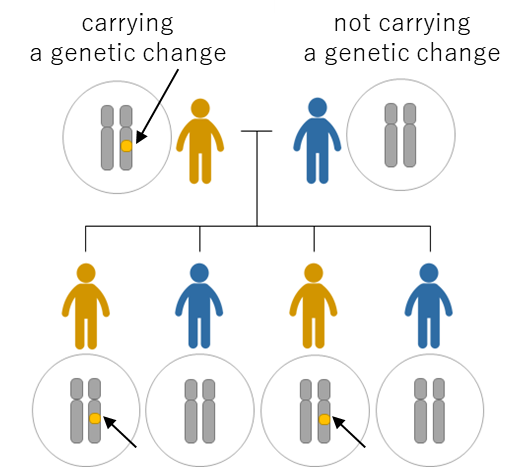
 **Risk of Family Members**

- There are two copies of *POT1*. One and another copy is inherited from the mother, and the other from the father, respectively.
- Each child, brother, sister, and parent have a 50% chance of inheriting genetic changes in *POT1*. Genetic testing can be performed to examine if similar changes are exhibited.
- Aunts, uncles, and cousins may share genetic changes and risks.
- Sharing your genetic information with your relatives may help them manage their health.

※ It is possible that the *POT1* gene change is not inherited from either parent but is a *de* *novo* change. Please consult your genetic specialist for information about the impact of such a case on your family.

**What Is Genetic Counseling?**

Genetic Counseling provides information on how genetic conditions may affect you and/or your family　and how to manage it. Please feel free to contact your genetic specialist if you have any relevant questions. ※Genetic testing(s) are not always covered by public health insurance in Japan. Genetic testing and follow-up care for healthy relatives who are unaffected by cancer are not covered by insurance. Please ask Genetic Services for details.

Table 1. Lifetime Risk and Recommended Management

|  | Japanese | Carrier of *POT1* pathogenic / likely pathogenic variant | | | |
| --- | --- | --- | --- | --- | --- |
|  | Lifetime risk^2)^ | Lifetime risk | | Recommended management | |
|  |  | Women^3)^ | Men^3)^ | Women^4)^ | Men^4)^ |
| Melanoma* | 0.1 % (Men)  0.1 % (Women) | Potential increased risk | | ・Comprehensive skin examination by a dermatologist, including total body skin, scalp, oral mucosa, and genitals every 6 months (if the nevus is stable: every 12 month) **  ・Monthly nevus self-examination** | |

* The annual prevalence of malignant melanoma is reported to be 24.3 in 100,000 people for Caucasians and 1.7 in 100,000 people for Asians^6)^, and the risk is considered to vary according to race, region, and other genetic factors.

** The recommended management in Table 1 is not specifically presented in Japan. Please contact the genetic specialist at your institution for further details.

※In addition to the above symptoms, hematological and neurological symptoms may be seen. Please contact specialists and Genetic Services for details.

※It is important to note that not all carriers who inherit genetic changes in *POT1* develop cancer.

※The likelihood of being symptomatic if carrying a genetic change and recommended management is based on international data^3,4)^.

※Table1 is based on information as of 2021. Recommendations may be revised as research progresses.

【References】

- *CDKN2A* genetic testing in melanoma-prone families in Sweden in the years 2015-2020: implications for novel national recommendations. Acta Oncologica, 2021; 60(7): 888. [ref. 1]
- 厚生労働省健康局がん・疾病対策課. 平成30年全国がん登録 罹患数・率報告 2018 [ref. 2]
- GeneReviews® *POT1* Tumor Predisposition. ver.2020.10.29 [ref. 3]
- ClinGen Actionability Reports: CDKN2A Adult. ver.1.2.1 (2020.8.19) [ref. 4]
- 日本皮膚科学会. 皮膚悪性腫瘍ガイドライン第 3 版 メラノーマ診療ガイドライン 2019. 日皮会誌, 2019; 129(9):1759. [ref. 5]

supervising editor： Research Group for the Research Project on Ethical, Legal, and Social Issues Supported by the Health, Labour and Welfare Sciences Research Grants “Extraction of ethical and social issues and improvement of social environment toward the realization of a society where people can benefit from genome medicine without anxiety,” Actionability Working Group-Japan

Edited by MONSTAR-SCREEN-2　Medical Genetic Office

***PTEN***

**Genetic Change and Hereditary Cancer Syndrome**

- - If genetic changes at birth are responsible for the hereditary cancer syndrome, the patient is considered to be predisposed to cancer.
  - In general, 5-10% of cancers are caused by genetic changes at birth.
  - Cancer susceptibility may be shared by relatives such as parents, children, siblings.
  - Understanding genetic risks allows better health management.
  - A blood test confirms whether the changes observed in genomic testing (tumor profiling) are hereditary.

**What Is *PTEN* Related To?**

- Genetic changes in the *PTEN* gene causes *PTEN* Hamartoma Tumor Syndrome (Cowden syndrome) (see Table 1).

**The Benefit of Confirmatory Testing**

- It is important to know the cancer risk for prevention and early detection of cancer.


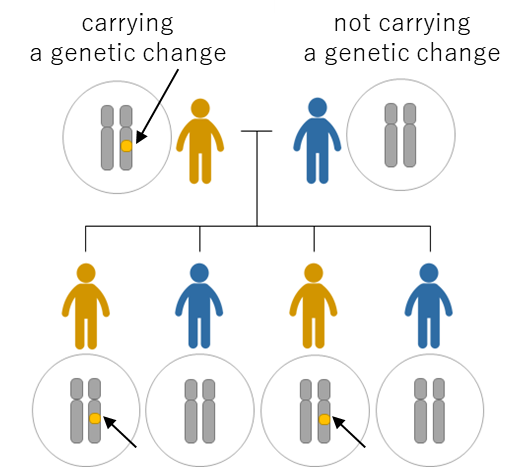
 **Risk of Family Members**

- There are two copies of *PTEN*. One and another copy is inherited from the mother, and the other from the father, respectively.
- Each child, brother, sister, and parent have a 50% chance of inheriting genetic changes in *PTEN*. Genetic testing can be performed to examine if similar changes are exhibited.
- Aunts, uncles, and cousins may share genetic changes and risks.
- Sharing your genetic information with your relatives may help them manage their health.

※ It is possible that the *PTEN* gene change is not inherited from either parent but is a *de* *novo* change. Please consult your genetic specialist for information about the impact of such a case on your family.

**What Is Genetic Counseling?**

Genetic Counseling provides information on how genetic conditions may affect you and/or your family　and how to manage it. Please feel free to contact your genetic specialist if you have any relevant questions. ※Genetic testing(s) are not always covered by public health insurance in Japan. Genetic testing and follow-up care for healthy relatives who are unaffected by cancer are not covered by insurance. Please ask Genetic Services for details.

Table 1. Lifetime Risk and Recommended Management

|  | Japanese | Carrier of *PTEN* pathogenic / likely pathogenic variant | | | |
| --- | --- | --- | --- | --- | --- |
|  | Lifetime risk^1)^ | Lifetime risk | | Recommended management | |
|  |  | Women^2)^ | Men^2)^ | Women^2,3)^ | Men^2,3)^ |
| Hamartoma/  Breast Cancer | Breast cancer  10.9 ％  (Women) | Hamartoma  No evidence of increased risk compared to the general population  Breast cancer  25-85 ％^3)^ | ＊Case report only  No evidence of apparent risk | ・Starting at age 18y: breast awareness  ・Starting at age 25y: clinical breast exam every 6-12 months  ・Starting at age 30y**: annual mammogram and breast MRI screening with contrast  **5–10 years earlier than the youngest diagnosis in the family | |
| Benign Thyroid Tumor/Thyroid Cancer | Thyroid cancer  10.8 ％  (Men and Women) | Benign thyroid tumor  30-68 %  Thyroid cancer  10-35 ％^1)^ | | ・Starting at age 7y or after diagnosis: Annual thyroid ultrasound | |
| Endometrial Cancer | Uterine cancer  2.0 %  (Women) | Endometrial cancer  19-28 % | ― | ・Starting at age 35y: endometrial biopsy every 1-2 years  ・Postmenopausal: consider transvaginal ultrasound  ・Completion of childbearing: Discuss hysterectomy | |
| Colon Polyp/  Colorectal Cancer | Colorectal Cancer  8.1 %  (Women)　10.3 % (Men) | Gastrointestinal polyps  90 %≦  Cancer  9~16 % | | ・Starting at age 35y***：colonoscopy every 5 years  ***5–10 years earlier than the youngest diagnosis in the family, if there is a family member diagnosed before 40y. | |
| Renal Cell Carcinoma | 6 in 100,000 people | 34% | | ・Starting at age 40y: renal ultrasound every 1-2 years | |

- Other conditions, such as skin, nerve, and vascular system, may occur in association with the genetic change in the *PTEN* gene. Please contact specialists and Genetic Services for further details.

※It is important to note that not all carriers who inherit genetic changes in *PTEN* develop symptom.

※The likelihood of being symptomatic if carrying a genetic change and recommended management is based on the Japanese guideline^2)^ and the National Comprehensive Cancer Network guideline^3)^. However, these recommendations are not always implemented in Japan. Please contact specialists and Genetic Services if management is provided at the hospital.

※Table1 is based on information as of 2021. Recommendations may be revised as research progresses.

【References】

- 国立がん研究センターがん情報サービス「累積がん罹患リスク (2018年データ)」<https://ganjoho.jp/reg_stat/statistics/stat/summary.html> [ref. 1]
- 小児・成人のためのCowden症候群/PTEN過誤腫症候群診療ガイドライン 2020年版. 遺伝性腫瘍, 2020; 20(2): 93. [ref. 2]
- NCCN Guidelines® Genetic/Familial High-Risk Assessment: Breast, Ovarian, and Pancreatic. ver.1.2022 (2021.8.11) [ref. 3]
- GeneRevies Japan: *PTEN*過誤腫症候群. ver.2021.3.1.
- ClinGen Actionability Reports: PTEN Adult. ver.2.1.2 (2021.6.21)

supervising editor： Research Group for the Research Project on Ethical, Legal, and Social Issues Supported by the Health, Labour and Welfare Sciences Research Grants “Extraction of ethical and social issues and improvement of social environment toward the realization of a society where people can benefit from genome medicine without anxiety,” Actionability Working Group-Japan

Edited by MONSTAR-SCREEN-2　Medical Genetic Office

***RAD51C***

**Genetic Change and Hereditary Cancer Syndrome**

- - If genetic changes at birth are responsible for the hereditary cancer syndrome, the patient is considered to be predisposed to cancer.
  - In general, 5-10% of cancers are caused by genetic changes at birth.
  - Cancer susceptibility may be shared by relatives such as parents, children, siblings.
  - Understanding genetic risks allows better health management.
  - A blood test confirms whether the changes observed in genomic testing (tumor profiling) are hereditary.

**What Is *RAD51C* Related To?**

- *RAD51C* has been shown to be associated with an increased risk of cancer.^1,2)^ (see Table 1)

**The Benefit of Confirmatory Testing**

- It is important to know the cancer risk for prevention and early detection of cancer.


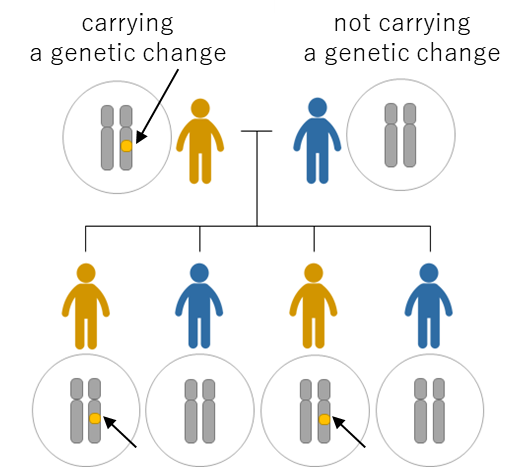
 **Risk of Family Members**

- There are two copies of *RAD51C*. One and another copy is inherited from the mother, and the other from the father, respectively.
- Each child, brother, sister, and parent have a 50% chance of inheriting genetic changes in *RAD51C*. Genetic testing can be performed to examine if similar changes are exhibited.
- Aunts, uncles, and cousins may share genetic changes and risks.
- Sharing your genetic information with your relatives may help them manage their health.

※ It is possible that the *RAD51C* gene change is not inherited from either parent but is a *de* *novo* change. Please consult your genetic specialist for information about the impact of such a case on your family.

**What Is Genetic Counseling?**

Genetic Counseling provides information on how genetic conditions may affect you and/or your family　and how to manage it. Please feel free to contact your genetic specialist if you have any relevant questions. ※Genetic testing(s) are not always covered by public health insurance in Japan. Genetic testing and follow-up care for healthy relatives who are unaffected by cancer are not covered by insurance. Please ask Genetic Services for details.

Table 1. Lifetime Risk and Recommended Management

|  | Japanese | Carrier of *RAD51C* pathogenic / likely pathogenic variant | | | |
| --- | --- | --- | --- | --- | --- |
|  | Lifetime risk^1)^ | Lifetime risk | | Recommended management | |
|  |  | Women^2)^ | Men | Women^2)^ | Men |
| Ovarian Cancer | 1.6 % (Women) | >10 % | ― | ・Starting at ages 45-50y: consider risk-reducing salpingo-oophorectpmy (RRSO)*  ※Risks are considered to vary depending on family and medical history. Currently, there is no established management. | ― |
| Breast Cancer | 10.9 ％ (Women) | 15-40 ％ | ― | ※Risks are considered to vary depending on family and medical history. Currently, there is no established management. | ― |

* The recommended management in Table 1 is not specifically presented in Japan. Please contact the genetic specialist at your institution for further details.

※It is important to note that not all carriers who inherit genetic changes in *RAD51C* develop cancer.

※The likelihood of being symptomatic if carrying a genetic change and recommended management is based on the National Comprehensive Cancer Network guideline^2)^.

※Table1 is based on information as of 2021. Recommendations may be revised as research progresses.

【References】

- 国立がん研究センターがん情報サービス「累積がん罹患リスク (2018年データ)」https://ganjoho.jp/reg_stat/statistics/stat/summary.html [ref. 1]
- NCCN Guidelines® Genetic/Familial High-Risk Assessment: Breast, Ovarian, and Pancreatic. ver.1.2022 (2021.8.11) [ref. 2]
- ClinGen Actionability Reports: BRIP1, RAD51C, RAD51D Adult. ver.1.1.1 (2020.7.13)

supervising editor： Research Group for the Research Project on Ethical, Legal, and Social Issues Supported by the Health, Labour and Welfare Sciences Research Grants “Extraction of ethical and social issues and improvement of social environment toward the realization of a society where people can benefit from genome medicine without anxiety,” Actionability Working Group-Japan

Edited by MONSTAR-SCREEN-2　Medical Genetic Office

***RAD51D***

**Genetic Change and Hereditary Cancer Syndrome**

- - If genetic changes at birth are responsible for the hereditary cancer syndrome, the patient is considered to be predisposed to cancer.
  - In general, 5-10% of cancers are caused by genetic changes at birth.
  - Cancer susceptibility may be shared by relatives such as parents, children, siblings.
  - Understanding genetic risks allows better health management.
  - A blood test confirms whether the changes observed in genomic testing (tumor profiling) are hereditary.

**What Is *RAD51D* Related To?**

- *RAD51D* has been shown to be associated with an increased risk of cancer.^1,2)^ (see Table 1)

**The Benefit of Confirmatory Testing**

- It is important to know the cancer risk for prevention and early detection of cancer.


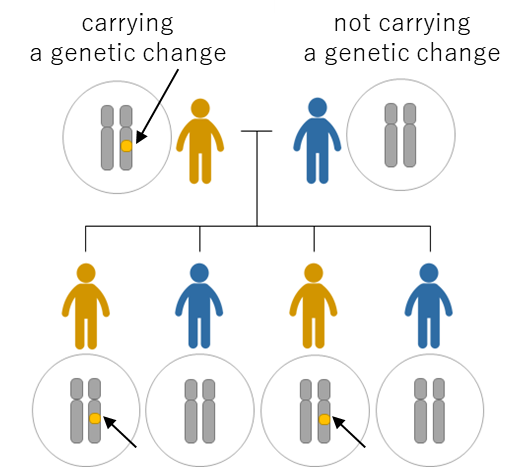
 **Risk of Family Members**

- There are two copies of *RAD51D*. One and another copy is inherited from the mother, and the other from the father, respectively.
- Each child, brother, sister, and parent have a 50% chance of inheriting genetic changes in *RAD51D*. Genetic testing can be performed to examine if similar changes are exhibited.
- Aunts, uncles, and cousins may share genetic changes and risks.
- Sharing your genetic information with your relatives may help them manage their health.

※ It is possible that the *RAD51D* gene change is not inherited from either parent but is a *de* *novo* change. Please consult your genetic specialist for information about the impact of such a case on your family.

**What Is Genetic Counseling?**

Genetic Counseling provides information on how genetic conditions may affect you and/or your family　and how to manage it. Please feel free to contact your genetic specialist if you have any relevant questions. ※Genetic testing(s) are not always covered by public health insurance in Japan. Genetic testing and follow-up care for healthy relatives who are unaffected by cancer are not covered by insurance. Please ask Genetic Services for details.

Table 1. Lifetime Risk and Recommended Management

|  | Japanese | Carrier of *RAD51D* pathogenic / likely pathogenic variant | | | |
| --- | --- | --- | --- | --- | --- |
|  | Lifetime risk^1)^ | Lifetime risk | | Recommended management | |
|  |  | Women^2)^ | Men | Women^2)^ | Men |
| Ovarian Cancer | 1.6 % (Women) | >10 % | ― | ・Starting at age 45-50y: consider risk-reducing salpingo-oophorectpmy (RRSO)*  ※Risks are considered to vary depending on family and medical history. Currently, there is no established management. | ― |
| Breast Cancer | 10.9 ％ (Women) | 15-40 ％ | ― | ※Risks are considered to vary depending on family and medical history. Currently, there is no established management. | ― |

* The recommended management in Table 1 is not specifically presented in Japan. Please contact the genetic specialist at your institution for further details.

※It is important to note that not all carriers who inherit genetic changes in *RAD51D* develop cancer.

※The likelihood of being symptomatic if carrying a genetic change and recommended management is based on the National Comprehensive Cancer Network guideline^2)^.

※Table1 is based on information as of 2021. Recommendations may be revised as research progresses.

【References】

- 国立がん研究センターがん情報サービス「累積がん罹患リスク (2018年データ)」https://ganjoho.jp/reg_stat/statistics/stat/summary.html [ref. 1]
- NCCN Guidelines® Genetic/Familial High-Risk Assessment: Breast, Ovarian, and Pancreatic. ver.1.2022 (2021.8.11) [ref. 2]
- ClinGen Actionability Reports: BRIP1, RAD51C, RAD51D Adult. ver.1.1.1 (2020.7.13)

supervising editor： Research Group for the Research Project on Ethical, Legal, and Social Issues Supported by the Health, Labour and Welfare Sciences Research Grants “Extraction of ethical and social issues and improvement of social environment toward the realization of a society where people can benefit from genome medicine without anxiety,” Actionability Working Group-Japan

Edited by MONSTAR-SCREEN-2　Medical Genetic Office

***RB1***

**Genetic Change and Hereditary Cancer Syndrome**

- - If genetic changes at birth are responsible for the hereditary cancer syndrome, the patient is considered to be predisposed to cancer.
  - In general, 5-10% of cancers are caused by genetic changes at birth.
  - Cancer susceptibility may be shared by relatives such as parents, children, siblings.
  - Understanding genetic risks allows better health management.
  - A blood test confirms whether the changes observed in genomic testing (tumor profiling) are hereditary.

**What Is *RB1* Related To?**

- Genetic changes in the *RB1* gene causes Hereditary Retinoblastoma (see Table 1).

**The Benefit of Confirmatory Testing**

- It is important to know the cancer risk for prevention and early detection of cancer.


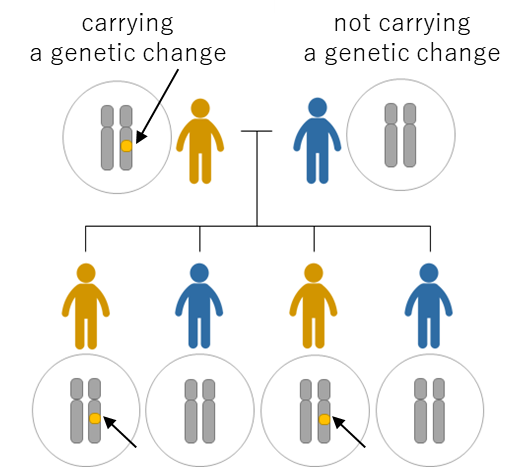
 **Risk of Family Members**

- There are two copies of *RB1*. One and another copy is inherited from the mother, and the other from the father, respectively.
- Each child, brother, sister, and parent have a 50% chance of inheriting genetic changes in *RB1*. Genetic testing can be performed to examine if similar changes are exhibited.
- Aunts, uncles, and cousins may share genetic changes and risks.
- Sharing your genetic information with your relatives may help them manage their health.

※ There is an approximately 80%^1)^ probability that individuals diagnosed with Retinoblastoma did not inherit it. This indicates that the genetic change appears in a family for the first time without the exact genetic change in the parents. Please consult your genetic specialist for information about the impact of such a case on your family.

**What Is Genetic Counseling?**

Genetic Counseling provides information on how genetic conditions may affect you and/or your family　and how to manage it. Please feel free to contact your genetic specialist if you have any relevant questions. ※Genetic testing(s) are not always covered by public health insurance in Japan. Genetic testing and follow-up care for healthy relatives who are unaffected by cancer are not covered by insurance. Please ask Genetic Services for details.

Table 1. Lifetime Risk and Recommended Management

|  | Japanese | Carrier of *RB1* pathogenic / likely pathogenic variant | | | |
| --- | --- | --- | --- | --- | --- |
|  | Lifetime risk^2)^ | Lifetime risk | | Recommended management | |
|  |  | Women^2)^ | Men^2)^ | Women^2)^ | Men^2)^ |
| Retinoblastoma | No data available  ※2.5-4％ in pediatric cancer | 90% | | ・Fundus examination:  Until 3-4y: every 3-4 months  Until 5-6y: every 6 months | |

※The likelihood of being symptomatic if carrying a genetic change and recommended management is based on the Japanese guideline^2)^. Please contact specialists and Genetic Services if management is provided at the hospital.

※Table1 is based on information as of 2021. Recommendations may be revised as research progresses.

【References】

- Retinoblastoma and Neuroblastoma Predisposition and Surveillance. Clin Cancer Res, 2017; 23(13): e98. [ref. 1]
- 日本小児血液がん学会. 小児がん診療ガイドライン (2016年版). (2016年8月) [ref. 2]
- GeneReviews®: Retinoblastoma ver.2018.11.21.
- ClinGen Actionability Reports: RB1 Pediatric. ver.1.3.2 (2021.10.18)

supervising editor： Research Group for the Research Project on Ethical, Legal, and Social Issues Supported by the Health, Labour and Welfare Sciences Research Grants “Extraction of ethical and social issues and improvement of social environment toward the realization of a society where people can benefit from genome medicine without anxiety,” Actionability Working Group-Japan

Edited by MONSTAR-SCREEN-2　Medical Genetic Office

***RET***

**Genetic Change and Hereditary Cancer Syndrome**

- - If genetic changes at birth are responsible for the hereditary cancer syndrome, the patient is considered to be predisposed to cancer.
  - In general, 5-10% of cancers are caused by genetic changes at birth.
  - Cancer susceptibility may be shared by relatives such as parents, children, siblings.
  - Understanding genetic risks allows better health management.
  - A blood test confirms whether the changes observed in genomic testing (tumor profiling) are hereditary.

**What Is *RET* Related To?**

- Genetic changes in the *RET* gene causes Multiple Endocrine Neoplasia Type 2. (see Table 1)

**The Benefit of Confirmatory Testing**

- It is important to know the cancer risk for prevention and early detection of cancer.


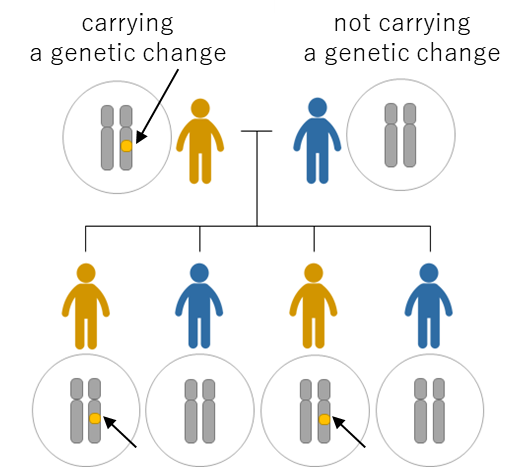
 **Risk of Family Members**

- There are two copies of *RET*. One and another copy is inherited from the mother, and the other from the father, respectively.
- Each child, brother, sister, and parent have a 50% chance of inheriting genetic changes in *RET*. Genetic testing can be performed to examine if similar changes are exhibited.
- Aunts, uncles, and cousins may share genetic changes and risks.
- Sharing your genetic information with your relatives may help them manage their health.

※ It is possible that the *RET* gene change is not inherited from either parent but is a *de* *novo* change. Please consult your genetic specialist for information about the impact of such a case on your family.

**What Is Genetic Counseling?**

Genetic Counseling provides information on how genetic conditions may affect you and/or your family　and how to manage it. Please feel free to contact your genetic specialist if you have any relevant questions. ※Genetic testing(s) are not always covered by public health insurance in Japan. Genetic testing and follow-up care for healthy relatives who are unaffected by cancer are not covered by insurance. Please ask Genetic Services for details.

Table 1. Lifetime Risk and Recommended Management

|  | Japanese | Carrier of *RET* pathogenic / likely pathogenic variant | | | |
| --- | --- | --- | --- | --- | --- |
|  | Lifetime risk^2,3,4)^ | Lifetime risk | | Recommended management | |
|  |  | Women^5)^ | Men^5)^ | Women^5,6)^ | Men^5,6)^ |
| Medullary Thyroid Carcinoma | 0.09 ％ | 90 ％ | | ・Annual blood test (serum calcitonin) and neck ultrasound  ※Starting age for testing depends on the type of the genetic change. For details, please contact a genetic specialist at your institution. | |
| Adrenal Pheochromocytoma | 0.01-0.02 ％ | 30-60 % | | ・Blood test (Plasma-free metanephrines) and abdominal plain CT, or MRI every 2-3 years  ※Starting age for testing depends on the location of the genetic change. For details, please contact a genetic specialist at your institution. | |
| Primary Hyperparathyroidism | 1 in 4,000-5,000 people | 8.1 ％ | | ・Blood test (serum calcium、serum intact PTH), urine test | |

※Other characteristic symptom may occur in association with the genetic change in *RET* gene. Please contact specialists and Genetic Services for further details.

※It is important to note that not all carriers who inherit genetic changes in *RET* are symptomatic.

※The likelihood of being symptomatic if carrying a genetic change and recommended management are based on the Japanese guidelines. Please contact specialists and Genetic Services for details.

※Table1 is based on information as of 2021. Recommendations may be revised as research progresses.

【References】

- GeneReviews Japan: 多発性内分泌腫瘍症2型. ver.2021.9.24 [ref. 1]
- 全国がん登録罹患数・率 報告 平成30年 <https://www.mhlw.go.jp/content/10900000/000794199.pdf> [ref. 2]
- 日本内分泌学会. 褐色細胞腫・パラガングリオーマ診療ガイドライン2018. (2018年7月) [ref. 3]
- Clinical features of multiple endocrine neoplasia type 1 (MEN1) phenocopy without germline MEN1 gene mutations: analysis of 20 Japanese sporadic cases with MEN1. Clin Endocrinol (Oxf), 2000; 52(4): 509. [ref. 4]
- 多発性内分泌腫瘍症診療ガイドブック編集委員会. 多発性内分泌腫瘍症 診療ガイドブック. (2013年4月) [ref. 5]
- RET遺伝子の発症前診断に用いる説明文書 <http://men-net.org/medical/child.html> [ref. 6]
- ClinGen Actionability Reports: RET Adult. ver.2.0.2 (2021.10.18)

supervising editor： Research Group for the Research Project on Ethical, Legal, and Social Issues Supported by the Health, Labour and Welfare Sciences Research Grants “Extraction of ethical and social issues and improvement of social environment toward the realization of a society where people can benefit from genome medicine without anxiety,” Actionability Working Group-Japan

Edited by MONSTAR-SCREEN-2　Medical Genetic Office

***■***　***SDHA***

**Genetic Change and Hereditary Cancer Syndrome**

- - If genetic changes at birth are responsible for the hereditary cancer syndrome, the patient is considered to be predisposed to cancer.
  - In general, 5-10% of cancers are caused by genetic changes at birth.
  - Cancer susceptibility may be shared by relatives such as parents, children, siblings.
  - Understanding genetic risks allows better health management.
  - A blood test confirms whether the changes observed in genomic testing (tumor profiling) are hereditary.

**What Is *SDHA* Related To?**

- *SDHA* has been shown to be associated with an increased risk of cancer. (see Table 1)

**The Benefit of Confirmatory Testing**

- It is important to know the cancer risk for prevention and early detection of cancer.


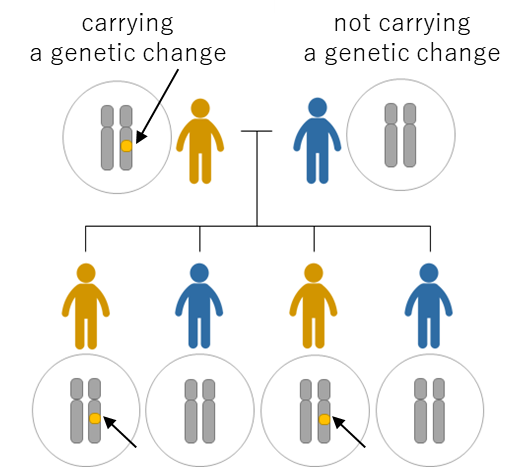
 **Risk of Family Members**

- There are two copies of *SDHA*. One and another copy is inherited from the mother, and the other from the father, respectively.
- Each child, brother, sister, and parent have a 50% chance of inheriting genetic changes in *SDHA*. Genetic testing can be performed to examine if similar changes are exhibited.
- Aunts, uncles, and cousins may share genetic changes and risks.
- Sharing your genetic information with your relatives may help them manage their health.

※ It is possible that the *SDHA* gene change is not inherited from either parent but is a *de* *novo* change. Please consult your genetic specialist for information about the impact of such a case on your family.

**What Is Genetic Counseling?**

Genetic Counseling provides information on how genetic conditions may affect you and/or your family　and how to manage it. Please feel free to contact your genetic specialist if you have any relevant questions. ※Genetic testing(s) are not always covered by public health insurance in Japan. Genetic testing and follow-up care for healthy relatives who are unaffected by cancer are not covered by insurance. Please ask Genetic Services for details.

Table 1. Lifetime Risk and Recommended Management

|  | Japanese | Carrier of *SDHA* pathogenic / likely pathogenic variant | | | |
| --- | --- | --- | --- | --- | --- |
|  | Lifetime risk ^1,2,3)^ | Lifetime risk | | Recommended management | |
|  |  | Women^3)^ | Men^3)^ | Women^3)^ | Men^3)^ |
| Paraganglioma | Japan: 1,500 patients/year  (USA: 2 in 1000,000 people) | Potential for increased risk | | ・Annual Blood test (Plasma-free metanephrines)  ・CT or MRI every 2 years  ・I-MIBG scintigraphy every 3 years | |
| Adrenal Pheochromocytoma | 0.01-0.02 ％ | Potential for increased risk | |  |  |

※It is important to note that not all carriers who inherit genetic changes in *SDHA* are symptomatic.

※The probabilities shown in Table 1 include benign, borderline malignant, and malignant tumors.

※Other conditions, such as gastrointestinal tract tumors, may occur in association with the genetic change in the *SDHA* gene. Please contact specialists and Genetic Services for further details.

※The likelihood of being symptomatic if carrying a genetic change and recommended management is based on the Japanese guideline^3)^. Please contact specialists and Genetic Services if management is provided at the hospital.

※Table1 is based on information as of 2021. Recommendations may be revised as research progresses.

【References】

- 国立がん研究センター希少がんセンター: パラガングリオーマ <https://www.ncc.go.jp/jp/rcc/about/paraganguriouma/index.html> [ref. 1]
- National Cancer Institute Center for Cancer Research <https://www.cancer.gov/pediatric-adult-rare-tumor/rare-tumors/rare-endocrine-tumor/paraganglioma> [ref. 2]
- 日本内分泌学会. 褐色細胞腫・パラガングリオーマ診療ガイドライン2018. (2018年7月) [ref. 3]
- GeneReviews Japan: 遺伝性パラガングリオーマ・褐色細胞腫症候群. ver.2020.7.15
- ClinGen Actionability Reports: MAX, SDHA, SDHAF2, SDHB, SDHC, SDHD, TMEM127 Adult. ver.1.1.3 (2022.1.3)

supervising editor： Research Group for the Research Project on Ethical, Legal, and Social Issues Supported by the Health, Labour and Welfare Sciences Research Grants “Extraction of ethical and social issues and improvement of social environment toward the realization of a society where people can benefit from genome medicine without anxiety,” Actionability Working Group-Japan

Edited by MONSTAR-SCREEN-2　Medical Genetic Office

***SDHAF2***

**Genetic Change and Hereditary Cancer Syndrome**

- - If genetic changes at birth are responsible for the hereditary cancer syndrome, the patient is considered to be predisposed to cancer.
  - In general, 5-10% of cancers are caused by genetic changes at birth.
  - Cancer susceptibility may be shared by relatives such as parents, children, siblings.
  - Understanding genetic risks allows better health management.
  - A blood test confirms whether the changes observed in genomic testing (tumor profiling) are hereditary.

**What Is *SDHAF2* Related To?**

- *SDHAF2* has been shown to be associated with an increased risk of cancer. (see Table 1)

**The Benefit of confirmatory testing**

- It is important to know the cancer risk for prevention and early detection of cancer.


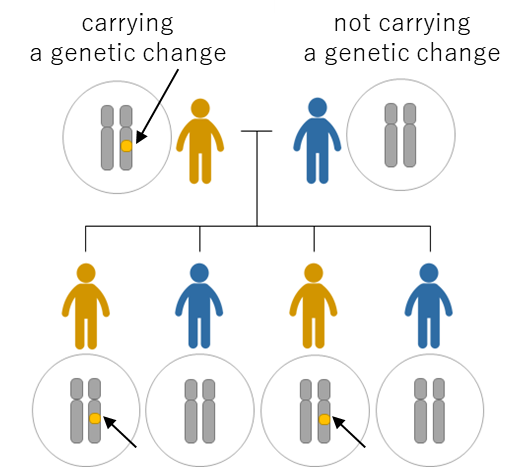
 **Risk of Family Members**

- There are two copies of *SDHAF2*. One and another copy is inherited from the mother, and the other from the father, respectively.
- Each child, brother, sister, and parent have a 50% chance of inheriting genetic changes in *SDHAF2*. Genetic testing can be performed to examine if similar changes are exhibited.
- Aunts, uncles, and cousins may share genetic changes and risks.
- Sharing your genetic information with your relatives may help them manage their health.

※ It is possible that the *SDHAF2* gene change is not inherited from either parent but is a *de* *novo* change. Please consult your genetic specialist for information about the impact of such a case on your family.

**What Is Genetic Counseling?**

Genetic Counseling provides information on how genetic conditions may affect you and/or your family　and how to manage it. Please feel free to contact your genetic specialist if you have any relevant questions. ※Genetic testing(s) are not always covered by public health insurance in Japan. Genetic testing and follow-up care for healthy relatives who are unaffected by cancer are not covered by insurance. Please ask Genetic Services for details.

Table 1. Lifetime Risk and Recommended Management

|  | Japanese | Carrier of *SDHAF2* pathogenic / likely pathogenic variant | | | |
| --- | --- | --- | --- | --- | --- |
|  | Lifetime risk ^1,2,3)^ | Lifetime risk | | Recommended management | |
|  |  | Women^3)^ | Men^3)^ | Women^3)^ | Men^3)^ |
| Paraganglioma | Japan: 1,500 patients/year  (USA: 2 in 1000,000 people) | Potential for increased risk | | ・Annual Blood test (Plasma-free metanephrines)  ・CT or MRI every 2 years  ・I-MIBG scintigraphy every 3 years | |
| Adrenal Pheochromocytoma | 0.01-0.02 ％ | Potential for increased risk | |  |  |

※It is important to note that not all carriers who inherit genetic changes in *SDHAF2* are symptomatic.

※The probabilities shown in Table 1 include benign, borderline malignant, and malignant tumors.

※The likelihood of being symptomatic if carrying a genetic change and recommended management is based on the Japanese guideline^3)^. Please contact specialists and Genetic Services if management is provided at the hospital.

※Table1 is based on information as of 2021. Recommendations may be revised as research progresses in the future.

【References】

- 国立がん研究センター希少がんセンター: パラガングリオーマ <https://www.ncc.go.jp/jp/rcc/about/paraganguriouma/index.html> [ref. 1]
- National Cancer Institute Center for Cancer Research <https://www.cancer.gov/pediatric-adult-rare-tumor/rare-tumors/rare-endocrine-tumor/paraganglioma> [ref. 2]
- 日本内分泌学会. 褐色細胞腫・パラガングリオーマ診療ガイドライン2018. (2018年7月) [ref. 3]
- GeneReviews Japan: 遺伝性パラガングリオーマ・褐色細胞腫症候群. ver.2020.7.15
- ClinGen Actionability Reports: MAX, SDHA, SDHAF2, SDHB, SDHC, SDHD, TMEM127 Adult. ver.1.1.3 (2022.1.3)

supervising editor： Research Group for the Research Project on Ethical, Legal, and Social Issues Supported by the Health, Labour and Welfare Sciences Research Grants “Extraction of ethical and social issues and improvement of social environment toward the realization of a society where people can benefit from genome medicine without anxiety,” Actionability Working Group-Japan

Edited by MONSTAR-SCREEN-2　Medical Genetic Office

***SDHB***

**Genetic Change and Hereditary Cancer Syndrome**

- - If genetic changes at birth are responsible for the hereditary cancer syndrome, the patient is considered to be predisposed to cancer.
  - In general, 5-10% of cancers are caused by genetic changes at birth.
  - Cancer susceptibility may be shared by relatives such as parents, children, siblings.
  - Understanding genetic risks allows better health management.
  - A blood test confirms whether the changes observed in genomic testing (tumor profiling) are hereditary.

**What Is *SDHB* Related To?**

- *SDHB* has been shown to be associated with an increased risk of cancer. (see Table 1)

**The Benefit of Confirmatory Testing**

- It is important to know the cancer risk for prevention and early detection of cancer.


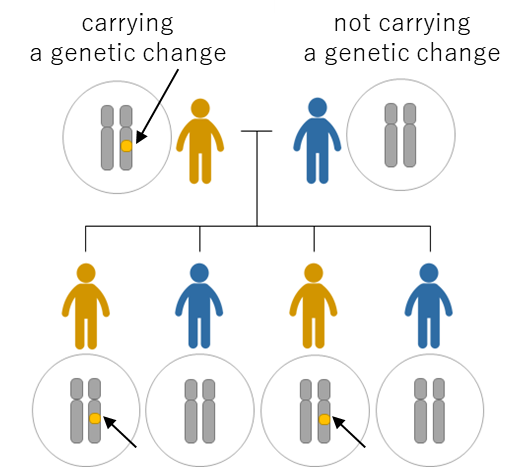
 **Risk of Family Members**

- There are two copies of *SDHB*. One and another copy is inherited from the mother, and the other from the father, respectively.
- Each child, brother, sister, and parent have a 50% chance of inheriting genetic changes in *SDHB*. Genetic testing can be performed to examine if similar changes are exhibited.
- Aunts, uncles, and cousins may share genetic changes and risks.
- Sharing your genetic information with your relatives may help them manage their health.

※ It is possible that the *SDHB* gene change is not inherited from either parent but is a *de* *novo* change. Please consult your genetic specialist for information about the impact of such a case on your family.

**What Is Genetic Counseling?**

Genetic Counseling provides information on how genetic conditions may affect you and/or your family　and how to manage it. Please feel free to contact your genetic specialist if you have any relevant questions. ※Genetic testing(s) are not always covered by public health insurance in Japan. Genetic testing and follow-up care for healthy relatives who are unaffected by cancer are not covered by insurance. Please ask Genetic Services for details.

Table 1. Lifetime Risk and Recommended Management

|  | Japanese | Carrier of *SDHB* pathogenic / likely pathogenic variant | | | |
| --- | --- | --- | --- | --- | --- |
|  | Lifetime risk^1,2,3)^ | Lifetime risk | | Recommended management | |
|  |  | Women^3)^ | Men^3)^ | Women^3)^ | Men^3)^ |
| Paraganglioma | Japan: 1,500 patients/year  (USA: 2 patients/million) | 30 ％ | | - Annual Blood test (Plasma-free metanephrines) - CT or MRI every 2 years - I-MIBG scintigraphy every 3 years | |
| Adrenal Pheochromocytoma | 0.01-0.02 ％ |  |  |  |  |

※It is important to note that not all carriers who inherit genetic change in *SDHB* are symptomatic.

※The probabilities shown in Table 1 include benign, borderline malignant, and malignant tumors. 34-97% of tumors in individuals with genetic changes in *SDHB* are at risk of malignancy^3)^.

※Other conditions, such as gastrointestinal tract tumors, may occur in association with the genetic change in the *SDHB* gene. Please contact specialists and Genetic Services for further details.

※The likelihood of being symptomatic if carrying a genetic change and recommended management is based on the Japanese guideline^3)^. Please contact specialists and Genetic Services if management is provided at the hospital.

※Table1 is based on information as of 2021. Recommendations may be revised as research progresses.

【References】

- 国立がん研究センター希少がんセンター: パラガングリオーマ <https://www.ncc.go.jp/jp/rcc/about/paraganguriouma/index.html> [ref. 1]
- National Cancer Institute Center for Cancer Research <https://www.cancer.gov/pediatric-adult-rare-tumor/rare-tumors/rare-endocrine-tumor/paraganglioma> [ref. 2]
- 日本内分泌学会. 褐色細胞腫・パラガングリオーマ診療ガイドライン2018. (2018年7月) [ref. 3]
- GeneReviews Japan: 遺伝性パラガングリオーマ・褐色細胞腫症候群. ver.2020.7.15
- ClinGen Actionability Reports: MAX, SDHA, SDHAF2, SDHB, SDHC, SDHD, TMEM127 Adult. ver.1.1.3 (2022.1.3)

supervising editor： Research Group for the Research Project on Ethical, Legal, and Social Issues Supported by the Health, Labour and Welfare Sciences Research Grants “Extraction of ethical and social issues and improvement of social environment toward the realization of a society where people can benefit from genome medicine without anxiety,” Actionability Working Group-Japan

Edited by MONSTAR-SCREEN-2　Medical Genetic Office

***SDHC***

**Genetic Change and Hereditary Cancer Syndrome**

- - If genetic changes at birth are responsible for the hereditary cancer syndrome, the patient is considered to be predisposed to cancer.
  - In general, 5-10% of cancers are caused by genetic changes at birth.
  - Cancer susceptibility may be shared by relatives such as parents, children, siblings.
  - Understanding genetic risks allows better health management.
  - A blood test confirms whether the changes observed in genomic testing (tumor profiling) are hereditary.

**What Is *SDHC* Related To?**

- *SDHC* has been shown to be associated with an increased risk of cancer. (see Table 1)

**The Benefit of Confirmatory Testing**

- It is important to know the cancer risk for prevention and early detection of cancer.


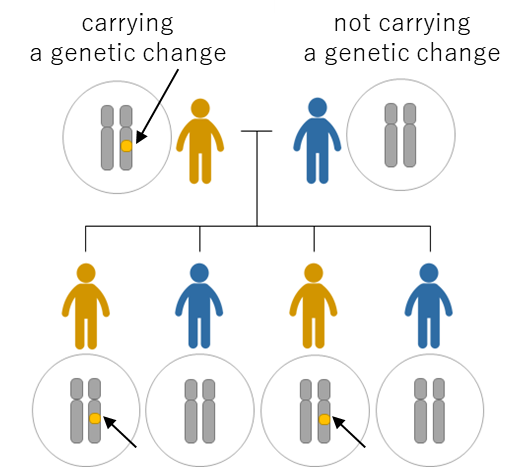
 **Risk of Family Members**

- There are two copies of *SDHC*. One and another copy is inherited from the mother, and the other from the father, respectively.
- Each child, brother, sister, and parent have a 50% chance of inheriting genetic changes in *SDHC*. Genetic testing can be performed to examine if similar changes are exhibited.
- Aunts, uncles, and cousins may share genetic changes and risks.
- Sharing your genetic information with your relatives may help them manage their health.

※ It is possible that the *SDHC* gene change is not inherited from either parent, but is a *de* *novo* change. Please consult your genetic specialist for information about the impact of such a case on your family.

**What Is Genetic Counseling?**

Genetic Counseling provides information on how genetic conditions may affect you and/or your family　and how to manage it. Please feel free to contact your genetic specialist if you have any relevant questions. ※Genetic testing(s) are not always covered by public health insurance in Japan. Genetic testing and follow-up care for healthy relatives who are unaffected by cancer are not covered by insurance. Please ask Genetic Services for details.

Table 1. Lifetime Risk and Recommended Management

|  | Japanese | Carrier of *SDHC* pathogenic / likely pathogenic variant | | | |
| --- | --- | --- | --- | --- | --- |
|  | Lifetime risk ^1,2,3)^ | Lifetime risk | | Recommended management | |
|  |  | Women^3)^ | Men^3)^ | Women^3)^ | Men^3)^ |
| Paraganglioma | Japan: 1,500 patients/year  (USA: 2 in 1000,000 people) | Potential for increased risk | | ・Annual Blood test (Plasma-free metanephrines)  ・CT or MRI every 2 years  ・I-MIBG scintigraphy every 3　years | |
| Adrenal Pheochromocytoma | 0.01-0.02 ％ | Potential for increased risk | |  |  |

※It is important to note that not all carriers who inherit genetic changes in *SDHC* be symptomatic.

※The probabilities shown in Table 1 include benign, borderline malignant, and malignant tumors.

※Other conditions, such as gastrointestinal tract tumors, may occur in association with the genetic change in the *SDHC* gene. Please contact specialists and Genetic Services for further details.

※The likelihood of being symptomatic if carrying a genetic change and recommended management is based on the Japanese guideline^3)^. Please contact specialists and Genetic Services if management is provided at the hospital.

※Table1 is based on information as of 2021. Recommendations may be revised as research progresses.

【References】

- 国立がん研究センター希少がんセンター: パラガングリオーマ <https://www.ncc.go.jp/jp/rcc/about/paraganguriouma/index.html> [ref. 1]
- National Cancer Institute Center for Cancer Research <https://www.cancer.gov/pediatric-adult-rare-tumor/rare-tumors/rare-endocrine-tumor/paraganglioma> [ref. 2]
- 日本内分泌学会. 褐色細胞腫・パラガングリオーマ診療ガイドライン2018. (2018年7月) [ref. 3]
- GeneReviews Japan: 遺伝性パラガングリオーマ・褐色細胞腫症候群. ver.2020.7.15
- ClinGen Actionability Reports: MAX, SDHA, SDHAF2, SDHB, SDHC, SDHD, TMEM127 Adult. ver.1.1.3 (2022.1.3)

supervising editor： Research Group for the Research Project on Ethical, Legal, and Social Issues Supported by the Health, Labour and Welfare Sciences Research Grants “Extraction of ethical and social issues and improvement of social environment toward the realization of a society where people can benefit from genome medicine without anxiety,” Actionability Working Group-Japan

Edited by MONSTAR-SCREEN-2　Medical Genetic Office

***SDHD***

**Genetic Change and Hereditary Cancer Syndrome**

- - If genetic changes at birth are responsible for the hereditary cancer syndrome, the patient is considered to be predisposed to cancer.
  - In general, 5-10% of cancers are caused by genetic changes at birth.
  - Cancer susceptibility may be shared by relatives such as parents, children, siblings.
  - Understanding genetic risks allows better health management.
  - A blood test confirms whether the changes observed in genomic testing (tumor profiling) are hereditary.

**What Is *SDHD* Related To?**

- *SDHD* has been shown to be associated with an increased risk of cancer. (see Table 1)

**The Benefit of Confirmatory Testing**

- It is important to know the cancer risk for prevention and early detection of cancer.


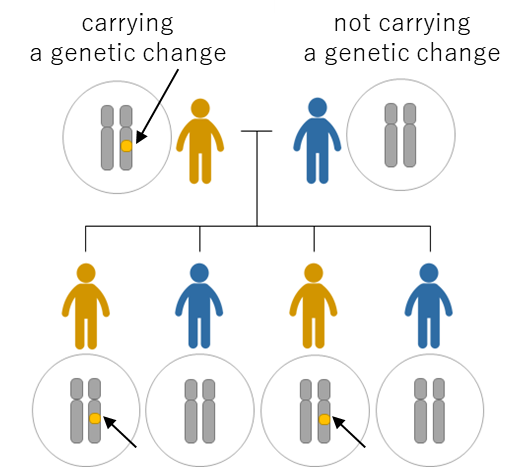
 **Risk of Family Members**

- There are two copies of *SDHD*. One and another copy is inherited from the mother, and the other from the father, respectively.
- Each child, brother, sister, and parent have a 50% chance of inheriting genetic changes in *SDHD*. Genetic testing can be performed to examine if similar changes are exhibited.
- Aunts, uncles, and cousins may share genetic changes and risks.
- Sharing your genetic information with your relatives may help them manage their health.

※ It is possible that the *SDHD* gene change is not inherited from either parent but is a *de* *novo* change. Please consult your genetic specialist for information about the impact of such a case on your family.

**What Is Genetic Counseling?**

Genetic Counseling provides information on how genetic conditions may affect you and/or your family　and how to manage it. Please feel free to contact your genetic specialist if you have any relevant questions. ※Genetic testing(s) are not always covered by public health insurance in Japan. Genetic testing and follow-up care for healthy relatives who are unaffected by cancer are not covered by insurance. Please ask Genetic Services for details.

Table 1. Lifetime Risk and Recommended Management

|  | Japanese | Carrier of *SDHD* pathogenic / likely pathogenic variant | | | |
| --- | --- | --- | --- | --- | --- |
|  | Lifetime risk ^1,2,3)^ | Lifetime risk | | Recommended management | |
|  |  | Women^3)^ | Men^3)^ | Women^3)^ | Men^3)^ |
| Paraganglioma | Japan: 1,500 patients/year  (USA: 2 in 1000,000 people) | Potential for increased risk | | ・Annual Blood test (Plasma-free metanephrines)  ・CT or MRI every 2 years  ・I-MIBG scintigraphy every 3 years | |
| Adrenal Pheochromocytoma | 0.01-0.02 ％ | Potential for increased risk | |  |  |

※It is important to note that not all carriers who inherit genetic changes in *SDHD* are symptomatic.

※The probabilities shown in Table 1 include benign, borderline malignant, and malignant tumors.

※Other conditions, such as gastrointestinal tract tumors, may occur in association with the genetic change in the *SDHD* gene. Please contact specialists and Genetic Services for further details.

※The likelihood of being symptomatic if carrying a genetic change and recommended management is based on the Japanese guideline^3)^. Please contact specialists and Genetic Services if management is provided at the hospital.

※Table1 is based on information as of 2021. Recommendations may be revised as research progresses.

【References】

- 国立がん研究センター希少がんセンター: パラガングリオーマ <https://www.ncc.go.jp/jp/rcc/about/paraganguriouma/index.html> [ref. 1]
- National Cancer Institute Center for Cancer Research <https://www.cancer.gov/pediatric-adult-rare-tumor/rare-tumors/rare-endocrine-tumor/paraganglioma> [ref. 2]
- 日本内分泌学会. 褐色細胞腫・パラガングリオーマ診療ガイドライン2018. (2018年7月) [ref. 3]
- GeneReviews Japan: 遺伝性パラガングリオーマ・褐色細胞腫症候群. ver.2020.7.15
- ClinGen Actionability Reports: MAX, SDHA, SDHAF2, SDHB, SDHC, SDHD, TMEM127 Adult. ver.1.1.3 (2022.1.3)

supervising editor： Research Group for the Research Project on Ethical, Legal, and Social Issues Supported by the Health, Labour and Welfare Sciences Research Grants “Extraction of ethical and social issues and improvement of social environment toward the realization of a society where people can benefit from genome medicine without anxiety,” Actionability Working Group-Japan

Edited by MONSTAR-SCREEN-2　Medical Genetic Office

***SMAD3***

**What Is *SMAD3* Related To?**

- - *SMAD3* is a gene that has been shown to be associated with risk of cardiac disease.
  - A genetic change in the *SMAD3* gene causes Loeys-Dietz Syndrome. (see Table 1)
  - A blood test will confirm whether the changes found in the genetic testing (tumor profiling) are hereditary.

**The Benefit of Confirmatory Testing**

- It is important to know the risk of cardiovascular disease for early detection.


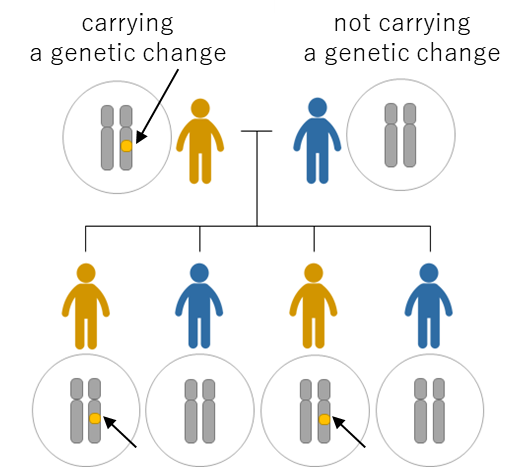
 **Risk of Family Members**

- There are two copies of *SMAD3*. One and another copy is inherited from the mother, and the other from the father, respectively.
- Each child, brother, sister, and parent have a 50% chance of inheriting genetic changes in *SMAD3*. Genetic testing can be performed to examine if similar changes are exhibited.
- Aunts, uncles, and cousins may share genetic changes and risks.
- Sharing your genetic information with your relatives may help them manage their health.

※ It is possible that the *SMAD3* gene change is not inherited from either parent but is a *de* *novo* change. Please consult your genetic specialist for information about the impact of such a case on your family.

※ There is an approximately 75%^2)^ probability that individuals diagnosed with Loeys-Dietz Syndrome did not inherit it. This indicates that the genetic change appears in a family for the first time without the exact genetic change in the parents. Please consult your genetic specialist for information about the impact of such a case on your family.

**What is Genetic Counseling?**

Genetic Counseling provides information on how genetic conditions may affect you and/or your family　and how to manage it. Please feel free to contact your genetic specialist if you have any relevant questions. ※Genetic testing(s) are not always covered by public health insurance in Japan. Genetic testing and follow-up care for healthy relatives who are unaffected by cancer are not covered by insurance. Please ask Genetic Services for details.

Table 1. Lifetime Risk and Recommended Management

|  | Japanese | Carrier of *SMAD3* pathogenic / likely pathogenic variant | |
| --- | --- | --- | --- |
|  | Lifetime risk^1)^ | Lifetime risk^2)^ | Recommended management^1)^ |
| Aortic Aneurysm/ Dissection | 3-10 in 100,000 people/year | 95% | ・Magnetic resonance angiography (MRA) or CT angiogram (CTA)  ・If aortic root dilatation is seen, consider aortic root replacement  ・Beta-adrenergic blockers or angiotensin receptor blockers: treatment for hypertension |

※Other characteristic skeletal and/or skin symptoms may occur in association with the genetic change in *SMAD3* gene.

※It is important to note that not all carriers who inherit genetic changes in *SMAD3* are symptomatic.

※The likelihood of being symptomatic if carrying a genetic change and recommended managements are based on the Japanese guideline^1)^. Please contact specialists and Genetic Services for further details.

【References】

- 日本循環器学会・日本心臓血管外科学会・日本胸部外科学会・日本血管外科学会合同ガイドライン. 大動脈瘤・大動脈解離診療ガイドライン 2020年改訂版. (2020年7月) [ref. 1]
- GeneReviews Japan: ロイス・ディーツ症候群. ver.2021.4.30 [ref. 2]
- ClinGen Actionability Reports: SMAD3, TGFB2, TGFB3, TGFBR1, TGFBR2 Adult. ver.3.0.4 (2021.8.24)

supervising editor： Research Group for the Research Project on Ethical, Legal, and Social Issues Supported by the Health, Labour and Welfare Sciences Research Grants “Extraction of ethical and social issues and improvement of social environment toward the realization of a society where people can benefit from genome medicine without anxiety,” Actionability Working Group-Japan

Edited by MONSTAR-SCREEN-2　Medical Genetic Office

***SMAD4***

**Genetic Change and Hereditary Cancer Syndrome**

- - If genetic changes at birth are responsible for the hereditary cancer syndrome, the patient is considered to be predisposed to cancer.
  - In general, 5-10% of cancers are caused by genetic changes at birth.
  - Cancer susceptibility may be shared by relatives such as parents, children, siblings.
  - Understanding genetic risks allows better health management.
  - A blood test confirms whether the changes observed in genomic testing (tumor profiling) are hereditary.

**What Is *SMAD4* Related To?**

- Genetic changes in the *SMAD4* gene causes Juvenile Polyposis Syndrome (see Table 1).

**The Benefit of Confirmatory Testing**

- It is important to know the cancer risk for prevention and early detection of cancer.


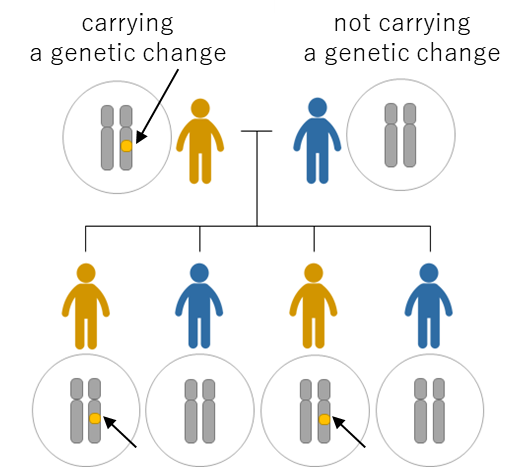
 **Risk of Family Members**

- There are two copies of *SMAD4*. One and another copy is inherited from the mother, and the other from the father, respectively.
- Each child, brother, sister, and parent have a 50% chance of inheriting genetic changes in *SMAD4*. Genetic testing can be performed to examine if similar changes are exhibited.
- Aunts, uncles, and cousins may share genetic changes and risks.
- Sharing your genetic information with your relatives may help them manage their health.

※ It is possible that the *SMAD4* gene change is not inherited from either parent but is a *de* *novo* change. Please consult your genetic specialist for information about the impact of such a case on your family.

**What Is Genetic Counseling?**

Genetic Counseling provides information on how genetic conditions may affect you and/or your family　and how to manage it. Please feel free to contact your genetic specialist if you have any relevant questions. ※Genetic testing(s) are not always covered by public health insurance in Japan. Genetic testing and follow-up care for healthy relatives who are unaffected by cancer are not covered by insurance. Please ask Genetic Services for details.

Table 1. Lifetime Risk and Recommended Management

|  | Japanese | Carrier of *SMAD4* pathogenic / likely pathogenic variant | | | |
| --- | --- | --- | --- | --- | --- |
|  | Lifetime risk^1,2)^ | Lifetime risk | | Recommended management | |
|  |  | Women^3,4)^ | Men^3,4)^ | Women^3,5)^ | Men^3,5)^ |
| Colon Polyp  (Risk of cancer) | Colorectal cancer  10.2 ％ (Men)  8.0 ％ (Women) | Colon polyp: 97 %  Colorectal cancer: ≦50 % | | ・Upper endoscopy and colonoscopy every 1-3 years | |
| Gastric Polyp  (Risk of cancer) | Gastric cancer  10.3 ％ (Men)  4.7 ％ (Women) | Gastric polyp: 68 %  Gastric cancer: ≦21 % | |  |  |
| Small Intestine Polyp | Small intestinal cancer  0.2% (Men)  0.1% (Women) | Low malignancy risk | |  |  |

※It is important to note that not all carriers who inherit genetic changes in *SMAD4* develop cancer. Most patients with juvenile polyposis syndrome are found to have several polyps by the age of 20. Please contact specialists and Genetic Services for details.

※Other conditions, such as cardiovascular malformation, may occur in association with the genetic change in the *SMAD4* gene. Please contact specialists and Genetic Services for further details.

※The likelihood of being symptomatic if carrying a genetic change and recommended management is based on the Japanese guideline^3)^ and the National Comprehensive Cancer Network guideline^5)^. The recommendations may not always be implemented in Japan. Please contact specialists and Genetic Services if management is provided at the hospital.

※Table1 is based on information as of 2021. Recommendations may be revised as research progresses.

【References】

- 国立がん研究センターがん情報サービス「累積がん罹患リスク (2018年データ)」<https://ganjoho.jp/reg_stat/statistics/stat/summary.html> [ref. 1]
- 厚生労働省健康局がん・疾病対策課. 平成30年全国がん登録 罹患数・率報告 2018 [ref. 2]
- 小児・成人のための若年性ポリポーシス 症候群診療ガイドライン 2020年版. 遺伝性腫瘍, 2020; 20(2): 79. [ref. 3]
- Appreciating the broad clinical features of SMAD4 mutation carriers: multicenter chart review. Genet Med, 2014; 16(8): 588. [ref. 4]
- NCCN Guidelines® Genetic/Familial High-Risk Assessment: Colorectal. ver.1.2021 (2021.5.11) [ref. 5]
- GeneReviews Japan: 若年性ポリポーシス症候群. ver.2014.3.3. ClinGen Actionability Reports: SMAD4, BMPR1A Adult. ver.1.1.1 (2021.1.19)

supervising editor： Research Group for the Research Project on Ethical, Legal, and Social Issues Supported by the Health, Labour and Welfare Sciences Research Grants “Extraction of ethical and social issues and improvement of social environment toward the realization of a society where people can benefit from genome medicine without anxiety,” Actionability Working Group-Japan

Edited by MONSTAR-SCREEN-2　Medical Genetic Office

***SMARCB1***

**Genetic Change and Hereditary Cancer Syndrome**

- - If genetic changes at birth are responsible for the hereditary cancer syndrome, the patient is considered to be predisposed to cancer.
  - In general, 5-10% of cancers are caused by genetic changes at birth.
  - Cancer susceptibility may be shared by relatives such as parents, children, siblings.
  - Understanding genetic risks allows better health management.
  - A blood test confirms whether the changes observed in genomic testing (tumor profiling) are hereditary.

**What Is *SMARCB1* Related To?**

- Genetic changes in the *SMARCB1* gene causes Rhabdoid Tumor Predisposition Syndrome 1 (see Table 1).

**The Benefit of Confirmatory Testing**

- It is important to know the cancer risk for prevention and early detection of cancer.


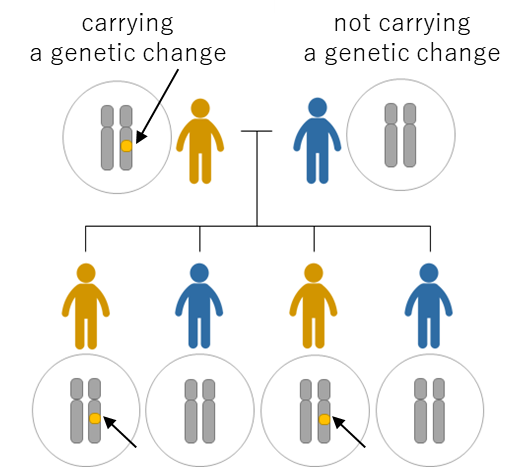
 **Risk of Family Members**

- There are two copies of *SMARCB1*. One and another copy is inherited from the mother, and the other from the father, respectively.
- Each child, brother, sister, and parent have a 50% chance of inheriting genetic changes in *SMARCB1*. Genetic testing can be performed to examine if similar changes are exhibited.
- Aunts, uncles, and cousins may share genetic changes and risks.
- Sharing your genetic information with your relatives may help them manage their health.

※ It is possible that the *SMARCB1* gene change is not inherited from either parent but is a *de* *novo* change. Please consult your genetic specialist for information about the impact of such a case on your family.

**What Is Genetic Counseling?**

Genetic Counseling provides information on how genetic conditions may affect you and/or your family　and how to manage it. Please feel free to contact your genetic specialist if you have any relevant questions. ※Genetic testing(s) are not always covered by public health insurance in Japan. Genetic testing and follow-up care for healthy relatives who are unaffected by cancer are not covered by insurance. Please ask Genetic Services for details.

Table 1. Lifetime Risk and Recommended Management

|  | Japanese | Carrier of *SMARCB1* pathogenic / likely pathogenic variant | | | |
| --- | --- | --- | --- | --- | --- |
|  | Lifetime risk^2)^ | Lifetime risk | | Recommended management | |
|  |  | Women^2)^ | Men^2)^ | Women^2)^ | Men^2)^ |
| Brain Tumor  (Rhabdoid Tumor) | 1-3 in 10,000 people/year | No data available | | ・From birth to 5y: brain MRI every 3 months* | |
| Renal Tumor  (Rhabdoid Tumor) | Under 15y:  1-2 in 10,000 people | No data available | | ・From birth to 5y: abdominal ultrasound every 3 months* | |
| Chondrosarcoma  (Rhabdoid Tumor) | No data available | No data available | | ・From birth to 5y: whole-body MRI* | |

* The recommended management in Table 1 is not specifically presented in Japan. Please contact the genetic specialist at your institution for further details.

※It is important to note that not all carriers who inherit genetic changes in *SMARCB1* are symptomatic.

※Table1 is based on information as of 2021. Recommendations may be revised as research progresses.

【References】

- GeneReviews: Rhabdoid Tumor Predisposition Syndrome. ver.2017.12.7 [ref. 1]
- ClinGen Actionability Reports: SMARCA4, SMARCB1 Adult. ver.1.0.0 (2021.8.2) [ref. 2]

supervising editor： Research Group for the Research Project on Ethical, Legal, and Social Issues Supported by the Health, Labour and Welfare Sciences Research Grants “Extraction of ethical and social issues and improvement of social environment toward the realization of a society where people can benefit from genome medicine without anxiety,” Actionability Working Group-Japan

Edited by MONSTAR-SCREEN-2　Medical Genetic Office

***STK11***

**Genetic Change and Hereditary Cancer Syndrome**

- - If genetic changes at birth are responsible for the hereditary cancer syndrome, the patient is considered to be predisposed to cancer.
  - In general, 5-10% of cancers are caused by genetic changes at birth.
  - Cancer susceptibility may be shared by relatives such as parents, children, siblings.
  - Understanding genetic risks allows better health management.
  - A blood test confirms whether the changes observed in genomic testing (tumor profiling) are hereditary.

**What Is *STK11* Related To?**

- Genetic changes in the *STK11* gene causes Peutz-Jeghers Syndrome (see Table 1).

**The Benefit of Confirmatory Testing**

- It is important to know the cancer risk for prevention and early detection of cancer.


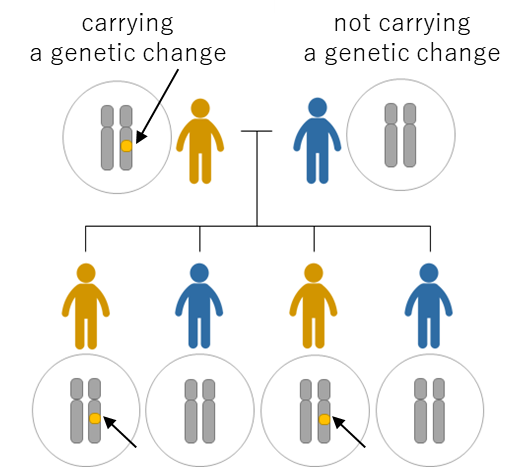
 **Risk of Family Members**

- There are two copies of *STK11*. One and another copy is inherited from the mother, and the other from the father, respectively.
- Each child, brother, sister, and parent have a 50% chance of inheriting genetic changes in *STK11*. Genetic testing can be performed to examine if similar changes are exhibited.
- Aunts, uncles, and cousins may share genetic changes and risks.
- Sharing your genetic information with your relatives may help them manage their health.

※ It is possible that the *STK11* gene change is not inherited from either parent but is a *de* *novo* change. Please consult your genetic specialist for information about the impact of such a case on your family.

**What Is Genetic Counseling?**

Genetic Counseling provides information on how genetic conditions may affect you and/or your family　and how to manage it. Please feel free to contact your genetic specialist if you have any relevant questions. ※Genetic testing(s) are not always covered by public health insurance in Japan. Genetic testing and follow-up care for healthy relatives who are unaffected by cancer are not covered by insurance. Please ask Genetic Services for details.

Table 1. Lifetime Risk and Recommended Management

|  | Japanese | Carrier of *STK11* pathogenic / likely pathogenic variant | | | | |
| --- | --- | --- | --- | --- | --- | --- |
|  | Lifetime risk^1,2)^ | Lifetime risk | | Recommended management | |  |
|  |  | Women^3,4)^ | Men^3,4)^ | Women^3,4)^ | Men^3,4)^ |  |
| Colon Polyp/Cancer | (Colorectal cancer)  10.2 ％ (Men)  8.0 ％ (Women) | (Polyp) 24-27 %  (Cancer) 36.4 % | | ・Upper endoscopy, colonoscopy, and capsule endoscopy  ・Starting at age 18y：every 3 years  ・Over 50y：every 1-2 years | |  |
| Gastric Polyp/Cancer | (Gastric cancer)  10.3 ％ (Men)  4.7 ％ (Women) | (Polyp) 24 %  (Cancer) 24 % | |  |  |  |
| Small Intestine Polyp/Cancer | (Small intestinal cancer)  0.2% (Men)  0.1% (Women) | (Polyp) 96 %  （Cancer）13.8 % | |  |  |  |
| Breast Cancer | 10.9 ％ (Women) | 19.3 % | ― | ・Starting at age 18y: annual breast awareness  ・Starting at age 25y: annual breast MRI screening with contrast or ultrasound  ・Over 50y: annual mammogram | ― |  |
| Cervical Cancer | 1.3 % (Women) | (Uterine cancer)  46.5% | ― | ・Starting at ages 18-25y: cervical cytology every 1-3 years | ― |  |
| Endometrial Cancer | 2.0% (Women) |  | ― | ・Starting at age 18-25y: annual gynecological examination | ― |  |
| Ovarian Cancer | 1.6% (Women) | 10.1 % | ― |  |  |  |
| Pancreatic Cancer | 2.6 ％ (Men)  2.6 ％ (Women) | 29.4 % | | ・Starting at age 30y: MRI/magnetic resonance cholangiopancreatography (MRCP) and/or endoscopic  ultrasound (EUS) every 1-2 years | |  |
| Testicular Tumor | 0.3% (Men) | ― | 9 % | ・Until adult： annual palpation/ultrasound (if an abnormality is found on palpation) | |  |
| Lung Cancer | 9.9 ％ (Men)  4.9 ％ (Women) | 7.6 % | | ・Consider cancer screening earlier than general population | |  |

※It is important to note that not all carriers who inherit genetic changes in *STK11* develop cancer.

※The likelihood of being symptomatic if carrying a genetic change and recommended management is based on the Japanese guideline^3)^ and the National Comprehensive Cancer Network guideline^4)^. The recommendations may not always be implemented in Japan. Please contact specialists and Genetic Services if management is provided at the hospital.

※Table1 is based on information as of 2021. Recommendations may be revised as research progresses.

【References】

- 国立がん研究センターがん情報サービス「累積がん罹患リスク (2018年データ)」 <https://ganjoho.jp/reg_stat/statistics/stat/summary.html> [ref. 1]
- 厚生労働省健康局がん・疾病対策課. 平成30年全国がん登録 罹患数・率報告 2018 [ref. 2]
- 小児・成人のためのPeutz-Jeghers症候群診療ガイドライン 2020年版. 遺伝性腫瘍, 2020; 20(2): 59. [ref. 3]
- NCCN Guidelines® Genetic/Familial High-Risk Assessment: Colorectal. ver.1.2021 (2021.5.11) [ref. 4]
- GeneReviews Japan: Peutz-Jeghers症候群. ver.2021.11.9 [ref. 5]
- ClinGen Actionability Reports: STK11 Adult. ver.2.0.1 (2021.3.29) [ref. 6]

supervising editor： Research Group for the Research Project on Ethical, Legal, and Social Issues Supported by the Health, Labour and Welfare Sciences Research Grants “Extraction of ethical and social issues and improvement of social environment toward the realization of a society where people can benefit from genome medicine without anxiety,” Actionability Working Group-Japan

Edited by MONSTAR-SCREEN-2　Medical Genetic Office

***TERF2IP***

**Genetic Change and Hereditary Cancer Syndrome**

- - If genetic changes at birth are responsible for the hereditary cancer syndrome, the patient is considered to be predisposed to cancer.
  - In general, 5-10% of cancers are caused by genetic changes at birth.
  - Cancer susceptibility may be shared by relatives such as parents, children, siblings.
  - Understanding genetic risks allows better health management.
  - A blood test confirms whether the changes observed in genomic testing (tumor profiling) are hereditary.

**What Is *TERF2IP* Related To?**

- *TERF2IP* has been shown to be associated with an increased risk of cancer^1)^ (see Table 1).

**The Benefit of Confirmatory Testing**

- It is important to know the cancer risk for prevention and early detection of cancer.


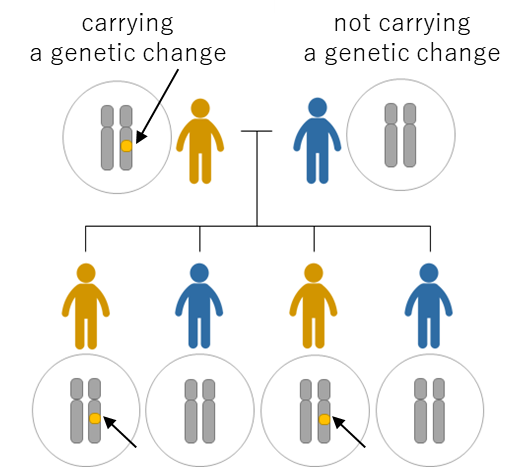
 **Risk of Family Members**

- There are two copies of *TERF2IP*. One and another copy is inherited from the mother, and the other from the father, respectively.
- Each child, brother, sister, and parent have a 50% chance of inheriting genetic changes in *TERF2IP*. Genetic testing can be performed to examine if similar changes are exhibited.
- Aunts, uncles, and cousins may share genetic changes and risks.
- Sharing your genetic information with your relatives may help them manage their health.

※ It is possible that the *TERF2IP* gene change is not inherited from either parent but is a *de* *novo* change. Please consult your genetic specialist for information about the impact of such a case on your family.

**What Is Genetic Counseling?**

Genetic Counseling provides information on how genetic conditions may affect you and/or your family　and how to manage it. Please feel free to contact your genetic specialist if you have any relevant questions. ※Genetic testing(s) are not always covered by public health insurance in Japan. Genetic testing and follow-up care for healthy relatives who are unaffected by cancer are not covered by insurance. Please ask Genetic Services for details.

Table 1. Lifetime Risk and Recommended Management

|  | Japanese | Carrier of *TERF2IP* pathogenic / likely pathogenic variant | | | |
| --- | --- | --- | --- | --- | --- |
|  | Lifetime risk^2)^ | Lifetime risk | | Recommended management | |
|  |  | Women^1)^ | Men^1)^ | Women^4)^ | Men^4)^ |
| Melanoma* | 0.1 % (Men)  0.1 % (Women) | Potential increased risk | | ・Comprehensive skin examination by a dermatologist, including total body skin, scalp, oral mucosa, and genitals every 6 months (if the nevus is stable: every 12 month) **  ・Monthly nevus self-examination** | |

* The annual prevalence of malignant melanoma is reported to be 24.3 in 100,000 people for Caucasians and 1.7 in 100,000 people for Asians^6)^, and the risk is considered to vary according to race, region, and other genetic factors.

** The recommended management in Table 1 is not specifically presented in Japan. Please contact the genetic specialist at your institution for further details.

※It is important to note that not all carriers who inherit genetic changes in *TERF2IP* develop cancer.

※The likelihood of being symptomatic if carrying a genetic change and recommended management is based on international data^1,4)^.

※Table1 is based on information as of 2021. Recommendations may be revised as research progresses.

【References】

- *CDKN2A* genetic testing in melanoma-prone families in Sweden in the years 2015-2020: implications for novel national recommendations. Acta Oncologica, 2021; 60(7): 888. [ref. 1]
- Guidelines for reporting secondary findings of genome sequencing in cancer genes: the SFMPP recommendations. Eur J Hum Genet, 2018; 26(12): 1732. [ref. 2]
- 厚生労働省健康局がん・疾病対策課. 平成30年全国がん登録 罹患数・率報告 2018 [ref. 3]
- ClinGen Actionability Reports: CDKN2A Adult. ver.1.2.1 (2020.8.19) [ref. 4]
- 日本皮膚科学会. 皮膚悪性腫瘍ガイドライン第 3 版 メラノーマ診療ガイドライン 2019. 日皮会誌, 2019; 129(9):1759. [ref. 5]

supervising editor： Research Group for the Research Project on Ethical, Legal, and Social Issues Supported by the Health, Labour and Welfare Sciences Research Grants “Extraction of ethical and social issues and improvement of social environment toward the realization of a society where people can benefit from genome medicine without anxiety,” Actionability Working Group-Japan

Edited by MONSTAR-SCREEN-2　Medical Genetic Office

***TERT***

**If a germline genetic change was detected in this gene, the variant interpretation of its pathogenicity should be discussed case-by-case. Please consult the research office first.**

**Genetic Change and Hereditary Cancer Syndrome**

- - If genetic changes at birth are responsible for the hereditary cancer syndrome, the patient is considered to be predisposed to cancer.
  - In general, 5-10% of cancers are caused by genetic changes at birth.
  - Cancer susceptibility may be shared by relatives such as parents, children, siblings.
  - Understanding genetic risks allows better health management.
  - A blood test confirms whether the changes observed in genomic testing (tumor profiling) are hereditary.

**What Is *TERT* Related To?**

- A genetic change in the *TERT* gene causes Dyskeratosis Congenita (DC)^1,2)^.
- Dyskeratosis congenita is a congenital bone marrow failure syndrome characterized by nail dystrophy, oral leukoplakia, and skin pigmentation.
- The incidence of malignancies in patient with DC is reportedly 11 times higher than that in healthy individuals, including squamous cell carcinoma of the lungs and head and neck, adenocarcinoma of the gastrointestinal tract, myelodysplastic syndrome, and myeloid leukemia.

**What Is Genetic Counseling?**

Genetic Counseling provides information on how genetic conditions may affect you and/or your family. Please feel free to contact your genetic specialist if you have any relevant questions.

【参考文献】

- 厚生労働科学研究費補助金　難治性疾患等政策研究事業　特発性造血障害に関する調査研究班. 先天性角化不全症診療の参照ガイド 令和 1 年改訂版. (2019年12月) [ref. 1]
- GeneReviews®: Dyskeratosis Congenita. ver.2019.11.21 [ref. 2]

supervising editor： Research Group for the Research Project on Ethical, Legal, and Social Issues Supported by the Health, Labour and Welfare Sciences Research Grants “Extraction of ethical and social issues and improvement of social environment toward the realization of a society where people can benefit from genome medicine without anxiety,” Actionability Working Group-Japan

Edited by MONSTAR-SCREEN-2　Medical Genetic Office

***TGFBR1***

**What Is *TGFBR1* Related To?**

- - *TGFBR1* is a gene that have been shown to be associated with risk of cardiac disease.
  - A genetic change in the *TGFBR1* gene causes Loeys-Dietz Syndrome. (see Table 1)
  - A blood test will confirm whether the changes found in the genetic testing (tumor profiling) are hereditary.

**The Benefit of Confirmatory Testing**

- It is important to know the risk of cardiovascular disease for early detection.


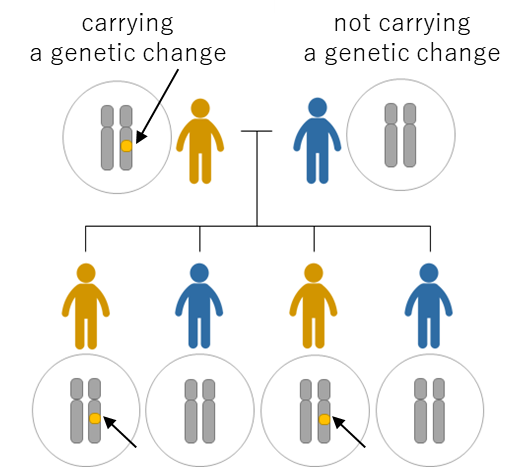
 **Risk of Family Members**

- There are two copies of *TGFBR1*. One and another copy is inherited from the mother, and the other from the father, respectively.
- Each child, brother, sister, and parent have a 50% chance of inheriting genetic changes in *TGFBR1*. Genetic testing can be performed to examine if similar changes are exhibited.
- Aunts, uncles, and cousins may share genetic changes and risks.
- Sharing your genetic information with your relatives may help them manage their health.

※ There is an approximately 75%^2)^ probability that individuals diagnosed with Loeys-Dietz Syndrome did not inherit it. This indicates that the genetic change appears in a family for the first time without the exact genetic change in the parents. Please consult your genetic specialist for information about the impact of such a case on your family.

**What Is Genetic Counseling?**

Genetic Counseling provides information on how genetic conditions may affect you and/or your family　and how to manage it. Please feel free to contact your genetic specialist if you have any relevant questions. ※Genetic testing(s) are not always covered by public health insurance in Japan. Genetic testing and follow-up care for healthy relatives who are unaffected by cancer are not covered by insurance. Please ask Genetic Services for details.

Table 1. Lifetime Risk and Recommended Management

|  | Japanese | Carrier of *TGFBR1* pathogenic / likely pathogenic variant | |
| --- | --- | --- | --- |
|  | Lifetime risk^1)^ | Lifetime risk^2)^ | Recommended management^1)^ |
| Aortic Aneurysm/ Dissection | 3-10 in 100,000 people/year | 95% | ・Magnetic resonance angiography (MRA) or CT angiogram (CTA)  ・If aortic root dilatation is seen, consider aortic root replacement  ・Beta-adrenergic blockers or angiotensin receptor blockers: treatment for hypertension |

※Other characteristic skeletal and/or skin symptoms may occur in association with the genetic change in *TGFBR1* gene.

※It is important to note that not all carriers who inherit genetic changes in *TGFBR1* be symptomatic.

※The likelihood of being symptomatic if carrying a genetic change and recommended managements are based on the Japanese guideline^1)^. Please contact specialists and Genetic Services for details.

【References】

- 日本循環器学会・日本心臓血管外科学会・日本胸部外科学会・日本血管外科学会合同ガイドライン. 大動脈瘤・大動脈解離診療ガイドライン 2020年改訂版. (2020年7月) [ref. 1]
- GeneReviews Japan: ロイス・ディーツ症候群. ver.2021.4.30 [ref. 2]
- ClinGen Actionability Reports: SMAD3, TGFB2, TGFB3, TGFBR1, TGFBR2 Adult. ver.3.0.4 (2021.8.24)

supervising editor： Research Group for the Research Project on Ethical, Legal, and Social Issues Supported by the Health, Labour and Welfare Sciences Research Grants “Extraction of ethical and social issues and improvement of social environment toward the realization of a society where people can benefit from genome medicine without anxiety,” Actionability Working Group-Japan

Edited by MONSTAR-SCREEN-2　Medical Genetic Office

***TGFBR2***

**What Is *TGFBR2* Related To?**

- - *TGFBR2* is a gene that has been shown to be associated with risk of cardiac disease.
  - A genetic change in the *TGFBR2* gene causes Loeys-Dietz Syndrome. (see Table 1)
  - A blood test will confirm whether the changes found in the genetic testing (tumor profiling) are hereditary.

**The Benefit of Confirmatory Testing**

- It is important to know the risk of cardiovascular disease for early detection.


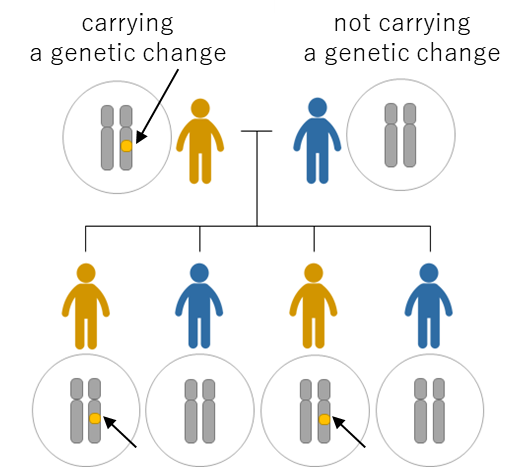
 **Risk of Family Members**

- There are two copies of *TGFBR2*. One and another copy is inherited from the mother, and the other from the father, respectively.
- Each child, brother, sister, and parent have a 50% chance of inheriting genetic changes in *TGFBR2*. Genetic testing can be performed to examine if similar changes are exhibited.
- Aunts, uncles, and cousins may share genetic changes and risks.
- Sharing your genetic information with your relatives may help them manage their health.

※ There is an approximately 75%^2)^ probability that individuals diagnosed with Loeys-Dietz Syndrome did not inherit it. This indicates that the genetic change appears in a family for the first time without the exact genetic change in the parents. Please consult your genetic specialist for information about the impact of such a case on your family.

**What Is Genetic Counseling?**

Genetic Counseling provides information on how genetic conditions may affect you and/or your family　and how to manage it. Please feel free to contact your genetic specialist if you have any relevant questions. ※Genetic testing(s) are not always covered by public health insurance in Japan. Genetic testing and follow-up care for healthy relatives who are unaffected by cancer are not covered by insurance. Please ask Genetic Services for details.

Table 1. Lifetime Risk and Recommended Management

|  | Japanese | Carrier of *TGFBR2* pathogenic / likely pathogenic variant | |
| --- | --- | --- | --- |
|  | Lifetime risk^1)^ | Lifetime risk^2)^ | Recommended management^1)^ |
| Aortic Aneurysm/ Dissection | 3-10 in 100,000 people/year | 95% | ・Magnetic resonance angiography (MRA) or CT angiogram (CTA)  ・If aortic root dilatation is seen, consider aortic root replacement  ・Beta-adrenergic blockers or angiotensin receptor blockers: treatment for hypertension |

※Other characteristic skeletal and/or skin symptoms may occur in association with the genetic change in *TGFBR2* gene.

※It is important to note that not all carriers who inherit genetic changes in *TGFBR2* be symptomatic.

※The likelihood of being symptomatic if carrying a genetic change and recommended management is based on the Japanese guideline^1)^. Please contact specialists and Genetic Services for further details.

【References】

- 日本循環器学会・日本心臓血管外科学会・日本胸部外科学会・日本血管外科学会合同ガイドライン. 大動脈瘤・大動脈解離診療ガイドライン 2020年改訂版. (2020年7月) [ref. 1]
- GeneReviews Japan: ロイス・ディーツ症候群. ver.2021.4.30 [ref. 2]
- ClinGen Actionability Reports: SMAD3, TGFB2, TGFB3, TGFBR1, TGFBR2 Adult. ver.3.0.4 (2021.8.24)

supervising editor： Research Group for the Research Project on Ethical, Legal, and Social Issues Supported by the Health, Labour and Welfare Sciences Research Grants “Extraction of ethical and social issues and improvement of social environment toward the realization of a society where people can benefit from genome medicine without anxiety,” Actionability Working Group-Japan

Edited by MONSTAR-SCREEN-2　Medical Genetic Office

***TMEM127***

**Genetic Change and Hereditary Cancer Syndrome**

- - If genetic changes at birth are responsible for the hereditary cancer syndrome, the patient is considered to be predisposed to cancer.
  - In general, 5-10% of cancers are caused by genetic changes at birth.
  - Cancer susceptibility may be shared by relatives such as parents, children, siblings.
  - Understanding genetic risks allows better health management.
  - A blood test confirms whether the changes observed in genomic testing (tumor profiling) are hereditary.

**What Is *TMEM127* Related To?**

- *TMEM127* has been shown to be associated with an increased risk of cancer (see Table 1).

**The Benefit of Confirmatory Testing**

- It is important to know the cancer risk for prevention and early detection of cancer.


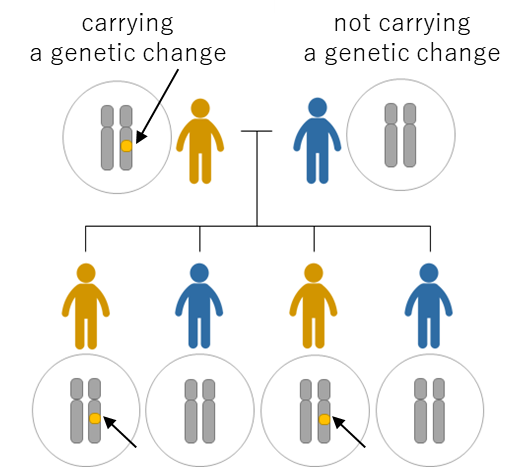
 **Risk of Family Members**

- There are two copies of *TMEM127*. One and another copy is inherited from the mother, and the other from the father, respectively.
- Each child, brother, sister, and parent have a 50% chance of inheriting genetic changes in *TMEM127*. Genetic testing can be performed to examine if similar changes are exhibited.
- Aunts, uncles, and cousins may share genetic changes and risks.
- Sharing your genetic information with your relatives may help them manage their health.

※ It is possible that the *TMEM127* gene change is not inherited from either parent but is a *de* *novo* change. Please consult your genetic specialist for information about the impact of such a case on your family.

**What Is Genetic Counseling?**

Genetic Counseling provides information on how genetic conditions may affect you and/or your family　and how to manage it. Please feel free to contact your genetic specialist if you have any relevant questions. ※Genetic testing(s) are not always covered by public health insurance in Japan. Genetic testing and follow-up care for healthy relatives who are unaffected by cancer are not covered by insurance. Please ask Genetic Services for details.

Table 1. Lifetime Risk and Recommended Management

|  | Japanese | Carrier of *TMEM127* pathogenic / likely pathogenic variant | | | |
| --- | --- | --- | --- | --- | --- |
|  | Lifetime risk ^1,2,3)^ | Lifetime risk | | Recommended management | |
|  |  | Women^3)^ | Men^3)^ | Women^3)^ | Men^3)^ |
| Paraganglioma | Japan: 1,500 patients/year  (USA: 2 in 1000,000 people) | Potential for increased risk | | ・Annual Blood test (Plasma-free metanephrines)  ・CT or MRI every 2 years  ・I-MIBG scintigraphy every 3 years | |
| Adrenal pheochromocytoma | 0.01-0.02 ％ | Potential for increased risk | |  |  |

※It is important to note that not all carriers who inherit genetic changes in *TMEM127* are symptomatic.

※The probabilities shown in Table 1 include benign, borderline malignant, and malignant tumors.

※The likelihood of being symptomatic if carrying a genetic change and recommended management is based on the Japanese guideline^3)^. Please contact specialists and Genetic Services if management is provided at the hospital.

※Table1 is based on information as of 2021. Recommendations may be revised as research progresses.

【References】

- 国立がん研究センター希少がんセンター: パラガングリオーマ <https://www.ncc.go.jp/jp/rcc/about/paraganguriouma/index.html> [ref. 1]
- National Cancer Institute Center for Cancer Research <https://www.cancer.gov/pediatric-adult-rare-tumor/rare-tumors/rare-endocrine-tumor/paraganglioma> [ref. 2]
- 日本内分泌学会. 褐色細胞腫・パラガングリオーマ診療ガイドライン2018. (2018年7月) [ref. 3]
- GeneReviews Japan: 遺伝性パラガングリオーマ・褐色細胞腫症候群. ver.2020.7.15
- ClinGen Actionability Reports: MAX, SDHA, SDHAF2, SDHB, SDHC, SDHD, TMEM127 Adult. ver.1.1.3 (2022.1.3)

supervising editor： Research Group for the Research Project on Ethical, Legal, and Social Issues Supported by the Health, Labour and Welfare Sciences Research Grants “Extraction of ethical and social issues and improvement of social environment toward the realization of a society where people can benefit from genome medicine without anxiety,” Actionability Working Group-Japan

Edited by MONSTAR-SCREEN-2　Medical Genetic Office

***TP53***

**Genetic Change and Hereditary Cancer Syndrome**

- - If genetic changes at birth are responsible for the hereditary cancer syndrome, the patient is considered to be predisposed to cancer.
  - In general, 5-10% of cancers are caused by genetic changes at birth.
  - Cancer susceptibility may be shared by relatives such as parents, children, siblings.
  - Understanding genetic risks allows better health management.
  - A blood test confirms whether the changes observed in genomic testing (tumor profiling) are hereditary.

**What Is *TP53* Related To?**

- Genetic changes in the *TP53* gene causes Li-Fraumeni Syndrome (see Table 1).

**The Benefit of Confirmatory Testing**

- It is important to know the cancer risk for prevention and early detection of cancer.


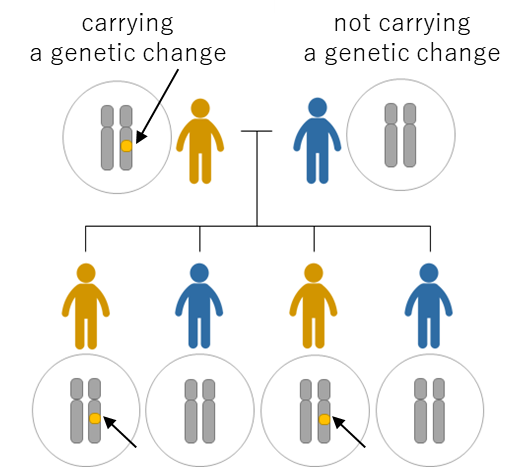
 **Risk of Family Members**

- There are two copies of *TP53*. One and another copy is inherited from the mother, and the other from the father, respectively.
- Each child, brother, sister, and parent have a 50% chance of inheriting genetic changes in *TP53*. Genetic testing can be performed to examine if similar changes are exhibited.
- Aunts, uncles, and cousins may share genetic changes and risks.
- Sharing your genetic information with your relatives may help them manage their health.

※ There is an approximately 25%^1)^ probability that individuals diagnosed with Li-Fraumeni Syndrome did not inherit it. This indicates that the genetic change appears in a family for the first time without the exact genetic change in the parents. Please consult your genetic specialist for information about the impact of such a case on your family.

**What Is Genetic Counseling?**

Genetic Counseling provides information on how genetic conditions may affect you and/or your family　and how to manage it. Please feel free to contact your genetic specialist if you have any relevant questions. ※Genetic testing(s) are not always covered by public health insurance in Japan. Genetic testing and follow-up care for healthy relatives who are unaffected by cancer are not covered by insurance. Please ask Genetic Services for details.

Table 1. Lifetime Risk and Recommended Management

|  | Japanese | Carrier of *TP53* pathogenic / likely pathogenic variant | | | |
| --- | --- | --- | --- | --- | --- |
|  | Lifetime risk^2,3,4,5)^ | Lifetime risk^1)^ | | Recommended management for adult ^1)^ | |
|  |  | Women | Men | Women | Men |
| Breast Cancer | 10.9 ％ (Women) | 25.0-59.6 ％ | ― | ・Starting at age 18y: breast awareness  ・Starting at age 20y: clinical breast exam every 6 months  ・Age 20-75y: annual breast MRI screening with contrast  ・Consider risk-reducing mastectomy | ― |
| Osteosarcoma | 500-800 patients/year | 6.3-15.5 % | | ・Annual whole-body MRI  ・Annual abdominopelvic ultrasound | |
| Soft Tissue Sarcoma | 1500 patients/year | 14.3-26.7 ％ | |  |  |
| Brain Tumor | 0.3 % (Men)  0.2 % (Women) | 5.4-13.0 % | | ・Annual brain MRI | |
| Adrenocortical Cancer | (USA: Approximately 0.72 in 1,000,000 people) | 1.7-13.0 % | | ・Currently, there is no established management. | |

※In addition to the symptoms listed in Table 1, regular screening of the gastrointestinal tract, skin, and systemic examination is recommended. Additional management is recommended for children (between birth to 18y). Radiation therapy, sun exposure, and smoking should be avoided to reduce the risk of developing cancer (including secondary cancers).^1,6,7)^

※It is important to note that not all carriers who inherit genetic changes in the *TP53* develop cancer. However, the lifetime risk of developing any types of cancer is nearly 100% in women and approximately 75% in men ^1)^.

※Table 1 is based on the Japanese guideline^1)^. Please contact specialists and Genetic Services for further details.

※Table1 is based on information as of 2021. Recommendations may be revised as research progresses.

【References】

- 厚⽣労働省科学研究費補助⾦（がん政策研究事業）⼩児期に発症する遺伝性腫瘍に対するがんゲノム医療体制実装のための研究. リー・フラウメニ症候群の診療ガイドライン 2019 年度版. ver.1.1 (2020年3月) [ref. 1]
- 国立がん研究センターがん情報サービス 「累積がん罹患リスク(2018年データ)」 <https://ganjoho.jp/reg_stat/statistics/stat/summary.html> [ref. 2]
- 国立がん研究センター希少がんセンター: 骨の肉腫 https://www.ncc.go.jp/jp/rcc/about/bone_sarcomas/index.html [ref. 3]
- 厚生労働省健康局がん・疾病対策課. 平成30年全国がん登録 罹患数・率報告 2018 [ref. 4]
- Adrenocortical carcinoma in the United States: treatment utilization and prognostic factors. Cancer, 2008; 113(11): 3130. [ref. 5]
- NCCN Guidelines® Genetic/Familial High-Risk Assessment: Breast, Ovarian, and Pancreatic. ver.1.2022 (2021.8.11) [ref. 6]
- ClinGen Actionability Reports: TP53 Adult. ver.3.0.0 (2021.7.21) [ref. 7]
- GeneReviews Japan: リ・フラウメニ症候群. ver.2020.3.6

supervising editor： Research Group for the Research Project on Ethical, Legal, and Social Issues Supported by the Health, Labour and Welfare Sciences Research Grants “Extraction of ethical and social issues and improvement of social environment toward the realization of a society where people can benefit from genome medicine without anxiety,” Actionability Working Group-Japan

Edited by MONSTAR-SCREEN-2　Medical Genetic Office

***TSC1***

**Genetic Change and Hereditary Cancer Syndrome**

- - If genetic changes at birth are responsible for the hereditary cancer syndrome, the patient is considered to be predisposed to cancer.
  - In general, 5-10% of cancers are caused by genetic changes at birth.
  - Cancer susceptibility may be shared by relatives such as parents, children, siblings.
  - Understanding genetic risks allows better health management.
  - A blood test confirms whether the changes observed in genomic testing (tumor profiling) are hereditary.

**What Is *TSC1* Related To?**

- Genetic changes in the *TSC1* gene causes Tuberous Sclerosis Complex (see Table 1).

**The Benefit of Confirmatory Testing**

- It is important to know the cancer risk for prevention and early detection of cancer.


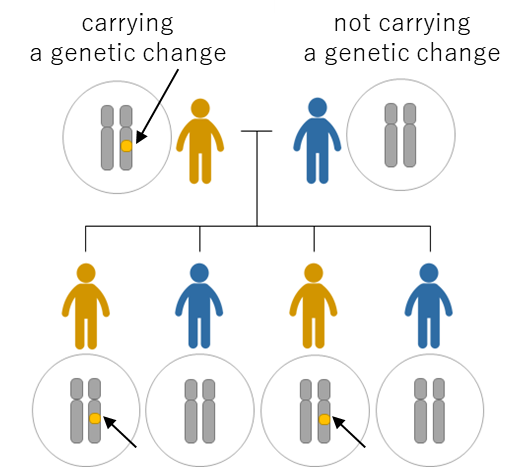
 **Risk of Family Members**

- There are two copies of *TSC1*. One and another copy is inherited from the mother, and the other from the father, respectively.
- Each child, brother, sister, and parent have a 50% chance of inheriting genetic changes in *TSC1*. Genetic testing can be performed to examine if similar changes are exhibited.
- Aunts, uncles, and cousins may share genetic changes and risks.
- Sharing your genetic information with your relatives may help them manage their health.

※ There is an approximately 60%^1,2,3)^ probability that individuals diagnosed with Tuberous sclerosis complex did not inherit it. This indicates that the genetic change appears in a family for the first time without the exact genetic change in the parents. Please consult your genetic specialist for information about the impact of such a case on your family.

**What Is Genetic Counseling?**

Genetic Counseling provides information on how genetic conditions may affect you and/or your family　and how to manage it. Please feel free to contact your genetic specialist if you have any relevant questions. ※Genetic testing(s) are not always covered by public health insurance in Japan. Genetic testing and follow-up care for healthy relatives who are unaffected by cancer are not covered by insurance. Please ask Genetic Services for details.

Table 1. Lifetime Risk and Recommended Management

|  | Japanese | Carrier of *TSC1* pathogenic / likely pathogenic variant | | | |
| --- | --- | --- | --- | --- | --- |
|  | Lifetime risk^4,5,6)^ | Lifetime risk | | Recommend management | |
|  |  | Women^1,2)^ | Men^1,2)^ | Women^1,2,7)^ | Men^1,2,7)^ |
| Facial Angiofibroma | No data available | 80% over 5y | | ・Surgical approaches: excision or lasers  ・mTORC1i: oral medication or ointment | |
| Rhabdomyoma | No data available | 47-67 % | | ・Echocardiology and electrocardiography every 1-3 years | |
| Epilepsy | 5-8 in 1,000 people | 84 % | | ・Patients predicted to develop epilepsy in infancy: consider electroencephalography up to 1 month  ・Pediatric patients: electroencephalography should be performed with or without epilepsy  ・For infantile spasms: vigabatrin  ・Other symptomatic treatments | |
| Subependymal Giant Cell Astrocytoma (SEGA) | No data available | 5-15 % | | Brain MRI or CT every 2-3 years | |
| Lymphangioleiomyomatosis　(LAM) | 1.9-4.5 in 1,000,000 people | 30-40 % | 10-12 % | ・Spirometry every 5-10 years  ・6-minute walk test, high-resolution CT | |
| Renal Cell Carcinoma | 10.1 (Men)  /3.7 (Women)  in 100,000 people | 2-4 % | | ・MRI or abdominal ultrasound every 1-2 years | |

※In addition to the above symptoms, other symptoms may be seen in the brain, heart, lungs, skin, and teeth. Please contact specialists and Genetic Services for details.

※It is important to note that not all carriers who inherit genetic changes in *TSC1* be symptomatic.

※The probabilities shown in Table 1 include benign, borderline malignant, and malignant tumors.

※The likelihood of being symptomatic if carrying a genetic change and recommended management is based on the Japanese guideline^1, 7)^ Please contact specialists and Genetic Services if management is provided at the hospital.

※Table1 is based on information as of 2021. Recommendations may be revised as research progresses.

【References】

- 日本皮膚科学会. 結節性硬化症の診断基準及び治療ガイドライン 改訂版. 日皮会誌, 2018; 128(1): 1. [ref. 1]
- GeneReviews Japan: 結節性硬化症. ver.2020.9.29 [ref. 2]
- ClinGen Actionability Reports: TSC1, TSC2 Adult. ver.1.1.2 (2020.4.29) [ref. 3]
- 厚生労働省 てんかん対策 <https://www.mhlw.go.jp/stf/seisakunitsuite/bunya/0000070789_00008.html> [ref. 4]
- 林ら. 肺リンパ脈管筋腫症に関する全国疫学調査 追跡調査および第2回目全国横断調査．厚生労働省難治性疾患呼吸不全に関する調査研究 平成19年度総括・分担研究報告書．2008 [ref. 5]
- 国立がん研究センターがん情報サービス「がん統計」(全国がん登録) 2016年～2018年 [ref. 6]
- 日本脳腫瘍学会. 脳腫瘍診療ガイドライン 2019年版 小児脳腫瘍編 上衣下巨細胞性星細胞腫 (SEGA) 診療ガイドライン. (2019年5月) [ref. 7]

supervising editor： Research Group for the Research Project on Ethical, Legal, and Social Issues Supported by the Health, Labour and Welfare Sciences Research Grants “Extraction of ethical and social issues and improvement of social environment toward the realization of a society where people can benefit from genome medicine without anxiety,” Actionability Working Group-Japan

Edited by MONSTAR-SCREEN-2　Medical Genetic Office

***TSC2***

**Genetic Change and Hereditary Cancer Syndrome**

- - If genetic changes at birth are responsible for the hereditary cancer syndrome, the patient is considered to be predisposed to cancer.
  - In general, 5-10% of cancers are caused by genetic changes at birth.
  - Cancer susceptibility may be shared by relatives such as parents, children, siblings.
  - Understanding genetic risks allows better health management.
  - A blood test confirms whether the changes observed in genomic testing (tumor profiling) are hereditary.

**What Is *TSC2* Related To?**

- Genetic changes in the *TSC2* gene causes Tuberous Sclerosis Complex (see Table 1).

**The Benefit of Confirmatory Testing**

- It is important to know the cancer risk for prevention and early detection of cancer.


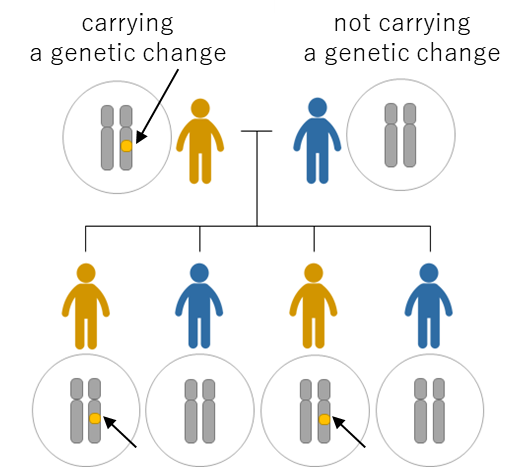
 **Risk of Family Members**

- There are two copies of *TSC2*. One and another copy is inherited from the mother, and the other from the father, respectively.
- Each child, brother, sister, and parent have a 50% chance of inheriting genetic changes in *TSC2*. Genetic testing can be performed to examine if similar changes are exhibited.
- Aunts, uncles, and cousins may share genetic changes and risks.
- Sharing your genetic information with your relatives may help them manage their health.

※ There is an approximately 60%^1,2,3)^ probability that individuals diagnosed with Tuberous sclerosis complex did not inherit it. This indicates that the genetic change appears in a family for the first time without the exact genetic change in the parents. Please consult your genetic specialist for information sbout the impact of such a case on your family.

**What Is Genetic Counseling?**

Genetic Counseling provides information on how genetic conditions may affect you and/or your family　and how to manage it. Please feel free to contact your genetic specialist if you have any relevant questions. ※Genetic testing(s) are not always covered by public health insurance in Japan. Genetic testing and follow-up care for healthy relatives who are unaffected by cancer are not covered by insurance. Please ask Genetic Services for details.

Table 1. Lifetime Risk and Recommended Management

|  | Japanese | Carrier of *TSC2* pathogenic / likely pathogenic variant | | | |
| --- | --- | --- | --- | --- | --- |
|  | Lifetime risk^4,5,6)^ | Lifetime risk | | Recommend management | |
|  |  | Women^1,2)^ | Men^1,2)^ | Women^1,2,7)^ | Men^1,2,7)^ |
| Facial Angiofibroma | No data available | 80% over 5 years old | | ・Surgical approaches: excision or lasers  ・mTORC1i: oral medication or ointment | |
| Rhabdomyoma | No data available | 47-67 % | | ・Echocardiology and electrocardiography every 1-3 years | |
| Epilepsy | 5-8 in 1,000 people | 84 % | | ・Patients predicted to develop epilepsy in infancy: consider electroencephalography up to 1 month  ・Pediatric patients: electroencephalography should be performed with or without epilepsy  ・For infantile spasms: vigabatrin  ・Other symptomatic treatments | |
| Subependymal Giant Cell Astrocytoma (SEGA) | No data available | 5-15 % | | Brain MRI or CT every 2-3 years | |
| Lymphangioleiomyomatosis　(LAM) | 1.9-4.5 in 1,000,000 people | 30-40 % | 10-12 % | ・Spirometry every 5-10 years  ・6-minute walk test, high-resolution CT | |
| Renal Cell Carcinoma | 10.1 (Men)  3.7 (Women)  in 100,000 people | 2-4 % | | ・MRI or abdominal ultrasound every 1-2 years | |

※In addition to the above symptoms, other symptoms may be seen in the brain, heart, lungs, skin, and teeth. Please contact specialists and Genetic Services for details.

※It is important to note that not all carriers who inherit genetic changes in *TSC1* be symptomatic.

※The probabilities shown in the Table 1 include benign, borderline malignant, and malignant tumors.

※The likelihood of being symptomatic if carrying a genetic change and recommended management is based on the Japanese guideline^1, 7)^ Please contact specialists or Genetic Services if management is provided at the hospital.

※Table1 is based on information as of 2021. Recommendations may be revised as research progresses.

【References】

- 日本皮膚科学会. 結節性硬化症の診断基準及び治療ガイドライン 改訂版. 日皮会誌, 2018; 128(1): 1. [ref. 1]
- GeneReviews Japan: 結節性硬化症. ver.2020.9.29 [ref. 2]
- ClinGen Actionability Reports: TSC1, TSC2 Adult. ver.1.1.2 (2020.4.29) [ref. 3]
- 厚生労働省 てんかん対策 <https://www.mhlw.go.jp/stf/seisakunitsuite/bunya/0000070789_00008.html> [ref. 4]
- 林ら. 肺リンパ脈管筋腫症に関する全国疫学調査 追跡調査および第2回目全国横断調査．厚生労働省難治性疾患呼吸不全に関する調査研究 平成19年度総括・分担研究報告書．2008 [ref. 5]
- 国立がん研究センターがん情報サービス「がん統計」(全国がん登録) 2016年～2018年 [ref. 6]
- 日本脳腫瘍学会. 脳腫瘍診療ガイドライン 2019年版 小児脳腫瘍編 上衣下巨細胞性星細胞腫 (SEGA) 診療ガイドライン. (2019年5月) [ref. 7]

supervising editor： Research Group for the Research Project on Ethical, Legal, and Social Issues Supported by the Health, Labour and Welfare Sciences Research Grants “Extraction of ethical and social issues and improvement of social environment toward the realization of a society where people can benefit from genome medicine without anxiety,” Actionability Working Group-Japan

Edited by MONSTAR-SCREEN-2　Medical Genetic Office

***VHL***

**Genetic Change and Hereditary Cancer Syndrome**

- - If genetic changes at birth are responsible for the hereditary cancer syndrome, the patient is considered to be predisposed to cancer.
  - In general, 5-10% of cancers are caused by genetic changes at birth.
  - Cancer susceptibility may be shared by relatives such as parents, children, siblings.
  - Understanding genetic risks allows better health management.
  - A blood test confirms whether the changes observed in genomic testing (tumor profiling) are hereditary.

**What Is *VHL* Related To?**

- Genetic changes in the *VHL* gene causes Von Hippel-Lindau Syndrome (see Table 1).

**The Benefit of Confirmatory Testing**

- It is important to know the cancer risk for prevention and early detection of cancer.


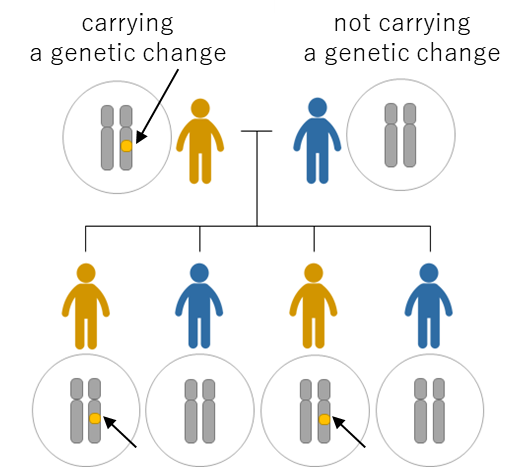
 **Risk of Family Members**

- There are two copies of *VHL*. One and another copy is inherited from the mother, and the other from the father, respectively.
- Each child, brother, sister, and parent have a 50% chance of inheriting genetic changes in *VHL*. Genetic testing can be performed to examine if similar changes are exhibited.
- Aunts, uncles, and cousins may share genetic changes and risks.
- Sharing your genetic information with your relatives may help them manage their health.

※ There is an approximately 20%^1)^ probability that individuals diagnosed with Von Hippel-Lindau Syndrome did not inherit it. This indicates that the genetic change appears in a family for the first time without the exact genetic change in the parents. Please consult your genetic specialist for information about the impact of such a case on your family.

**What Is Genetic Counseling?**

Genetic Counseling provides information on how genetic conditions may affect you and/or your family　and how to manage it. Please feel free to contact your genetic specialist if you have any relevant questions. ※Genetic testing(s) are not always covered by public health insurance in Japan. Genetic testing and follow-up care for healthy relatives who are unaffected by cancer are not covered by insurance. Please ask Genetic Services for details.

Table 1. Lifetime Risk and Recommended Management

|  | Japanese | Carrier of *VHL* pathogenic / likely pathogenic variant | | | |
| --- | --- | --- | --- | --- | --- |
|  | Lifetime risk^,3,4,5,6)^ | Lifetime risk | | Recommended management | |
|  |  | Women^7)^ | Men^7)^ | Women^7)^ | Men^7)^ |
| Central Nervous System (CNS) Hemangioblastoma | 5-6 in 10,000,000 people | 60-80 ％ | | ・MRI with contrast every 2 years | |
| Retinal Hemangioblastoma | No data available | 40-70 ％ | | ・Ophthalmoscopy every 3 years | |
| Pancreatic Cyst | 0.21-13.5% | 17-61 ％ | | ・Follow-up or treatment is not required until clinical symptoms, such as compression of other organs by enlarged cyst, occur. | |
| Renal Cyst | No data available | 60-80 ％ | | ・Follow-up is recommended regardless of cyst size | |
| Renal Cell Carcinoma | 10.1 (Men)  3.7 (Women)  in 100,000 people | 25-50 % | | ・Examination every other year Mutual ultrasound and non-contrast MRI scans on an annual basis | |
| Gastroenteropancreatic Neuroendocrine Neoplasm | 2-3 in 100,000 people | 8-17 ％ | | ・Examination and CT every 2-3 years  ・If no pancreatic neuroendocrine tumors are detected, perform abdominal ultrasound and non-contrast abdominal MRI once a year | |
| Adrenal Pheochromocytoma | 0.01-0.02 ％ | 10-20 % | | ・Pooled urine test (metanephrine, normetanephrine, adrenaline, norepinephrine) and blood test (catecholamine)  ・Imaging medical examination | |

※In addition to the symptoms listed in Table 1, other testis, uterus, inner ear-related, etc., characteristics may be detected. Please contact specialists and Genetic Services for details.

※It is important to note that not all carriers who inherit genetic changes in *VHL* develop cancer.

※The probabilities shown in Table 1 include benign, borderline malignant, and malignant tumors.

※The likelihood of being symptomatic if carrying a genetic change and recommended management is based on the Japanese guideline^7)^. Please contact specialists and Genetic Services if management is provided at the hospital.

※Table1 is based on information as of 2021. Recommendations may be revised as research progresses.

【References】

- GeneReviews Japan: フォンヒッペル・リンドウ病. ver.2018.8.25 [ref. 1]
- 全国がん登録罹患数・率 報告 平成30年 <https://www.mhlw.go.jp/content/10900000/000794199.pdf> [ref. 2]
- 中井. 膵嚢胞性腫瘍ガイドラインをめぐって〜超音波内視鏡診断・治療の役割〜. 埼玉医科大学雑誌, 2017; 43(2): 129. [ref. 3]
- 国立がん研究センターがん情報サービス「がん統計」(全国がん登録) 2016年～2018年 [ref. 4]
- 日本神経内分泌腫瘍研究会. 膵・消化管神経内分泌腫瘍診療ガイドライン第2版作成委員会. 膵・消化管神経内分泌腫瘍 (NEN) 診療ガイドライン 2019年第2版. (2019年9月) [ref. 5]
- 日本内分泌学会. 褐色細胞腫・パラガングリオーマ診療ガイドライン2018. (2018年7月) [ref. 6]
- 「多彩な内分泌異常を生じる遺伝性疾患(多発性内分泌腫瘍症およびフォン・ヒッペル・リンドウ病)の実態把握と診療標準化の研究」班. フォン・ヒッペル・リンドウ (VHL) 病 診療ガイドライン2017年版. (2017年) [ref. 7]
- ClinGen Actionability Reports: VHL Adult. ver.1.2.1 (2020.12.23) [ref. 8]

supervising editor： Research Group for the Research Project on Ethical, Legal, and Social Issues Supported by the Health, Labour and Welfare Sciences Research Grants “Extraction of ethical and social issues and improvement of social environment toward the realization of a society where people can benefit from genome medicine without anxiety,” Actionability Working Group-Japan

Edited by MONSTAR-SCREEN-2　Medical Genetic Office

***WT1***

**Genetic Change and Hereditary Cancer Syndrome**

- - If genetic changes at birth are responsible for the hereditary cancer syndrome, the patient is considered to be predisposed to cancer.
  - In general, 5-10% of cancers are caused by genetic changes at birth.
  - Cancer susceptibility may be shared by relatives such as parents, children, siblings.
  - Understanding genetic risks allows better health management.
  - A blood test confirms whether the changes observed in genomic testing (tumor profiling) are hereditary.

**What Is *WT1* Related To?**

- Genetic changes in the *WT1* gene causes WT1-Related Wilms Tumor Syndrome (see Table 1).

**The Benefit of Confirmatory Testing**

- It is important to know the cancer risk for prevention and early detection of cancer.


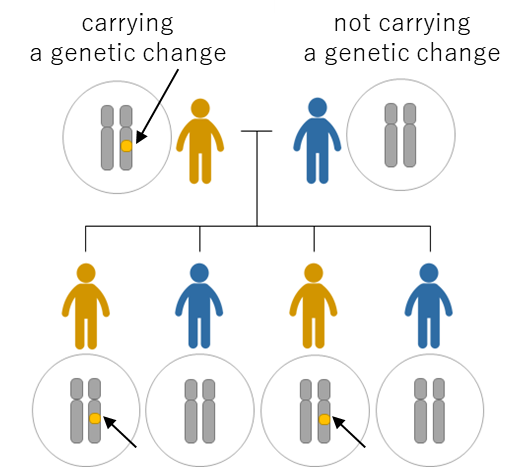
 **Risk of Family Members**

- There are two copies of *WT1*. One and another copy is inherited from the mother, and the other from the father, respectively.
- Each child, brother, sister, and parent have a 50% chance of inheriting genetic changes in *WT1*. Genetic testing can be performed to examine if similar changes are exhibited.
- Aunts, uncles, and cousins may share genetic changes and risks.
- Sharing your genetic information with your relatives may help them manage their health.

※ It is possible that the *WT1* gene change is not inherited from either parent but is a *de* *novo* change. Please consult your genetic specialist for information about the impact of such a case on your family.

**What Is Genetic Counseling?**

Genetic Counseling provides information on how genetic conditions may affect you and/or your family　and how to manage it. Please feel free to contact your genetic specialist if you have any relevant questions. ※Genetic testing(s) are not always covered by public health insurance in Japan. Genetic testing and follow-up care for healthy relatives who are unaffected by cancer are not covered by insurance. Please ask Genetic Services for details.

Table 1. Lifetime Risk and Recommended Management

|  | Japanese | Carrier of *WT1* pathogenic / likely pathogenic variant | | | |
| --- | --- | --- | --- | --- | --- |
|  | Lifetime risk^1)^ | Lifetime risk^2,3)^ | | Recommended management^4)^ | |
|  |  | Women | Men | Women | Men |
| Wilms Tumor (Nephroblastoma) | No data available  70-100 cases/year | 38 %  Onset of most cases is approximately 3 y of age | | Routine abdominal ultrasound* | |

* The recommended management in Table 1 is not specifically presented in Japan. Please contact the genetic specialist at your institution for further details.

※The likelihood of being symptomatic if carrying a genetic change and recommended management is based on the international data^2,3,4)^.

※Table1 is based on information as of 2021. Recommendations may be revised as research progresses.

【References】

- 国立がん研究センターがん情報サービス 「累積がん罹患リスク (2018年データ)」 <https://ganjoho.jp/reg_stat/statistics/stat/summary.html> [ref. 1]
- Genotype-phenotype associations in WT1 glomerulopathy. Kidney Int, 2014; 85(5): 1169. [ref. 2]
- ClinGen Actionability Reports: WT1 Adult. ver.1.0.0 (2021.10.18) [ref. 3]
- GeneReviews Japan: ウィルムス腫瘍易罹患性. ver.2018.8.22 [ref. 4]

supervising editor： Research Group for the Research Project on Ethical, Legal, and Social Issues Supported by the Health, Labour and Welfare Sciences Research Grants “Extraction of ethical and social issues and improvement of social environment toward the realization of a society where people can benefit from genome medicine without anxiety,” Actionability Working Group-Japan

Edited by MONSTAR-SCREEN-2　Medical Genetic Office
