# Supplementary material for: Establishment of a comprehensive set of fact sheets for cancer predisposition genes for medical oncologists practicing cancer genome profiling
Source: Int J Clin Oncol. 2025 Apr 4;30(5):827–36. doi: 10.1007/s10147-025-02746-w (PMC12014809; doi:10.1007/s10147-025-02746-w)
Supplement: Supplementary file 3 — Supplementary file3 (DOCX 26 KB) [file 10147_2025_2746_MOESM3_ESM.docx]

**MONSTAR-SCREEN-2 Fact Sheet Questionnaire**

The MONSTAR-SCREEN-2 Fact Sheet was developed as an educational tool for medical oncologists under the supervision of the SCRUM-Japan Genetic Specialist Committee. Genes were selected based on the Comprehensive Tumor Genomics Profiling List of secondary findings to be disclosed to patients according to the level of recommendation (Ver3.1_20210815).

The results of this questionnaire will be used to revise the fact sheet and develop other educational tools. We would appreciate your cooperation in completing this survey.

 MONSTAR-SCREEN-2 genetic office

 ※About 5-10 minutes

If you do not have a full version of the fact sheet (55 genes), please visit the URL below and enter the access code for download.

URL: *MONSTAR-SCREEN-2 ファクトシート（PDF）*

Access code：XXXX

**1.** **Awareness and frequency of using the MONSTAR-SCREEN-2 Fact Sheet (Fact Sheet)**

(1) Did you know about the Fact Sheet?

□ Yes →Go to the next part

□ No →Go to (2)

(2) Have you ever used the Fact Sheet, including referring to it, before visiting your clinic?

□ Yes

□ No

□ Others:

**2. Overall Evaluation of the Fact Sheet**

(1) The Fact Sheet contains accurate and reliable information

□Strongly agree □Agree □Neutral □Disagree □Strongly disagree □Unsure

(2) The act sheet was useful and easy to use.

□Strongly agree □Agree □Neutral □Disagree □Strongly disagree □Unsure

(3) The Fact Sheet is a more educational tool than previous tools for gathering information about each gene and hereditary cancer.

□Strongly agree □Agree □Neither agree or disagree □Disagree □Strongly disagree

**3. Evaluation and Opinion on the Contents of the Fact Sheet**

(1) Layout

□Very Good □Good □Neutral □Poor □Very poor □Unsure

(2) This Fact Sheet was developed with the reference priority below, prioritizing Japanese guidelines by referring to Japanese data and medical guidance. Select the item that best reflects your opinion.

1. Japanese (domestic) guidelines

2. National Comprehensive Cancer Network (NCCN) guidelines

3. GeneReviews Japan, ClinGen Actionability Summary Report.

4. GeneReviews

□Strongly agree □Agree □Neither agree or disagree □Disagree □Strongly disagree □Unsure

(3) Referring to Japanese Guidelines, select the item that best describes your opinion.

□Strongly agree □Agree □Neutral □Disagree □Strongly disagree □Unsure

(4) Select the item that best describes your opinion referring to NCCN Guidelines.

□Strongly agree □Agree □Neutral □Disagree □Strongly disagree □Unsure

(5) Select the item that best describes your opinion referring to GeneReviews Japan and/or GeneReviews.

GeneReviews（<https://www.ncbi.nlm.nih.gov/books/NBK1116/>）: A comprehensive information site on clinical genetic medicine supported by the NIH and the U.S. Department of Energy.

□Strongly agree □Agree □Neutral □Disagree □Strongly disagree □Unsure

(6) Select the item that best describes your opinion referring to the ClinGen Actionability Summary Report.

ClinGen Actionability Summary Report: One of the working groups of ClinGen (https://clinicalgenome.org/) is a comprehensive knowledge base on genetic medicine supported and operated by the National Institutes of Health (NIH). It was created by the Actionability Working Group, which scores the preventability and treatability of genetic diseases based on evidence.

□Strongly agree □Agree □Neutral □Disagree □Strongly disagree □Unsure

(7) The Fact Sheets were separated by gene.

□Very useful □Useful □Neutral □Not useful □Not very useful □Unsure

**4. Evaluation of the Fact Sheet Contents of each Chapter**

(1) “GENETIC CHANGE AND HEREDITARY CANCER SYNDROME”

□Very useful □Useful □Neutral □Not useful □Not very useful □Unsure

(2) “THE BENEFIT OF CONFIRMATORY TESTING”

□Very useful □Useful □Neutral □Not useful □Not very useful □Unsure

(3) “RISK OF FAMILY MEMBERS”

□Very useful □Useful □Neutral □Not useful □Not very useful □Unsure

(4) “WHAT IS GENETIC COUNSELING?”

□Very useful □Useful □Neutral □Not useful □Not very useful □Unsure

(5) “LIFETIME RISK”

□Very useful □Useful □Neutral □Not useful □Not very useful □Unsure

(6) “RECOMMENDED MANAGEMENT”

□Very useful □Useful □Neutral □Not useful □Not very useful □Unsure

(7) “REFERENCE”

□Very useful □Useful □Neutral □Not useful □Not very useful □Unsure

**5. Free comments: Additional comments, information to be added, and suggestions for improvements in the evaluation of the Fact Sheet.**

**6. Question about yourself**

(1) Department

□Department of Hepatobiliary and Pancreatic Oncology

□Department of Medical Oncology

□Department of Gastrointestinal Oncology

□Department of Experimental Therapeutics

□Department of General Internal Medicine

□Department of Head and Neck Medical Oncology

□Others

(2) Years of clinical experience).

( years)

(3) Japanese Society of Clinical Oncology Board certification

　　□Yes □No

(4) Japanese Board of Cancer Therapy-certified

　　□Yes □No

These were used in the survey.

Thank you for your time and cooperation.
